# Relationship between the tissue-specificity of mouse gene expression and the evolutionary origin and function of the proteins

**DOI:** 10.1186/gb-2005-6-7-r56

**Published:** 2005-06-29

**Authors:** Shiri Freilich, Tim Massingham, Sumit Bhattacharyya, Hannes Ponstingl, Paul A Lyons, Tom C Freeman, Janet M Thornton

**Affiliations:** 1 EMBL-EBI, Wellcome Trust Genome Campus, Cambridge, CB10 1SB, UK; 2 Rosalind Franklin Centre Genomics Research, Wellcome Trust Genome Campus, Cambridge, CB10 1SB, UK

## Abstract

**Background:**

The combination of complete genome sequence information with expression data enables us to characterize the relationship between a protein's evolutionary origin or functional category and its expression pattern. In this study, mouse proteins were assigned into functional and phyletic groups and the gene expression patterns of the different protein groupings were examined by microarray analysis in various mouse tissues.

**Results:**

Our results suggest that the proteins that are universally distributed in all tissues are predominantly enzymes and transporters. In contrast, the tissue-specific set is dominated by regulatory proteins (signal transduction and transcription factors). An increased tendency to tissue-specificity is observed for metazoan-specific proteins. As the composition of the phyletic groups highly correlates with that of the functional groups, the data were tested in order to determine which of the two factors - function or phyletic age - is dominant in shaping the expression profile of a protein. The observed differences in expression patterns of genes between functional groups were found mainly to reflect their different phyletic origin. The connection between tissue specificity and phyletic age cannot be explained by the recent rate of evolution. Finally, although metazoan-specific proteins tend to be tissue-specific compared with phyletically conserved proteins present in all domains of life, many such 'universal' proteins are also tissue-specific.

**Conclusion:**

The minimal cellular transcriptome of the metazoan cell differs from that of the ancestral unicellular eukaryote: new functions were added (metazoan-specific proteins), whilst other functions became specialized and no longer took place in all cells (tissue-specific pre-metazoan proteins).

## Abstract 2

A microaaray analysis of mouse gene expression combined with the proteins functional and phyletic classification suggests that phyletic age (and not function) is the dominant factor shaping the expression profle of a protein.

## Background

Higher animals are characterized by differentiated tissue types, where each tissue has its own unique cellular composition and physiological function. Comparative genomic studies have shown that the evolution of the metazoan lineage involves the expansion of those specific protein families known to participate in cellular communication and transcriptional regulation [1,2]. However, at the cellular level, it is not yet clear how processes taking place in specific tissues relate to similar processes that took place in the ancestral unicellular species. The recent availability of fully sequenced genomes, together with analysis platforms capable of generating 'global' profiles, enables us not only to identify those proteins that are unique to multicellular species but also to examine their contribution to tissue diversity. We can now study the protein content of a mammalian tissue in comparison with the protein content of unicellular organisms. To what extent does the differentiation process involve gaining new functions and to what extent does it involve specialization of pathways that existed in a unicellular ancestor? Will 'young' proteins (that is, proteins that are unique to multicellular species) exhibit a different expression pattern than 'ancient' or universal proteins?

Recent studies have related several characteristics of a protein to its expression profile. Subramanian and Kumar [3] have shown a connection between a protein's phyletic age and the intensity of expression, as measured by the number of expressed sequence tags. Lehner and Fraser [4] showed that protein domains differ in their tendency to be specifically or widely expressed and that many of the tissue-specific domains are metazoan-specific. Tissue-specific genes evolve more rapidly than broadly expressed ones [5-7]. We have studied the relationship between the phyletic age of a protein and its expression profile, and related this to the function of the protein. The term 'phyletic age' used here describes an estimated point in time when a protein integrated into the mouse genome. The universal and eukaryotic specific phyletic groups include proteins that are estimated to be found in the ancestral mouse genome before the transition from unicellularity to multicellularity. The metazoan-specific and mammalian-specific protein groups describe those proteins that are estimated to be integrated into the mouse genome after the transition. As the phyletic protein groups differ in their functions we wanted to determine whether a protein's expression profile better reflects function or age.

Finally, we wanted to verify that the phyletic age of a protein is indeed a major factor in shaping its expression profile rather than merely a reflection of the level of conservation in a protein - a factor that has already been shown to play a role in determining expression [5-7]. To rule out the possibility that the connection between age and expression is spurious due to the misclassification of rapidly evolving genes and the connection between tissue expression and recent rate of evolution, Subramanian and Kumar [3] showed that the connection still exists in a slowly evolving set of data. However, the assumption that the slow rate of evolution of the genes assumes a correct age classification may not hold if there has been a change in rate during their evolutionary history, for example, diversifying selection followed by conservation after a gene duplication [8]. In this paper, we propose a direct test to show that the connection between phyletic age and tissue expression of a gene cannot be explained by the connection between rate and tissue expression alone, a test which does not assume homotachy and makes use of all the available data.

**Figure 1:**
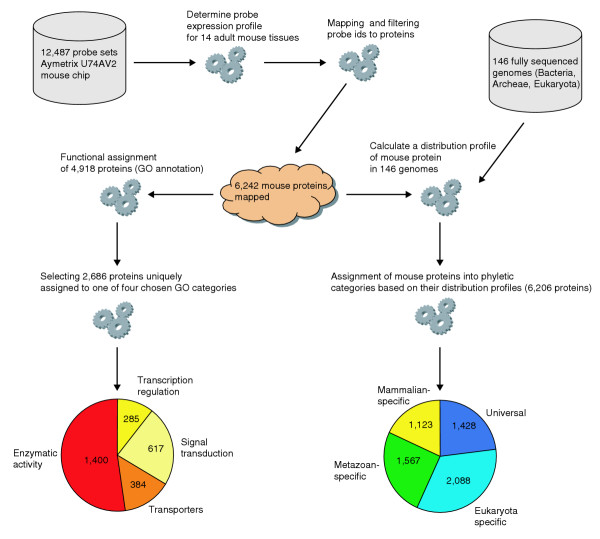
A schematic description of the expression profile determination and protein annotation as described in Materials and methods. The numbers in the pie charts indicate the number of proteins assigned to the relevant category. GO, Gene Ontology.

In order to tackle these questions we have studied expression patterns in 14 mouse tissues. Gene expression patterns (for example, ubiquitous in all tissues examined or tissue-specific) were related to the evolutionary origin of the protein as reflected in the distribution of proteins in different phyla. Firstly, we have assigned mouse proteins to one of four functional categories: two regulatory categories (signal transduction and transcription regulation) and two metabolic categories (enzymes and transporters). Next, the proteins were assigned to a phyletic category: mammalian-specific proteins, metazoan-specific proteins, eukaryote-specific proteins and universal proteins - present in prokaryote species. Then we compared the expression pattern of the different categories within various mouse tissues and studied the tendency of proteins within these groups to be tissue-specific or ubiquitous. The assignment process is described in Figure 1.

## Results

### Comparing expression patterns within different tissues

**Figure 2:**
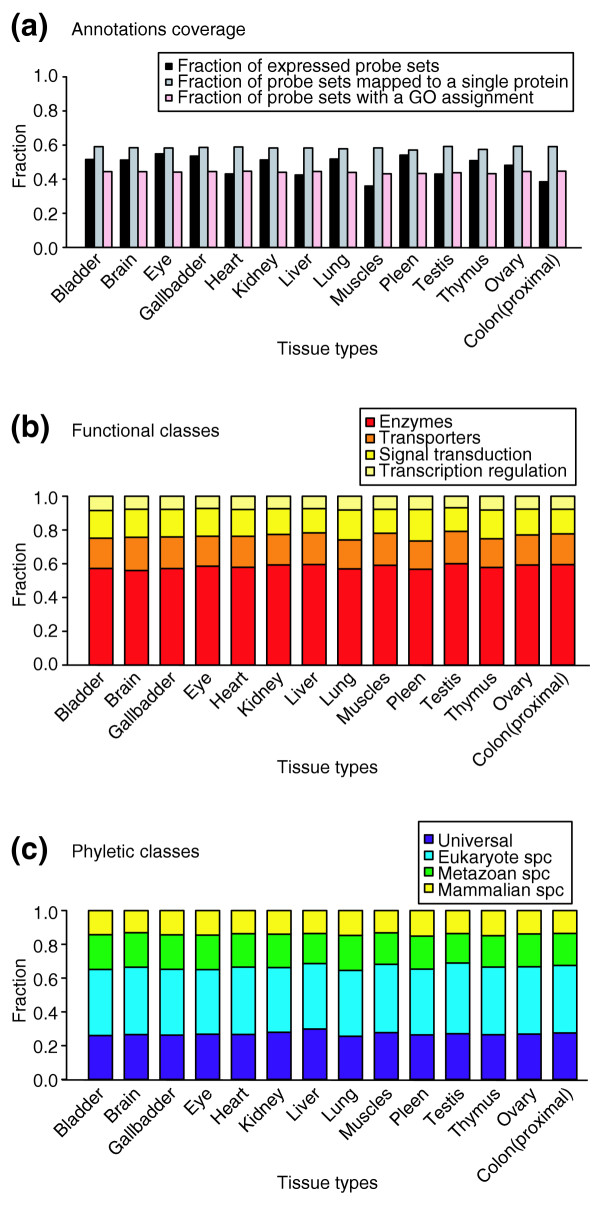
Fraction of expressed probe sets (out of all 12,488 probe sets) in a tissue (black). The grey bars represent the fraction of expressed probe sets in a tissue that can be uniquely mapped to a single protein. **(a) **The pink bars represent the fraction of expressed probe sets in a tissue that are assigned with a GO annotation. **(b) **Distribution of the four functional categories of all annotated proteins expressed in a tissue. **(c) **Distribution of the four phyletic categories of all the mapped proteins expressed in a tissue.

For each tissue we counted the number of expressed probe sets. The fraction of probe sets expressed in each tissue ranges from 0.35 (muscle) to 0.55 (eye). Nearly a constant fraction (~60%) of the probe sets in each tissue is mapped to proteins. Similarly a constant fraction (~45%) of the proteins in each tissue can be assigned a Gene Ontology (GO) annotation (Figure 2a). We compared the tissues for their content of functional and phyletic groups (Figure 2b,c). All tissues display a strikingly similar functional and phyletic composition. The functional composition of annotated proteins in a tissue is approximately 60% enzymes, 20% transporters, 15% signal transduction proteins and 5% transcription regulation proteins. The phyletic composition of proteins in a tissue is found to be approximately 25% universal proteins, 40% eukaryotic-specific proteins, 20% metazoan-specific proteins and 15% mammalian-specific proteins.

**Figure 3:**
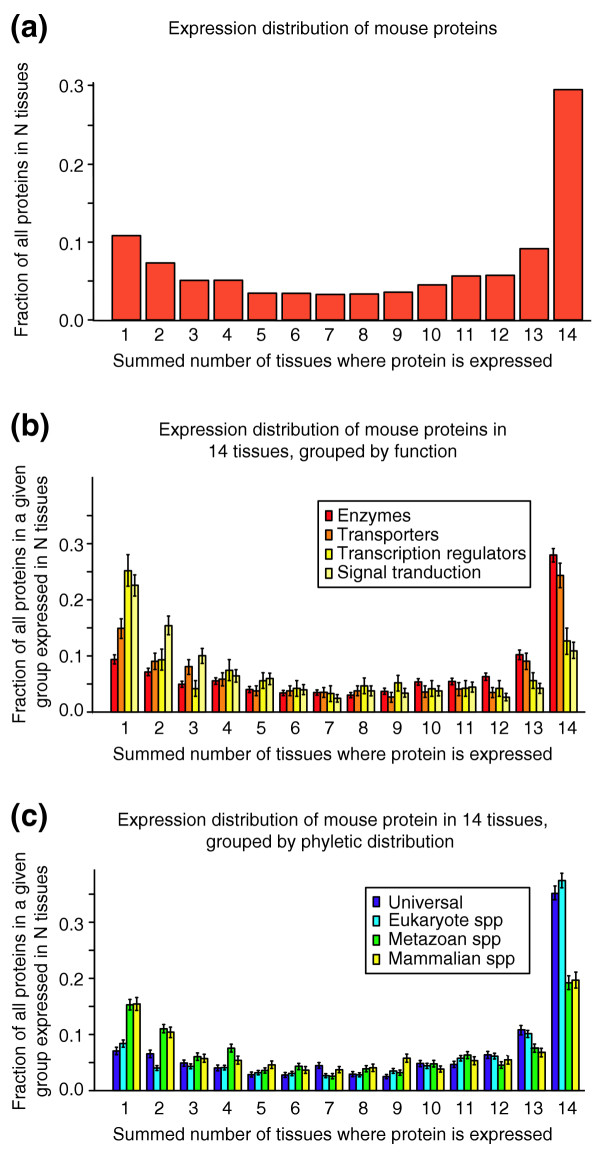
Expression distributions of mouse proteins. **(a) **Expression pattern of all mapped proteins expressed in at least a single tissue (5,528 out of 6,242 proteins). **(b) **Expression pattern of proteins in different functional groups and **(c) **in different phyletic groups. The plot presents the fraction of proteins in a group that are expressed in N tissues. The analysis is restricted to those proteins expressed in at least a single tissue. Sample size (number of assigned proteins): 1,294 enzymes; 343 transporters; 450 signal transduction; 214 transcription regulation; 1,359 universal; 1,951 eukaryote-specific; 1,281 metazoan-specific; and 910 mammalian-specific.

As the tissues seem to have almost identical overall composition of functional categories (Figure 2b), tissue diversity must be achieved through differences in the protein composition within each different category. We counted the number of proteins expressed in one tissue, two tissues, and so on (Figure 3a). About a third of the proteins are expressed in all tissues examined, so variation is seen for two-thirds of the proteins in our sample.

### Comparing expression patterns within functional and phyletic categories

We further studied the contribution of different functional and phyletic groups to tissue variation. Are some functional categories more tissue-specific than others? We examined the expression profile of proteins from the four functional categories within 14 different mouse tissues. For each group, we calculated the fraction of its protein members expressed in one tissue, two tissues, and so on (Figure 3b). Surprisingly, less than one-third of the enzymes and transporters are ubiquitously expressed in all tissues examined. The fraction is even lower for the other functional groups where only about one-tenth of the transcription factors and signal transduction proteins are expressed in all tissues examined. Two different patterns of expression can be observed: the relative abundance of enzymes and transporter proteins is higher among proteins that are ubiquitously expressed; in contrast, a larger fraction of transcription factors and signal transduction proteins are tissue-specific.

Signal transduction proteins and transcription factors are known to be the main functional categories that were expanded in the metazoa lineage while enzymes and transporter proteins are usually more highly conserved between the different domains of life [1,2]. Therefore, unsurprisingly, the distribution of the functional groups in our dataset largely correlates with the phyletic clusters (Table 1). Reproducing the expression data charts using the phyletic groups naturally reveals the trend predicted from Table 1 - the relative abundance of universal and eukaryote-specific proteins is higher among proteins that are expressed in a wide variety of tissues, while mammalian-specific proteins have a higher tendency to be tissue-specific (Figure 3c). The observations are compatible with those obtained in a recent study where metazoan-specific protein domains and protein domains involved in intercellular communication were shown to be tissue-specific [4]. As the expression of the functional (Figure 3b) and phyletic (Figure 3c) groups represent two sides of the same coin, the remaining question is whether enzymes tend to be ubiquitously expressed due to their phyletic-universal origin or whether phyletic-universal proteins tend to be ubiquitously expressed due to being enzymes.

### The inter-relationship between function, 'phyletic age' and expression

In order to identify whether a protein's expression pattern better reflects function or age, the inter-relationship between these three factors was compared statistically. After phyletic age was taken into account, only a weak dependence between function and tissue specificity was detected (test statistic 264.7, *p *value 0.04), suggesting that most of the relationship observed between function and tissue specificity is accounted for by the age of the gene. The relationship between phyletic age and tissue specificity is not explained by a gene's function (test statistic 339.1, *p *< 0.0001), nor is the relationship between phyletic age and function explained by the tissue specificity (test statistic 967.2, *p *<< 0.0001). Therefore, the results imply that enzymes tend to be ubiquitously expressed mainly due to their phyletic universal origin (rather than due to their functional classification).

**Figure 4:**
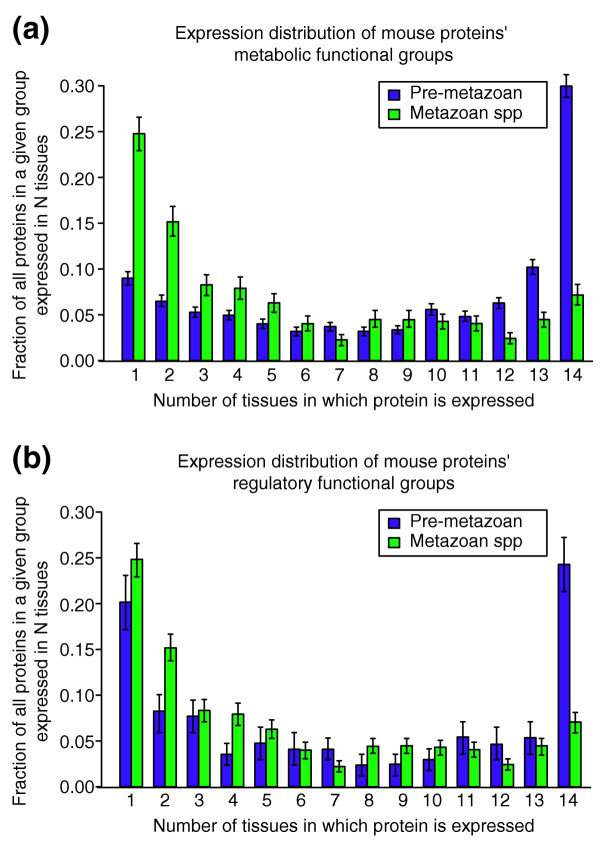
Expression distribution of mouse protein functional groups. **(a)** The metabolic functional group includes enzymes and transporter proteins. **(b)** The regulatory group includes signal transduction and transcription regulation proteins. The pre-metazoa group includes universal proteins and eukaryote specific proteins. The metazoan-specific group includes metazoan-specific and mammalian-specific proteins. The plot presents the fraction of proteins in a group that are expressed in N tissues. The analysis is restricted to those proteins expressed in at least a single tissue. Sample size (number of assigned proteins): pre-metazoa metabolic proteins 1370; metazoan metabolic proteins 267, pre-metazoa regulatory proteins 169; metazoan regulatory proteins 493. The error bars indicate the standard error estimate using bootstrap resampling.

In order to show the extent to which ancient metabolic proteins are widely expressed, or to which young regulatory proteins are tissue-specific, we have divided the functional groups according to their phyletic groups (Figure 4). To have a sufficient sample size for the bootstrap error analysis in Figure 4, we merged the four functional categories into two: metabolism (enzymes and transporters) and regulation (transcription factors and signal transduction). The expression pattern of the two functional categories was examined in two phyletic groups: the pre-metazoan group (universal and eukaryote-specific groups) and the metazoan-specific group (metazoan and mammalian-specific proteins) (Figure 4a,b).

From the expression distribution of metabolic proteins (enzymes and transporters, Figure 4a), one can observe (as can be inferred from the statistical test reported above) obvious differences between the 'older' pre-metazoan proteins (universal and eukaryote-specific groups) and the more recent metazoan proteins. Differences can be observed for the regulatory proteins as well (Figure 4b): metazoan-specific proteins tend to be more tissue-specific compared with pre-metazoan ones, regardless of their functional class. A notable difference between the expression patterns in Figure 4a and 4b occurs for specifically expressed pre-metazoan proteins, where the proportion of regulatory proteins is much higher than metabolic proteins, and it is not significantly different from the fraction of metazoan-specific proteins. This confirms that the function has some influence on expression independent of age but suggests that the effect is stronger in specifically expressed pre-metazoan proteins.

Yet, although pre-metazoan proteins tend to be more widely expressed, less than one-third of the pre-metazoan metabolic proteins are expressed in all tissues. *Ldhc *(testis-specific lactate dehydrogenase) is one example of a universal enzyme whose expression is limited to few cell types in mammals. *Ldh *participates in anaerobic glycolysis - a nearly universal pathway that converts glucose into pyruvate. The sequence of reactions in the pathway is similar in all organisms and in all cell types. In contrast, the fate of pyruvate is variable. In a variety of microorganisms, lactate is normally formed from pyruvate in a reaction catalyzed by *Ldh*. In higher organisms, most cells do not convert pyruvate to lactate and the reaction is limited to few tissues [9]. In germ cells, where lactate is a preferred energy source [10], we observe specific expression of *Ldhc *(testis-specific expression). The expression of *Ldhc *is an example of a function occurring in the ancestral unicellular cell that becomes tissue-specific in multicellular species.

The testis-specific expression of two other universal enzymes in our dataset - glucose-6-phosphate dehydrogenase 2 (*G6pd-2*) and phosphoglycerate kinase 2 (*Pgk-2*) - provides a different example for a specific expression of universal enzymes. *G6pd-2 *and *Pgk-2 *are believed to arise from their isoenzymes, *G6pd *and *Pgk-1*, respectively, by a gene duplication event. *G6pd *and *Pgk-1 *are essential, widely expressed, X chromosome-encoded genes. The absence of those two enzymes during the inactivation of the X chromosome in postmeiotic spermatogenic cells is compensated for by the expression of their autosomal testis-specific isoenzymes *G6pd-2 *and *Pgk-2 *[11,12]. Duplication events can therefore explain some of the cases where universal enzymes are specifically expressed.

### The inter-relationship between 'phyletic age', evolutionary rate and expression

Tissue-specific genes tend to evolve more rapidly than broadly expressed ones [5-7]. Therefore, difficulties might arise in identifying their distant homologs, leading to a correlation between age and rate of evolution. We wanted to verify that the expression patterns observed here cannot be explained purely in terms of variation in the recent rate of evolution, and so we have studied the expression profile of different phyletic groups in a subset of the data where all phyletic groups have evolved at approximately the same rate. For each protein in our dataset we calculated an evolutionary rate by measuring the *K*_a_/*K*_s _ratio with its ortholog in rat (see Methods). The chi-squared test statistic for the independence of age and tissue expression given rate in our data was 226.5, whereas the maximum observed statistic in 10,000 random draws, generated as described in Methods, was 84.3. The connection between phyletic age and tissue specificity that we observed in our data cannot be explained purely in terms of both factors' mutual correlation with the recent rate of evolution.

## Discussion

It is important to remember that our analysis is based only on those proteins that are present on the Affymetrix chip and have GO annotation. Our dataset covers approximately one-quarter of mouse proteins. Clearly, a better coverage for the expression and annotation of proteins is desirable and could change the conclusion presented below. In order to decrease the probability that our results are arbitrary, we repeated the experiment with a different set of tissues (seven components of the gastrointestinal tract). The results obtained are compatible with the observations we report here (data not shown).

We show here that multicellular specific proteins tend to be more tissue-specific than 'ancient' universal proteins. Most of the 'late' evolutionary proteins are transcription factors and signal transduction proteins, categories that have previously been suggested to play a crucial role in tissue differentiation. However, our analysis suggests that more recent enzymes and transporters also contribute to tissue diversity as many of them are tissue-specific (Figure 4a). The selective expression pattern of recent genes implies that a new protein is often selected to perform a tissue-specific function rather than a global one. A greater evolutionary flexibility of tissue-specific proteins is compatible with previous studies suggesting that tissue-specific proteins evolve more rapidly [5-7] due to less strict functional constraints compared with broadly expressed proteins [5,13].

Despite this trend, many metazoan-specific proteins are ubiquitous and many universal proteins are tissue-specific. The minimal cellular transcriptome of the metazoan cell differs from that of the ancestral unicellular eukaryote: new functions were added (metazoan-specific proteins), whilst other functions became specialized and no longer took place in all cells (tissue-specific pre-metazoan proteins). The extent of the cellular specialization can be implied from the observation that only one-third of the proteins are expressed in all tissues examined. In some of these cases, functions occurring in the unicellular cell become tissue-specific in multicellular species. In other cases, universal genes that have been duplicated become specific to a tissue whilst a second copy maintains its original expression pattern. Only about one-third of the pre-metazoan metabolic enzymes are expressed in all tissues. Tissue differentiation is at least in part achieved by tissue specialization of metabolism - either by differentially expressing two-thirds of the ancient metabolic proteins, or by encoding new metabolic proteins. Presumably, the additional transcription-related proteins provide the necessary control. We aim to further characterize the expression patterns of processes that exist in the ancestral metazoa and those that are specific to metazoa. In particular, we are interested in studying the contribution of function differentiation versus gene duplication to tissue diversity in multicellular species.

## Materials and methods

### Expression profile determination

Tissues were dissected and snap frozen from 8-12-week old C57/BL6 male mice with the exception of the ovaries, which were taken from females. Tissue was pooled from between four to six animals, the RNA extracted and 10 mg of total RNA was labeled and then hybridized to the Affymetrix U74AV2 GeneChip using standard protocols (Affymetrix Inc, CA, USA); complete experimental details for each of these stages are given at [14]. Expression values and presence/absence flags were generated from the CEL files using the Microarray Suite 5.0 package (Affymetrix MAS 5.0) and its default settings. The global scaling normalization method operated with a target value setting of 100. Expression data flagged with a marginal call was excluded from further analysis. The quality of the microarray data was assessed first using the parameters defined in the report file generated by MAS 5.0, next through recording the percentage of outliers reported by dCHIP on calculating expression values [15] and finally by using the Affy package in the BioConductor suite of microarray analysis programs to generate degradation plots [16]. Chips failing the quality control parameters recommended by the authors of these programs were omitted from further analysis. The microarray data (accession ID = E-HGMP-2) used in this study is now available to download from ArrayExpress [17].

A subset of 14 samples representing distinct non-redundant organs was chosen out of the complete dataset. The tissues are listed in Figure 2. The use of different cut-offs within the range of 0.025 <*P *value < 0.075 for absent/present flags labeling has no effect on the analysis. Only absent/present calls were used to define tissue-specificity and expression levels have not been a factor in this analysis.

### Mapping probe sets to mouse proteins

Out of 12,487 probe sets, 8,218 were mapped into EnsEmbl mouse transcripts using Ensmart [18] (version 13.1, [19]). Mapping of EnsEmbl transcripts to SWISS-PROT [20] (release 41.25) and TrEmbl proteins (Release 24.13) were obtained from the International Protein Index (IPI, [21]). Proteins represented by more than a single probe set were discarded in order to avoid re-counting. Different proteins sharing the same probe sets were also eliminated (with the exception of splice variants). A single and unique probe set therefore represents each of the 6,242 remaining proteins.

### Mouse protein functional annotation

A total of 4,918 proteins were assigned with a GO annotation [22]. For simplicity and in order to avoid overlaps between the functional categories, we studied the tissue distribution of proteins assigned to four main categories from the highest hierarchy level of the functional classification: enzymatic activity, transporters, transcription regulation and signal transduction. Proteins assigned to more than a single category were discarded, leaving 2,686 proteins distributed as follows: 1,400 enzymes, 384 transporters, 617 proteins involved in signal transduction and 285 proteins that regulate transcription. The functional assignments are available from [23].

### Mouse protein phyletic assignment

We used four categories to describe the evolutionary origin of mouse proteins: universal proteins, that is, ubiquitous in the three domains of life (bacteria, archaea and eukaryotes), eukaryote-specific proteins, metazoan-specific proteins and mammalian-specific proteins. The 6,242 mouse proteins were classified into the phyletic categories according to the results of a BLAST [24] search against 146 fully sequenced species. A protein could only be assigned to a single category. The classification process is hierarchical: proteins with hits to more than five prokaryote species are classified as universal; the remaining mouse proteins with at least a single hit to non-metazoan eukaryotes are classified as eukaryote-specific; the remaining mouse proteins with at least a single hit to non-mammalian metazoa are classified as metazoan-specific; and, finally, proteins recognizing only other mammalian proteins are classified as mammalian-specific. The cut-off used was BLAST e-score < 1e-3. Genomes were downloaded from the COGENT [25] database (release 152).

The observations reported here are maintained using different cut-offs within the range of 1e-10 < e-score < 1e-1. The observations are also maintained when a universal protein is defined as a protein with a hit in at least a single prokaryote species or when it defined as a protein with a hit in at least ten prokaryote species (examined under e-value cut-off of e-score < 1e-3). Additional tests were performed in order to assure that the phyletic distribution truly describes a complete sequence distribution rather than domain distribution. The classification of a protein to a phyletic group was done when additional filters were added. This was in order to discard those cases where a match between query and hit is based only on recognition of a conserved domain rather than a complete sequence (e-score <1e-3). Firstly, pfam domain composition: all query-hit pairs that do not share an identical pfam domain composition were discarded from our dataset. Secondly, full coverage: all query-hit pairs where the alignment does not cover the full length (80%) of both proteins were discarded from our dataset.

When repeating the analysis with the filtered data, the results confirm that the trends reported here are maintained (not shown). Similar results were also obtained when using the homologous clusters database STRING [26] for a phyletic classification (universal proteins have to be recognized in at least five prokaryote species). The 6,242 proteins are distributed in phyletic categories as follows: 1,428 universal proteins, 2,088 eukaryote specific proteins, 1,567 metazoan proteins, 1,123 mammalian-specific proteins; and 36 unclassified proteins. The phyletic assignments are available from [23].

### *K*_a_/*K*_s _values

Mouse and rat 1:1 ortholog pairs were obtained from EnsEmbl [16]. In those cases where a mouse protein had more than a single rat ortholog it was discarded from the analysis unless one of the ortholog pairs was annotated as 'best reciprocal hit' (BRH). The ratio of K_a _(the number of non-synonymous substitutions per non-synonymous site) to *K*_s _(the number of synonymous substitutions per synonymous site) was calculated using the codeml program from the PAML 3.13d package [27]. Two sequences had one or fewer nucleotide mutations and so their *K*_a_/*K*_s _ratio could not be reliably estimated. These sequences were discarded from the analysis. In total, the *K*_a_/*K*_s _ratio was calculated for 4,056 mouse proteins from the 5,501 proteins classified to a phyletic category and expressed in at least a single tissue (as shown in Figure 3c).

### Statistical tests

By grouping the genes into equal bins of similar recent rates of evolution (*K*_a_/*K*_s _values) and then shuffling the phyletic ages within each group, sample sets of data can be created which have no connection between phyletic age and tissue expression other than through their common connection to recent rate of evolution. The connection between rate and tissue specificity in each of these sample sets is identical to the observed data and, because all genes in each bin have a similar rate of evolution, the connection between age and rate is similar to the observed data. By generating random samples in the manner as described above, the expected contingency table of age/expression dependence and the null distribution of the chi-squared test statistic can be estimated. Using the estimated expected table, the chi-squared statistic for the observed data can be calculated. The significance of the observation was assessed by comparing the observed test statistic with those from 10,000 sets of data, of equal size to the observed data, randomly generated according to the expected contingency table (and so satisfying the null hypothesis).

The relationship between tissue specificity and phyletic age and function was investigated using a contingency table test under the null hypothesis that function and specificity are independent of given age. Conceptually, the genes are divided up according to age and a separate contingency table for specificity and function is formed for each group. The chi-squared test statistic [25] for independence between function and specificity is calculated for each table and then pooled, weighted by the proportion of genes of each age, to give the test statistic for independence between function and specificity given age. The dependence between specificity and age given function, and age and function given specificity, were calculated similarly. The tables analyzed had cells expected to contain a small number of observations, so it was inappropriate to assess the significance of the test statistic using tables of pre-calculated critical values. Instead, 10,000 sets of data, of equal size to that observed, were generated in accordance to the expected contingency table and the test statistics of these were used to form an estimate of their distribution under the null hypothesis, to which the observed test statistic can be compared.

## Additional data files

The following additional data are available with the online version of this paper. Additional data file 1 contains the functional assignments of the proteins used in the analysis. Additional data file 2 contains the phyletic assignments of the proteins used in the analysis. Additional data file 3 contains the sequences of the proteins used in the analysis.

## Supplementary Material

Additional File 1 Functional assignments of the proteins used in the analysis. Functional assignments of the proteins used in the analysis Click here for file

Additional File 2 Phyletic assignments of the proteins used in the analysis. Phyletic assignments of the proteins used in the analysis Click here for file

Additional File 3 The sequences of the proteins used in the analysis. The sequences of the proteins used in the analysis Click here for file

## Figures and Tables

**Table 1:** The distribution of function within the phyletic groups

Phyletic groups/functional groups	Total functionally annotated proteins (%)	Enzymes, %	Transporters, %	Transcription factors, %	Signal transduction, %
Universal	833 (31%)	82	15	2	1
Eukaryote-specific	823 (31%)	60	18	12	10
Metazoan-specific	656 (24%)	26	7	23	44
Mammalian-specific	372 (14%)	13	17	6	64
All	2,684	52	14	11	23
