# Supplementary material for: Relationship between the tissue-specificity of mouse gene expression and the evolutionary origin and function of the proteins
Source: Genome Biol. 2005 Jun 29;6(7):R56. doi: 10.1186/gb-2005-6-7-r56 (PMC1175987; doi:10.1186/gb-2005-6-7-r56)
Supplement: Additional File 1 — Functional assignments of the proteins used in the analysis. Functional assignments of the proteins used in the analysis [file gb-2005-6-7-r56-S1.htm]

#### Legend

*E - enzymes   
T - transporters   
ST - proteins involved in
signal transduction   
TR - proteins that regulate transcription* 


|  |  |  |
| --- | --- | --- |
| **EnsembleProtein** | **Affymetrix\_ID** | **Functional\_assignment** |
| ENSMUSP00000021726 | 98435\_at | E |
| ENSMUSP00000015998 | 93445\_at | ST |
| ENSMUSP00000040343 | 97923\_at | E |
| ENSMUSP00000053446 | 94838\_r\_at | ST |
| ENSMUSP00000045973 | 97122\_at | TR |
| ENSMUSP00000003626 | 161297\_f\_at | ST |
| ENSMUSP00000036159 | 101282\_at | ST |
| ENSMUSP00000028764 | 94775\_at | ST |
| ENSMUSP00000001964 | 160927\_at | E |
| ENSMUSP00000027264 | 97797\_at | E |
| ENSMUSP00000002126 | 92674\_at | TR |
| ENSMUSP00000043427 | 97809\_at | E |
| ENSMUSP00000047379 | 102348\_at | E |
| ENSMUSP00000033184 | 93595\_at | E |
| ENSMUSP00000024956 | 104725\_at | E |
| ENSMUSP00000025826 | 92950\_at | T |
| ENSMUSP00000020546 | 102624\_at | ST |
| ENSMUSP00000053307 | 99373\_at | T |
| ENSMUSP00000031084 | 98994\_at | T |
| ENSMUSP00000028358 | 93871\_at | ST |
| ENSMUSP00000020507 | 95316\_at | ST |
| ENSMUSP00000057513 | 96760\_at | T |
| ENSMUSP00000062979 | 93070\_at | T |
| ENSMUSP00000036761 | 98338\_at | TR |
| ENSMUSP00000021203 | 93348\_at | T |
| ENSMUSP00000049002 | 94937\_at | T |
| ENSMUSP00000025891 | 100932\_at | E |
| ENSMUSP00000025377 | 92691\_at | E |
| ENSMUSP00000031263 | 93624\_at | T |
| ENSMUSP00000050343 | 101185\_at | TR |
| ENSMUSP00000002180 | 102641\_at | TR |
| ENSMUSP00000029653 | 102774\_at | ST |
| ENSMUSP00000032886 | 99402\_at | E |
| ENSMUSP00000022570 | 99390\_at | ST |
| ENSMUSP00000018287 | 102919\_at | TR |
| ENSMUSP00000036983 | 98079\_at | E |
| ENSMUSP00000026415 | 94412\_at | E |
| ENSMUSP00000023599 | 93089\_at | E |
| ENSMUSP00000004971 | 160552\_at | T |
| ENSMUSP00000016033 | 100540\_at | E |
| ENSMUSP00000027237 | 95333\_at | ST |
| ENSMUSP00000034537 | 95599\_at | E |
| ENSMUSP00000040797 | 99811\_at | ST |
| ENSMUSP00000024892 | 104628\_at | E |
| ENSMUSP00000024047 | 94020\_at | E |
| ENSMUSP00000025292 | 100559\_at | E |
| ENSMUSP00000024796 | 97175\_at | E |
| ENSMUSP00000057085 | 92185\_at | E |
| ENSMUSP00000031101 | 98909\_at | E |
| ENSMUSP00000025196 | 102791\_at | E |
| ENSMUSP00000034830 | 98108\_at | T |
| ENSMUSP00000034466 | 94562\_at | E |
| ENSMUSP00000000219 | 100690\_at | E |
| ENSMUSP00000031390 | 92461\_at | E |
| ENSMUSP00000029837 | 92606\_at | E |
| ENSMUSP00000038783 | 97729\_at | E |
| ENSMUSP00000025180 | 95350\_at | T |
| ENSMUSP00000022599 | 95628\_at | E |
| ENSMUSP00000040522 | 101623\_at | T |
| ENSMUSP00000015278 | 98372\_at | E |
| ENSMUSP00000023132 | 93382\_at | E |
| ENSMUSP00000033276 | 97993\_at | E |
| ENSMUSP00000025019 | 102677\_at | ST |
| ENSMUSP00000033098 | 100443\_at | E |
| ENSMUSP00000005583 | 100576\_at | E |
| ENSMUSP00000025844 | 92214\_at | E |
| ENSMUSP00000033730 | 98781\_at | ST |
| ENSMUSP00000051187 | 103332\_at | ST |
| ENSMUSP00000019470 | 93803\_at | E |
| ENSMUSP00000022264 | 93936\_at | E |
| ENSMUSP00000036372 | 102820\_at | E |
| ENSMUSP00000045085 | 93268\_at | E |
| ENSMUSP00000034488 | 94724\_at | E |
| ENSMUSP00000013990 | 99847\_at | E |
| ENSMUSP00000020528 | 94857\_at | E |
| ENSMUSP00000058855 | 92623\_at | E |
| ENSMUSP00000062412 | 103741\_at | TR |
| ENSMUSP00000020765 | 98155\_r\_at | E |
| ENSMUSP00000019930 | 95512\_at | E |
| ENSMUSP00000000326 | 94189\_at | TR |
| ENSMUSP00000002678 | 101918\_at | ST |
| ENSMUSP00000045150 | 103218\_at | T |
| ENSMUSP00000040035 | 104662\_at | ST |
| ENSMUSP00000063038 | 96433\_at | E |
| ENSMUSP00000063117 | 99322\_at | T |
| ENSMUSP00000009789 | 104139\_at | E |
| ENSMUSP00000019332 | 96117\_r\_at | E |
| ENSMUSP00000057212 | 98810\_at | ST |
| ENSMUSP00000021948 | 92509\_at | E |
| ENSMUSP00000020586 | 92497\_at | T |
| ENSMUSP00000058354 | 101526\_at | TR |
| ENSMUSP00000055886 | 101659\_at | E |
| ENSMUSP00000020118 | 93285\_at | E |
| ENSMUSP00000045118 | 94741\_at | E |
| ENSMUSP00000031633 | 101639\_r\_at | E |
| ENSMUSP00000016094 | 97763\_at | E |
| ENSMUSP00000034131 | 92640\_at | T |
| ENSMUSP00000018710 | 102314\_at | T |
| ENSMUSP00000033820 | 92918\_at | E |
| ENSMUSP00000003790 | 94073\_at | E |
| ENSMUSP00000029938 | 160225\_at | TR |
| ENSMUSP00000022545 | 100346\_at | ST |
| ENSMUSP00000056004 | 100479\_at | E |
| ENSMUSP00000039239 | 101802\_at | ST |
| ENSMUSP00000033939 | 93706\_at | E |
| ENSMUSP00000041696 | 98829\_at | T |
| ENSMUSP00000031843 | 103235\_at | ST |
| ENSMUSP00000040089 | 98028\_at | TR |
| ENSMUSP00000034207 | 102989\_at | T |
| ENSMUSP00000054665 | 104023\_at | ST |
| ENSMUSP00000003877 | 160501\_at | T |
| ENSMUSP00000045955 | 99617\_at | E |
| ENSMUSP00000038925 | 100755\_at | E |
| ENSMUSP00000003770 | 97516\_at | E |
| ENSMUSP00000033330 | 93970\_at | T |
| ENSMUSP00000019422 | 103644\_at | E |
| ENSMUSP00000047359 | 102788\_s\_at | TR |
| ENSMUSP00000062096 | 98304\_at | ST |
| ENSMUSP00000023897 | 102995\_s\_at | E |
| ENSMUSP00000001347 | 104432\_at | E |
| ENSMUSP00000028624 | 96336\_at | E |
| ENSMUSP00000062186 | 92790\_at | T |
| ENSMUSP00000018273 | 97925\_at | E |
| ENSMUSP00000006761 | 92935\_at | TR |
| ENSMUSP00000029076 | 160375\_at | E |
| ENSMUSP00000057112 | 100496\_at | E |
| ENSMUSP00000018313 | 100508\_at | E |
| ENSMUSP00000016602 | 93856\_at | TR |
| ENSMUSP00000029367 | 94644\_at | E |
| ENSMUSP00000049132 | 104318\_at | T |
| ENSMUSP00000031314 | 94777\_at | T |
| ENSMUSP00000000419 | 102809\_s\_at | E |
| ENSMUSP00000037146 | 92543\_at | E |
| ENSMUSP00000025856 | 103806\_at | ST |
| ENSMUSP00000028138 | 97983\_s\_at | T |
| ENSMUSP00000045606 | 103939\_at | E |
| ENSMUSP00000035116 | 160128\_at | E |
| ENSMUSP00000055603 | 98321\_at | ST |
| ENSMUSP00000021045 | 99910\_at | T |
| ENSMUSP00000021333 | 161049\_at | TR |
| ENSMUSP00000057843 | 100525\_at | E |
| ENSMUSP00000022697 | 98730\_at | ST |
| ENSMUSP00000058691 | 92429\_at | ST |
| ENSMUSP00000026858 | 98996\_at | E |
| ENSMUSP00000036265 | 103414\_at | E |
| ENSMUSP00000013419 | 103547\_at | T |
| ENSMUSP00000002663 | 96895\_at | E |
| ENSMUSP00000056822 | 102902\_at | TR |
| ENSMUSP00000028623 | 99929\_at | E |
| ENSMUSP00000031645 | 160946\_at | E |
| ENSMUSP00000034588 | 96094\_at | T |
| ENSMUSP00000034128 | 96106\_at | E |
| ENSMUSP00000023465 | 103823\_at | E |
| ENSMUSP00000055531 | 94005\_at | E |
| ENSMUSP00000023677 | 99128\_at | E |
| ENSMUSP00000053554 | 100133\_at | E |
| ENSMUSP00000051726 | 103288\_at | TR |
| ENSMUSP00000033015 | 96515\_at | E |
| ENSMUSP00000022295 | 160590\_r\_at | E |
| ENSMUSP00000014848 | 102643\_at | TR |
| ENSMUSP00000029905 | 99404\_at | E |
| ENSMUSP00000033516 | 94414\_at | E |
| ENSMUSP00000031478 | 161060\_i\_at | E |
| ENSMUSP00000032165 | 99537\_at | E |
| ENSMUSP00000038983 | 98880\_at | E |
| ENSMUSP00000046640 | 97436\_at | E |
| ENSMUSP00000039705 | 100951\_at | T |
| ENSMUSP00000059782 | 96256\_at | E |
| ENSMUSP00000027086 | 97712\_at | E |
| ENSMUSP00000025862 | 102384\_at | E |
| ENSMUSP00000027991 | 94155\_at | ST |
| ENSMUSP00000026178 | 160307\_at | E |
| ENSMUSP00000034902 | 101872\_at | E |
| ENSMUSP00000027238 | 98500\_at | ST |
| ENSMUSP00000019374 | 93643\_at | TR |
| ENSMUSP00000020272 | 96665\_at | T |
| ENSMUSP00000001708 | 102660\_at | TR |
| ENSMUSP00000019009 | 94564\_at | E |
| ENSMUSP00000030693 | 94697\_at | E |
| ENSMUSP00000055611 | 104238\_at | E |
| ENSMUSP00000004295 | 97320\_at | T |
| ENSMUSP00000029610 | 95485\_at | E |
| ENSMUSP00000058773 | 101625\_at | T |
| ENSMUSP00000043369 | 98374\_at | ST |
| ENSMUSP00000049338 | 93251\_at | E |
| ENSMUSP00000026262 | 94840\_at | E |
| ENSMUSP00000034140 | 93529\_at | ST |
| ENSMUSP00000035065 | 104647\_at | E |
| ENSMUSP00000030773 | 94172\_at | E |
| ENSMUSP00000028783 | 97206\_at | E |
| ENSMUSP00000037337 | 103201\_at | E |
| ENSMUSP00000042410 | 93938\_at | T |
| ENSMUSP00000013773 | 96827\_at | E |
| ENSMUSP00000059295 | 101499\_at | E |
| ENSMUSP00000015587 | 102822\_at | E |
| ENSMUSP00000019266 | 104388\_at | ST |
| ENSMUSP00000037279 | 94726\_at | ST |
| ENSMUSP00000033004 | 102021\_at | ST |
| ENSMUSP00000030251 | 103610\_at | E |
| ENSMUSP00000021217 | 92625\_at | E |
| ENSMUSP00000005907 | 102287\_at | ST |
| ENSMUSP00000028846 | 92758\_at | E |
| ENSMUSP00000043616 | 101775\_at | TR |
| ENSMUSP00000007156 | 98391\_at | E |
| ENSMUSP00000025271 | 103075\_at | TR |
| ENSMUSP00000006611 | 92540\_f\_at | E |
| ENSMUSP00000049872 | 96435\_at | TR |
| ENSMUSP00000035092 | 102430\_at | ST |
| ENSMUSP00000062147 | 104008\_at | TR |
| ENSMUSP00000030578 | 100595\_at | E |
| ENSMUSP00000003453 | 100607\_at | E |
| ENSMUSP00000035743 | 97489\_at | E |
| ENSMUSP00000033466 | 98812\_at | ST |
| ENSMUSP00000025223 | 93225\_s\_at | E |
| ENSMUSP00000031523 | 92499\_at | TR |
| ENSMUSP00000025338 | 98011\_at | ST |
| ENSMUSP00000004955 | 160750\_at | E |
| ENSMUSP00000029078 | 92642\_at | E |
| ENSMUSP00000048183 | 102316\_at | E |
| ENSMUSP00000034933 | 103905\_at | E |
| ENSMUSP00000021177 | 95664\_at | T |
| ENSMUSP00000025403 | 98420\_at | E |
| ENSMUSP00000025001 | 97109\_at | E |
| ENSMUSP00000056033 | 93430\_at | ST |
| ENSMUSP00000035649 | 161015\_at | T |
| ENSMUSP00000033765 | 96585\_at | ST |
| ENSMUSP00000030047 | 101136\_at | ST |
| ENSMUSP00000059592 | 92636\_f\_at | T |
| ENSMUSP00000047480 | 102725\_at | T |
| ENSMUSP00000033959 | 99474\_at | E |
| ENSMUSP00000054303 | 160491\_at | E |
| ENSMUSP00000005057 | 104025\_at | E |
| ENSMUSP00000028036 | 100757\_at | T |
| ENSMUSP00000048684 | 97518\_at | E |
| ENSMUSP00000046097 | 92528\_at | ST |
| ENSMUSP00000013737 | 93972\_at | T |
| ENSMUSP00000032273 | 103513\_at | ST |
| ENSMUSP00000028207 | 103646\_at | E |
| ENSMUSP00000020824 | 98439\_at | E |
| ENSMUSP00000033023 | 102200\_at | T |
| ENSMUSP00000001595 | 99082\_at | E |
| ENSMUSP00000062402 | 94104\_at | E |
| ENSMUSP00000025652 | 100365\_at | T |
| ENSMUSP00000053922 | 93725\_at | ST |
| ENSMUSP00000047646 | 93858\_at | ST |
| ENSMUSP00000038915 | 96747\_at | E |
| ENSMUSP00000049532 | 101286\_at | T |
| ENSMUSP00000051838 | 99503\_at | E |
| ENSMUSP00000023691 | 99491\_at | ST |
| ENSMUSP00000018700 | 104175\_at | T |
| ENSMUSP00000032555 | 160653\_at | T |
| ENSMUSP00000003569 | 97402\_at | E |
| ENSMUSP00000035052 | 97390\_at | E |
| ENSMUSP00000022195 | 102074\_at | TR |
| ENSMUSP00000055565 | 103663\_at | E |
| ENSMUSP00000034612 | 92678\_at | E |
| ENSMUSP00000027961 | 101562\_at | E |
| ENSMUSP00000034723 | 101707\_at | E |
| ENSMUSP00000057301 | 98323\_at | ST |
| ENSMUSP00000043198 | 98456\_at | E |
| ENSMUSP00000051965 | 93466\_at | T |
| ENSMUSP00000062320 | 104729\_at | T |
| ENSMUSP00000048152 | 160861\_s\_at | E |
| ENSMUSP00000023885 | 99377\_at | T |
| ENSMUSP00000015664 | 160406\_at | E |
| ENSMUSP00000022246 | 95976\_at | ST |
| ENSMUSP00000009732 | 100382\_at | E |
| ENSMUSP00000027696 | 98732\_at | TR |
| ENSMUSP00000022996 | 98865\_at | E |
| ENSMUSP00000040315 | 103416\_at | E |
| ENSMUSP00000033076 | 101629\_s\_at | T |
| ENSMUSP00000063136 | 101170\_at | ST |
| ENSMUSP00000033157 | 96909\_at | E |
| ENSMUSP00000030768 | 102892\_at | T |
| ENSMUSP00000026900 | 101448\_at | E |
| ENSMUSP00000041560 | 102904\_at | ST |
| ENSMUSP00000041475 | 160670\_at | ST |
| ENSMUSP00000022684 | 160948\_at | E |
| ENSMUSP00000025749 | 103825\_at | E |
| ENSMUSP00000022296 | 92660\_f\_at | E |
| ENSMUSP00000060724 | 101658\_f\_at | ST |
| ENSMUSP00000030859 | 101857\_at | E |
| ENSMUSP00000021550 | 98473\_at | E |
| ENSMUSP00000054198 | 93483\_at | E |
| ENSMUSP00000026546 | 103024\_at | E |
| ENSMUSP00000027503 | 98618\_at | E |
| ENSMUSP00000018401 | 93628\_at | ST |
| ENSMUSP00000002809 | 104746\_at | E |
| ENSMUSP00000026060 | 96517\_at | E |
| ENSMUSP00000034385 | 99394\_at | E |
| ENSMUSP00000053027 | 99406\_at | E |
| ENSMUSP00000029942 | 94416\_at | E |
| ENSMUSP00000035982 | 93103\_at | E |
| ENSMUSP00000041557 | 104221\_at | T |
| ENSMUSP00000038170 | 99815\_at | ST |
| ENSMUSP00000006586 | 160164\_at | E |
| ENSMUSP00000028384 | 98502\_at | E |
| ENSMUSP00000038483 | 98768\_at | ST |
| ENSMUSP00000044004 | 101073\_at | ST |
| ENSMUSP00000018702 | 102662\_at | ST |
| ENSMUSP00000043077 | 99423\_at | E |
| ENSMUSP00000025580 | 97322\_at | ST |
| ENSMUSP00000024159 | 92332\_at | TR |
| ENSMUSP00000049803 | 101482\_at | E |
| ENSMUSP00000020408 | 98110\_at | E |
| ENSMUSP00000020362 | 99832\_at | T |
| ENSMUSP00000021197 | 94842\_at | E |
| ENSMUSP00000023214 | 104371\_at | E |
| ENSMUSP00000001780 | 100970\_at | E |
| ENSMUSP00000058637 | 97731\_at | E |
| ENSMUSP00000033952 | 97997\_at | ST |
| ENSMUSP00000033484 | 160181\_at | T |
| ENSMUSP00000032816 | 95630\_at | E |
| ENSMUSP00000034304 | 101891\_at | E |
| ENSMUSP00000036461 | 93795\_at | E |
| ENSMUSP00000027760 | 92671\_f\_at | ST |
| ENSMUSP00000057161 | 96829\_at | T |
| ENSMUSP00000048635 | 101682\_f\_at | T |
| ENSMUSP00000020461 | 93006\_at | TR |
| ENSMUSP00000058198 | 93139\_at | E |
| ENSMUSP00000027444 | 160602\_at | E |
| ENSMUSP00000002064 | 104124\_at | E |
| ENSMUSP00000003502 | 160868\_at | E |
| ENSMUSP00000023752 | 103612\_at | T |
| ENSMUSP00000052119 | 94717\_f\_at | ST |
| ENSMUSP00000022196 | 103878\_at | T |
| ENSMUSP00000031268 | 160067\_at | E |
| ENSMUSP00000045127 | 95516\_at | E |
| ENSMUSP00000006391 | 98405\_at | E |
| ENSMUSP00000047949 | 102380\_s\_at | TR |
| ENSMUSP00000054588 | 93270\_at | E |
| ENSMUSP00000033012 | 94992\_at | T |
| ENSMUSP00000027059 | 92903\_at | TR |
| ENSMUSP00000054426 | 94191\_at | ST |
| ENSMUSP00000025992 | 94203\_at | TR |
| ENSMUSP00000030530 | 99326\_at | E |
| ENSMUSP00000048222 | 94469\_at | E |
| ENSMUSP00000021359 | 101920\_at | E |
| ENSMUSP00000019672 | 100597\_at | E |
| ENSMUSP00000027144 | 93824\_at | E |
| ENSMUSP00000047518 | 92368\_at | ST |
| ENSMUSP00000028881 | 103486\_at | ST |
| ENSMUSP00000025833 | 96713\_at | E |
| ENSMUSP00000027639 | 102974\_at | ST |
| ENSMUSP00000043477 | 102318\_at | E |
| ENSMUSP00000011323 | 160084\_at | E |
| ENSMUSP00000060517 | 101806\_at | ST |
| ENSMUSP00000029029 | 93271\_s\_at | ST |
| ENSMUSP00000001092 | 93432\_at | T |
| ENSMUSP00000041175 | 104550\_at | E |
| ENSMUSP00000021968 | 92920\_at | TR |
| ENSMUSP00000057989 | 102727\_at | ST |
| ENSMUSP00000055458 | 99343\_at | TR |
| ENSMUSP00000026407 | 160493\_at | T |
| ENSMUSP00000021038 | 100759\_at | T |
| ENSMUSP00000031093 | 92252\_at | ST |
| ENSMUSP00000042174 | 96730\_at | E |
| ENSMUSP00000051349 | 93040\_at | T |
| ENSMUSP00000057094 | 93173\_at | T |
| ENSMUSP00000049070 | 98296\_at | ST |
| ENSMUSP00000028360 | 104436\_at | ST |
| ENSMUSP00000021268 | 97784\_at | E |
| ENSMUSP00000046103 | 102335\_at | T |
| ENSMUSP00000033847 | 103924\_at | E |
| ENSMUSP00000037358 | 95550\_at | T |
| ENSMUSP00000005497 | 97128\_at | ST |
| ENSMUSP00000025581 | 103256\_at | ST |
| ENSMUSP00000022971 | 104712\_at | TR |
| ENSMUSP00000014678 | 100674\_f\_at | ST |
| ENSMUSP00000023364 | 161034\_at | E |
| ENSMUSP00000053295 | 101155\_at | TR |
| ENSMUSP00000015581 | 102877\_at | E |
| ENSMUSP00000022269 | 94370\_at | ST |
| ENSMUSP00000062820 | 94515\_at | E |
| ENSMUSP00000010348 | 104044\_at | T |
| ENSMUSP00000021201 | 160655\_at | E |
| ENSMUSP00000028056 | 95000\_g\_at | E |
| ENSMUSP00000019323 | 93991\_at | E |
| ENSMUSP00000004480 | 95436\_at | ST |
| ENSMUSP00000026406 | 101431\_at | E |
| ENSMUSP00000022059 | 101709\_at | ST |
| ENSMUSP00000034992 | 93202\_at | E |
| ENSMUSP00000042124 | 160931\_at | E |
| ENSMUSP00000025867 | 97813\_at | TR |
| ENSMUSP00000006660 | 97946\_at | ST |
| ENSMUSP00000020828 | 102352\_at | E |
| ENSMUSP00000047483 | 92956\_at | ST |
| ENSMUSP00000015858 | 160396\_at | TR |
| ENSMUSP00000028298 | 160408\_at | ST |
| ENSMUSP00000048904 | 100384\_at | TR |
| ENSMUSP00000032937 | 93611\_at | TR |
| ENSMUSP00000015771 | 96500\_at | TR |
| ENSMUSP00000055806 | 99522\_at | E |
| ENSMUSP00000026818 | 99655\_at | E |
| ENSMUSP00000029172 | 100938\_at | ST |
| ENSMUSP00000041118 | 92697\_at | TR |
| ENSMUSP00000032207 | 95320\_at | ST |
| ENSMUSP00000000364 | 101581\_at | E |
| ENSMUSP00000020015 | 101726\_at | ST |
| ENSMUSP00000030136 | 101859\_at | T |
| ENSMUSP00000015791 | 99931\_at | ST |
| ENSMUSP00000005233 | 94941\_at | E |
| ENSMUSP00000023934 | 101869\_s\_at | T |
| ENSMUSP00000002061 | 97963\_at | ST |
| ENSMUSP00000038540 | 96519\_at | E |
| ENSMUSP00000029608 | 101058\_at | E |
| ENSMUSP00000034188 | 99408\_at | T |
| ENSMUSP00000052103 | 160558\_at | E |
| ENSMUSP00000032373 | 101990\_at | E |
| ENSMUSP00000031458 | 100679\_at | ST |
| ENSMUSP00000021913 | 96650\_at | E |
| ENSMUSP00000048672 | 96783\_at | E |
| ENSMUSP00000050933 | 101334\_at | TR |
| ENSMUSP00000007436 | 99672\_at | T |
| ENSMUSP00000046169 | 160701\_at | ST |
| ENSMUSP00000035976 | 99817\_at | TR |
| ENSMUSP00000039823 | 94827\_at | T |
| ENSMUSP00000027687 | 100955\_at | E |
| ENSMUSP00000005631 | 92581\_at | E |
| ENSMUSP00000022746 | 102255\_at | ST |
| ENSMUSP00000034199 | 103844\_at | ST |
| ENSMUSP00000040846 | 95470\_at | E |
| ENSMUSP00000020990 | 94026\_at | ST |
| ENSMUSP00000056814 | 97048\_at | ST |
| ENSMUSP00000052768 | 102664\_at | E |
| ENSMUSP00000030333 | 102797\_at | E |
| ENSMUSP00000022176 | 99425\_at | E |
| ENSMUSP00000037385 | 100430\_at | ST |
| ENSMUSP00000025468 | 100696\_at | E |
| ENSMUSP00000060218 | 97324\_at | E |
| ENSMUSP00000036346 | 103452\_at | E |
| ENSMUSP00000001812 | 96812\_at | ST |
| ENSMUSP00000003066 | 95356\_at | T |
| ENSMUSP00000025262 | 102940\_at | ST |
| ENSMUSP00000004097 | 104240\_at | TR |
| ENSMUSP00000036437 | 99834\_at | ST |
| ENSMUSP00000031693 | 92743\_at | E |
| ENSMUSP00000046027 | 99033\_at | E |
| ENSMUSP00000051896 | 94176\_at | TR |
| ENSMUSP00000041814 | 160328\_at | E |
| ENSMUSP00000057950 | 101760\_at | E |
| ENSMUSP00000060996 | 98787\_at | T |
| ENSMUSP00000000709 | 93809\_at | E |
| ENSMUSP00000030952 | 102681\_at | ST |
| ENSMUSP00000007449 | 102826\_at | E |
| ENSMUSP00000057527 | 102959\_at | TR |
| ENSMUSP00000052196 | 160604\_at | TR |
| ENSMUSP00000023840 | 102025\_at | ST |
| ENSMUSP00000031773 | 93940\_at | E |
| ENSMUSP00000062156 | 103614\_at | TR |
| ENSMUSP00000029456 | 95373\_at | ST |
| ENSMUSP00000033006 | 100057\_at | T |
| ENSMUSP00000023390 | 101779\_at | ST |
| ENSMUSP00000027401 | 93272\_at | E |
| ENSMUSP00000003568 | 98395\_at | ST |
| ENSMUSP00000008542 | 99984\_at | TR |
| ENSMUSP00000035105 | 97750\_at | ST |
| ENSMUSP00000001872 | 102567\_at | TR |
| ENSMUSP00000001125 | 94060\_at | T |
| ENSMUSP00000059127 | 94193\_at | T |
| ENSMUSP00000009503 | 94205\_at | E |
| ENSMUSP00000001551 | 99328\_at | TR |
| ENSMUSP00000043892 | 100599\_at | TR |
| ENSMUSP00000061136 | 160178\_r\_at | TR |
| ENSMUSP00000000153 | 97227\_at | ST |
| ENSMUSP00000034834 | 96066\_s\_at | E |
| ENSMUSP00000052182 | 93681\_at | ST |
| ENSMUSP00000027939 | 93826\_at | E |
| ENSMUSP00000028292 | 96848\_at | E |
| ENSMUSP00000029684 | 98015\_at | T |
| ENSMUSP00000031632 | 104010\_at | TR |
| ENSMUSP00000018816 | 104143\_at | T |
| ENSMUSP00000047564 | 160754\_at | E |
| ENSMUSP00000025951 | 96047\_at | T |
| ENSMUSP00000009356 | 92978\_s\_at | E |
| ENSMUSP00000029670 | 97769\_at | ST |
| ENSMUSP00000021967 | 100074\_at | T |
| ENSMUSP00000000804 | 101542\_f\_at | E |
| ENSMUSP00000048119 | 98424\_at | ST |
| ENSMUSP00000000755 | 93434\_at | E |
| ENSMUSP00000023689 | 100483\_at | ST |
| ENSMUSP00000034497 | 98833\_at | E |
| ENSMUSP00000000349 | 98966\_at | E |
| ENSMUSP00000003017 | 92387\_at | E |
| ENSMUSP00000056705 | 96998\_at | E |
| ENSMUSP00000048924 | 102993\_at | E |
| ENSMUSP00000060087 | 92805\_s\_at | E |
| ENSMUSP00000037039 | 93042\_at | ST |
| ENSMUSP00000059655 | 104160\_at | E |
| ENSMUSP00000053161 | 94764\_at | ST |
| ENSMUSP00000033075 | 103781\_at | T |
| ENSMUSP00000030551 | 92796\_at | E |
| ENSMUSP00000045434 | 95552\_at | E |
| ENSMUSP00000035607 | 101825\_at | E |
| ENSMUSP00000028362 | 103258\_at | ST |
| ENSMUSP00000020051 | 161036\_at | T |
| ENSMUSP00000050372 | 102746\_at | ST |
| ENSMUSP00000059983 | 94372\_at | E |
| ENSMUSP00000059985 | 94517\_r\_at | ST |
| ENSMUSP00000018333 | 100512\_at | E |
| ENSMUSP00000030964 | 100778\_at | E |
| ENSMUSP00000039112 | 92271\_at | TR |
| ENSMUSP00000032715 | 97539\_at | E |
| ENSMUSP00000031524 | 103401\_at | E |
| ENSMUSP00000019050 | 98983\_at | E |
| ENSMUSP00000055095 | 102078\_at | ST |
| ENSMUSP00000021940 | 93993\_at | T |
| ENSMUSP00000057669 | 95438\_at | E |
| ENSMUSP00000021527 | 99916\_at | E |
| ENSMUSP00000047356 | 160933\_at | E |
| ENSMUSP00000026661 | 96081\_at | E |
| ENSMUSP00000038117 | 103810\_at | TR |
| ENSMUSP00000032344 | 94258\_at | ST |
| ENSMUSP00000034973 | 93613\_at | TR |
| ENSMUSP00000044838 | 103275\_at | T |
| ENSMUSP00000026756 | 96502\_at | E |
| ENSMUSP00000017836 | 96490\_at | E |
| ENSMUSP00000000708 | 102896\_at | ST |
| ENSMUSP00000034818 | 94534\_at | E |
| ENSMUSP00000020849 | 104063\_at | T |
| ENSMUSP00000029771 | 97689\_at | ST |
| ENSMUSP00000032703 | 92699\_at | T |
| ENSMUSP00000063119 | 103829\_at | E |
| ENSMUSP00000022852 | 95588\_at | E |
| ENSMUSP00000014743 | 101450\_at | ST |
| ENSMUSP00000060003 | 101728\_at | ST |
| ENSMUSP00000045571 | 93354\_at | T |
| ENSMUSP00000003067 | 99933\_at | ST |
| ENSMUSP00000020664 | 103028\_at | E |
| ENSMUSP00000049394 | 96110\_at | E |
| ENSMUSP00000023580 | 93087\_r\_at | E |
| ENSMUSP00000002964 | 97832\_at | ST |
| ENSMUSP00000044234 | 97965\_at | E |
| ENSMUSP00000037762 | 94142\_at | ST |
| ENSMUSP00000030446 | 94275\_at | E |
| ENSMUSP00000018430 | 101992\_at | E |
| ENSMUSP00000013842 | 100548\_at | T |
| ENSMUSP00000021209 | 101191\_at | T |
| ENSMUSP00000019701 | 102925\_at | E |
| ENSMUSP00000034370 | 92450\_at | T |
| ENSMUSP00000001036 | 97573\_at | E |
| ENSMUSP00000006887 | 102257\_at | TR |
| ENSMUSP00000048246 | 99950\_at | TR |
| ENSMUSP00000020078 | 104634\_at | T |
| ENSMUSP00000001460 | 96260\_at | E |
| ENSMUSP00000006749 | 101077\_at | T |
| ENSMUSP00000004770 | 102666\_at | E |
| ENSMUSP00000032349 | 94292\_at | ST |
| ENSMUSP00000001878 | 99427\_at | TR |
| ENSMUSP00000057231 | 100698\_at | TR |
| ENSMUSP00000050907 | 92191\_at | E |
| ENSMUSP00000034848 | 97459\_at | E |
| ENSMUSP00000050388 | 98915\_at | E |
| ENSMUSP00000004473 | 103454\_at | TR |
| ENSMUSP00000002883 | 92469\_at | ST |
| ENSMUSP00000014271 | 99580\_s\_at | E |
| ENSMUSP00000004729 | 96947\_at | T |
| ENSMUSP00000034369 | 101486\_at | E |
| ENSMUSP00000025279 | 98114\_at | ST |
| ENSMUSP00000035422 | 93257\_at | E |
| ENSMUSP00000006907 | 99836\_at | E |
| ENSMUSP00000026568 | 160853\_at | E |
| ENSMUSP00000008031 | 100974\_at | TR |
| ENSMUSP00000032539 | 92612\_at | E |
| ENSMUSP00000036320 | 97735\_at | ST |
| ENSMUSP00000004171 | 97868\_at | ST |
| ENSMUSP00000025192 | 102274\_at | ST |
| ENSMUSP00000005755 | 99035\_at | E |
| ENSMUSP00000030041 | 94045\_at | T |
| ENSMUSP00000033139 | 103475\_s\_at | E |
| ENSMUSP00000006856 | 103207\_at | E |
| ENSMUSP00000034995 | 104651\_at | ST |
| ENSMUSP00000031037 | 102683\_at | T |
| ENSMUSP00000001271 | 99444\_at | ST |
| ENSMUSP00000020129 | 99577\_at | ST |
| ENSMUSP00000000356 | 94454\_at | T |
| ENSMUSP00000061997 | 104128\_at | T |
| ENSMUSP00000047231 | 160739\_at | E |
| ENSMUSP00000031029 | 100582\_at | ST |
| ENSMUSP00000031740 | 97343\_at | E |
| ENSMUSP00000039762 | 92353\_at | E |
| ENSMUSP00000027271 | 93942\_at | E |
| ENSMUSP00000018754 | 92486\_at | T |
| ENSMUSP00000032934 | 160090\_f\_at | E |
| ENSMUSP00000004054 | 101370\_at | T |
| ENSMUSP00000060686 | 100059\_at | E |
| ENSMUSP00000021129 | 101515\_at | E |
| ENSMUSP00000029850 | 98131\_at | E |
| ENSMUSP00000058575 | 101648\_at | TR |
| ENSMUSP00000027195 | 93274\_at | E |
| ENSMUSP00000032403 | 93419\_at | E |
| ENSMUSP00000020984 | 160870\_at | E |
| ENSMUSP00000041450 | 104404\_at | E |
| ENSMUSP00000032242 | 92762\_at | ST |
| ENSMUSP00000033477 | 102291\_at | E |
| ENSMUSP00000025081 | 99052\_at | TR |
| ENSMUSP00000021034 | 101924\_at | E |
| ENSMUSP00000033430 | 103224\_at | E |
| ENSMUSP00000035255 | 93683\_at | E |
| ENSMUSP00000029240 | 103357\_at | T |
| ENSMUSP00000032326 | 96717\_at | E |
| ENSMUSP00000031377 | 101389\_at | ST |
| ENSMUSP00000006956 | 102712\_at | T |
| ENSMUSP00000063080 | 99606\_at | E |
| ENSMUSP00000031350 | 160623\_at | E |
| ENSMUSP00000004565 | 160756\_at | E |
| ENSMUSP00000004474 | 97505\_at | E |
| ENSMUSP00000044879 | 92515\_at | TR |
| ENSMUSP00000034368 | 160744\_r\_at | E |
| ENSMUSP00000029481 | 92648\_at | T |
| ENSMUSP00000021789 | 95392\_at | E |
| ENSMUSP00000029729 | 160088\_at | E |
| ENSMUSP00000004920 | 95537\_at | E |
| ENSMUSP00000015618 | 98426\_at | E |
| ENSMUSP00000028010 | 104421\_at | E |
| ENSMUSP00000050412 | 104687\_at | ST |
| ENSMUSP00000023952 | 101009\_at | E |
| ENSMUSP00000015583 | 92924\_at | E |
| ENSMUSP00000011178 | 94357\_at | T |
| ENSMUSP00000023889 | 160509\_at | E |
| ENSMUSP00000000834 | 97113\_at | ST |
| ENSMUSP00000021907 | 97379\_at | E |
| ENSMUSP00000022013 | 103241\_at | E |
| ENSMUSP00000056310 | 93978\_at | E |
| ENSMUSP00000037555 | 103519\_at | E |
| ENSMUSP00000022235 | 101140\_at | ST |
| ENSMUSP00000036240 | 160640\_at | E |
| ENSMUSP00000051085 | 99889\_at | ST |
| ENSMUSP00000001327 | 100906\_at | ST |
| ENSMUSP00000007737 | 93418\_g\_at | TR |
| ENSMUSP00000057182 | 96199\_at | E |
| ENSMUSP00000020323 | 92532\_at | ST |
| ENSMUSP00000053496 | 160117\_at | TR |
| ENSMUSP00000058662 | 100093\_at | E |
| ENSMUSP00000025835 | 93320\_at | E |
| ENSMUSP00000003772 | 93453\_at | ST |
| ENSMUSP00000025027 | 95114\_s\_at | T |
| ENSMUSP00000026839 | 161038\_at | E |
| ENSMUSP00000010899 | 104048\_at | E |
| ENSMUSP00000000304 | 160659\_at | E |
| ENSMUSP00000020930 | 100514\_at | ST |
| ENSMUSP00000027302 | 92273\_at | E |
| ENSMUSP00000041675 | 103391\_at | E |
| ENSMUSP00000033241 | 103403\_at | E |
| ENSMUSP00000015956 | 101290\_at | ST |
| ENSMUSP00000009875 | 101302\_at | T |
| ENSMUSP00000053508 | 101435\_at | ST |
| ENSMUSP00000025827 | 99640\_at | E |
| ENSMUSP00000027669 | 98329\_at | E |
| ENSMUSP00000034874 | 96228\_at | E |
| ENSMUSP00000033423 | 92682\_at | T |
| ENSMUSP00000021944 | 92827\_at | TR |
| ENSMUSP00000029932 | 103812\_at | T |
| ENSMUSP00000005952 | 95571\_at | T |
| ENSMUSP00000017597 | 101844\_at | E |
| ENSMUSP00000059061 | 101977\_at | E |
| ENSMUSP00000025396 | 97149\_at | TR |
| ENSMUSP00000032419 | 98593\_at | E |
| ENSMUSP00000045044 | 103011\_at | TR |
| ENSMUSP00000028283 | 101176\_at | T |
| ENSMUSP00000008605 | 99381\_at | E |
| ENSMUSP00000028900 | 160410\_at | T |
| ENSMUSP00000032195 | 104065\_at | E |
| ENSMUSP00000019939 | 160543\_at | T |
| ENSMUSP00000005062 | 104428\_s\_at | E |
| ENSMUSP00000044866 | 92302\_at | ST |
| ENSMUSP00000027769 | 92568\_at | E |
| ENSMUSP00000011152 | 103686\_at | T |
| ENSMUSP00000006539 | 101585\_at | ST |
| ENSMUSP00000000505 | 93356\_at | E |
| ENSMUSP00000058728 | 99802\_at | ST |
| ENSMUSP00000034866 | 96112\_at | T |
| ENSMUSP00000004507 | 96245\_at | E |
| ENSMUSP00000031059 | 102240\_at | TR |
| ENSMUSP00000024995 | 92711\_at | E |
| ENSMUSP00000026922 | 160695\_i\_at | ST |
| ENSMUSP00000035533 | 94277\_at | T |
| ENSMUSP00000024737 | 95733\_at | T |
| ENSMUSP00000038329 | 160429\_at | T |
| ENSMUSP00000034378 | 100417\_at | T |
| ENSMUSP00000029147 | 95065\_at | E |
| ENSMUSP00000029894 | 96521\_at | E |
| ENSMUSP00000059116 | 96787\_at | E |
| ENSMUSP00000021866 | 102782\_at | E |
| ENSMUSP00000033981 | 99410\_at | T |
| ENSMUSP00000020316 | 98087\_at | E |
| ENSMUSP00000020161 | 93097\_at | E |
| ENSMUSP00000024227 | 104082\_at | E |
| ENSMUSP00000013970 | 160693\_at | E |
| ENSMUSP00000051079 | 104227\_at | ST |
| ENSMUSP00000049529 | 97442\_at | T |
| ENSMUSP00000005066 | 92585\_at | E |
| ENSMUSP00000032080 | 95619\_at | E |
| ENSMUSP00000003964 | 98496\_at | E |
| ENSMUSP00000001802 | 93373\_at | E |
| ENSMUSP00000027713 | 99952\_at | E |
| ENSMUSP00000031386 | 102402\_at | E |
| ENSMUSP00000010241 | 101079\_at | T |
| ENSMUSP00000026891 | 99151\_at | E |
| ENSMUSP00000001706 | 92891\_f\_at | TR |
| ENSMUSP00000038514 | 92205\_at | ST |
| ENSMUSP00000032557 | 97328\_at | E |
| ENSMUSP00000031318 | 98772\_at | ST |
| ENSMUSP00000045067 | 98917\_at | T |
| ENSMUSP00000030482 | 103353\_f\_at | E |
| ENSMUSP00000036946 | 102811\_at | E |
| ENSMUSP00000001304 | 93126\_at | E |
| ENSMUSP00000034865 | 94715\_at | E |
| ENSMUSP00000021757 | 99838\_at | E |
| ENSMUSP00000047220 | 96148\_at | E |
| ENSMUSP00000028922 | 92747\_at | TR |
| ENSMUSP00000046625 | 103732\_at | E |
| ENSMUSP00000024974 | 100042\_at | E |
| ENSMUSP00000042164 | 101764\_at | ST |
| ENSMUSP00000051105 | 103064\_at | E |
| ENSMUSP00000041857 | 104653\_at | E |
| ENSMUSP00000061397 | 99347\_f\_at | E |
| ENSMUSP00000030443 | 96557\_at | ST |
| ENSMUSP00000059587 | 102552\_at | T |
| ENSMUSP00000061999 | 94323\_at | T |
| ENSMUSP00000023215 | 100451\_at | TR |
| ENSMUSP00000030627 | 103340\_at | T |
| ENSMUSP00000022917 | 92355\_at | TR |
| ENSMUSP00000027009 | 92488\_at | E |
| ENSMUSP00000001792 | 102029\_at | ST |
| ENSMUSP00000055288 | 98573\_r\_at | ST |
| ENSMUSP00000017344 | 99855\_at | E |
| ENSMUSP00000031199 | 104539\_at | E |
| ENSMUSP00000046974 | 102293\_at | TR |
| ENSMUSP00000027682 | 102305\_at | ST |
| ENSMUSP00000003074 | 97887\_at | T |
| ENSMUSP00000026210 | 103882\_at | E |
| ENSMUSP00000004913 | 92909\_at | ST |
| ENSMUSP00000025714 | 160071\_at | E |
| ENSMUSP00000025546 | 160349\_at | E |
| ENSMUSP00000033495 | 101926\_at | E |
| ENSMUSP00000028045 | 103226\_at | ST |
| ENSMUSP00000022019 | 96574\_at | ST |
| ENSMUSP00000060098 | 98809\_s\_at | ST |
| ENSMUSP00000045999 | 99330\_at | ST |
| ENSMUSP00000031741 | 99463\_at | E |
| ENSMUSP00000056502 | 93029\_at | E |
| ENSMUSP00000006787 | 104014\_at | ST |
| ENSMUSP00000030018 | 104147\_at | E |
| ENSMUSP00000059910 | 94618\_at | TR |
| ENSMUSP00000035579 | 97507\_at | ST |
| ENSMUSP00000033005 | 103490\_at | ST |
| ENSMUSP00000022727 | 103635\_at | E |
| ENSMUSP00000034585 | 100078\_at | T |
| ENSMUSP00000021674 | 160901\_at | TR |
| ENSMUSP00000062344 | 104689\_at | T |
| ENSMUSP00000031103 | 102322\_at | E |
| ENSMUSP00000027061 | 99349\_at | ST |
| ENSMUSP00000040477 | 101810\_at | ST |
| ENSMUSP00000033640 | 161021\_at | E |
| ENSMUSP00000034264 | 95147\_at | E |
| ENSMUSP00000022906 | 101142\_at | ST |
| ENSMUSP00000018711 | 96869\_at | ST |
| ENSMUSP00000048648 | 102864\_at | TR |
| ENSMUSP00000021921 | 104031\_at | ST |
| ENSMUSP00000032122 | 94635\_at | ST |
| ENSMUSP00000020284 | 104164\_at | E |
| ENSMUSP00000054602 | 104297\_at | T |
| ENSMUSP00000021065 | 100896\_at | T |
| ENSMUSP00000056284 | 101959\_r\_at | TR |
| ENSMUSP00000053649 | 96068\_at | E |
| ENSMUSP00000020645 | 92401\_at | E |
| ENSMUSP00000029868 | 92534\_at | E |
| ENSMUSP00000061942 | 102063\_at | E |
| ENSMUSP00000043190 | 102196\_at | ST |
| ENSMUSP00000023429 | 102208\_at | E |
| ENSMUSP00000036442 | 98398\_s\_at | E |
| ENSMUSP00000030687 | 98312\_at | T |
| ENSMUSP00000035992 | 98445\_at | T |
| ENSMUSP00000058321 | 99901\_at | TR |
| ENSMUSP00000058825 | 104573\_at | TR |
| ENSMUSP00000018433 | 96344\_at | E |
| ENSMUSP00000030800 | 97800\_at | E |
| ENSMUSP00000051843 | 97933\_at | E |
| ENSMUSP00000036604 | 92810\_at | E |
| ENSMUSP00000025828 | 100516\_at | E |
| ENSMUSP00000051962 | 97398\_at | E |
| ENSMUSP00000031795 | 93731\_at | E |
| ENSMUSP00000007444 | 103405\_at | E |
| ENSMUSP00000018748 | 103538\_at | TR |
| ENSMUSP00000000964 | 95297\_at | TR |
| ENSMUSP00000057937 | 96753\_at | E |
| ENSMUSP00000020190 | 104181\_at | E |
| ENSMUSP00000031929 | 93208\_at | E |
| ENSMUSP00000034903 | 96085\_at | E |
| ENSMUSP00000005787 | 92551\_at | E |
| ENSMUSP00000026050 | 97819\_at | E |
| ENSMUSP00000057802 | 95573\_at | TR |
| ENSMUSP00000029297 | 101713\_at | T |
| ENSMUSP00000057569 | 103013\_at | TR |
| ENSMUSP00000058250 | 93617\_at | ST |
| ENSMUSP00000026289 | 101045\_at | E |
| ENSMUSP00000036853 | 92960\_at | ST |
| ENSMUSP00000040488 | 160545\_at | E |
| ENSMUSP00000027054 | 97695\_s\_at | TR |
| ENSMUSP00000047402 | 97235\_f\_at | E |
| ENSMUSP00000057257 | 96770\_at | E |
| ENSMUSP00000047551 | 101587\_at | E |
| ENSMUSP00000059705 | 99804\_at | ST |
| ENSMUSP00000043587 | 99937\_at | TR |
| ENSMUSP00000000001 | 94814\_at | ST |
| ENSMUSP00000030802 | 102242\_at | ST |
| ENSMUSP00000030938 | 99961\_s\_at | E |
| ENSMUSP00000017868 | 92979\_at | TR |
| ENSMUSP00000019074 | 94146\_at | ST |
| ENSMUSP00000051952 | 104474\_s\_at | ST |
| ENSMUSP00000015320 | 95735\_at | T |
| ENSMUSP00000060079 | 101996\_at | E |
| ENSMUSP00000029331 | 97168\_at | ST |
| ENSMUSP00000028907 | 100390\_s\_at | E |
| ENSMUSP00000037274 | 96523\_at | TR |
| ENSMUSP00000057181 | 101062\_at | E |
| ENSMUSP00000044394 | 102651\_at | E |
| ENSMUSP00000039107 | 99412\_at | ST |
| ENSMUSP00000036472 | 94555\_at | E |
| ENSMUSP00000005923 | 98557\_f\_at | E |
| ENSMUSP00000034435 | 160421\_r\_at | E |
| ENSMUSP00000002087 | 93910\_at | E |
| ENSMUSP00000034552 | 92587\_at | T |
| ENSMUSP00000026378 | 95343\_at | E |
| ENSMUSP00000033300 | 104638\_at | E |
| ENSMUSP00000025363 | 92730\_at | ST |
| ENSMUSP00000028328 | 97986\_at | E |
| ENSMUSP00000021692 | 99020\_at | TR |
| ENSMUSP00000030044 | 100436\_at | T |
| ENSMUSP00000041005 | 92195\_at | TR |
| ENSMUSP00000026198 | 161103\_at | E |
| ENSMUSP00000003035 | 101357\_at | T |
| ENSMUSP00000034121 | 99562\_at | E |
| ENSMUSP00000024778 | 160724\_at | E |
| ENSMUSP00000002053 | 100712\_at | ST |
| ENSMUSP00000016016 | 97739\_at | E |
| ENSMUSP00000061576 | 92616\_at | E |
| ENSMUSP00000055730 | 94049\_at | E |
| ENSMUSP00000047996 | 95493\_at | E |
| ENSMUSP00000028681 | 101899\_at | E |
| ENSMUSP00000032958 | 93392\_at | T |
| ENSMUSP00000024946 | 98527\_at | E |
| ENSMUSP00000014477 | 94776\_f\_at | ST |
| ENSMUSP00000020969 | 103066\_at | E |
| ENSMUSP00000027860 | 103199\_at | ST |
| ENSMUSP00000062006 | 93162\_f\_at | ST |
| ENSMUSP00000041524 | 102554\_at | ST |
| ENSMUSP00000029626 | 94458\_at | E |
| ENSMUSP00000029353 | 100320\_at | T |
| ENSMUSP00000019133 | 100453\_at | E |
| ENSMUSP00000027105 | 92357\_at | E |
| ENSMUSP00000059196 | 98936\_at | E |
| ENSMUSP00000021475 | 92544\_f\_at | E |
| ENSMUSP00000055551 | 96968\_at | ST |
| ENSMUSP00000040245 | 98002\_at | TR |
| ENSMUSP00000015769 | 102963\_at | TR |
| ENSMUSP00000006687 | 94734\_at | T |
| ENSMUSP00000016400 | 92633\_at | E |
| ENSMUSP00000023820 | 97889\_at | T |
| ENSMUSP00000055673 | 102295\_at | T |
| ENSMUSP00000028123 | 92899\_at | E |
| ENSMUSP00000030763 | 93421\_at | E |
| ENSMUSP00000020726 | 98544\_at | E |
| ENSMUSP00000050935 | 103083\_at | E |
| ENSMUSP00000028389 | 104672\_at | ST |
| ENSMUSP00000043367 | 103228\_at | E |
| ENSMUSP00000021665 | 101127\_at | TR |
| ENSMUSP00000030719 | 102716\_at | E |
| ENSMUSP00000050077 | 99332\_at | ST |
| ENSMUSP00000032374 | 102849\_at | T |
| ENSMUSP00000042351 | 160482\_at | E |
| ENSMUSP00000045325 | 104016\_at | T |
| ENSMUSP00000028288 | 97497\_at | ST |
| ENSMUSP00000025718 | 102048\_at | TR |
| ENSMUSP00000028897 | 103492\_at | E |
| ENSMUSP00000037115 | 103504\_at | TR |
| ENSMUSP00000023088 | 103637\_at | E |
| ENSMUSP00000020932 | 96852\_at | E |
| ENSMUSP00000060928 | 96985\_at | ST |
| ENSMUSP00000024004 | 101403\_at | ST |
| ENSMUSP00000000033 | 98623\_g\_at | ST |
| ENSMUSP00000021054 | 92783\_at | ST |
| ENSMUSP00000026925 | 103913\_at | T |
| ENSMUSP00000032194 | 104701\_at | TR |
| ENSMUSP00000021779 | 103378\_at | ST |
| ENSMUSP00000031748 | 93849\_at | E |
| ENSMUSP00000023974 | 161023\_at | ST |
| ENSMUSP00000043512 | 95016\_at | ST |
| ENSMUSP00000052255 | 95149\_at | T |
| ENSMUSP00000027239 | 101144\_at | ST |
| ENSMUSP00000033960 | 96738\_at | E |
| ENSMUSP00000015585 | 102733\_at | E |
| ENSMUSP00000002735 | 93048\_at | E |
| ENSMUSP00000032226 | 160511\_at | ST |
| ENSMUSP00000055313 | 104166\_at | E |
| ENSMUSP00000035306 | 104299\_at | TR |
| ENSMUSP00000022371 | 97526\_at | T |
| ENSMUSP00000036227 | 92403\_at | E |
| ENSMUSP00000021733 | 98970\_at | E |
| ENSMUSP00000002283 | 102198\_at | T |
| ENSMUSP00000055225 | 103787\_at | T |
| ENSMUSP00000027153 | 95425\_at | E |
| ENSMUSP00000036299 | 101420\_at | T |
| ENSMUSP00000022642 | 101723\_r\_at | E |
| ENSMUSP00000009883 | 99891\_at | TR |
| ENSMUSP00000033621 | 102341\_at | E |
| ENSMUSP00000040271 | 94112\_at | ST |
| ENSMUSP00000053638 | 99368\_at | ST |
| ENSMUSP00000027748 | 94378\_at | ST |
| ENSMUSP00000030254 | 93600\_at | ST |
| ENSMUSP00000005329 | 103055\_r\_at | E |
| ENSMUSP00000029438 | 103262\_at | E |
| ENSMUSP00000040369 | 98856\_at | ST |
| ENSMUSP00000033413 | 98989\_at | E |
| ENSMUSP00000058629 | 161040\_at | ST |
| ENSMUSP00000062555 | 101161\_at | ST |
| ENSMUSP00000044610 | 95299\_at | E |
| ENSMUSP00000030455 | 96888\_at | E |
| ENSMUSP00000050995 | 102750\_at | T |
| ENSMUSP00000023405 | 101439\_at | E |
| ENSMUSP00000060752 | 94521\_at | E |
| ENSMUSP00000034727 | 104328\_at | T |
| ENSMUSP00000056369 | 96087\_at | E |
| ENSMUSP00000027495 | 97543\_at | E |
| ENSMUSP00000052302 | 92420\_at | ST |
| ENSMUSP00000022573 | 92553\_at | E |
| ENSMUSP00000029541 | 92686\_at | E |
| ENSMUSP00000006713 | 103949\_at | E |
| ENSMUSP00000036543 | 100126\_at | E |
| ENSMUSP00000056293 | 101715\_at | E |
| ENSMUSP00000028473 | 98597\_at | E |
| ENSMUSP00000019038 | 98609\_at | E |
| ENSMUSP00000021271 | 93619\_at | ST |
| ENSMUSP00000047725 | 104604\_at | TR |
| ENSMUSP00000032454 | 161059\_at | T |
| ENSMUSP00000017798 | 92962\_at | ST |
| ENSMUSP00000003779 | 94407\_at | E |
| ENSMUSP00000042827 | 97151\_at | ST |
| ENSMUSP00000021241 | 98873\_at | TR |
| ENSMUSP00000056389 | 97429\_at | E |
| ENSMUSP00000015145 | 92439\_at | E |
| ENSMUSP00000004588 | 100061\_f\_at | E |
| ENSMUSP00000051577 | 95328\_at | E |
| ENSMUSP00000026461 | 96772\_at | E |
| ENSMUSP00000022817 | 99806\_at | ST |
| ENSMUSP00000028161 | 99939\_at | E |
| ENSMUSP00000051642 | 104345\_at | T |
| ENSMUSP00000027299 | 102244\_at | E |
| ENSMUSP00000022856 | 99005\_at | E |
| ENSMUSP00000060513 | 103833\_at | E |
| ENSMUSP00000049560 | 92848\_at | E |
| ENSMUSP00000017460 | 94148\_at | ST |
| ENSMUSP00000030896 | 95737\_at | T |
| ENSMUSP00000049729 | 98481\_at | E |
| ENSMUSP00000035614 | 103032\_at | E |
| ENSMUSP00000029625 | 93503\_at | ST |
| ENSMUSP00000034594 | 96525\_at | ST |
| ENSMUSP00000056882 | 101209\_at | ST |
| ENSMUSP00000023244 | 99414\_at | ST |
| ENSMUSP00000053962 | 160564\_at | T |
| ENSMUSP00000039663 | 104086\_at | E |
| ENSMUSP00000020188 | 100552\_at | ST |
| ENSMUSP00000035056 | 97446\_at | E |
| ENSMUSP00000052866 | 98890\_at | E |
| ENSMUSP00000031399 | 92589\_at | E |
| ENSMUSP00000040153 | 95345\_at | TR |
| ENSMUSP00000028159 | 96801\_at | E |
| ENSMUSP00000034808 | 101473\_at | E |
| ENSMUSP00000051355 | 98367\_at | TR |
| ENSMUSP00000029975 | 94700\_at | ST |
| ENSMUSP00000004327 | 94966\_at | E |
| ENSMUSP00000022618 | 92732\_at | E |
| ENSMUSP00000006136 | 92865\_at | E |
| ENSMUSP00000062782 | 92998\_at | ST |
| ENSMUSP00000023231 | 94298\_at | E |
| ENSMUSP00000034293 | 95754\_at | E |
| ENSMUSP00000047630 | 100438\_at | ST |
| ENSMUSP00000040332 | 103327\_at | TR |
| ENSMUSP00000036640 | 101081\_at | E |
| ENSMUSP00000033121 | 104140\_s\_at | E |
| ENSMUSP00000001258 | 99564\_at | T |
| ENSMUSP00000061126 | 92600\_f\_at | E |
| ENSMUSP00000036872 | 94719\_at | T |
| ENSMUSP00000059495 | 100009\_r\_at | TR |
| ENSMUSP00000057822 | 92340\_at | ST |
| ENSMUSP00000037497 | 102147\_at | E |
| ENSMUSP00000033809 | 95507\_at | E |
| ENSMUSP00000005810 | 100046\_at | E |
| ENSMUSP00000060521 | 101502\_at | TR |
| ENSMUSP00000016511 | 98384\_at | E |
| ENSMUSP00000021607 | 93261\_at | E |
| ENSMUSP00000018403 | 98529\_at | ST |
| ENSMUSP00000021621 | 103068\_at | E |
| ENSMUSP00000027218 | 102423\_at | E |
| ENSMUSP00000036203 | 102556\_at | ST |
| ENSMUSP00000019290 | 94182\_at | T |
| ENSMUSP00000007831 | 100588\_at | E |
| ENSMUSP00000048079 | 98793\_at | ST |
| ENSMUSP00000025521 | 103477\_at | TR |
| ENSMUSP00000027219 | 93948\_at | E |
| ENSMUSP00000057424 | 102832\_at | E |
| ENSMUSP00000020504 | 99581\_at | E |
| ENSMUSP00000026356 | 98137\_at | E |
| ENSMUSP00000060390 | 94736\_at | T |
| ENSMUSP00000020979 | 160876\_at | T |
| ENSMUSP00000020959 | 102031\_at | E |
| ENSMUSP00000061742 | 102164\_at | T |
| ENSMUSP00000018491 | 102297\_at | TR |
| ENSMUSP00000014141 | 160075\_at | E |
| ENSMUSP00000006679 | 104541\_at | E |
| ENSMUSP00000060926 | 102573\_at | ST |
| ENSMUSP00000046127 | 160351\_at | E |
| ENSMUSP00000033133 | 160629\_at | ST |
| ENSMUSP00000058874 | 97100\_at | T |
| ENSMUSP00000029692 | 92376\_at | E |
| ENSMUSP00000059420 | 96987\_at | TR |
| ENSMUSP00000002292 | 98287\_at | E |
| ENSMUSP00000049175 | 94753\_at | ST |
| ENSMUSP00000022782 | 96186\_at | ST |
| ENSMUSP00000015612 | 92652\_at | ST |
| ENSMUSP00000036245 | 160104\_at | E |
| ENSMUSP00000026408 | 101814\_at | ST |
| ENSMUSP00000058419 | 101947\_at | E |
| ENSMUSP00000055957 | 101146\_at | TR |
| ENSMUSP00000042691 | 94361\_at | E |
| ENSMUSP00000003032 | 94494\_at | E |
| ENSMUSP00000039252 | 100634\_at | E |
| ENSMUSP00000028841 | 98972\_at | E |
| ENSMUSP00000003828 | 92405\_at | T |
| ENSMUSP00000037528 | 94745\_f\_at | T |
| ENSMUSP00000042187 | 100099\_at | E |
| ENSMUSP00000001903 | 101555\_at | E |
| ENSMUSP00000033473 | 99893\_at | ST |
| ENSMUSP00000033245 | 99905\_at | E |
| ENSMUSP00000060571 | 160922\_at | E |
| ENSMUSP00000040777 | 96348\_at | E |
| ENSMUSP00000023593 | 99104\_at | ST |
| ENSMUSP00000028636 | 160121\_at | E |
| ENSMUSP00000002708 | 101831\_at | E |
| ENSMUSP00000060784 | 97003\_at | ST |
| ENSMUSP00000022529 | 101964\_at | E |
| ENSMUSP00000056307 | 98580\_at | E |
| ENSMUSP00000016157 | 98508\_s\_at | E |
| ENSMUSP00000025903 | 93602\_at | E |
| ENSMUSP00000014495 | 98858\_at | ST |
| ENSMUSP00000015358 | 103264\_at | E |
| ENSMUSP00000036555 | 95035\_at | T |
| ENSMUSP00000038204 | 101163\_at | E |
| ENSMUSP00000057981 | 101308\_at | T |
| ENSMUSP00000030412 | 99646\_at | E |
| ENSMUSP00000021956 | 160663\_at | E |
| ENSMUSP00000050389 | 104185\_at | E |
| ENSMUSP00000057038 | 102229\_at | E |
| ENSMUSP00000002172 | 92688\_at | E |
| ENSMUSP00000025230 | 103673\_at | E |
| ENSMUSP00000000984 | 103818\_at | T |
| ENSMUSP00000020099 | 100128\_at | E |
| ENSMUSP00000044573 | 101717\_at | ST |
| ENSMUSP00000030669 | 99922\_at | T |
| ENSMUSP00000023352 | 93476\_at | E |
| ENSMUSP00000038870 | 94932\_at | ST |
| ENSMUSP00000023065 | 96498\_at | E |
| ENSMUSP00000021930 | 92831\_at | T |
| ENSMUSP00000052894 | 99387\_at | ST |
| ENSMUSP00000021332 | 160416\_at | E |
| ENSMUSP00000051863 | 94397\_at | E |
| ENSMUSP00000046012 | 100392\_at | ST |
| ENSMUSP00000053977 | 97153\_at | ST |
| ENSMUSP00000027554 | 92296\_at | E |
| ENSMUSP00000005606 | 103559\_at | E |
| ENSMUSP00000045039 | 101180\_at | E |
| ENSMUSP00000033326 | 101458\_at | E |
| ENSMUSP00000054057 | 99530\_at | ST |
| ENSMUSP00000006094 | 94540\_at | E |
| ENSMUSP00000056315 | 99808\_at | TR |
| ENSMUSP00000022787 | 104214\_at | T |
| ENSMUSP00000045409 | 94818\_at | E |
| ENSMUSP00000040364 | 92717\_at | TR |
| ENSMUSP00000025935 | 101867\_at | E |
| ENSMUSP00000052586 | 93360\_at | E |
| ENSMUSP00000003442 | 98483\_at | T |
| ENSMUSP00000021793 | 94393\_r\_at | E |
| ENSMUSP00000033086 | 97971\_at | E |
| ENSMUSP00000036316 | 101199\_at | ST |
| ENSMUSP00000024831 | 160300\_at | ST |
| ENSMUSP00000045720 | 100554\_at | TR |
| ENSMUSP00000004140 | 93543\_f\_at | E |
| ENSMUSP00000049654 | 97448\_at | E |
| ENSMUSP00000015549 | 160986\_r\_at | E |
| ENSMUSP00000027241 | 93914\_at | ST |
| ENSMUSP00000036271 | 95347\_at | TR |
| ENSMUSP00000059689 | 96803\_at | E |
| ENSMUSP00000054941 | 101342\_at | ST |
| ENSMUSP00000031410 | 104364\_at | E |
| ENSMUSP00000026551 | 93379\_at | E |
| ENSMUSP00000015576 | 99958\_at | E |
| ENSMUSP00000049683 | 104509\_at | E |
| ENSMUSP00000045465 | 92601\_at | E |
| ENSMUSP00000026892 | 97857\_at | TR |
| ENSMUSP00000034524 | 94034\_at | E |
| ENSMUSP00000016086 | 161080\_f\_at | T |
| ENSMUSP00000024223 | 99566\_at | E |
| ENSMUSP00000033050 | 160583\_at | ST |
| ENSMUSP00000003044 | 97332\_at | E |
| ENSMUSP00000050985 | 97465\_at | T |
| ENSMUSP00000041357 | 103593\_at | ST |
| ENSMUSP00000055816 | 95364\_at | ST |
| ENSMUSP00000034689 | 101492\_at | E |
| ENSMUSP00000060884 | 94852\_at | E |
| ENSMUSP00000029450 | 97741\_at | ST |
| ENSMUSP00000039864 | 99041\_at | T |
| ENSMUSP00000048059 | 94184\_at | ST |
| ENSMUSP00000025675 | 160191\_at | E |
| ENSMUSP00000043390 | 160336\_at | E |
| ENSMUSP00000024884 | 93672\_at | E |
| ENSMUSP00000053818 | 98807\_at | ST |
| ENSMUSP00000029685 | 103346\_at | E |
| ENSMUSP00000005669 | 102701\_at | E |
| ENSMUSP00000009138 | 94460\_at | E |
| ENSMUSP00000006973 | 160745\_at | E |
| ENSMUSP00000028815 | 104267\_at | T |
| ENSMUSP00000015815 | 100733\_at | E |
| ENSMUSP00000022428 | 96038\_at | E |
| ENSMUSP00000050087 | 102033\_at | E |
| ENSMUSP00000020522 | 92637\_at | E |
| ENSMUSP00000051278 | 95381\_at | T |
| ENSMUSP00000022696 | 95526\_at | T |
| ENSMUSP00000029214 | 95659\_at | E |
| ENSMUSP00000040610 | 98415\_at | ST |
| ENSMUSP00000004392 | 93425\_at | TR |
| ENSMUSP00000032954 | 103087\_at | E |
| ENSMUSP00000033496 | 93558\_at | T |
| ENSMUSP00000059421 | 97903\_at | E |
| ENSMUSP00000050862 | 95465\_s\_at | T |
| ENSMUSP00000006686 | 102575\_at | T |
| ENSMUSP00000040163 | 95935\_at | T |
| ENSMUSP00000058590 | 97090\_at | TR |
| ENSMUSP00000026896 | 100474\_at | E |
| ENSMUSP00000001976 | 101930\_at | TR |
| ENSMUSP00000042193 | 92378\_at | E |
| ENSMUSP00000002699 | 95001\_at | E |
| ENSMUSP00000028398 | 93033\_at | E |
| ENSMUSP00000033429 | 98289\_at | TR |
| ENSMUSP00000028882 | 94755\_at | ST |
| ENSMUSP00000033512 | 160907\_at | T |
| ENSMUSP00000035120 | 96055\_at | ST |
| ENSMUSP00000042373 | 97511\_at | E |
| ENSMUSP00000044612 | 92521\_at | TR |
| ENSMUSP00000015723 | 97777\_at | TR |
| ENSMUSP00000027189 | 102328\_at | E |
| ENSMUSP00000002765 | 94087\_at | E |
| ENSMUSP00000022256 | 160239\_at | E |
| ENSMUSP00000023396 | 101949\_at | E |
| ENSMUSP00000029904 | 93575\_at | E |
| ENSMUSP00000026927 | 104693\_at | E |
| ENSMUSP00000060350 | 92930\_at | TR |
| ENSMUSP00000041030 | 101148\_at | E |
| ENSMUSP00000037629 | 100491\_at | T |
| ENSMUSP00000042026 | 97385\_at | E |
| ENSMUSP00000061498 | 93851\_at | E |
| ENSMUSP00000021776 | 98023\_r\_at | ST |
| ENSMUSP00000019633 | 98318\_at | ST |
| ENSMUSP00000038473 | 99895\_at | ST |
| ENSMUSP00000028838 | 93328\_at | E |
| ENSMUSP00000061227 | 104313\_at | E |
| ENSMUSP00000032512 | 94772\_at | ST |
| ENSMUSP00000043730 | 100912\_at | E |
| ENSMUSP00000033938 | 96217\_at | E |
| ENSMUSP00000029274 | 97661\_at | E |
| ENSMUSP00000006362 | 102212\_at | TR |
| ENSMUSP00000021471 | 97939\_at | T |
| ENSMUSP00000041427 | 102345\_at | ST |
| ENSMUSP00000028630 | 99094\_at | T |
| ENSMUSP00000033464 | 94116\_at | ST |
| ENSMUSP00000058337 | 99239\_at | ST |
| ENSMUSP00000037417 | 160123\_at | E |
| ENSMUSP00000053273 | 95693\_at | E |
| ENSMUSP00000060748 | 97005\_at | ST |
| ENSMUSP00000023519 | 98582\_at | E |
| ENSMUSP00000023207 | 93592\_at | T |
| ENSMUSP00000021259 | 98727\_at | E |
| ENSMUSP00000051368 | 103266\_at | ST |
| ENSMUSP00000031611 | 93737\_at | E |
| ENSMUSP00000049258 | 161044\_at | T |
| ENSMUSP00000060640 | 96481\_at | T |
| ENSMUSP00000005733 | 102887\_at | ST |
| ENSMUSP00000032270 | 99370\_at | ST |
| ENSMUSP00000029141 | 160665\_at | E |
| ENSMUSP00000031984 | 98991\_at | E |
| ENSMUSP00000019445 | 92557\_at | E |
| ENSMUSP00000055563 | 96890\_at | E |
| ENSMUSP00000035549 | 101719\_at | E |
| ENSMUSP00000034955 | 95430\_f\_at | E |
| ENSMUSP00000033410 | 94934\_at | ST |
| ENSMUSP00000060029 | 92833\_at | E |
| ENSMUSP00000000220 | 100150\_f\_at | ST |
| ENSMUSP00000022082 | 95722\_at | T |
| ENSMUSP00000026241 | 97742\_s\_at | ST |
| ENSMUSP00000033142 | 100406\_at | E |
| ENSMUSP00000002013 | 101850\_at | E |
| ENSMUSP00000027269 | 97155\_at | ST |
| ENSMUSP00000061951 | 100539\_at | E |
| ENSMUSP00000036783 | 93621\_at | TR |
| ENSMUSP00000028609 | 103283\_at | TR |
| ENSMUSP00000015304 | 93754\_at | E |
| ENSMUSP00000028167 | 98984\_f\_at | E |
| ENSMUSP00000022849 | 95054\_at | E |
| ENSMUSP00000059539 | 102771\_at | E |
| ENSMUSP00000036039 | 99532\_at | TR |
| ENSMUSP00000024720 | 99665\_at | TR |
| ENSMUSP00000005288 | 104071\_at | T |
| ENSMUSP00000055885 | 100670\_at | T |
| ENSMUSP00000010250 | 97431\_at | T |
| ENSMUSP00000022875 | 100948\_at | T |
| ENSMUSP00000000402 | 92441\_at | E |
| ENSMUSP00000045449 | 97709\_at | ST |
| ENSMUSP00000005488 | 103837\_at | E |
| ENSMUSP00000026001 | 101591\_at | E |
| ENSMUSP00000030112 | 100147\_at | E |
| ENSMUSP00000059325 | 93362\_at | T |
| ENSMUSP00000026328 | 93495\_at | E |
| ENSMUSP00000023108 | 103036\_at | E |
| ENSMUSP00000034543 | 104758\_at | E |
| ENSMUSP00000040505 | 102657\_at | TR |
| ENSMUSP00000025747 | 160290\_at | E |
| ENSMUSP00000042606 | 160435\_at | E |
| ENSMUSP00000018441 | 160568\_at | E |
| ENSMUSP00000057353 | 92182\_at | T |
| ENSMUSP00000055412 | 97317\_at | E |
| ENSMUSP00000062723 | 93771\_at | T |
| ENSMUSP00000000704 | 103445\_at | TR |
| ENSMUSP00000036849 | 103578\_at | T |
| ENSMUSP00000033189 | 101344\_at | ST |
| ENSMUSP00000055290 | 102800\_at | TR |
| ENSMUSP00000004326 | 102933\_at | ST |
| ENSMUSP00000002331 | 104233\_at | TR |
| ENSMUSP00000029877 | 160711\_at | E |
| ENSMUSP00000034570 | 160844\_at | E |
| ENSMUSP00000058866 | 92736\_at | T |
| ENSMUSP00000025893 | 103854\_at | E |
| ENSMUSP00000033752 | 95625\_at | T |
| ENSMUSP00000057563 | 101620\_at | T |
| ENSMUSP00000026221 | 95758\_at | E |
| ENSMUSP00000051477 | 162297\_s\_at | E |
| ENSMUSP00000014686 | 104642\_at | ST |
| ENSMUSP00000025484 | 92595\_r\_at | E |
| ENSMUSP00000058274 | 102674\_at | ST |
| ENSMUSP00000028637 | 99435\_at | ST |
| ENSMUSP00000038199 | 104119\_at | ST |
| ENSMUSP00000035844 | 97334\_at | TR |
| ENSMUSP00000025700 | 98923\_at | E |
| ENSMUSP00000053551 | 99844\_at | ST |
| ENSMUSP00000031004 | 104383\_at | E |
| ENSMUSP00000020672 | 99977\_at | T |
| ENSMUSP00000000310 | 94987\_at | E |
| ENSMUSP00000033762 | 96154\_at | E |
| ENSMUSP00000057854 | 96287\_at | E |
| ENSMUSP00000035157 | 101114\_at | ST |
| ENSMUSP00000003285 | 96696\_at | E |
| ENSMUSP00000058723 | 96708\_at | T |
| ENSMUSP00000034230 | 98008\_at | ST |
| ENSMUSP00000013807 | 160614\_at | E |
| ENSMUSP00000005087 | 104269\_at | T |
| ENSMUSP00000000788 | 98480\_s\_at | E |
| ENSMUSP00000031905 | 92873\_f\_at | E |
| ENSMUSP00000025940 | 104582\_g\_at | TR |
| ENSMUSP00000021806 | 102035\_at | E |
| ENSMUSP00000045918 | 93950\_at | TR |
| ENSMUSP00000062684 | 102168\_at | ST |
| ENSMUSP00000028997 | 92639\_at | E |
| ENSMUSP00000006764 | 93282\_at | E |
| ENSMUSP00000023737 | 99861\_at | E |
| ENSMUSP00000026579 | 96171\_at | TR |
| ENSMUSP00000001051 | 92770\_at | ST |
| ENSMUSP00000051837 | 97893\_at | TR |
| ENSMUSP00000018614 | 102577\_at | T |
| ENSMUSP00000024123 | 103900\_at | E |
| ENSMUSP00000047665 | 93512\_f\_at | E |
| ENSMUSP00000055602 | 95792\_at | ST |
| ENSMUSP00000038646 | 101932\_at | E |
| ENSMUSP00000026119 | 103498\_at | ST |
| ENSMUSP00000041104 | 96858\_at | E |
| ENSMUSP00000028129 | 101397\_at | T |
| ENSMUSP00000028807 | 104153\_at | E |
| ENSMUSP00000023101 | 104286\_at | T |
| ENSMUSP00000022099 | 160897\_at | E |
| ENSMUSP00000029646 | 100752\_at | ST |
| ENSMUSP00000027931 | 100885\_at | E |
| ENSMUSP00000035128 | 102052\_at | E |
| ENSMUSP00000015594 | 97779\_at | E |
| ENSMUSP00000055622 | 101540\_at | E |
| ENSMUSP00000034868 | 93311\_at | E |
| ENSMUSP00000036094 | 97922\_at | E |
| ENSMUSP00000043956 | 99488\_at | ST |
| ENSMUSP00000003152 | 160372\_at | E |
| ENSMUSP00000025112 | 100505\_at | E |
| ENSMUSP00000034363 | 100493\_at | E |
| ENSMUSP00000000187 | 97121\_at | ST |
| ENSMUSP00000048573 | 93720\_at | E |
| ENSMUSP00000022641 | 98976\_at | E |
| ENSMUSP00000054361 | 101281\_at | ST |
| ENSMUSP00000000287 | 98042\_at | E |
| ENSMUSP00000050773 | 104170\_at | ST |
| ENSMUSP00000057965 | 99909\_at | T |
| ENSMUSP00000015264 | 97530\_at | E |
| ENSMUSP00000032142 | 92818\_at | E |
| ENSMUSP00000026377 | 103803\_at | E |
| ENSMUSP00000040890 | 99096\_at | E |
| ENSMUSP00000030121 | 103002\_at | E |
| ENSMUSP00000021090 | 101034\_at | ST |
| ENSMUSP00000027914 | 96628\_at | E |
| ENSMUSP00000053394 | 99372\_at | ST |
| ENSMUSP00000031483 | 103544\_at | E |
| ENSMUSP00000030769 | 95448\_at | E |
| ENSMUSP00000033008 | 96892\_at | E |
| ENSMUSP00000029591 | 93214\_at | E |
| ENSMUSP00000052778 | 98337\_at | TR |
| ENSMUSP00000027675 | 99926\_at | ST |
| ENSMUSP00000025025 | 104598\_at | E |
| ENSMUSP00000025169 | 94936\_at | E |
| ENSMUSP00000025585 | 92690\_at | T |
| ENSMUSP00000028663 | 102231\_at | TR |
| ENSMUSP00000042469 | 92835\_at | E |
| ENSMUSP00000056402 | 102364\_at | TR |
| ENSMUSP00000054270 | 100130\_at | TR |
| ENSMUSP00000014578 | 101985\_at | E |
| ENSMUSP00000022646 | 97157\_at | TR |
| ENSMUSP00000021205 | 98613\_at | E |
| ENSMUSP00000032469 | 103285\_at | E |
| ENSMUSP00000044734 | 104741\_at | TR |
| ENSMUSP00000044217 | 101184\_at | E |
| ENSMUSP00000045879 | 102773\_at | E |
| ENSMUSP00000027979 | 99401\_at | E |
| ENSMUSP00000032437 | 99534\_at | ST |
| ENSMUSP00000009036 | 160551\_at | T |
| ENSMUSP00000037320 | 93088\_at | ST |
| ENSMUSP00000022212 | 92310\_at | E |
| ENSMUSP00000032170 | 97699\_at | ST |
| ENSMUSP00000026011 | 103706\_at | T |
| ENSMUSP00000016010 | 103839\_at | E |
| ENSMUSP00000027431 | 95332\_at | ST |
| ENSMUSP00000000924 | 100016\_at | E |
| ENSMUSP00000041729 | 101738\_at | ST |
| ENSMUSP00000020657 | 93509\_at | E |
| ENSMUSP00000031378 | 104482\_at | T |
| ENSMUSP00000059898 | 94953\_at | T |
| ENSMUSP00000028818 | 104627\_at | E |
| ENSMUSP00000034376 | 97975\_at | TR |
| ENSMUSP00000025200 | 94285\_at | ST |
| ENSMUSP00000031530 | 98908\_at | E |
| ENSMUSP00000030242 | 96662\_at | E |
| ENSMUSP00000041543 | 96795\_at | E |
| ENSMUSP00000054591 | 102802\_at | ST |
| ENSMUSP00000037968 | 104102\_at | E |
| ENSMUSP00000031509 | 160713\_at | T |
| ENSMUSP00000022075 | 94694\_at | E |
| ENSMUSP00000057595 | 100967\_at | E |
| ENSMUSP00000000641 | 92460\_at | ST |
| ENSMUSP00000020980 | 102001\_at | E |
| ENSMUSP00000032114 | 96139\_at | E |
| ENSMUSP00000060822 | 102267\_at | E |
| ENSMUSP00000022744 | 92738\_at | ST |
| ENSMUSP00000033418 | 103723\_at | ST |
| ENSMUSP00000047962 | 101622\_at | TR |
| ENSMUSP00000035551 | 93381\_at | ST |
| ENSMUSP00000041282 | 99960\_at | E |
| ENSMUSP00000048271 | 96270\_at | E |
| ENSMUSP00000059206 | 102376\_r\_at | ST |
| ENSMUSP00000013562 | 96548\_at | E |
| ENSMUSP00000051055 | 102410\_at | E |
| ENSMUSP00000033763 | 100575\_at | E |
| ENSMUSP00000033979 | 92213\_at | T |
| ENSMUSP00000006627 | 97336\_at | E |
| ENSMUSP00000053426 | 98780\_at | TR |
| ENSMUSP00000039910 | 103331\_at | E |
| ENSMUSP00000022725 | 103597\_at | E |
| ENSMUSP00000027952 | 95368\_at | ST |
| ENSMUSP00000026677 | 94723\_at | E |
| ENSMUSP00000046789 | 99846\_at | TR |
| ENSMUSP00000041841 | 160730\_at | TR |
| ENSMUSP00000024894 | 99979\_at | E |
| ENSMUSP00000051727 | 160996\_at | TR |
| ENSMUSP00000058624 | 100984\_at | TR |
| ENSMUSP00000040847 | 102151\_at | ST |
| ENSMUSP00000032994 | 92622\_at | T |
| ENSMUSP00000000966 | 97745\_at | TR |
| ENSMUSP00000025900 | 102429\_at | T |
| ENSMUSP00000061463 | 99045\_at | E |
| ENSMUSP00000028522 | 95511\_at | ST |
| ENSMUSP00000029686 | 94188\_at | T |
| ENSMUSP00000007131 | 160207\_at | E |
| ENSMUSP00000022227 | 98400\_at | E |
| ENSMUSP00000029465 | 103072\_at | E |
| ENSMUSP00000037418 | 99321\_at | E |
| ENSMUSP00000030266 | 104005\_at | E |
| ENSMUSP00000042202 | 104138\_at | E |
| ENSMUSP00000058020 | 100737\_at | TR |
| ENSMUSP00000022223 | 92230\_at | E |
| ENSMUSP00000030962 | 103759\_at | E |
| ENSMUSP00000044603 | 96841\_at | E |
| ENSMUSP00000062261 | 100069\_at | E |
| ENSMUSP00000051158 | 98419\_at | TR |
| ENSMUSP00000033207 | 93429\_at | ST |
| ENSMUSP00000022218 | 104547\_at | E |
| ENSMUSP00000031188 | 96796\_f\_at | E |
| ENSMUSP00000019723 | 96318\_at | ST |
| ENSMUSP00000018767 | 92917\_at | E |
| ENSMUSP00000061825 | 101801\_at | ST |
| ENSMUSP00000025078 | 97106\_at | E |
| ENSMUSP00000008966 | 93560\_at | E |
| ENSMUSP00000057050 | 160934\_s\_at | E |
| ENSMUSP00000033051 | 98828\_at | ST |
| ENSMUSP00000006914 | 103367\_at | E |
| ENSMUSP00000048239 | 161012\_at | ST |
| ENSMUSP00000028304 | 101133\_at | T |
| ENSMUSP00000048057 | 102988\_at | E |
| ENSMUSP00000025385 | 97515\_at | E |
| ENSMUSP00000032200 | 103510\_at | T |
| ENSMUSP00000036952 | 92658\_at | TR |
| ENSMUSP00000026154 | 103643\_at | TR |
| ENSMUSP00000036031 | 95547\_at | E |
| ENSMUSP00000057521 | 98291\_at | E |
| ENSMUSP00000062942 | 94902\_at | E |
| ENSMUSP00000059498 | 104697\_at | E |
| ENSMUSP00000005220 | 96202\_at | T |
| ENSMUSP00000050812 | 98112\_r\_at | E |
| ENSMUSP00000030201 | 97924\_at | E |
| ENSMUSP00000005709 | 94101\_at | TR |
| ENSMUSP00000047218 | 95084\_f\_at | E |
| ENSMUSP00000027839 | 94367\_at | E |
| ENSMUSP00000019362 | 100495\_at | ST |
| ENSMUSP00000054389 | 100507\_at | ST |
| ENSMUSP00000004485 | 92266\_at | ST |
| ENSMUSP00000031280 | 98845\_at | ST |
| ENSMUSP00000000369 | 93855\_at | E |
| ENSMUSP00000029082 | 93988\_at | E |
| ENSMUSP00000002452 | 96611\_at | T |
| ENSMUSP00000006474 | 101150\_at | TR |
| ENSMUSP00000029734 | 96744\_at | E |
| ENSMUSP00000025493 | 99500\_at | T |
| ENSMUSP00000000793 | 160650\_at | E |
| ENSMUSP00000035459 | 104172\_at | ST |
| ENSMUSP00000039205 | 94643\_at | ST |
| ENSMUSP00000061661 | 99899\_at | ST |
| ENSMUSP00000002790 | 104317\_at | T |
| ENSMUSP00000045286 | 92412\_s\_at | E |
| ENSMUSP00000024596 | 100916\_at | T |
| ENSMUSP00000053334 | 96076\_at | T |
| ENSMUSP00000035250 | 102216\_at | E |
| ENSMUSP00000055361 | 102289\_r\_at | ST |
| ENSMUSP00000025946 | 98320\_at | E |
| ENSMUSP00000004774 | 93330\_at | T |
| ENSMUSP00000003274 | 93608\_at | ST |
| ENSMUSP00000060592 | 92888\_s\_at | E |
| ENSMUSP00000015227 | 104726\_at | T |
| ENSMUSP00000045392 | 92951\_at | TR |
| ENSMUSP00000024773 | 101169\_at | ST |
| ENSMUSP00000031723 | 99374\_at | ST |
| ENSMUSP00000010807 | 160391\_at | E |
| ENSMUSP00000026572 | 160536\_at | E |
| ENSMUSP00000006718 | 98862\_at | ST |
| ENSMUSP00000015941 | 97418\_at | E |
| ENSMUSP00000029069 | 98995\_at | TR |
| ENSMUSP00000004508 | 96894\_at | T |
| ENSMUSP00000004202 | 101445\_at | E |
| ENSMUSP00000005705 | 93071\_at | T |
| ENSMUSP00000057947 | 98339\_at | T |
| ENSMUSP00000037694 | 93216\_at | ST |
| ENSMUSP00000051844 | 104334\_at | E |
| ENSMUSP00000033160 | 160812\_at | T |
| ENSMUSP00000005509 | 100933\_at | T |
| ENSMUSP00000023206 | 92704\_at | E |
| ENSMUSP00000012849 | 102366\_at | ST |
| ENSMUSP00000021892 | 97678\_r\_at | E |
| ENSMUSP00000030698 | 160277\_at | T |
| ENSMUSP00000062201 | 93480\_at | E |
| ENSMUSP00000027491 | 93625\_at | E |
| ENSMUSP00000034371 | 161065\_at | E |
| ENSMUSP00000050148 | 102642\_at | T |
| ENSMUSP00000054708 | 99391\_at | T |
| ENSMUSP00000039351 | 92445\_at | T |
| ENSMUSP00000029663 | 92578\_at | ST |
| ENSMUSP00000030723 | 100018\_at | TR |
| ENSMUSP00000036898 | 104351\_at | E |
| ENSMUSP00000022843 | 96122\_at | E |
| ENSMUSP00000005601 | 102250\_at | ST |
| ENSMUSP00000027606 | 97844\_at | ST |
| ENSMUSP00000028378 | 99011\_at | E |
| ENSMUSP00000027415 | 92987\_at | T |
| ENSMUSP00000025712 | 100282\_at | ST |
| ENSMUSP00000032348 | 100427\_at | E |
| ENSMUSP00000049039 | 92186\_at | TR |
| ENSMUSP00000026292 | 96664\_at | E |
| ENSMUSP00000031587 | 102792\_at | E |
| ENSMUSP00000048814 | 98109\_at | ST |
| ENSMUSP00000031316 | 94563\_at | T |
| ENSMUSP00000062677 | 94696\_at | ST |
| ENSMUSP00000022989 | 160848\_at | TR |
| ENSMUSP00000029633 | 100703\_at | ST |
| ENSMUSP00000019051 | 102269\_at | E |
| ENSMUSP00000033060 | 96940\_at | TR |
| ENSMUSP00000038901 | 101624\_at | T |
| ENSMUSP00000039776 | 103057\_at | E |
| ENSMUSP00000003808 | 104646\_at | ST |
| ENSMUSP00000000161 | 94171\_at | ST |
| ENSMUSP00000039048 | 99439\_at | ST |
| ENSMUSP00000006209 | 160323\_at | ST |
| ENSMUSP00000045945 | 160589\_at | E |
| ENSMUSP00000034862 | 95905\_at | T |
| ENSMUSP00000030814 | 100444\_at | E |
| ENSMUSP00000030238 | 101900\_at | E |
| ENSMUSP00000019469 | 103333\_at | E |
| ENSMUSP00000029808 | 95092\_at | E |
| ENSMUSP00000020216 | 96959\_at | E |
| ENSMUSP00000021262 | 99848\_at | E |
| ENSMUSP00000001155 | 102286\_at | ST |
| ENSMUSP00000020174 | 160064\_at | T |
| ENSMUSP00000049605 | 160197\_at | E |
| ENSMUSP00000030345 | 95646\_at | E |
| ENSMUSP00000003592 | 98390\_at | E |
| ENSMUSP00000000335 | 98535\_at | E |
| ENSMUSP00000045134 | 93412\_at | ST |
| ENSMUSP00000035172 | 103219\_at | E |
| ENSMUSP00000005771 | 104663\_at | E |
| ENSMUSP00000032471 | 96567\_at | ST |
| ENSMUSP00000003145 | 102695\_at | ST |
| ENSMUSP00000010605 | 99323\_at | ST |
| ENSMUSP00000048508 | 94200\_at | TR |
| ENSMUSP00000025007 | 160473\_at | E |
| ENSMUSP00000038505 | 100461\_at | E |
| ENSMUSP00000061124 | 98811\_at | ST |
| ENSMUSP00000033751 | 92365\_at | ST |
| ENSMUSP00000028916 | 98944\_at | T |
| ENSMUSP00000027214 | 103483\_at | E |
| ENSMUSP00000001565 | 102039\_at | TR |
| ENSMUSP00000021463 | 98143\_at | E |
| ENSMUSP00000034601 | 102971\_at | ST |
| ENSMUSP00000062704 | 104271\_at | ST |
| ENSMUSP00000019608 | 99998\_at | ST |
| ENSMUSP00000007012 | 96042\_at | E |
| ENSMUSP00000035236 | 99064\_at | E |
| ENSMUSP00000046636 | 99197\_at | T |
| ENSMUSP00000030642 | 94219\_at | E |
| ENSMUSP00000021097 | 97108\_at | E |
| ENSMUSP00000003064 | 103091\_at | TR |
| ENSMUSP00000044986 | 104680\_at | ST |
| ENSMUSP00000004233 | 93695\_at | E |
| ENSMUSP00000063168 | 92889\_r\_at | TR |
| ENSMUSP00000001411 | 101135\_at | ST |
| ENSMUSP00000061132 | 102857\_at | ST |
| ENSMUSP00000039046 | 93039\_at | E |
| ENSMUSP00000050940 | 97517\_at | E |
| ENSMUSP00000005719 | 92527\_at | E |
| ENSMUSP00000015100 | 100088\_at | E |
| ENSMUSP00000043088 | 93448\_at | E |
| ENSMUSP00000006792 | 97781\_at | ST |
| ENSMUSP00000018061 | 92803\_at | ST |
| ENSMUSP00000023732 | 92750\_s\_at | ST |
| ENSMUSP00000050375 | 94236\_at | ST |
| ENSMUSP00000042860 | 94369\_at | E |
| ENSMUSP00000029441 | 100364\_at | T |
| ENSMUSP00000037317 | 100497\_at | T |
| ENSMUSP00000017451 | 103386\_at | E |
| ENSMUSP00000005218 | 103005\_s\_at | ST |
| ENSMUSP00000022496 | 93857\_at | T |
| ENSMUSP00000038884 | 101152\_at | ST |
| ENSMUSP00000003461 | 96879\_at | E |
| ENSMUSP00000031615 | 102874\_at | E |
| ENSMUSP00000046090 | 104174\_at | E |
| ENSMUSP00000061361 | 160652\_at | E |
| ENSMUSP00000029345 | 100773\_at | ST |
| ENSMUSP00000026845 | 102218\_at | ST |
| ENSMUSP00000031598 | 95433\_at | E |
| ENSMUSP00000027288 | 101706\_at | T |
| ENSMUSP00000019044 | 98322\_at | T |
| ENSMUSP00000031935 | 101839\_at | E |
| ENSMUSP00000032865 | 98588\_at | E |
| ENSMUSP00000047771 | 103006\_at | TR |
| ENSMUSP00000034533 | 99376\_at | T |
| ENSMUSP00000029358 | 93741\_at | T |
| ENSMUSP00000021832 | 103415\_at | E |
| ENSMUSP00000026466 | 103548\_at | ST |
| ENSMUSP00000029084 | 95319\_at | ST |
| ENSMUSP00000033990 | 102891\_at | E |
| ENSMUSP00000007251 | 99652\_at | E |
| ENSMUSP00000041442 | 94795\_at | E |
| ENSMUSP00000031018 | 104336\_at | E |
| ENSMUSP00000018295 | 97684\_at | E |
| ENSMUSP00000021662 | 92561\_at | E |
| ENSMUSP00000053923 | 92694\_at | E |
| ENSMUSP00000032920 | 97829\_at | E |
| ENSMUSP00000030407 | 102235\_at | TR |
| ENSMUSP00000035877 | 101886\_f\_at | ST |
| ENSMUSP00000006164 | 94139\_at | T |
| ENSMUSP00000002101 | 100001\_at | ST |
| ENSMUSP00000038232 | 103023\_at | E |
| ENSMUSP00000023538 | 93482\_at | E |
| ENSMUSP00000050065 | 100673\_f\_at | ST |
| ENSMUSP00000036512 | 104745\_at | E |
| ENSMUSP00000027075 | 103582\_r\_at | E |
| ENSMUSP00000000962 | 92970\_at | TR |
| ENSMUSP00000017904 | 101055\_at | E |
| ENSMUSP00000059582 | 96649\_at | T |
| ENSMUSP00000028165 | 101188\_at | T |
| ENSMUSP00000012281 | 99393\_at | ST |
| ENSMUSP00000033087 | 160422\_at | E |
| ENSMUSP00000026485 | 98881\_at | E |
| ENSMUSP00000040080 | 103432\_at | E |
| ENSMUSP00000022457 | 95469\_at | E |
| ENSMUSP00000057777 | 102920\_at | T |
| ENSMUSP00000027743 | 99146\_at | T |
| ENSMUSP00000027449 | 94156\_at | ST |
| ENSMUSP00000053703 | 101740\_at | ST |
| ENSMUSP00000014126 | 100429\_at | E |
| ENSMUSP00000054914 | 98501\_at | ST |
| ENSMUSP00000035955 | 95077\_at | E |
| ENSMUSP00000023362 | 96666\_at | E |
| ENSMUSP00000041053 | 102661\_at | TR |
| ENSMUSP00000053489 | 102794\_at | ST |
| ENSMUSP00000023601 | 94432\_at | E |
| ENSMUSP00000016231 | 94698\_at | TR |
| ENSMUSP00000024916 | 100705\_at | ST |
| ENSMUSP00000029400 | 92331\_at | E |
| ENSMUSP00000019677 | 92464\_at | E |
| ENSMUSP00000021300 | 93920\_at | E |
| ENSMUSP00000047570 | 95486\_at | ST |
| ENSMUSP00000031434 | 101481\_at | T |
| ENSMUSP00000022640 | 101626\_at | E |
| ENSMUSP00000011315 | 98375\_at | ST |
| ENSMUSP00000002091 | 93252\_at | T |
| ENSMUSP00000030815 | 99831\_at | ST |
| ENSMUSP00000047413 | 93385\_at | E |
| ENSMUSP00000047400 | 94841\_at | E |
| ENSMUSP00000009828 | 103059\_at | T |
| ENSMUSP00000025786 | 104648\_at | E |
| ENSMUSP00000003981 | 99030\_at | ST |
| ENSMUSP00000052444 | 94173\_at | ST |
| ENSMUSP00000028106 | 100313\_at | ST |
| ENSMUSP00000038245 | 98784\_at | ST |
| ENSMUSP00000029935 | 103202\_at | E |
| ENSMUSP00000034349 | 93794\_at | E |
| ENSMUSP00000059224 | 96550\_at | E |
| ENSMUSP00000002846 | 96828\_at | E |
| ENSMUSP00000021922 | 102956\_at | TR |
| ENSMUSP00000023608 | 98128\_at | T |
| ENSMUSP00000031555 | 160601\_at | E |
| ENSMUSP00000031497 | 92481\_at | E |
| ENSMUSP00000057227 | 102288\_at | ST |
| ENSMUSP00000017808 | 103877\_at | E |
| ENSMUSP00000031895 | 99049\_at | E |
| ENSMUSP00000025541 | 99981\_at | ST |
| ENSMUSP00000023140 | 94190\_at | ST |
| ENSMUSP00000000127 | 99325\_at | ST |
| ENSMUSP00000036259 | 104616\_g\_at | E |
| ENSMUSP00000021920 | 100608\_at | E |
| ENSMUSP00000056282 | 103352\_at | E |
| ENSMUSP00000001042 | 93823\_at | E |
| ENSMUSP00000031123 | 95389\_at | T |
| ENSMUSP00000031129 | 96845\_at | T |
| ENSMUSP00000021691 | 101384\_at | E |
| ENSMUSP00000041983 | 104273\_at | E |
| ENSMUSP00000015237 | 160884\_at | T |
| ENSMUSP00000032472 | 94744\_at | ST |
| ENSMUSP00000026168 | 96177\_at | E |
| ENSMUSP00000021579 | 92510\_at | ST |
| ENSMUSP00000032568 | 93431\_at | E |
| ENSMUSP00000029162 | 98554\_at | E |
| ENSMUSP00000001365 | 93564\_at | E |
| ENSMUSP00000036580 | 103238\_at | ST |
| ENSMUSP00000024936 | 93709\_at | E |
| ENSMUSP00000054158 | 102581\_at | TR |
| ENSMUSP00000031767 | 102726\_at | ST |
| ENSMUSP00000021854 | 94485\_at | E |
| ENSMUSP00000031243 | 97519\_at | ST |
| ENSMUSP00000025249 | 93840\_at | T |
| ENSMUSP00000024708 | 103514\_at | ST |
| ENSMUSP00000053389 | 95418\_at | E |
| ENSMUSP00000007253 | 101546\_at | E |
| ENSMUSP00000033771 | 101679\_at | ST |
| ENSMUSP00000025968 | 98295\_at | E |
| ENSMUSP00000028823 | 98307\_at | ST |
| ENSMUSP00000021011 | 94761\_at | ST |
| ENSMUSP00000004232 | 94906\_at | E |
| ENSMUSP00000034232 | 97783\_at | ST |
| ENSMUSP00000022698 | 102334\_at | ST |
| ENSMUSP00000032491 | 92793\_at | ST |
| ENSMUSP00000036373 | 99083\_at | E |
| ENSMUSP00000054634 | 94105\_at | E |
| ENSMUSP00000045191 | 94238\_at | E |
| ENSMUSP00000047358 | 101822\_at | ST |
| ENSMUSP00000027951 | 103255\_at | ST |
| ENSMUSP00000015578 | 102876\_at | E |
| ENSMUSP00000025477 | 99504\_at | E |
| ENSMUSP00000039018 | 92977\_s\_at | TR |
| ENSMUSP00000031221 | 103797\_at | E |
| ENSMUSP00000018778 | 95290\_at | ST |
| ENSMUSP00000062902 | 101735\_f\_at | E |
| ENSMUSP00000043173 | 98324\_at | TR |
| ENSMUSP00000005812 | 98457\_at | T |
| ENSMUSP00000020575 | 104452\_at | ST |
| ENSMUSP00000001921 | 102351\_at | E |
| ENSMUSP00000026899 | 99112\_at | T |
| ENSMUSP00000025263 | 102629\_at | ST |
| ENSMUSP00000055340 | 100383\_at | E |
| ENSMUSP00000002274 | 101972\_at | E |
| ENSMUSP00000052463 | 98600\_at | ST |
| ENSMUSP00000028083 | 94263\_f\_at | E |
| ENSMUSP00000031768 | 98866\_at | TR |
| ENSMUSP00000023043 | 98999\_at | E |
| ENSMUSP00000003318 | 101171\_at | ST |
| ENSMUSP00000032180 | 101316\_at | ST |
| ENSMUSP00000060844 | 94664\_at | ST |
| ENSMUSP00000051561 | 94797\_at | T |
| ENSMUSP00000022470 | 160949\_at | E |
| ENSMUSP00000006440 | 96109\_at | TR |
| ENSMUSP00000034458 | 97553\_at | E |
| ENSMUSP00000027165 | 102237\_at | ST |
| ENSMUSP00000028874 | 92225\_f\_at | E |
| ENSMUSP00000047333 | 103826\_at | TR |
| ENSMUSP00000062590 | 103959\_at | T |
| ENSMUSP00000025245 | 95585\_at | E |
| ENSMUSP00000001830 | 101042\_f\_at | E |
| ENSMUSP00000029812 | 98427\_s\_at | TR |
| ENSMUSP00000041343 | 101725\_at | E |
| ENSMUSP00000026156 | 101858\_at | E |
| ENSMUSP00000034026 | 93351\_at | E |
| ENSMUSP00000002297 | 103025\_at | E |
| ENSMUSP00000029259 | 94940\_at | E |
| ENSMUSP00000047199 | 104614\_at | ST |
| ENSMUSP00000031780 | 99395\_at | ST |
| ENSMUSP00000020302 | 95060\_at | T |
| ENSMUSP00000034811 | 101200\_at | E |
| ENSMUSP00000037088 | 93092\_at | ST |
| ENSMUSP00000020333 | 99671\_at | E |
| ENSMUSP00000001416 | 92580\_at | E |
| ENSMUSP00000001480 | 96259\_at | E |
| ENSMUSP00000034198 | 103843\_at | ST |
| ENSMUSP00000055131 | 94025\_at | E |
| ENSMUSP00000005714 | 160165\_at | E |
| ENSMUSP00000030810 | 100020\_at | T |
| ENSMUSP00000035211 | 100286\_at | E |
| ENSMUSP00000029406 | 101742\_at | ST |
| ENSMUSP00000002839 | 101875\_at | E |
| ENSMUSP00000023087 | 93646\_at | E |
| ENSMUSP00000030538 | 101074\_at | E |
| ENSMUSP00000033498 | 96668\_at | T |
| ENSMUSP00000002284 | 102663\_at | ST |
| ENSMUSP00000026631 | 99424\_at | ST |
| ENSMUSP00000031601 | 94434\_at | TR |
| ENSMUSP00000037346 | 104108\_at | ST |
| ENSMUSP00000038485 | 100562\_at | ST |
| ENSMUSP00000014476 | 100326\_f\_at | ST |
| ENSMUSP00000025104 | 92200\_at | ST |
| ENSMUSP00000046585 | 97456\_at | E |
| ENSMUSP00000060448 | 103451\_at | E |
| ENSMUSP00000020768 | 92599\_at | E |
| ENSMUSP00000018699 | 93433\_s\_at | ST |
| ENSMUSP00000045273 | 96811\_at | E |
| ENSMUSP00000032238 | 99700\_at | ST |
| ENSMUSP00000005889 | 96511\_s\_at | ST |
| ENSMUSP00000008094 | 104372\_at | E |
| ENSMUSP00000028148 | 160850\_at | E |
| ENSMUSP00000025773 | 94843\_at | E |
| ENSMUSP00000026452 | 97732\_at | E |
| ENSMUSP00000000342 | 92742\_at | ST |
| ENSMUSP00000051959 | 99032\_at | E |
| ENSMUSP00000026012 | 102416\_at | E |
| ENSMUSP00000033913 | 160327\_at | E |
| ENSMUSP00000062083 | 98786\_at | ST |
| ENSMUSP00000030487 | 101103\_at | E |
| ENSMUSP00000027175 | 101091\_at | E |
| ENSMUSP00000045530 | 93007\_at | ST |
| ENSMUSP00000059711 | 104258\_at | E |
| ENSMUSP00000019585 | 103613\_at | E |
| ENSMUSP00000038924 | 103879\_at | E |
| ENSMUSP00000042338 | 101778\_at | T |
| ENSMUSP00000042413 | 98394\_at | TR |
| ENSMUSP00000039600 | 98406\_at | ST |
| ENSMUSP00000022592 | 93416\_at | ST |
| ENSMUSP00000009553 | 94860\_at | T |
| ENSMUSP00000030269 | 96160\_at | T |
| ENSMUSP00000024112 | 102699\_at | E |
| ENSMUSP00000035904 | 99327\_at | E |
| ENSMUSP00000020512 | 93680\_at | E |
| ENSMUSP00000002391 | 97359\_at | T |
| ENSMUSP00000041299 | 98147\_at | E |
| ENSMUSP00000000423 | 96046\_at | E |
| ENSMUSP00000025373 | 103630\_at | E |
| ENSMUSP00000055743 | 160085\_at | E |
| ENSMUSP00000022518 | 100073\_at | E |
| ENSMUSP00000054343 | 98423\_at | T |
| ENSMUSP00000043849 | 93300\_at | ST |
| ENSMUSP00000021060 | 93699\_at | E |
| ENSMUSP00000056048 | 96588\_at | ST |
| ENSMUSP00000060365 | 102583\_at | ST |
| ENSMUSP00000015481 | 160494\_at | E |
| ENSMUSP00000022844 | 160506\_at | E |
| ENSMUSP00000021005 | 98832\_at | E |
| ENSMUSP00000025186 | 103371\_at | T |
| ENSMUSP00000016172 | 103516\_at | ST |
| ENSMUSP00000029464 | 103649\_at | E |
| ENSMUSP00000020518 | 95275\_at | ST |
| ENSMUSP00000029641 | 96997\_at | T |
| ENSMUSP00000020350 | 98297\_at | ST |
| ENSMUSP00000050119 | 98309\_at | ST |
| ENSMUSP00000027038 | 99886\_at | ST |
| ENSMUSP00000006692 | 160770\_at | E |
| ENSMUSP00000018041 | 104292\_at | E |
| ENSMUSP00000033833 | 93319\_at | ST |
| ENSMUSP00000014130 | 160915\_at | E |
| ENSMUSP00000059235 | 96063\_at | E |
| ENSMUSP00000048877 | 100903\_at | E |
| ENSMUSP00000004773 | 97652\_at | ST |
| ENSMUSP00000060483 | 92807\_at | T |
| ENSMUSP00000002026 | 160114\_at | ST |
| ENSMUSP00000026315 | 94107\_at | E |
| ENSMUSP00000030069 | 98440\_at | E |
| ENSMUSP00000044517 | 93728\_at | TR |
| ENSMUSP00000025853 | 96617\_at | TR |
| ENSMUSP00000012426 | 99361\_at | ST |
| ENSMUSP00000018429 | 94371\_at | E |
| ENSMUSP00000034944 | 99506\_at | E |
| ENSMUSP00000041202 | 160789\_at | E |
| ENSMUSP00000041019 | 100511\_at | T |
| ENSMUSP00000003741 | 97405\_at | E |
| ENSMUSP00000026613 | 97538\_at | E |
| ENSMUSP00000040412 | 92415\_at | ST |
| ENSMUSP00000025579 | 102104\_f\_at | ST |
| ENSMUSP00000035423 | 103666\_at | TR |
| ENSMUSP00000006776 | 95304\_at | E |
| ENSMUSP00000021672 | 93336\_at | T |
| ENSMUSP00000018744 | 98459\_at | E |
| ENSMUSP00000031325 | 99915\_at | ST |
| ENSMUSP00000011029 | 94925\_at | ST |
| ENSMUSP00000035053 | 92824\_at | E |
| ENSMUSP00000000299 | 102353\_at | ST |
| ENSMUSP00000033392 | 92957\_at | ST |
| ENSMUSP00000002891 | 94124\_at | E |
| ENSMUSP00000057660 | 94257\_at | E |
| ENSMUSP00000034243 | 93612\_at | E |
| ENSMUSP00000020368 | 92289\_at | E |
| ENSMUSP00000028076 | 93878\_at | TR |
| ENSMUSP00000037045 | 101318\_at | ST |
| ENSMUSP00000001974 | 99656\_at | E |
| ENSMUSP00000027927 | 97422\_at | E |
| ENSMUSP00000056067 | 93455\_s\_at | ST |
| ENSMUSP00000022718 | 103550\_at | ST |
| ENSMUSP00000034166 | 103683\_at | E |
| ENSMUSP00000033248 | 96910\_at | E |
| ENSMUSP00000033229 | 97180\_f\_at | T |
| ENSMUSP00000034267 | 93486\_at | E |
| ENSMUSP00000033501 | 104471\_at | E |
| ENSMUSP00000003445 | 97964\_at | E |
| ENSMUSP00000031251 | 102370\_at | E |
| ENSMUSP00000062072 | 92816\_r\_at | E |
| ENSMUSP00000005678 | 94141\_at | ST |
| ENSMUSP00000019535 | 94274\_at | E |
| ENSMUSP00000001475 | 94419\_at | T |
| ENSMUSP00000004265 | 160426\_at | E |
| ENSMUSP00000037259 | 101991\_at | E |
| ENSMUSP00000038717 | 101335\_at | E |
| ENSMUSP00000034405 | 94376\_s\_at | E |
| ENSMUSP00000034946 | 94550\_at | T |
| ENSMUSP00000016208 | 100956\_at | E |
| ENSMUSP00000027697 | 96128\_at | E |
| ENSMUSP00000001983 | 92582\_at | T |
| ENSMUSP00000025694 | 102123\_at | E |
| ENSMUSP00000032732 | 92727\_at | T |
| ENSMUSP00000060544 | 94027\_at | ST |
| ENSMUSP00000037302 | 95471\_at | E |
| ENSMUSP00000045536 | 160146\_r\_at | E |
| ENSMUSP00000035914 | 98360\_at | T |
| ENSMUSP00000034352 | 93515\_at | E |
| ENSMUSP00000044335 | 104500\_at | E |
| ENSMUSP00000033054 | 102798\_at | ST |
| ENSMUSP00000058490 | 99426\_at | TR |
| ENSMUSP00000050692 | 99559\_at | E |
| ENSMUSP00000001716 | 100564\_at | E |
| ENSMUSP00000035265 | 97325\_at | E |
| ENSMUSP00000002073 | 92335\_at | E |
| ENSMUSP00000020602 | 102009\_at | E |
| ENSMUSP00000026667 | 101485\_at | E |
| ENSMUSP00000033919 | 94712\_at | ST |
| ENSMUSP00000021506 | 104374\_at | E |
| ENSMUSP00000016338 | 97867\_at | E |
| ENSMUSP00000034181 | 99034\_at | TR |
| ENSMUSP00000027989 | 94177\_at | E |
| ENSMUSP00000031359 | 100317\_at | E |
| ENSMUSP00000028507 | 103061\_at | E |
| ENSMUSP00000004964 | 98788\_at | TR |
| ENSMUSP00000024099 | 104650\_at | E |
| ENSMUSP00000024738 | 96421\_at | E |
| ENSMUSP00000012348 | 93009\_at | E |
| ENSMUSP00000020580 | 160738\_at | E |
| ENSMUSP00000037110 | 92352\_at | ST |
| ENSMUSP00000028921 | 97475\_at | E |
| ENSMUSP00000024642 | 93941\_at | TR |
| ENSMUSP00000024049 | 92485\_at | ST |
| ENSMUSP00000053011 | 98130\_at | T |
| ENSMUSP00000025944 | 98408\_at | TR |
| ENSMUSP00000026571 | 104669\_at | TR |
| ENSMUSP00000025542 | 96295\_at | E |
| ENSMUSP00000000329 | 102290\_at | E |
| ENSMUSP00000031749 | 102568\_at | TR |
| ENSMUSP00000023805 | 99184\_at | E |
| ENSMUSP00000019183 | 160346\_at | E |
| ENSMUSP00000028610 | 160479\_at | E |
| ENSMUSP00000001126 | 94339\_at | T |
| ENSMUSP00000055667 | 101923\_at | E |
| ENSMUSP00000043613 | 93682\_at | TR |
| ENSMUSP00000050156 | 96849\_at | T |
| ENSMUSP00000058677 | 101388\_at | E |
| ENSMUSP00000008684 | 93026\_at | E |
| ENSMUSP00000030296 | 97492\_at | T |
| ENSMUSP00000021813 | 92514\_at | TR |
| ENSMUSP00000029881 | 98280\_at | E |
| ENSMUSP00000043926 | 93290\_at | E |
| ENSMUSP00000047954 | 93435\_at | E |
| ENSMUSP00000006366 | 99479\_at | ST |
| ENSMUSP00000060765 | 94489\_at | E |
| ENSMUSP00000015394 | 100484\_at | E |
| ENSMUSP00000019965 | 101940\_at | E |
| ENSMUSP00000055177 | 92255\_at | TR |
| ENSMUSP00000020024 | 98967\_at | T |
| ENSMUSP00000046474 | 96999\_at | ST |
| ENSMUSP00000056082 | 102861\_at | T |
| ENSMUSP00000000003 | 94632\_at | T |
| ENSMUSP00000025851 | 104306\_at | E |
| ENSMUSP00000010209 | 104439\_at | E |
| ENSMUSP00000020258 | 100905\_at | E |
| ENSMUSP00000030922 | 96198\_at | E |
| ENSMUSP00000038005 | 102338\_at | E |
| ENSMUSP00000048520 | 103782\_at | T |
| ENSMUSP00000055509 | 95420\_at | E |
| ENSMUSP00000052872 | 98575\_at | E |
| ENSMUSP00000003137 | 93585\_at | E |
| ENSMUSP00000029645 | 99269\_g\_at | E |
| ENSMUSP00000034148 | 161037\_at | ST |
| ENSMUSP00000039127 | 99508\_at | T |
| ENSMUSP00000004407 | 100779\_at | ST |
| ENSMUSP00000021043 | 94166\_g\_at | ST |
| ENSMUSP00000046920 | 95306\_at | ST |
| ENSMUSP00000027534 | 96750\_at | E |
| ENSMUSP00000029325 | 95439\_at | E |
| ENSMUSP00000062256 | 93193\_at | ST |
| ENSMUSP00000021346 | 98328\_at | ST |
| ENSMUSP00000062742 | 92959\_at | E |
| ENSMUSP00000029429 | 94126\_at | ST |
| ENSMUSP00000029946 | 93614\_at | E |
| ENSMUSP00000025242 | 101175\_at | E |
| ENSMUSP00000032742 | 102631\_at | E |
| ENSMUSP00000041702 | 99380\_at | T |
| ENSMUSP00000028170 | 100530\_at | ST |
| ENSMUSP00000036403 | 104635\_r\_at | T |
| ENSMUSP00000040001 | 92434\_at | ST |
| ENSMUSP00000062552 | 101729\_at | T |
| ENSMUSP00000025931 | 103029\_at | E |
| ENSMUSP00000031131 | 96244\_at | E |
| ENSMUSP00000046388 | 97966\_at | E |
| ENSMUSP00000010239 | 99133\_at | E |
| ENSMUSP00000005849 | 99399\_at | ST |
| ENSMUSP00000028619 | 94276\_at | E |
| ENSMUSP00000043806 | 100416\_at | E |
| ENSMUSP00000020531 | 100549\_at | T |
| ENSMUSP00000006181 | 98887\_at | T |
| ENSMUSP00000037348 | 95064\_at | E |
| ENSMUSP00000031591 | 101192\_at | TR |
| ENSMUSP00000031937 | 101337\_at | E |
| ENSMUSP00000025224 | 102926\_at | ST |
| ENSMUSP00000060774 | 99542\_at | E |
| ENSMUSP00000022794 | 97441\_at | E |
| ENSMUSP00000005964 | 98625\_s\_at | E |
| ENSMUSP00000059559 | 92584\_at | E |
| ENSMUSP00000019896 | 103714\_at | E |
| ENSMUSP00000034211 | 95340\_at | T |
| ENSMUSP00000000822 | 103046\_at | E |
| ENSMUSP00000055238 | 102401\_at | TR |
| ENSMUSP00000010044 | 102667\_at | ST |
| ENSMUSP00000059801 | 94438\_at | E |
| ENSMUSP00000029866 | 97182\_at | E |
| ENSMUSP00000025651 | 97327\_at | E |
| ENSMUSP00000023282 | 93781\_at | E |
| ENSMUSP00000029676 | 103588\_at | E |
| ENSMUSP00000031897 | 96670\_at | E |
| ENSMUSP00000015950 | 96948\_at | E |
| ENSMUSP00000032457 | 102943\_at | E |
| ENSMUSP00000034641 | 93258\_at | E |
| ENSMUSP00000046515 | 160721\_at | TR |
| ENSMUSP00000040307 | 160854\_at | E |
| ENSMUSP00000030672 | 96014\_at | T |
| ENSMUSP00000029386 | 97869\_at | E |
| ENSMUSP00000054510 | 92746\_at | E |
| ENSMUSP00000031032 | 92879\_at | E |
| ENSMUSP00000006397 | 103997\_at | ST |
| ENSMUSP00000034863 | 94046\_at | E |
| ENSMUSP00000039920 | 104652\_at | T |
| ENSMUSP00000029727 | 93667\_at | TR |
| ENSMUSP00000048541 | 103208\_at | T |
| ENSMUSP00000014747 | 96423\_at | TR |
| ENSMUSP00000022977 | 94322\_at | E |
| ENSMUSP00000023104 | 160595\_at | E |
| ENSMUSP00000035075 | 93002\_r\_at | ST |
| ENSMUSP00000019744 | 104129\_at | E |
| ENSMUSP00000027405 | 98800\_at | T |
| ENSMUSP00000039335 | 97477\_at | T |
| ENSMUSP00000043755 | 98933\_at | E |
| ENSMUSP00000008035 | 93810\_at | E |
| ENSMUSP00000028087 | 160821\_r\_at | E |
| ENSMUSP00000052809 | 95376\_at | T |
| ENSMUSP00000053484 | 98132\_at | T |
| ENSMUSP00000026703 | 93142\_at | TR |
| ENSMUSP00000019918 | 99854\_at | E |
| ENSMUSP00000030804 | 160871\_at | E |
| ENSMUSP00000018113 | 104538\_at | E |
| ENSMUSP00000048111 | 97886\_at | E |
| ENSMUSP00000029644 | 103881\_at | E |
| ENSMUSP00000026081 | 160070\_at | E |
| ENSMUSP00000029700 | 94063\_at | ST |
| ENSMUSP00000004330 | 94196\_at | E |
| ENSMUSP00000024793 | 100469\_at | TR |
| ENSMUSP00000059717 | 103080\_at | E |
| ENSMUSP00000043204 | 93551\_at | E |
| ENSMUSP00000062878 | 94138\_s\_at | E |
| ENSMUSP00000052209 | 103225\_at | E |
| ENSMUSP00000062032 | 98018\_at | ST |
| ENSMUSP00000017629 | 99462\_at | E |
| ENSMUSP00000052402 | 97001\_r\_at | ST |
| ENSMUSP00000033283 | 100612\_at | E |
| ENSMUSP00000030399 | 98950\_at | E |
| ENSMUSP00000025509 | 92516\_at | E |
| ENSMUSP00000055217 | 102337\_s\_at | ST |
| ENSMUSP00000062506 | 103634\_at | TR |
| ENSMUSP00000059764 | 98282\_at | ST |
| ENSMUSP00000024944 | 93304\_at | E |
| ENSMUSP00000001720 | 96326\_at | E |
| ENSMUSP00000003448 | 102321\_at | E |
| ENSMUSP00000026217 | 99070\_at | E |
| ENSMUSP00000022062 | 94080\_at | E |
| ENSMUSP00000026252 | 160498\_at | TR |
| ENSMUSP00000007116 | 100486\_at | E |
| ENSMUSP00000047037 | 101942\_at | E |
| ENSMUSP00000025503 | 97247\_at | E |
| ENSMUSP00000063026 | 102863\_at | T |
| ENSMUSP00000020278 | 94634\_at | ST |
| ENSMUSP00000053355 | 160774\_at | E |
| ENSMUSP00000033053 | 104308\_at | ST |
| ENSMUSP00000040227 | 92400\_at | E |
| ENSMUSP00000032907 | 92533\_at | ST |
| ENSMUSP00000036591 | 97789\_at | TR |
| ENSMUSP00000019516 | 102207\_at | E |
| ENSMUSP00000052181 | 92666\_at | ST |
| ENSMUSP00000000983 | 160118\_at | E |
| ENSMUSP00000048519 | 95688\_at | E |
| ENSMUSP00000003527 | 100094\_at | TR |
| ENSMUSP00000058345 | 103651\_r\_at | TR |
| ENSMUSP00000032201 | 101828\_at | E |
| ENSMUSP00000043436 | 99900\_at | TR |
| ENSMUSP00000028923 | 93454\_at | ST |
| ENSMUSP00000005685 | 102847\_s\_at | E |
| ENSMUSP00000055000 | 161039\_at | E |
| ENSMUSP00000021158 | 94242\_at | T |
| ENSMUSP00000029909 | 99365\_at | E |
| ENSMUSP00000000642 | 94375\_at | E |
| ENSMUSP00000035870 | 100370\_at | T |
| ENSMUSP00000018066 | 92274\_at | ST |
| ENSMUSP00000048001 | 97397\_at | E |
| ENSMUSP00000028353 | 98853\_at | ST |
| ENSMUSP00000034081 | 103392\_at | E |
| ENSMUSP00000026552 | 93996\_at | E |
| ENSMUSP00000005400 | 95030\_at | ST |
| ENSMUSP00000002398 | 95308\_at | E |
| ENSMUSP00000026557 | 98052\_at | T |
| ENSMUSP00000031365 | 101436\_at | ST |
| ENSMUSP00000023295 | 93207\_at | E |
| ENSMUSP00000018156 | 104180\_at | E |
| ENSMUSP00000029053 | 94929\_at | E |
| ENSMUSP00000015763 | 100924\_at | TR |
| ENSMUSP00000034602 | 92683\_at | ST |
| ENSMUSP00000029055 | 92828\_at | E |
| ENSMUSP00000023432 | 160135\_at | E |
| ENSMUSP00000026381 | 103278\_at | E |
| ENSMUSP00000026013 | 93749\_at | E |
| ENSMUSP00000026124 | 96360\_at | ST |
| ENSMUSP00000030090 | 101044\_at | E |
| ENSMUSP00000015891 | 94404\_at | T |
| ENSMUSP00000029046 | 160544\_at | T |
| ENSMUSP00000002081 | 92436\_at | E |
| ENSMUSP00000011400 | 103554\_at | E |
| ENSMUSP00000003863 | 101453\_at | ST |
| ENSMUSP00000033689 | 98347\_at | TR |
| ENSMUSP00000025463 | 96113\_at | E |
| ENSMUSP00000045146 | 92712\_at | E |
| ENSMUSP00000022792 | 92845\_at | E |
| ENSMUSP00000021595 | 160152\_at | E |
| ENSMUSP00000056720 | 94145\_at | ST |
| ENSMUSP00000018569 | 160285\_at | E |
| ENSMUSP00000045569 | 101862\_at | E |
| ENSMUSP00000043320 | 93500\_at | E |
| ENSMUSP00000062221 | 98942\_r\_at | E |
| ENSMUSP00000053188 | 93899\_at | E |
| ENSMUSP00000026576 | 95066\_at | E |
| ENSMUSP00000050898 | 99411\_at | ST |
| ENSMUSP00000028672 | 160561\_at | ST |
| ENSMUSP00000023687 | 160694\_at | ST |
| ENSMUSP00000062051 | 94687\_at | TR |
| ENSMUSP00000030826 | 160839\_at | T |
| ENSMUSP00000022322 | 92586\_at | E |
| ENSMUSP00000052123 | 95342\_at | ST |
| ENSMUSP00000032402 | 100026\_at | E |
| ENSMUSP00000045335 | 101748\_at | ST |
| ENSMUSP00000006439 | 98497\_at | ST |
| ENSMUSP00000041082 | 99953\_at | ST |
| ENSMUSP00000045993 | 103048\_at | TR |
| ENSMUSP00000020286 | 160314\_at | E |
| ENSMUSP00000029570 | 160447\_at | T |
| ENSMUSP00000036570 | 100302\_at | TR |
| ENSMUSP00000052581 | 100435\_at | ST |
| ENSMUSP00000023775 | 93783\_at | E |
| ENSMUSP00000019986 | 103457\_at | E |
| ENSMUSP00000054463 | 96672\_at | TR |
| ENSMUSP00000053616 | 101356\_at | E |
| ENSMUSP00000050414 | 94571\_at | ST |
| ENSMUSP00000062670 | 104378\_at | E |
| ENSMUSP00000038813 | 97460\_at | E |
| ENSMUSP00000006669 | 100977\_at | E |
| ENSMUSP00000020550 | 94048\_at | E |
| ENSMUSP00000027559 | 101632\_at | ST |
| ENSMUSP00000021397 | 99970\_at | E |
| ENSMUSP00000028964 | 94457\_at | T |
| ENSMUSP00000031324 | 98802\_at | ST |
| ENSMUSP00000044853 | 97479\_at | E |
| ENSMUSP00000029433 | 92356\_at | E |
| ENSMUSP00000030381 | 103341\_at | E |
| ENSMUSP00000031513 | 96834\_at | T |
| ENSMUSP00000032264 | 93011\_at | ST |
| ENSMUSP00000051433 | 104262\_at | ST |
| ENSMUSP00000026441 | 100994\_at | E |
| ENSMUSP00000026569 | 97755\_at | ST |
| ENSMUSP00000033292 | 97888\_at | ST |
| ENSMUSP00000043066 | 102306\_at | E |
| ENSMUSP00000037487 | 92898\_at | E |
| ENSMUSP00000021169 | 99055\_at | E |
| ENSMUSP00000040840 | 160217\_at | E |
| ENSMUSP00000007257 | 95654\_at | T |
| ENSMUSP00000015667 | 98543\_at | E |
| ENSMUSP00000005829 | 104671\_at | E |
| ENSMUSP00000031181 | 103227\_at | E |
| ENSMUSP00000022377 | 161005\_at | T |
| ENSMUSP00000045263 | 99331\_at | E |
| ENSMUSP00000059813 | 99464\_at | ST |
| ENSMUSP00000029804 | 104015\_at | E |
| ENSMUSP00000029017 | 160481\_at | E |
| ENSMUSP00000019037 | 100614\_at | T |
| ENSMUSP00000059308 | 97363\_at | E |
| ENSMUSP00000021314 | 102047\_at | E |
| ENSMUSP00000047721 | 103503\_at | E |
| ENSMUSP00000020241 | 95407\_at | E |
| ENSMUSP00000021257 | 101402\_at | ST |
| ENSMUSP00000020171 | 93294\_at | ST |
| ENSMUSP00000049898 | 99873\_at | TR |
| ENSMUSP00000030429 | 98429\_at | E |
| ENSMUSP00000005003 | 160890\_at | ST |
| ENSMUSP00000051467 | 96053\_i\_at | E |
| ENSMUSP00000022368 | 97772\_at | E |
| ENSMUSP00000055525 | 92927\_at | TR |
| ENSMUSP00000005548 | 160101\_at | E |
| ENSMUSP00000053422 | 100488\_at | E |
| ENSMUSP00000002681 | 97249\_at | E |
| ENSMUSP00000034218 | 93570\_at | T |
| ENSMUSP00000030583 | 95148\_at | E |
| ENSMUSP00000047004 | 96592\_at | E |
| ENSMUSP00000041825 | 101143\_at | ST |
| ENSMUSP00000034860 | 102998\_at | E |
| ENSMUSP00000004904 | 93047\_at | T |
| ENSMUSP00000022803 | 101558\_s\_at | E |
| ENSMUSP00000040599 | 104165\_at | E |
| ENSMUSP00000019911 | 160643\_at | E |
| ENSMUSP00000039170 | 94636\_at | TR |
| ENSMUSP00000018765 | 94769\_at | E |
| ENSMUSP00000006875 | 100764\_at | ST |
| ENSMUSP00000061482 | 100909\_at | E |
| ENSMUSP00000047945 | 103520\_at | ST |
| ENSMUSP00000027015 | 102064\_at | E |
| ENSMUSP00000033617 | 92668\_at | E |
| ENSMUSP00000060102 | 103653\_at | E |
| ENSMUSP00000046312 | 102209\_at | TR |
| ENSMUSP00000059138 | 101552\_at | T |
| ENSMUSP00000000500 | 99890\_at | ST |
| ENSMUSP00000006991 | 99902\_at | T |
| ENSMUSP00000039559 | 98579\_at | TR |
| ENSMUSP00000022653 | 93589\_at | E |
| ENSMUSP00000060727 | 104719\_at | T |
| ENSMUSP00000035029 | 92811\_at | T |
| ENSMUSP00000000430 | 97934\_at | E |
| ENSMUSP00000005618 | 160384\_at | E |
| ENSMUSP00000030677 | 92276\_at | E |
| ENSMUSP00000059335 | 103394\_at | T |
| ENSMUSP00000006349 | 103539\_at | E |
| ENSMUSP00000003515 | 96621\_at | E |
| ENSMUSP00000031326 | 101160\_at | ST |
| ENSMUSP00000051649 | 101293\_at | E |
| ENSMUSP00000052088 | 101305\_at | TR |
| ENSMUSP00000030985 | 104182\_at | E |
| ENSMUSP00000003908 | 160938\_at | E |
| ENSMUSP00000049082 | 103670\_at | E |
| ENSMUSP00000038158 | 92685\_at | ST |
| ENSMUSP00000047457 | 103948\_at | TR |
| ENSMUSP00000057863 | 95441\_at | T |
| ENSMUSP00000006659 | 101874\_s\_at | ST |
| ENSMUSP00000060247 | 160137\_at | E |
| ENSMUSP00000016294 | 101714\_at | E |
| ENSMUSP00000034707 | 98463\_at | E |
| ENSMUSP00000046324 | 104603\_at | E |
| ENSMUSP00000001867 | 96507\_at | TR |
| ENSMUSP00000018842 | 92961\_at | TR |
| ENSMUSP00000025941 | 102635\_at | T |
| ENSMUSP00000042150 | 94394\_at | E |
| ENSMUSP00000017534 | 160546\_at | E |
| ENSMUSP00000023835 | 97150\_at | T |
| ENSMUSP00000050852 | 98872\_at | E |
| ENSMUSP00000049295 | 96771\_at | E |
| ENSMUSP00000038576 | 102911\_at | TR |
| ENSMUSP00000045216 | 101588\_at | T |
| ENSMUSP00000033720 | 93081\_at | TR |
| ENSMUSP00000001548 | 104211\_at | ST |
| ENSMUSP00000022276 | 98349\_at | ST |
| ENSMUSP00000026907 | 104344\_at | E |
| ENSMUSP00000047393 | 94815\_at | E |
| ENSMUSP00000029038 | 160729\_f\_at | T |
| ENSMUSP00000004634 | 100943\_at | T |
| ENSMUSP00000051488 | 97692\_at | ST |
| ENSMUSP00000057199 | 102243\_at | TR |
| ENSMUSP00000039586 | 94147\_at | E |
| ENSMUSP00000003250 | 100142\_at | ST |
| ENSMUSP00000026305 | 103297\_at | E |
| ENSMUSP00000026318 | 96657\_at | E |
| ENSMUSP00000051527 | 102652\_at | TR |
| ENSMUSP00000026911 | 99413\_at | ST |
| ENSMUSP00000007130 | 160430\_at | TR |
| ENSMUSP00000021220 | 92794\_f\_at | E |
| ENSMUSP00000003493 | 100684\_at | E |
| ENSMUSP00000054112 | 93911\_at | E |
| ENSMUSP00000025800 | 103573\_at | E |
| ENSMUSP00000033646 | 95344\_at | ST |
| ENSMUSP00000009143 | 93243\_at | ST |
| ENSMUSP00000028499 | 98366\_at | ST |
| ENSMUSP00000033389 | 97721\_at | ST |
| ENSMUSP00000027878 | 99021\_at | TR |
| ENSMUSP00000032421 | 94164\_at | E |
| ENSMUSP00000027097 | 102672\_g\_at | TR |
| ENSMUSP00000061503 | 94297\_at | E |
| ENSMUSP00000054404 | 97053\_at | ST |
| ENSMUSP00000018476 | 98775\_at | E |
| ENSMUSP00000001882 | 93785\_at | ST |
| ENSMUSP00000025912 | 101358\_at | E |
| ENSMUSP00000054062 | 99430\_at | ST |
| ENSMUSP00000041186 | 102947\_at | T |
| ENSMUSP00000053251 | 93129\_at | TR |
| ENSMUSP00000049471 | 102013\_at | E |
| ENSMUSP00000044453 | 103602\_at | E |
| ENSMUSP00000035216 | 102279\_at | E |
| ENSMUSP00000006716 | 103735\_at | ST |
| ENSMUSP00000037752 | 99972\_at | E |
| ENSMUSP00000016621 | 93405\_at | TR |
| ENSMUSP00000035660 | 93538\_at | ST |
| ENSMUSP00000016491 | 99449\_at | T |
| ENSMUSP00000030741 | 100454\_at | E |
| ENSMUSP00000006263 | 103210\_at | ST |
| ENSMUSP00000041999 | 98804\_at | TR |
| ENSMUSP00000026142 | 96703\_at | TR |
| ENSMUSP00000026755 | 98136\_at | E |
| ENSMUSP00000002950 | 104131\_at | ST |
| ENSMUSP00000004968 | 160742\_at | E |
| ENSMUSP00000006838 | 94868\_at | E |
| ENSMUSP00000022124 | 100730\_at | ST |
| ENSMUSP00000046969 | 96035\_at | E |
| ENSMUSP00000028904 | 102296\_at | E |
| ENSMUSP00000020400 | 160074\_at | E |
| ENSMUSP00000030155 | 101651\_at | ST |
| ENSMUSP00000004375 | 98545\_at | ST |
| ENSMUSP00000061045 | 93688\_at | E |
| ENSMUSP00000020978 | 103229\_at | ST |
| ENSMUSP00000022221 | 102572\_at | ST |
| ENSMUSP00000020440 | 94210\_at | T |
| ENSMUSP00000053540 | 160350\_at | E |
| ENSMUSP00000033634 | 104017\_at | E |
| ENSMUSP00000025641 | 95932\_at | ST |
| ENSMUSP00000006544 | 160628\_at | E |
| ENSMUSP00000006952 | 92242\_at | T |
| ENSMUSP00000031648 | 98821\_at | TR |
| ENSMUSP00000033321 | 102248\_f\_at | E |
| ENSMUSP00000052874 | 96986\_at | ST |
| ENSMUSP00000052823 | 101537\_at | E |
| ENSMUSP00000060590 | 97774\_at | ST |
| ENSMUSP00000026129 | 103914\_at | E |
| ENSMUSP00000028430 | 92929\_at | T |
| ENSMUSP00000056325 | 160091\_at | E |
| ENSMUSP00000031737 | 94229\_at | TR |
| ENSMUSP00000020767 | 104690\_at | E |
| ENSMUSP00000000194 | 93717\_at | ST |
| ENSMUSP00000025170 | 104702\_at | E |
| ENSMUSP00000030264 | 101278\_at | ST |
| ENSMUSP00000019937 | 99350\_at | T |
| ENSMUSP00000058674 | 97527\_at | E |
| ENSMUSP00000023015 | 92404\_at | ST |
| ENSMUSP00000029752 | 102199\_at | E |
| ENSMUSP00000033941 | 93981\_at | E |
| ENSMUSP00000037981 | 103655\_at | E |
| ENSMUSP00000026538 | 95426\_at | E |
| ENSMUSP00000023116 | 96870\_at | E |
| ENSMUSP00000055797 | 99892\_at | ST |
| ENSMUSP00000004786 | 93325\_at | E |
| ENSMUSP00000058713 | 92192\_s\_at | E |
| ENSMUSP00000021028 | 99904\_at | ST |
| ENSMUSP00000062918 | 93458\_at | TR |
| ENSMUSP00000040588 | 104443\_at | ST |
| ENSMUSP00000028944 | 160921\_at | E |
| ENSMUSP00000060446 | 97791\_at | ST |
| ENSMUSP00000003284 | 99103\_at | TR |
| ENSMUSP00000038536 | 103931\_at | ST |
| ENSMUSP00000028306 | 99236\_at | T |
| ENSMUSP00000023612 | 94246\_at | TR |
| ENSMUSP00000035010 | 95702\_at | E |
| ENSMUSP00000025755 | 100374\_at | TR |
| ENSMUSP00000021933 | 101963\_at | E |
| ENSMUSP00000052256 | 103408\_at | E |
| ENSMUSP00000033958 | 100526\_f\_at | E |
| ENSMUSP00000030483 | 101307\_at | E |
| ENSMUSP00000003687 | 102751\_at | ST |
| ENSMUSP00000030883 | 104184\_at | ST |
| ENSMUSP00000001240 | 160807\_at | E |
| ENSMUSP00000029248 | 97411\_at | ST |
| ENSMUSP00000031338 | 95310\_at | ST |
| ENSMUSP00000004377 | 102851\_s\_at | E |
| ENSMUSP00000005019 | 100127\_at | T |
| ENSMUSP00000041372 | 101716\_at | E |
| ENSMUSP00000021235 | 104460\_at | T |
| ENSMUSP00000046168 | 96231\_at | E |
| ENSMUSP00000021114 | 97820\_at | E |
| ENSMUSP00000025583 | 96509\_at | ST |
| ENSMUSP00000047041 | 99386\_at | TR |
| ENSMUSP00000020763 | 100403\_at | E |
| ENSMUSP00000033153 | 95329\_at | E |
| ENSMUSP00000021909 | 96918\_at | E |
| ENSMUSP00000025965 | 93228\_at | E |
| ENSMUSP00000051717 | 104213\_at | E |
| ENSMUSP00000036765 | 104479\_at | ST |
| ENSMUSP00000019071 | 92849\_at | ST |
| ENSMUSP00000025979 | 95738\_at | E |
| ENSMUSP00000047894 | 100277\_at | ST |
| ENSMUSP00000055009 | 101733\_at | ST |
| ENSMUSP00000040442 | 101866\_at | E |
| ENSMUSP00000006005 | 98482\_at | ST |
| ENSMUSP00000051125 | 103299\_at | E |
| ENSMUSP00000025571 | 93637\_at | ST |
| ENSMUSP00000056119 | 104622\_at | TR |
| ENSMUSP00000053594 | 101065\_at | E |
| ENSMUSP00000008090 | 92980\_at | TR |
| ENSMUSP00000033502 | 102654\_at | TR |
| ENSMUSP00000039660 | 99415\_at | ST |
| ENSMUSP00000019246 | 99548\_at | E |
| ENSMUSP00000060377 | 160565\_at | E |
| ENSMUSP00000055333 | 98102\_at | E |
| ENSMUSP00000002949 | 93112\_at | E |
| ENSMUSP00000031456 | 94701\_at | E |
| ENSMUSP00000001703 | 93378\_at | TR |
| ENSMUSP00000017881 | 99957\_at | E |
| ENSMUSP00000034915 | 94834\_at | E |
| ENSMUSP00000027777 | 101957\_f\_at | E |
| ENSMUSP00000056368 | 92733\_at | E |
| ENSMUSP00000022355 | 97989\_at | E |
| ENSMUSP00000046105 | 92866\_at | ST |
| ENSMUSP00000003215 | 99023\_at | E |
| ENSMUSP00000030893 | 100294\_at | E |
| ENSMUSP00000023167 | 93654\_at | ST |
| ENSMUSP00000035115 | 98777\_at | ST |
| ENSMUSP00000021508 | 102816\_at | E |
| ENSMUSP00000037431 | 99432\_at | TR |
| ENSMUSP00000054322 | 104116\_at | E |
| ENSMUSP00000027488 | 97331\_at | E |
| ENSMUSP00000046118 | 92341\_at | E |
| ENSMUSP00000034923 | 103592\_at | E |
| ENSMUSP00000020629 | 103737\_at | E |
| ENSMUSP00000020549 | 95363\_at | E |
| ENSMUSP00000021153 | 95496\_at | E |
| ENSMUSP00000021412 | 96952\_at | E |
| ENSMUSP00000060672 | 98385\_at | E |
| ENSMUSP00000050444 | 99841\_at | ST |
| ENSMUSP00000029970 | 104380\_at | T |
| ENSMUSP00000022294 | 160991\_at | E |
| ENSMUSP00000020489 | 104525\_at | E |
| ENSMUSP00000003435 | 96284\_at | E |
| ENSMUSP00000001008 | 102424\_at | ST |
| ENSMUSP00000021195 | 99040\_at | T |
| ENSMUSP00000025110 | 160190\_at | T |
| ENSMUSP00000029769 | 160335\_at | E |
| ENSMUSP00000016498 | 98794\_at | E |
| ENSMUSP00000041912 | 93671\_at | TR |
| ENSMUSP00000022293 | 160529\_r\_at | T |
| ENSMUSP00000018992 | 96693\_at | E |
| ENSMUSP00000046787 | 102700\_at | TR |
| ENSMUSP00000025823 | 96838\_at | E |
| ENSMUSP00000027067 | 93015\_at | E |
| ENSMUSP00000024769 | 98138\_at | TR |
| ENSMUSP00000049315 | 104000\_at | E |
| ENSMUSP00000034063 | 160611\_at | E |
| ENSMUSP00000023007 | 94737\_at | E |
| ENSMUSP00000041008 | 100998\_at | ST |
| ENSMUSP00000040345 | 102298\_at | ST |
| ENSMUSP00000049567 | 101551\_s\_at | T |
| ENSMUSP00000003135 | 99059\_at | TR |
| ENSMUSP00000004829 | 95380\_at | ST |
| ENSMUSP00000028511 | 160076\_at | T |
| ENSMUSP00000037958 | 101786\_at | T |
| ENSMUSP00000039012 | 103086\_at | TR |
| ENSMUSP00000059519 | 93557\_at | E |
| ENSMUSP00000034912 | 96313\_at | ST |
| ENSMUSP00000020145 | 97890\_at | E |
| ENSMUSP00000057312 | 92912\_at | TR |
| ENSMUSP00000011285 | 102574\_at | ST |
| ENSMUSP00000039376 | 92244\_at | E |
| ENSMUSP00000037192 | 98823\_at | T |
| ENSMUSP00000048736 | 103362\_at | ST |
| ENSMUSP00000030801 | 93966\_at | E |
| ENSMUSP00000004850 | 103507\_at | ST |
| ENSMUSP00000031766 | 95133\_at | E |
| ENSMUSP00000041152 | 96855\_at | E |
| ENSMUSP00000058868 | 101406\_at | E |
| ENSMUSP00000047508 | 98022\_at | E |
| ENSMUSP00000007161 | 98288\_at | E |
| ENSMUSP00000029666 | 93298\_at | E |
| ENSMUSP00000029852 | 94754\_at | TR |
| ENSMUSP00000034798 | 97776\_at | ST |
| ENSMUSP00000017316 | 102327\_at | E |
| ENSMUSP00000019058 | 94086\_at | ST |
| ENSMUSP00000032125 | 101815\_at | ST |
| ENSMUSP00000027970 | 98431\_at | E |
| ENSMUSP00000020964 | 103248\_at | E |
| ENSMUSP00000053471 | 93719\_at | ST |
| ENSMUSP00000061149 | 94100\_s\_at | T |
| ENSMUSP00000001713 | 95019\_at | E |
| ENSMUSP00000027975 | 96608\_at | E |
| ENSMUSP00000000193 | 102736\_at | ST |
| ENSMUSP00000034046 | 94507\_at | E |
| ENSMUSP00000028332 | 104036\_at | E |
| ENSMUSP00000034927 | 104169\_at | TR |
| ENSMUSP00000030817 | 160647\_at | E |
| ENSMUSP00000015547 | 98840\_at | E |
| ENSMUSP00000032751 | 93850\_at | ST |
| ENSMUSP00000026159 | 92406\_at | ST |
| ENSMUSP00000021962 | 101423\_at | E |
| ENSMUSP00000012664 | 98317\_at | TR |
| ENSMUSP00000047322 | 104312\_at | E |
| ENSMUSP00000024210 | 99906\_at | TR |
| ENSMUSP00000001507 | 94916\_at | E |
| ENSMUSP00000002007 | 103933\_at | E |
| ENSMUSP00000019060 | 92948\_at | ST |
| ENSMUSP00000056885 | 94115\_at | ST |
| ENSMUSP00000051709 | 160122\_at | E |
| ENSMUSP00000003117 | 94248\_at | T |
| ENSMUSP00000034015 | 160388\_at | E |
| ENSMUSP00000009234 | 95704\_at | T |
| ENSMUSP00000060230 | 97004\_at | ST |
| ENSMUSP00000001383 | 98859\_at | E |
| ENSMUSP00000020710 | 93736\_at | T |
| ENSMUSP00000028024 | 101164\_at | ST |
| ENSMUSP00000058040 | 101309\_at | ST |
| ENSMUSP00000033450 | 102886\_at | ST |
| ENSMUSP00000057748 | 99514\_at | TR |
| ENSMUSP00000027156 | 99647\_at | E |
| ENSMUSP00000032118 | 97679\_at | TR |
| ENSMUSP00000001709 | 95312\_at | TR |
| ENSMUSP00000031355 | 103018\_at | T |
| ENSMUSP00000050576 | 99388\_at | E |
| ENSMUSP00000019906 | 100393\_at | ST |
| ENSMUSP00000023707 | 100538\_at | E |
| ENSMUSP00000006020 | 101181\_at | T |
| ENSMUSP00000024627 | 101459\_at | E |
| ENSMUSP00000054575 | 102915\_at | ST |
| ENSMUSP00000052203 | 93085\_at | E |
| ENSMUSP00000021535 | 160681\_at | E |
| ENSMUSP00000004340 | 99809\_at | TR |
| ENSMUSP00000034624 | 97430\_at | T |
| ENSMUSP00000016336 | 92440\_at | TR |
| ENSMUSP00000028728 | 102247\_at | E |
| ENSMUSP00000018311 | 95607\_at | T |
| ENSMUSP00000034197 | 100279\_at | E |
| ENSMUSP00000006090 | 99940\_at | T |
| ENSMUSP00000037779 | 92982\_at | ST |
| ENSMUSP00000015197 | 102789\_at | TR |
| ENSMUSP00000034000 | 94282\_at | E |
| ENSMUSP00000027440 | 99417\_at | ST |
| ENSMUSP00000018382 | 160567\_at | ST |
| ENSMUSP00000023559 | 97316\_at | E |
| ENSMUSP00000031634 | 93770\_at | E |
| ENSMUSP00000009329 | 92459\_at | ST |
| ENSMUSP00000062716 | 104232\_at | T |
| ENSMUSP00000026507 | 160710\_at | E |
| ENSMUSP00000021549 | 100964\_at | T |
| ENSMUSP00000054026 | 96136\_at | E |
| ENSMUSP00000049063 | 97580\_at | T |
| ENSMUSP00000050917 | 96269\_at | E |
| ENSMUSP00000029468 | 92590\_at | E |
| ENSMUSP00000046326 | 92735\_at | E |
| ENSMUSP00000006525 | 102397\_at | TR |
| ENSMUSP00000047898 | 99025\_at | E |
| ENSMUSP00000026472 | 103986\_at | ST |
| ENSMUSP00000020650 | 94168\_at | ST |
| ENSMUSP00000057944 | 98779\_at | ST |
| ENSMUSP00000022821 | 96678\_at | E |
| ENSMUSP00000026624 | 100572\_at | T |
| ENSMUSP00000018851 | 97333\_at | E |
| ENSMUSP00000021850 | 102017\_at | E |
| ENSMUSP00000032211 | 92476\_at | ST |
| ENSMUSP00000016138 | 98121\_at | E |
| ENSMUSP00000021390 | 93131\_at | E |
| ENSMUSP00000001652 | 98387\_at | ST |
| ENSMUSP00000020846 | 93264\_at | TR |
| ENSMUSP00000051303 | 94720\_at | TR |
| ENSMUSP00000022512 | 160860\_at | ST |
| ENSMUSP00000049909 | 93397\_at | ST |
| ENSMUSP00000028795 | 104527\_at | E |
| ENSMUSP00000025908 | 94185\_at | T |
| ENSMUSP00000002923 | 93540\_at | E |
| ENSMUSP00000046512 | 93673\_at | ST |
| ENSMUSP00000027368 | 96562\_at | T |
| ENSMUSP00000016452 | 96695\_at | E |
| ENSMUSP00000056046 | 96707\_at | TR |
| ENSMUSP00000048587 | 102690\_at | E |
| ENSMUSP00000024575 | 98007\_at | E |
| ENSMUSP00000006507 | 102968\_at | E |
| ENSMUSP00000020886 | 94461\_at | E |
| ENSMUSP00000026009 | 104135\_at | E |
| ENSMUSP00000023515 | 100601\_at | ST |
| ENSMUSP00000023081 | 94539\_f\_at | E |
| ENSMUSP00000038745 | 100066\_at | E |
| ENSMUSP00000010205 | 99860\_at | ST |
| ENSMUSP00000035943 | 99993\_at | E |
| ENSMUSP00000042602 | 93559\_at | E |
| ENSMUSP00000036996 | 104677\_at | E |
| ENSMUSP00000034231 | 102310\_at | ST |
| ENSMUSP00000003876 | 97892\_at | ST |
| ENSMUSP00000032456 | 102576\_at | ST |
| ENSMUSP00000030557 | 94214\_at | T |
| ENSMUSP00000021844 | 97091\_at | E |
| ENSMUSP00000021990 | 101931\_at | ST |
| ENSMUSP00000031106 | 103231\_at | E |
| ENSMUSP00000052248 | 98825\_at | T |
| ENSMUSP00000039580 | 93702\_at | E |
| ENSMUSP00000030434 | 93835\_at | E |
| ENSMUSP00000030808 | 103509\_at | ST |
| ENSMUSP00000021016 | 101396\_at | TR |
| ENSMUSP00000020359 | 101408\_at | E |
| ENSMUSP00000004987 | 102985\_at | E |
| ENSMUSP00000057917 | 99613\_at | E |
| ENSMUSP00000030670 | 104152\_at | E |
| ENSMUSP00000002303 | 96056\_at | E |
| ENSMUSP00000040244 | 100884\_at | E |
| ENSMUSP00000054374 | 92522\_at | T |
| ENSMUSP00000051012 | 92655\_at | E |
| ENSMUSP00000023616 | 103918\_at | T |
| ENSMUSP00000025409 | 160095\_at | E |
| ENSMUSP00000061187 | 160107\_at | E |
| ENSMUSP00000022380 | 93310\_at | E |
| ENSMUSP00000020182 | 104706\_at | T |
| ENSMUSP00000014917 | 92931\_at | ST |
| ENSMUSP00000026666 | 160516\_at | E |
| ENSMUSP00000023958 | 92263\_at | E |
| ENSMUSP00000030765 | 103526\_at | E |
| ENSMUSP00000058349 | 101280\_at | ST |
| ENSMUSP00000029137 | 101425\_at | E |
| ENSMUSP00000042977 | 98041\_at | E |
| ENSMUSP00000022619 | 93051\_at | E |
| ENSMUSP00000007624 | 99896\_at | ST |
| ENSMUSP00000025921 | 99459\_f\_at | E |
| ENSMUSP00000031718 | 99908\_at | TR |
| ENSMUSP00000034441 | 94918\_at | E |
| ENSMUSP00000035498 | 96218\_at | E |
| ENSMUSP00000062272 | 92672\_at | ST |
| ENSMUSP00000006577 | 97807\_at | E |
| ENSMUSP00000025241 | 102346\_at | E |
| ENSMUSP00000037206 | 160257\_at | E |
| ENSMUSP00000017078 | 95694\_at | E |
| ENSMUSP00000025914 | 103001\_at | ST |
| ENSMUSP00000030339 | 98728\_at | ST |
| ENSMUSP00000030398 | 93738\_at | T |
| ENSMUSP00000033509 | 96627\_at | E |
| ENSMUSP00000042321 | 101166\_at | TR |
| ENSMUSP00000038371 | 99371\_at | T |
| ENSMUSP00000002625 | 94381\_at | E |
| ENSMUSP00000034905 | 99649\_at | E |
| ENSMUSP00000033230 | 102192\_r\_at | E |
| ENSMUSP00000029730 | 92425\_at | E |
| ENSMUSP00000043008 | 102087\_at | TR |
| ENSMUSP00000046883 | 95314\_at | E |
| ENSMUSP00000021277 | 98469\_at | E |
| ENSMUSP00000014566 | 104272\_s\_at | E |
| ENSMUSP00000029937 | 104597\_at | E |
| ENSMUSP00000010195 | 102230\_at | E |
| ENSMUSP00000000010 | 103952\_at | TR |
| ENSMUSP00000023734 | 94134\_at | ST |
| ENSMUSP00000025842 | 100407\_at | ST |
| ENSMUSP00000052989 | 103284\_at | E |
| ENSMUSP00000023328 | 93755\_at | ST |
| ENSMUSP00000019117 | 93888\_at | TR |
| ENSMUSP00000019480 | 104495\_f\_at | E |
| ENSMUSP00000027721 | 101183\_at | T |
| ENSMUSP00000055306 | 102772\_at | E |
| ENSMUSP00000043819 | 102917\_at | TR |
| ENSMUSP00000005826 | 99666\_at | E |
| ENSMUSP00000032944 | 104217\_at | E |
| ENSMUSP00000038147 | 100671\_at | ST |
| ENSMUSP00000025767 | 92575\_at | TR |
| ENSMUSP00000022338 | 95331\_at | ST |
| ENSMUSP00000006367 | 96920\_at | E |
| ENSMUSP00000056177 | 100015\_at | E |
| ENSMUSP00000059977 | 95597\_at | E |
| ENSMUSP00000028533 | 101737\_at | ST |
| ENSMUSP00000046595 | 104481\_at | E |
| ENSMUSP00000049161 | 103037\_at | ST |
| ENSMUSP00000054328 | 101898\_s\_at | ST |
| ENSMUSP00000018186 | 94284\_at | E |
| ENSMUSP00000046121 | 95740\_at | E |
| ENSMUSP00000003645 | 100424\_at | E |
| ENSMUSP00000028917 | 97318\_at | E |
| ENSMUSP00000028259 | 103446\_at | E |
| ENSMUSP00000005341 | 93917\_at | ST |
| ENSMUSP00000036384 | 103579\_at | E |
| ENSMUSP00000023210 | 95072\_at | T |
| ENSMUSP00000023760 | 92592\_at | E |
| ENSMUSP00000040310 | 102266\_at | ST |
| ENSMUSP00000021784 | 92737\_at | TR |
| ENSMUSP00000030746 | 94037\_at | E |
| ENSMUSP00000058642 | 100032\_at | TR |
| ENSMUSP00000002740 | 100298\_at | ST |
| ENSMUSP00000033993 | 98515\_at | TR |
| ENSMUSP00000035847 | 104510\_at | T |
| ENSMUSP00000049595 | 93658\_at | E |
| ENSMUSP00000032399 | 97991\_at | E |
| ENSMUSP00000005073 | 102675\_at | ST |
| ENSMUSP00000029679 | 97468\_at | E |
| ENSMUSP00000038935 | 98924\_at | E |
| ENSMUSP00000026414 | 103596\_at | E |
| ENSMUSP00000034775 | 160656\_i\_at | ST |
| ENSMUSP00000060207 | 98123\_at | E |
| ENSMUSP00000033659 | 93133\_at | T |
| ENSMUSP00000005490 | 99845\_at | T |
| ENSMUSP00000060956 | 160862\_at | E |
| ENSMUSP00000002588 | 102283\_at | ST |
| ENSMUSP00000021513 | 94187\_at | TR |
| ENSMUSP00000027802 | 160339\_at | E |
| ENSMUSP00000035164 | 103071\_at | E |
| ENSMUSP00000028063 | 93542\_at | E |
| ENSMUSP00000038838 | 103349\_at | E |
| ENSMUSP00000062764 | 99320\_at | E |
| ENSMUSP00000053945 | 100736\_at | TR |
| ENSMUSP00000049414 | 92362\_at | E |
| ENSMUSP00000024044 | 103480\_at | ST |
| ENSMUSP00000001520 | 103625\_at | E |
| ENSMUSP00000034428 | 96840\_at | T |
| ENSMUSP00000025656 | 100068\_at | E |
| ENSMUSP00000002457 | 101657\_at | ST |
| ENSMUSP00000030948 | 98418\_at | ST |
| ENSMUSP00000020022 | 94872\_at | E |
| ENSMUSP00000059710 | 104679\_at | E |
| ENSMUSP00000007244 | 97894\_at | E |
| ENSMUSP00000032980 | 92916\_at | T |
| ENSMUSP00000020756 | 99194\_at | E |
| ENSMUSP00000013738 | 94216\_at | E |
| ENSMUSP00000030491 | 160356\_at | E |
| ENSMUSP00000027726 | 103922\_f\_at | E |
| ENSMUSP00000028600 | 103233\_at | E |
| ENSMUSP00000057481 | 93704\_at | TR |
| ENSMUSP00000042405 | 101132\_at | T |
| ENSMUSP00000031640 | 96726\_at | E |
| ENSMUSP00000004745 | 101398\_at | T |
| ENSMUSP00000040920 | 104021\_at | TR |
| ENSMUSP00000009631 | 94625\_at | E |
| ENSMUSP00000003191 | 160632\_at | E |
| ENSMUSP00000005336 | 104287\_at | E |
| ENSMUSP00000028004 | 96243\_f\_at | E |
| ENSMUSP00000045888 | 160898\_at | TR |
| ENSMUSP00000052888 | 92524\_at | T |
| ENSMUSP00000018727 | 103642\_at | T |
| ENSMUSP00000043606 | 98302\_at | T |
