# Supplementary material for: Relationship between the tissue-specificity of mouse gene expression and the evolutionary origin and function of the proteins
Source: Genome Biol. 2005 Jun 29;6(7):R56. doi: 10.1186/gb-2005-6-7-r56 (PMC1175987; doi:10.1186/gb-2005-6-7-r56)
Supplement: Additional File 2 — Phyletic assignments of the proteins used in the analysis. Phyletic assignments of the proteins used in the analysis [file gb-2005-6-7-r56-S2.htm]

#### Legend

*U - universal   
E - eukaryota specific   
Z - metazoan
specific   
M - mammalian specific* 


|  |  |  |
| --- | --- | --- |
| **EnsembleProtein** | **Affymetrix\_ID** | **Phylogenetic\_assignment** |
| ENSMUSP00000021726 | 98435\_at | U |
| ENSMUSP00000021148 | 93312\_at | E |
| ENSMUSP00000015998 | 93445\_at | Z |
| ENSMUSP00000023336 | 104430\_at | M |
| ENSMUSP00000023220 | 96201\_at | Z |
| ENSMUSP00000006625 | 100138\_f\_at | E |
| ENSMUSP00000040343 | 97923\_at | E |
| ENSMUSP00000008076 | 94233\_at | Z |
| ENSMUSP00000026857 | 99489\_at | U |
| ENSMUSP00000034313 | 94366\_at | U |
| ENSMUSP00000053446 | 94838\_r\_at | M |
| ENSMUSP00000030914 | 160518\_at | E |
| ENSMUSP00000045973 | 97122\_at | Z |
| ENSMUSP00000037328 | 101950\_at | Z |
| ENSMUSP00000003626 | 161297\_f\_at | M |
| ENSMUSP00000031845 | 103250\_at | M |
| ENSMUSP00000030410 | 93721\_at | E |
| ENSMUSP00000007733 | 98977\_at | M |
| ENSMUSP00000047980 | 92398\_at | Z |
| ENSMUSP00000039493 | 95021\_at | Z |
| ENSMUSP00000054254 | 161698\_f\_at | M |
| ENSMUSP00000040756 | 96610\_at | E |
| ENSMUSP00000021429 | 96743\_at | U |
| ENSMUSP00000020909 | 96876\_at | Z |
| ENSMUSP00000036159 | 101282\_at | Z |
| ENSMUSP00000031903 | 102871\_at | E |
| ENSMUSP00000031968 | 99632\_at | E |
| ENSMUSP00000021734 | 99898\_at | Z |
| ENSMUSP00000028764 | 94775\_at | M |
| ENSMUSP00000001964 | 160927\_at | Z |
| ENSMUSP00000029654 | 104449\_at | Z |
| ENSMUSP00000005234 | 96075\_at | U |
| ENSMUSP00000009777 | 97531\_at | M |
| ENSMUSP00000016771 | 100915\_at | Z |
| ENSMUSP00000029088 | 102070\_at | Z |
| ENSMUSP00000027150 | 92541\_at | E |
| ENSMUSP00000031426 | 102215\_at | Z |
| ENSMUSP00000027264 | 97797\_at | U |
| ENSMUSP00000043427 | 97809\_at | U |
| ENSMUSP00000002126 | 92674\_at | E |
| ENSMUSP00000047379 | 102348\_at | E |
| ENSMUSP00000030198 | 103804\_at | Z |
| ENSMUSP00000029803 | 99097\_at | E |
| ENSMUSP00000060275 | 99109\_at | M |
| ENSMUSP00000055275 | 160126\_at | Z |
| ENSMUSP00000056779 | 160259\_at | M |
| ENSMUSP00000063175 | 95696\_at | U |
| ENSMUSP00000029385 | 95708\_at | E |
| ENSMUSP00000045608 | 101969\_at | M |
| ENSMUSP00000031653 | 98452\_at | Z |
| ENSMUSP00000022848 | 99856\_r\_at | E |
| ENSMUSP00000033184 | 93595\_at | U |
| ENSMUSP00000010804 | 104580\_at | E |
| ENSMUSP00000024956 | 104725\_at | E |
| ENSMUSP00000055881 | 96351\_at | E |
| ENSMUSP00000028617 | 101035\_at | E |
| ENSMUSP00000025826 | 92950\_at | E |
| ENSMUSP00000020546 | 102624\_at | M |
| ENSMUSP00000025955 | 94250\_at | E |
| ENSMUSP00000053307 | 99373\_at | M |
| ENSMUSP00000031674 | 94383\_at | Z |
| ENSMUSP00000053175 | 160390\_at | E |
| ENSMUSP00000032210 | 99518\_at | M |
| ENSMUSP00000031099 | 104057\_at | U |
| ENSMUSP00000023146 | 94528\_at | U |
| ENSMUSP00000029087 | 160668\_at | M |
| ENSMUSP00000029180 | 92282\_at | E |
| ENSMUSP00000031084 | 98994\_at | U |
| ENSMUSP00000048501 | 92427\_at | E |
| ENSMUSP00000028358 | 93871\_at | M |
| ENSMUSP00000020899 | 102089\_at | Z |
| ENSMUSP00000020507 | 95316\_at | Z |
| ENSMUSP00000057513 | 96760\_at | E |
| ENSMUSP00000021087 | 95449\_at | Z |
| ENSMUSP00000032398 | 101444\_at | U |
| ENSMUSP00000054386 | 101577\_at | U |
| ENSMUSP00000062979 | 93070\_at | E |
| ENSMUSP00000036761 | 98338\_at | Z |
| ENSMUSP00000021203 | 93348\_at | E |
| ENSMUSP00000061412 | 99927\_at | Z |
| ENSMUSP00000031621 | 160811\_at | E |
| ENSMUSP00000026566 | 160944\_at | E |
| ENSMUSP00000030886 | 104466\_at | M |
| ENSMUSP00000049002 | 94937\_at | E |
| ENSMUSP00000037618 | 92529\_s\_at | E |
| ENSMUSP00000034158 | 96092\_at | Z |
| ENSMUSP00000025891 | 100932\_at | E |
| ENSMUSP00000019333 | 96104\_at | E |
| ENSMUSP00000051732 | 96237\_at | M |
| ENSMUSP00000025377 | 92691\_at | E |
| ENSMUSP00000041254 | 97826\_at | E |
| ENSMUSP00000017643 | 103821\_at | U |
| ENSMUSP00000032094 | 103954\_at | E |
| ENSMUSP00000027985 | 92969\_at | Z |
| ENSMUSP00000029690 | 95580\_at | Z |
| ENSMUSP00000003470 | 94269\_at | E |
| ENSMUSP00000043682 | 101325\_r\_at | M |
| ENSMUSP00000024005 | 100131\_at | Z |
| ENSMUSP00000050056 | 95725\_at | U |
| ENSMUSP00000032800 | 100397\_at | M |
| ENSMUSP00000034383 | 100409\_at | Z |
| ENSMUSP00000028857 | 98614\_at | E |
| ENSMUSP00000031263 | 93624\_at | U |
| ENSMUSP00000028330 | 103286\_at | E |
| ENSMUSP00000034220 | 95057\_at | Z |
| ENSMUSP00000018547 | 96513\_at | Z |
| ENSMUSP00000050343 | 101185\_at | E |
| ENSMUSP00000002180 | 102641\_at | Z |
| ENSMUSP00000029653 | 102774\_at | Z |
| ENSMUSP00000032886 | 99402\_at | M |
| ENSMUSP00000022570 | 99390\_at | Z |
| ENSMUSP00000018287 | 102919\_at | Z |
| ENSMUSP00000036983 | 98079\_at | U |
| ENSMUSP00000026415 | 94412\_at | E |
| ENSMUSP00000023849 | 99535\_at | E |
| ENSMUSP00000023599 | 93089\_at | U |
| ENSMUSP00000004971 | 160552\_at | E |
| ENSMUSP00000016033 | 100540\_at | U |
| ENSMUSP00000030475 | 97434\_at | U |
| ENSMUSP00000027237 | 95333\_at | Z |
| ENSMUSP00000034285 | 95466\_at | Z |
| ENSMUSP00000042195 | 100017\_at | Z |
| ENSMUSP00000034537 | 95599\_at | E |
| ENSMUSP00000028308 | 93232\_at | M |
| ENSMUSP00000018632 | 98488\_at | E |
| ENSMUSP00000040797 | 99811\_at | Z |
| ENSMUSP00000020777 | 94821\_at | E |
| ENSMUSP00000030572 | 99944\_at | E |
| ENSMUSP00000051579 | 104483\_at | E |
| ENSMUSP00000038012 | 103835\_f\_at | E |
| ENSMUSP00000031072 | 94954\_at | E |
| ENSMUSP00000029694 | 93632\_g\_at | E |
| ENSMUSP00000024892 | 104628\_at | U |
| ENSMUSP00000005620 | 96254\_at | U |
| ENSMUSP00000022467 | 97843\_at | M |
| ENSMUSP00000022391 | 97976\_at | U |
| ENSMUSP00000046727 | 102382\_at | E |
| ENSMUSP00000021141 | 103971\_at | M |
| ENSMUSP00000024047 | 94020\_at | E |
| ENSMUSP00000014117 | 160160\_at | M |
| ENSMUSP00000056359 | 160293\_at | U |
| ENSMUSP00000026560 | 95742\_at | E |
| ENSMUSP00000004003 | 101870\_at | M |
| ENSMUSP00000025292 | 100559\_at | U |
| ENSMUSP00000024796 | 97175\_at | U |
| ENSMUSP00000057085 | 92185\_at | E |
| ENSMUSP00000033038 | 103315\_at | Z |
| ENSMUSP00000031101 | 98909\_at | U |
| ENSMUSP00000021942 | 93774\_at | E |
| ENSMUSP00000061611 | 103448\_at | M |
| ENSMUSP00000033627 | 93919\_at | E |
| ENSMUSP00000003556 | 95074\_at | E |
| ENSMUSP00000048457 | 96663\_at | E |
| ENSMUSP00000015620 | 96808\_at | M |
| ENSMUSP00000025196 | 102791\_at | U |
| ENSMUSP00000034830 | 98108\_at | Z |
| ENSMUSP00000023356 | 99552\_at | Z |
| ENSMUSP00000034466 | 94562\_at | U |
| ENSMUSP00000013880 | 160847\_at | U |
| ENSMUSP00000000219 | 100690\_at | U |
| ENSMUSP00000005334 | 100702\_at | Z |
| ENSMUSP00000024963 | 97451\_at | Z |
| ENSMUSP00000057628 | 100968\_at | E |
| ENSMUSP00000056888 | 102002\_at | E |
| ENSMUSP00000031390 | 92461\_at | E |
| ENSMUSP00000038783 | 97729\_at | E |
| ENSMUSP00000029837 | 92606\_at | E |
| ENSMUSP00000059780 | 92739\_at | M |
| ENSMUSP00000025180 | 95350\_at | U |
| ENSMUSP00000034976 | 99029\_at | E |
| ENSMUSP00000027562 | 100034\_at | U |
| ENSMUSP00000022599 | 95628\_at | E |
| ENSMUSP00000040522 | 101623\_at | U |
| ENSMUSP00000015278 | 98372\_at | U |
| ENSMUSP00000023132 | 93382\_at | E |
| ENSMUSP00000036981 | 103056\_at | U |
| ENSMUSP00000037412 | 94971\_at | M |
| ENSMUSP00000062312 | 104512\_at | M |
| ENSMUSP00000054674 | 104645\_at | Z |
| ENSMUSP00000060817 | 96271\_at | M |
| ENSMUSP00000054694 | 102411\_at | M |
| ENSMUSP00000027469 | 96549\_at | Z |
| ENSMUSP00000033276 | 97993\_at | E |
| ENSMUSP00000028889 | 160555\_r\_at | E |
| ENSMUSP00000025019 | 102677\_at | E |
| ENSMUSP00000029842 | 94448\_at | M |
| ENSMUSP00000033098 | 100443\_at | U |
| ENSMUSP00000005583 | 100576\_at | U |
| ENSMUSP00000033730 | 98781\_at | Z |
| ENSMUSP00000025844 | 92214\_at | E |
| ENSMUSP00000019470 | 93803\_at | Z |
| ENSMUSP00000051187 | 103332\_at | Z |
| ENSMUSP00000038135 | 103598\_at | U |
| ENSMUSP00000022264 | 93936\_at | E |
| ENSMUSP00000010127 | 161110\_at | U |
| ENSMUSP00000032440 | 95091\_at | U |
| ENSMUSP00000034226 | 95103\_at | Z |
| ENSMUSP00000041087 | 96825\_at | E |
| ENSMUSP00000036372 | 102820\_at | E |
| ENSMUSP00000033540 | 101509\_at | E |
| ENSMUSP00000002341 | 102953\_at | M |
| ENSMUSP00000032726 | 98125\_at | Z |
| ENSMUSP00000045683 | 104120\_at | Z |
| ENSMUSP00000045085 | 93268\_at | U |
| ENSMUSP00000034488 | 94724\_at | E |
| ENSMUSP00000013990 | 99847\_at | E |
| ENSMUSP00000055722 | 160864\_at | Z |
| ENSMUSP00000020528 | 94857\_at | U |
| ENSMUSP00000044123 | 100985\_at | E |
| ENSMUSP00000027174 | 92569\_f\_at | U |
| ENSMUSP00000058855 | 92623\_at | Z |
| ENSMUSP00000062412 | 103741\_at | Z |
| ENSMUSP00000020765 | 98155\_r\_at | E |
| ENSMUSP00000053469 | 99179\_at | Z |
| ENSMUSP00000019930 | 95512\_at | U |
| ENSMUSP00000058237 | 95453\_f\_at | M |
| ENSMUSP00000027339 | 160196\_at | E |
| ENSMUSP00000045073 | 160208\_at | E |
| ENSMUSP00000000326 | 94189\_at | M |
| ENSMUSP00000028241 | 100051\_at | U |
| ENSMUSP00000002678 | 101918\_at | Z |
| ENSMUSP00000022533 | 98534\_at | E |
| ENSMUSP00000032268 | 93677\_at | E |
| ENSMUSP00000045150 | 103218\_at | U |
| ENSMUSP00000040035 | 104662\_at | M |
| ENSMUSP00000063038 | 96433\_at | E |
| ENSMUSP00000044356 | 96699\_at | M |
| ENSMUSP00000019291 | 102694\_at | Z |
| ENSMUSP00000063117 | 99322\_at | U |
| ENSMUSP00000037064 | 94835\_f\_at | E |
| ENSMUSP00000034930 | 102839\_at | E |
| ENSMUSP00000005749 | 100460\_at | U |
| ENSMUSP00000008043 | 160617\_at | E |
| ENSMUSP00000009789 | 104139\_at | U |
| ENSMUSP00000027672 | 100593\_at | M |
| ENSMUSP00000030184 | 100605\_at | Z |
| ENSMUSP00000019332 | 96117\_r\_at | E |
| ENSMUSP00000037169 | 97354\_at | Z |
| ENSMUSP00000025234 | 92231\_at | Z |
| ENSMUSP00000057212 | 98810\_at | Z |
| ENSMUSP00000027467 | 97487\_at | U |
| ENSMUSP00000034881 | 93820\_at | M |
| ENSMUSP00000021948 | 92509\_at | Z |
| ENSMUSP00000020586 | 92497\_at | U |
| ENSMUSP00000029603 | 93953\_at | Z |
| ENSMUSP00000020896 | 95120\_at | Z |
| ENSMUSP00000019447 | 102970\_at | E |
| ENSMUSP00000058354 | 101526\_at | Z |
| ENSMUSP00000055886 | 101659\_at | U |
| ENSMUSP00000020118 | 93285\_at | E |
| ENSMUSP00000045118 | 94741\_at | Z |
| ENSMUSP00000025791 | 104270\_at | E |
| ENSMUSP00000010904 | 104548\_at | M |
| ENSMUSP00000031633 | 101639\_r\_at | U |
| ENSMUSP00000016094 | 97763\_at | E |
| ENSMUSP00000006565 | 96319\_at | U |
| ENSMUSP00000034131 | 92640\_at | E |
| ENSMUSP00000018710 | 102314\_at | U |
| ENSMUSP00000021285 | 99063\_at | E |
| ENSMUSP00000051643 | 103903\_at | E |
| ENSMUSP00000033820 | 92918\_at | Z |
| ENSMUSP00000049925 | 99196\_at | M |
| ENSMUSP00000003790 | 94073\_at | U |
| ENSMUSP00000029938 | 160225\_at | U |
| ENSMUSP00000022545 | 100346\_at | Z |
| ENSMUSP00000039239 | 101802\_at | Z |
| ENSMUSP00000056004 | 100479\_at | E |
| ENSMUSP00000062762 | 97107\_at | M |
| ENSMUSP00000006036 | 103090\_at | U |
| ENSMUSP00000032172 | 93561\_at | M |
| ENSMUSP00000031843 | 103235\_at | M |
| ENSMUSP00000041696 | 98829\_at | Z |
| ENSMUSP00000033939 | 93706\_at | U |
| ENSMUSP00000025667 | 93839\_at | E |
| ENSMUSP00000031563 | 95139\_at | E |
| ENSMUSP00000029823 | 101001\_at | Z |
| ENSMUSP00000041456 | 96728\_at | E |
| ENSMUSP00000040089 | 98028\_at | Z |
| ENSMUSP00000034207 | 102989\_at | M |
| ENSMUSP00000054665 | 104023\_at | M |
| ENSMUSP00000045955 | 99617\_at | E |
| ENSMUSP00000003877 | 160501\_at | E |
| ENSMUSP00000044376 | 160634\_at | Z |
| ENSMUSP00000032070 | 94627\_at | Z |
| ENSMUSP00000030818 | 104289\_at | U |
| ENSMUSP00000028025 | 100622\_at | U |
| ENSMUSP00000038925 | 100755\_at | Z |
| ENSMUSP00000004000 | 93583\_s\_at | M |
| ENSMUSP00000003770 | 97516\_at | U |
| ENSMUSP00000041850 | 103511\_at | M |
| ENSMUSP00000033330 | 93970\_at | E |
| ENSMUSP00000019422 | 103644\_at | U |
| ENSMUSP00000007031 | 160099\_at | Z |
| ENSMUSP00000047359 | 102788\_s\_at | Z |
| ENSMUSP00000053420 | 101410\_at | M |
| ENSMUSP00000023897 | 102995\_s\_at | Z |
| ENSMUSP00000062096 | 98304\_at | E |
| ENSMUSP00000025803 | 99881\_at | M |
| ENSMUSP00000001347 | 104432\_at | E |
| ENSMUSP00000021338 | 104565\_at | E |
| ENSMUSP00000019475 | 96191\_at | E |
| ENSMUSP00000034777 | 96203\_at | E |
| ENSMUSP00000028624 | 96336\_at | U |
| ENSMUSP00000062186 | 92790\_at | E |
| ENSMUSP00000033336 | 102331\_at | E |
| ENSMUSP00000018273 | 97925\_at | U |
| ENSMUSP00000006761 | 92935\_at | Z |
| ENSMUSP00000056929 | 94090\_at | M |
| ENSMUSP00000037675 | 160242\_at | E |
| ENSMUSP00000029076 | 160375\_at | U |
| ENSMUSP00000020273 | 94368\_at | U |
| ENSMUSP00000035279 | 100363\_at | E |
| ENSMUSP00000057112 | 100496\_at | Z |
| ENSMUSP00000018313 | 100508\_at | Z |
| ENSMUSP00000023669 | 101952\_at | E |
| ENSMUSP00000044001 | 103252\_at | Z |
| ENSMUSP00000018713 | 99561\_f\_at | M |
| ENSMUSP00000021523 | 98979\_at | E |
| ENSMUSP00000016602 | 93856\_at | Z |
| ENSMUSP00000043668 | 161030\_at | Z |
| ENSMUSP00000044290 | 95023\_at | Z |
| ENSMUSP00000021290 | 101151\_at | E |
| ENSMUSP00000034400 | 96878\_at | E |
| ENSMUSP00000025197 | 102873\_at | U |
| ENSMUSP00000026475 | 101429\_at | M |
| ENSMUSP00000029367 | 94644\_at | U |
| ENSMUSP00000033036 | 93188\_at | M |
| ENSMUSP00000031314 | 94777\_at | M |
| ENSMUSP00000049132 | 104318\_at | U |
| ENSMUSP00000022766 | 160929\_at | E |
| ENSMUSP00000037484 | 97400\_at | E |
| ENSMUSP00000039010 | 100917\_at | U |
| ENSMUSP00000000419 | 102809\_s\_at | Z |
| ENSMUSP00000003911 | 92410\_at | E |
| ENSMUSP00000037146 | 92543\_at | U |
| ENSMUSP00000003313 | 102217\_at | E |
| ENSMUSP00000025856 | 103806\_at | Z |
| ENSMUSP00000045606 | 103939\_at | U |
| ENSMUSP00000028138 | 97983\_s\_at | E |
| ENSMUSP00000035116 | 160128\_at | U |
| ENSMUSP00000041724 | 95565\_at | M |
| ENSMUSP00000037341 | 95698\_at | E |
| ENSMUSP00000055603 | 98321\_at | M |
| ENSMUSP00000040596 | 98454\_at | M |
| ENSMUSP00000021045 | 99910\_at | Z |
| ENSMUSP00000018803 | 94920\_at | U |
| ENSMUSP00000042367 | 93609\_at | E |
| ENSMUSP00000021790 | 96353\_at | E |
| ENSMUSP00000021333 | 161049\_at | E |
| ENSMUSP00000059395 | 94252\_at | U |
| ENSMUSP00000057871 | 160392\_at | E |
| ENSMUSP00000039866 | 160404\_at | M |
| ENSMUSP00000062210 | 100380\_at | E |
| ENSMUSP00000057843 | 100525\_at | U |
| ENSMUSP00000028278 | 97274\_at | E |
| ENSMUSP00000022697 | 98730\_at | Z |
| ENSMUSP00000030389 | 93740\_at | U |
| ENSMUSP00000028979 | 97419\_at | M |
| ENSMUSP00000026858 | 98996\_at | E |
| ENSMUSP00000058691 | 92429\_at | Z |
| ENSMUSP00000036265 | 103414\_at | U |
| ENSMUSP00000028584 | 104302\_f\_at | M |
| ENSMUSP00000013419 | 103547\_at | U |
| ENSMUSP00000035151 | 95318\_at | M |
| ENSMUSP00000002663 | 96895\_at | Z |
| ENSMUSP00000012332 | 96907\_at | E |
| ENSMUSP00000028991 | 102890\_at | Z |
| ENSMUSP00000000305 | 101446\_at | Z |
| ENSMUSP00000027792 | 101579\_at | E |
| ENSMUSP00000056822 | 102902\_at | E |
| ENSMUSP00000001186 | 99651\_at | E |
| ENSMUSP00000034240 | 104335\_at | E |
| ENSMUSP00000025563 | 94794\_at | U |
| ENSMUSP00000022268 | 94806\_at | U |
| ENSMUSP00000028623 | 99929\_at | E |
| ENSMUSP00000031645 | 160946\_at | E |
| ENSMUSP00000035781 | 94939\_at | Z |
| ENSMUSP00000034128 | 96106\_at | U |
| ENSMUSP00000034588 | 96094\_at | E |
| ENSMUSP00000002275 | 96239\_at | E |
| ENSMUSP00000021728 | 97828\_at | M |
| ENSMUSP00000021468 | 92693\_at | U |
| ENSMUSP00000031565 | 92838\_at | Z |
| ENSMUSP00000023465 | 103823\_at | U |
| ENSMUSP00000044785 | 103956\_at | Z |
| ENSMUSP00000023677 | 99128\_at | U |
| ENSMUSP00000055531 | 94005\_at | U |
| ENSMUSP00000056916 | 160145\_at | E |
| ENSMUSP00000056729 | 160278\_at | M |
| ENSMUSP00000053554 | 100133\_at | Z |
| ENSMUSP00000032999 | 101855\_at | M |
| ENSMUSP00000046059 | 93481\_at | E |
| ENSMUSP00000031822 | 93626\_at | U |
| ENSMUSP00000051726 | 103288\_at | M |
| ENSMUSP00000029875 | 104744\_at | Z |
| ENSMUSP00000033015 | 96515\_at | U |
| ENSMUSP00000030436 | 95059\_at | M |
| ENSMUSP00000022295 | 160590\_r\_at | U |
| ENSMUSP00000057836 | 101054\_at | Z |
| ENSMUSP00000057485 | 96648\_at | U |
| ENSMUSP00000021495 | 101187\_at | U |
| ENSMUSP00000014848 | 102643\_at | Z |
| ENSMUSP00000019997 | 99392\_at | Z |
| ENSMUSP00000029905 | 99404\_at | U |
| ENSMUSP00000033516 | 94414\_at | U |
| ENSMUSP00000031478 | 161060\_i\_at | U |
| ENSMUSP00000032165 | 99537\_at | U |
| ENSMUSP00000022960 | 160554\_at | E |
| ENSMUSP00000029947 | 100675\_at | Z |
| ENSMUSP00000038983 | 98880\_at | M |
| ENSMUSP00000046640 | 97436\_at | U |
| ENSMUSP00000045297 | 103564\_at | E |
| ENSMUSP00000028500 | 92579\_at | E |
| ENSMUSP00000020490 | 96924\_at | U |
| ENSMUSP00000003071 | 101463\_at | M |
| ENSMUSP00000020346 | 101608\_at | Z |
| ENSMUSP00000027062 | 93234\_at | Z |
| ENSMUSP00000006444 | 93367\_at | U |
| ENSMUSP00000025805 | 160830\_at | E |
| ENSMUSP00000060056 | 94823\_at | U |
| ENSMUSP00000005120 | 104485\_at | E |
| ENSMUSP00000053619 | 160963\_at | M |
| ENSMUSP00000003625 | 94956\_at | Z |
| ENSMUSP00000039705 | 100951\_at | Z |
| ENSMUSP00000059782 | 96256\_at | U |
| ENSMUSP00000027086 | 97712\_at | U |
| ENSMUSP00000032737 | 102251\_at | Z |
| ENSMUSP00000028281 | 97845\_at | U |
| ENSMUSP00000025862 | 102384\_at | U |
| ENSMUSP00000054024 | 103840\_at | U |
| ENSMUSP00000041538 | 92855\_at | E |
| ENSMUSP00000043981 | 94022\_at | E |
| ENSMUSP00000027991 | 94155\_at | Z |
| ENSMUSP00000027829 | 160162\_at | E |
| ENSMUSP00000014750 | 160295\_at | E |
| ENSMUSP00000045816 | 94288\_at | M |
| ENSMUSP00000026178 | 160307\_at | E |
| ENSMUSP00000034902 | 101872\_at | E |
| ENSMUSP00000027753 | 100428\_at | Z |
| ENSMUSP00000027238 | 98500\_at | Z |
| ENSMUSP00000057631 | 97177\_at | M |
| ENSMUSP00000032183 | 98633\_at | Z |
| ENSMUSP00000019374 | 93643\_at | E |
| ENSMUSP00000022449 | 98766\_at | Z |
| ENSMUSP00000022815 | 93776\_at | E |
| ENSMUSP00000031281 | 104761\_at | M |
| ENSMUSP00000023086 | 95076\_at | Z |
| ENSMUSP00000020272 | 96665\_at | E |
| ENSMUSP00000001708 | 102660\_at | Z |
| ENSMUSP00000056257 | 102793\_at | M |
| ENSMUSP00000022020 | 102938\_at | M |
| ENSMUSP00000019009 | 94564\_at | E |
| ENSMUSP00000030693 | 94697\_at | E |
| ENSMUSP00000055611 | 104238\_at | M |
| ENSMUSP00000045539 | 160716\_at | M |
| ENSMUSP00000023454 | 160849\_at | U |
| ENSMUSP00000004295 | 97320\_at | U |
| ENSMUSP00000014370 | 92596\_at | E |
| ENSMUSP00000029610 | 95485\_at | U |
| ENSMUSP00000034766 | 101889\_s\_at | Z |
| ENSMUSP00000023055 | 101480\_at | E |
| ENSMUSP00000058773 | 101625\_at | U |
| ENSMUSP00000052335 | 101758\_at | M |
| ENSMUSP00000043369 | 98374\_at | M |
| ENSMUSP00000049338 | 93251\_at | Z |
| ENSMUSP00000026262 | 94840\_at | U |
| ENSMUSP00000021406 | 160980\_at | Z |
| ENSMUSP00000043340 | 104514\_at | E |
| ENSMUSP00000034140 | 93529\_at | Z |
| ENSMUSP00000037628 | 94973\_at | E |
| ENSMUSP00000035065 | 104647\_at | E |
| ENSMUSP00000034001 | 96140\_at | M |
| ENSMUSP00000000799 | 97995\_at | E |
| ENSMUSP00000037079 | 102413\_at | E |
| ENSMUSP00000032477 | 92872\_at | E |
| ENSMUSP00000025997 | 99162\_at | E |
| ENSMUSP00000030773 | 94172\_at | U |
| ENSMUSP00000012627 | 160324\_at | M |
| ENSMUSP00000061977 | 160457\_at | M |
| ENSMUSP00000028783 | 97206\_at | Z |
| ENSMUSP00000037337 | 103201\_at | E |
| ENSMUSP00000026999 | 92216\_at | Z |
| ENSMUSP00000036974 | 92349\_at | Z |
| ENSMUSP00000028342 | 93805\_at | M |
| ENSMUSP00000026608 | 103334\_at | E |
| ENSMUSP00000008893 | 98928\_at | U |
| ENSMUSP00000042410 | 93938\_at | U |
| ENSMUSP00000034959 | 161112\_at | Z |
| ENSMUSP00000026986 | 95105\_at | E |
| ENSMUSP00000013773 | 96827\_at | U |
| ENSMUSP00000059295 | 101499\_at | E |
| ENSMUSP00000015587 | 102822\_at | Z |
| ENSMUSP00000015877 | 98127\_at | E |
| ENSMUSP00000020910 | 93137\_at | Z |
| ENSMUSP00000038009 | 104255\_at | E |
| ENSMUSP00000021628 | 160733\_at | U |
| ENSMUSP00000019266 | 104388\_at | M |
| ENSMUSP00000037279 | 94726\_at | M |
| ENSMUSP00000053940 | 97470\_at | M |
| ENSMUSP00000033004 | 102021\_at | M |
| ENSMUSP00000030251 | 103610\_at | E |
| ENSMUSP00000021217 | 92625\_at | U |
| ENSMUSP00000005907 | 102287\_at | M |
| ENSMUSP00000028846 | 92758\_at | E |
| ENSMUSP00000025290 | 101501\_r\_at | E |
| ENSMUSP00000043616 | 101775\_at | E |
| ENSMUSP00000025298 | 98403\_at | U |
| ENSMUSP00000007156 | 98391\_at | Z |
| ENSMUSP00000025271 | 103075\_at | Z |
| ENSMUSP00000010189 | 94990\_at | E |
| ENSMUSP00000006611 | 92540\_f\_at | U |
| ENSMUSP00000009157 | 96298\_f\_at | E |
| ENSMUSP00000049872 | 96435\_at | E |
| ENSMUSP00000032159 | 96568\_at | Z |
| ENSMUSP00000035092 | 102430\_at | Z |
| ENSMUSP00000043533 | 92901\_at | Z |
| ENSMUSP00000041013 | 102708\_at | M |
| ENSMUSP00000023566 | 99324\_at | Z |
| ENSMUSP00000060209 | 99457\_at | Z |
| ENSMUSP00000031613 | 160341\_at | Z |
| ENSMUSP00000062147 | 104008\_at | Z |
| ENSMUSP00000048742 | 160619\_at | E |
| ENSMUSP00000030578 | 100595\_at | E |
| ENSMUSP00000003453 | 100607\_at | Z |
| ENSMUSP00000019302 | 97356\_at | M |
| ENSMUSP00000023698 | 92233\_at | E |
| ENSMUSP00000020060 | 92366\_at | Z |
| ENSMUSP00000033466 | 98812\_at | M |
| ENSMUSP00000035743 | 97489\_at | U |
| ENSMUSP00000025223 | 93225\_s\_at | E |
| ENSMUSP00000031523 | 92499\_at | Z |
| ENSMUSP00000020413 | 93955\_at | M |
| ENSMUSP00000041279 | 96711\_at | U |
| ENSMUSP00000058849 | 98564\_f\_at | E |
| ENSMUSP00000049998 | 95388\_at | M |
| ENSMUSP00000025338 | 98011\_at | U |
| ENSMUSP00000018246 | 93833\_s\_at | M |
| ENSMUSP00000051583 | 93021\_at | M |
| ENSMUSP00000004955 | 160750\_at | U |
| ENSMUSP00000016902 | 93287\_at | M |
| ENSMUSP00000032088 | 99999\_at | M |
| ENSMUSP00000029078 | 92642\_at | U |
| ENSMUSP00000034096 | 103760\_at | E |
| ENSMUSP00000061440 | 92775\_at | E |
| ENSMUSP00000048183 | 102316\_at | E |
| ENSMUSP00000050251 | 103893\_at | M |
| ENSMUSP00000063115 | 99065\_at | M |
| ENSMUSP00000034933 | 103905\_at | U |
| ENSMUSP00000032467 | 99198\_at | U |
| ENSMUSP00000021177 | 95664\_at | E |
| ENSMUSP00000025403 | 98420\_at | U |
| ENSMUSP00000025001 | 97109\_at | Z |
| ENSMUSP00000048705 | 97097\_at | E |
| ENSMUSP00000056033 | 93430\_at | Z |
| ENSMUSP00000046956 | 98553\_at | M |
| ENSMUSP00000049057 | 103092\_at | M |
| ENSMUSP00000025337 | 104681\_at | E |
| ENSMUSP00000023504 | 93696\_at | Z |
| ENSMUSP00000035649 | 161015\_at | E |
| ENSMUSP00000033765 | 96585\_at | Z |
| ENSMUSP00000059592 | 92636\_f\_at | E |
| ENSMUSP00000030047 | 101136\_at | M |
| ENSMUSP00000047480 | 102725\_at | U |
| ENSMUSP00000055548 | 102858\_at | M |
| ENSMUSP00000033959 | 99474\_at | Z |
| ENSMUSP00000054303 | 160491\_at | E |
| ENSMUSP00000020153 | 94484\_at | U |
| ENSMUSP00000005057 | 104025\_at | U |
| ENSMUSP00000062608 | 99619\_at | U |
| ENSMUSP00000057882 | 160503\_at | U |
| ENSMUSP00000021428 | 104158\_at | E |
| ENSMUSP00000018586 | 160769\_at | E |
| ENSMUSP00000028036 | 100757\_at | Z |
| ENSMUSP00000021089 | 97373\_at | E |
| ENSMUSP00000034731 | 98962\_at | Z |
| ENSMUSP00000048684 | 97518\_at | U |
| ENSMUSP00000046097 | 92528\_at | Z |
| ENSMUSP00000032273 | 103513\_at | Z |
| ENSMUSP00000013737 | 93972\_at | U |
| ENSMUSP00000028207 | 103646\_at | E |
| ENSMUSP00000043197 | 103779\_at | Z |
| ENSMUSP00000057905 | 95417\_at | E |
| ENSMUSP00000032874 | 101412\_at | E |
| ENSMUSP00000025419 | 100089\_at | U |
| ENSMUSP00000020824 | 98439\_at | E |
| ENSMUSP00000052417 | 104301\_at | M |
| ENSMUSP00000060668 | 93316\_at | E |
| ENSMUSP00000029862 | 94893\_at | M |
| ENSMUSP00000039519 | 93449\_at | M |
| ENSMUSP00000032841 | 104567\_at | E |
| ENSMUSP00000017188 | 96060\_at | U |
| ENSMUSP00000032570 | 96193\_at | U |
| ENSMUSP00000006745 | 97782\_at | M |
| ENSMUSP00000003858 | 96338\_at | U |
| ENSMUSP00000033023 | 102200\_at | U |
| ENSMUSP00000001063 | 102333\_at | E |
| ENSMUSP00000021405 | 92804\_at | E |
| ENSMUSP00000005452 | 92937\_at | Z |
| ENSMUSP00000001595 | 99082\_at | U |
| ENSMUSP00000052471 | 102599\_at | E |
| ENSMUSP00000062402 | 94104\_at | E |
| ENSMUSP00000038113 | 160377\_at | E |
| ENSMUSP00000025652 | 100365\_at | E |
| ENSMUSP00000040808 | 101821\_at | Z |
| ENSMUSP00000054825 | 101954\_at | E |
| ENSMUSP00000020915 | 93580\_at | E |
| ENSMUSP00000034716 | 97259\_at | E |
| ENSMUSP00000047475 | 103254\_at | E |
| ENSMUSP00000053922 | 93725\_at | Z |
| ENSMUSP00000022682 | 98848\_at | E |
| ENSMUSP00000061725 | 103387\_at | E |
| ENSMUSP00000047646 | 93858\_at | M |
| ENSMUSP00000013683 | 95025\_at | U |
| ENSMUSP00000025163 | 95158\_at | E |
| ENSMUSP00000051307 | 100113\_s\_at | E |
| ENSMUSP00000038915 | 96747\_at | E |
| ENSMUSP00000049532 | 101286\_at | M |
| ENSMUSP00000021063 | 93971\_f\_at | E |
| ENSMUSP00000023691 | 99491\_at | M |
| ENSMUSP00000051838 | 99503\_at | E |
| ENSMUSP00000037834 | 104042\_at | E |
| ENSMUSP00000022163 | 93057\_at | E |
| ENSMUSP00000001127 | 99636\_at | U |
| ENSMUSP00000032555 | 160653\_at | E |
| ENSMUSP00000018700 | 104175\_at | U |
| ENSMUSP00000052860 | 95215\_f\_at | E |
| ENSMUSP00000035052 | 97390\_at | E |
| ENSMUSP00000003569 | 97402\_at | Z |
| ENSMUSP00000061345 | 96079\_at | E |
| ENSMUSP00000019109 | 97535\_at | E |
| ENSMUSP00000022195 | 102074\_at | Z |
| ENSMUSP00000002444 | 102219\_at | E |
| ENSMUSP00000034612 | 92678\_at | U |
| ENSMUSP00000055565 | 103663\_at | E |
| ENSMUSP00000043513 | 101756\_f\_at | M |
| ENSMUSP00000021609 | 103808\_at | E |
| ENSMUSP00000001049 | 95301\_at | M |
| ENSMUSP00000031421 | 95434\_at | E |
| ENSMUSP00000027961 | 101562\_at | U |
| ENSMUSP00000057301 | 98323\_at | Z |
| ENSMUSP00000034723 | 101707\_at | U |
| ENSMUSP00000043198 | 98456\_at | M |
| ENSMUSP00000038781 | 93333\_at | E |
| ENSMUSP00000029566 | 103007\_at | Z |
| ENSMUSP00000000466 | 98589\_at | E |
| ENSMUSP00000033532 | 99912\_at | Z |
| ENSMUSP00000051965 | 93466\_at | E |
| ENSMUSP00000061800 | 93599\_at | M |
| ENSMUSP00000027960 | 160219\_r\_at | M |
| ENSMUSP00000062320 | 104729\_at | M |
| ENSMUSP00000028402 | 96222\_at | E |
| ENSMUSP00000016806 | 97811\_at | E |
| ENSMUSP00000033899 | 101039\_at | Z |
| ENSMUSP00000001884 | 99111\_at | U |
| ENSMUSP00000048152 | 160861\_s\_at | E |
| ENSMUSP00000053849 | 94121\_at | Z |
| ENSMUSP00000023885 | 99377\_at | M |
| ENSMUSP00000020954 | 160394\_at | E |
| ENSMUSP00000029277 | 94387\_at | U |
| ENSMUSP00000015664 | 160406\_at | U |
| ENSMUSP00000009732 | 100382\_at | E |
| ENSMUSP00000022246 | 95976\_at | Z |
| ENSMUSP00000029562 | 97010\_at | Z |
| ENSMUSP00000017332 | 100527\_at | M |
| ENSMUSP00000006254 | 97276\_at | E |
| ENSMUSP00000027696 | 98732\_at | Z |
| ENSMUSP00000015124 | 93742\_at | M |
| ENSMUSP00000022996 | 98865\_at | U |
| ENSMUSP00000040315 | 103416\_at | E |
| ENSMUSP00000005011 | 103549\_at | Z |
| ENSMUSP00000000012 | 160476\_f\_at | E |
| ENSMUSP00000033076 | 101629\_s\_at | E |
| ENSMUSP00000030358 | 95042\_at | E |
| ENSMUSP00000063136 | 101170\_at | Z |
| ENSMUSP00000033157 | 96909\_at | U |
| ENSMUSP00000026900 | 101448\_at | E |
| ENSMUSP00000006462 | 98064\_at | U |
| ENSMUSP00000030768 | 102892\_at | U |
| ENSMUSP00000041560 | 102904\_at | M |
| ENSMUSP00000004910 | 94530\_at | U |
| ENSMUSP00000041475 | 160670\_at | M |
| ENSMUSP00000037858 | 160815\_at | Z |
| ENSMUSP00000022684 | 160948\_at | U |
| ENSMUSP00000015983 | 92562\_at | Z |
| ENSMUSP00000052752 | 92707\_at | M |
| ENSMUSP00000043880 | 92695\_at | M |
| ENSMUSP00000025749 | 103825\_at | E |
| ENSMUSP00000022296 | 92660\_f\_at | E |
| ENSMUSP00000002070 | 95451\_at | Z |
| ENSMUSP00000006697 | 100002\_at | U |
| ENSMUSP00000051967 | 95584\_at | M |
| ENSMUSP00000060724 | 101658\_f\_at | M |
| ENSMUSP00000030782 | 101724\_at | Z |
| ENSMUSP00000030859 | 101857\_at | U |
| ENSMUSP00000021550 | 98473\_at | U |
| ENSMUSP00000026546 | 103024\_at | E |
| ENSMUSP00000054198 | 93483\_at | Z |
| ENSMUSP00000027503 | 98618\_at | U |
| ENSMUSP00000018401 | 93628\_at | M |
| ENSMUSP00000002809 | 104746\_at | U |
| ENSMUSP00000026060 | 96517\_at | Z |
| ENSMUSP00000000590 | 101056\_at | Z |
| ENSMUSP00000032092 | 96009\_s\_at | E |
| ENSMUSP00000057532 | 101189\_at | M |
| ENSMUSP00000040145 | 102645\_at | U |
| ENSMUSP00000051326 | 94271\_at | M |
| ENSMUSP00000034385 | 99394\_at | U |
| ENSMUSP00000053027 | 99406\_at | E |
| ENSMUSP00000029942 | 94416\_at | E |
| ENSMUSP00000036725 | 160423\_at | U |
| ENSMUSP00000056355 | 160556\_at | M |
| ENSMUSP00000033376 | 97293\_at | E |
| ENSMUSP00000000208 | 92315\_at | M |
| ENSMUSP00000023363 | 96781\_at | E |
| ENSMUSP00000045158 | 96926\_at | M |
| ENSMUSP00000016652 | 98081\_at | M |
| ENSMUSP00000027274 | 101465\_at | Z |
| ENSMUSP00000025910 | 99670\_at | M |
| ENSMUSP00000035982 | 93103\_at | U |
| ENSMUSP00000041557 | 104221\_at | U |
| ENSMUSP00000038170 | 99815\_at | Z |
| ENSMUSP00000025523 | 104354\_at | Z |
| ENSMUSP00000060310 | 93369\_at | U |
| ENSMUSP00000034713 | 160832\_at | Z |
| ENSMUSP00000035653 | 94958\_at | M |
| ENSMUSP00000002378 | 96125\_at | Z |
| ENSMUSP00000058021 | 100953\_at | E |
| ENSMUSP00000028005 | 96258\_at | E |
| ENSMUSP00000033470 | 97847\_at | U |
| ENSMUSP00000042444 | 99014\_at | Z |
| ENSMUSP00000030776 | 92857\_at | E |
| ENSMUSP00000049640 | 103975\_at | M |
| ENSMUSP00000006760 | 94024\_at | Z |
| ENSMUSP00000006586 | 160164\_at | E |
| ENSMUSP00000015899 | 160309\_at | M |
| ENSMUSP00000044251 | 92984\_g\_at | E |
| ENSMUSP00000027340 | 100285\_at | U |
| ENSMUSP00000028384 | 98502\_at | E |
| ENSMUSP00000045283 | 97179\_at | M |
| ENSMUSP00000038483 | 98768\_at | Z |
| ENSMUSP00000033805 | 103319\_at | U |
| ENSMUSP00000044004 | 101073\_at | Z |
| ENSMUSP00000046355 | 96667\_at | E |
| ENSMUSP00000018702 | 102662\_at | Z |
| ENSMUSP00000032760 | 102795\_at | Z |
| ENSMUSP00000043077 | 99423\_at | E |
| ENSMUSP00000023099 | 94433\_at | E |
| ENSMUSP00000025393 | 160440\_at | Z |
| ENSMUSP00000030635 | 104095\_at | U |
| ENSMUSP00000021729 | 94566\_at | Z |
| ENSMUSP00000022451 | 160718\_at | E |
| ENSMUSP00000030461 | 94699\_at | E |
| ENSMUSP00000008036 | 100694\_at | E |
| ENSMUSP00000025580 | 97322\_at | M |
| ENSMUSP00000024159 | 92332\_at | E |
| ENSMUSP00000030284 | 160205\_f\_at | E |
| ENSMUSP00000025444 | 103099\_f\_at | E |
| ENSMUSP00000055703 | 96943\_at | E |
| ENSMUSP00000049803 | 101482\_at | U |
| ENSMUSP00000026561 | 160851\_r\_at | M |
| ENSMUSP00000020408 | 98110\_at | Z |
| ENSMUSP00000020362 | 99832\_at | M |
| ENSMUSP00000023214 | 104371\_at | E |
| ENSMUSP00000021197 | 94842\_at | U |
| ENSMUSP00000018920 | 93386\_at | M |
| ENSMUSP00000023119 | 99965\_at | Z |
| ENSMUSP00000039668 | 160982\_at | M |
| ENSMUSP00000041925 | 104516\_at | M |
| ENSMUSP00000001780 | 100970\_at | E |
| ENSMUSP00000058637 | 97731\_at | E |
| ENSMUSP00000033952 | 97997\_at | Z |
| ENSMUSP00000029698 | 99164\_at | Z |
| ENSMUSP00000022031 | 94041\_at | E |
| ENSMUSP00000036487 | 94174\_at | Z |
| ENSMUSP00000033484 | 160181\_at | Z |
| ENSMUSP00000032816 | 95630\_at | U |
| ENSMUSP00000025128 | 94319\_at | E |
| ENSMUSP00000010191 | 100447\_at | Z |
| ENSMUSP00000034304 | 101891\_at | U |
| ENSMUSP00000004657 | 97196\_at | Z |
| ENSMUSP00000018640 | 92218\_at | E |
| ENSMUSP00000036172 | 93807\_at | M |
| ENSMUSP00000036461 | 93795\_at | U |
| ENSMUSP00000036357 | 103469\_at | E |
| ENSMUSP00000032239 | 96551\_at | Z |
| ENSMUSP00000027760 | 92671\_f\_at | E |
| ENSMUSP00000001569 | 95095\_at | U |
| ENSMUSP00000028633 | 101090\_at | Z |
| ENSMUSP00000033570 | 101102\_at | E |
| ENSMUSP00000057161 | 96829\_at | M |
| ENSMUSP00000048635 | 101682\_f\_at | M |
| ENSMUSP00000056621 | 102957\_at | Z |
| ENSMUSP00000020461 | 93006\_at | Z |
| ENSMUSP00000058198 | 93139\_at | U |
| ENSMUSP00000027444 | 160602\_at | Z |
| ENSMUSP00000002064 | 104124\_at | U |
| ENSMUSP00000034361 | 160735\_at | E |
| ENSMUSP00000003502 | 160868\_at | E |
| ENSMUSP00000023040 | 97472\_at | E |
| ENSMUSP00000023752 | 103612\_at | U |
| ENSMUSP00000052119 | 94717\_f\_at | M |
| ENSMUSP00000020912 | 103745\_at | E |
| ENSMUSP00000049772 | 97200\_f\_at | E |
| ENSMUSP00000022196 | 103878\_at | E |
| ENSMUSP00000031268 | 160067\_at | U |
| ENSMUSP00000045127 | 95516\_at | E |
| ENSMUSP00000022340 | 93563\_s\_at | Z |
| ENSMUSP00000023117 | 95649\_at | E |
| ENSMUSP00000006391 | 98405\_at | U |
| ENSMUSP00000047949 | 102380\_s\_at | Z |
| ENSMUSP00000054588 | 93270\_at | U |
| ENSMUSP00000010421 | 98538\_at | E |
| ENSMUSP00000032815 | 99982\_at | U |
| ENSMUSP00000002818 | 104400\_at | E |
| ENSMUSP00000033012 | 94992\_at | E |
| ENSMUSP00000045895 | 104666\_at | Z |
| ENSMUSP00000027059 | 92903\_at | Z |
| ENSMUSP00000024954 | 102698\_at | Z |
| ENSMUSP00000054426 | 94191\_at | Z |
| ENSMUSP00000025992 | 94203\_at | Z |
| ENSMUSP00000030530 | 99326\_at | Z |
| ENSMUSP00000014648 | 160343\_at | E |
| ENSMUSP00000025679 | 94336\_at | E |
| ENSMUSP00000048222 | 94469\_at | U |
| ENSMUSP00000029977 | 100464\_at | Z |
| ENSMUSP00000021359 | 101920\_at | E |
| ENSMUSP00000019672 | 100597\_at | E |
| ENSMUSP00000039342 | 97358\_at | Z |
| ENSMUSP00000050715 | 103220\_at | E |
| ENSMUSP00000002315 | 98814\_at | M |
| ENSMUSP00000027144 | 93824\_at | U |
| ENSMUSP00000047518 | 92368\_at | M |
| ENSMUSP00000028881 | 103486\_at | M |
| ENSMUSP00000000926 | 93957\_at | M |
| ENSMUSP00000025833 | 96713\_at | U |
| ENSMUSP00000028046 | 96846\_at | U |
| ENSMUSP00000035834 | 101385\_at | Z |
| ENSMUSP00000036524 | 98013\_at | E |
| ENSMUSP00000027639 | 102974\_at | Z |
| ENSMUSP00000026673 | 99590\_at | U |
| ENSMUSP00000048101 | 98146\_at | E |
| ENSMUSP00000023110 | 104141\_at | E |
| ENSMUSP00000029711 | 98279\_at | Z |
| ENSMUSP00000028742 | 160752\_at | U |
| ENSMUSP00000026093 | 94878\_at | E |
| ENSMUSP00000051895 | 100740\_at | U |
| ENSMUSP00000049284 | 96045\_at | Z |
| ENSMUSP00000001115 | 102040\_at | E |
| ENSMUSP00000007320 | 97767\_at | E |
| ENSMUSP00000043477 | 102318\_at | E |
| ENSMUSP00000029846 | 92777\_at | Z |
| ENSMUSP00000002733 | 103762\_at | Z |
| ENSMUSP00000038476 | 103895\_at | M |
| ENSMUSP00000033828 | 99067\_at | Z |
| ENSMUSP00000011323 | 160084\_at | U |
| ENSMUSP00000003759 | 100072\_at | U |
| ENSMUSP00000035884 | 95666\_at | E |
| ENSMUSP00000060517 | 101806\_at | Z |
| ENSMUSP00000029029 | 93271\_s\_at | E |
| ENSMUSP00000046251 | 98555\_at | E |
| ENSMUSP00000001092 | 93432\_at | Z |
| ENSMUSP00000022145 | 103094\_at | Z |
| ENSMUSP00000021717 | 93565\_at | U |
| ENSMUSP00000041175 | 104550\_at | E |
| ENSMUSP00000031392 | 97929\_r\_at | E |
| ENSMUSP00000010178 | 93698\_at | E |
| ENSMUSP00000032342 | 93866\_s\_at | M |
| ENSMUSP00000000409 | 96321\_at | U |
| ENSMUSP00000051996 | 93250\_r\_at | E |
| ENSMUSP00000021968 | 92920\_at | Z |
| ENSMUSP00000007797 | 101138\_at | Z |
| ENSMUSP00000057989 | 102727\_at | M |
| ENSMUSP00000055458 | 99343\_at | E |
| ENSMUSP00000023001 | 99476\_at | M |
| ENSMUSP00000026407 | 160493\_at | Z |
| ENSMUSP00000021239 | 94486\_at | U |
| ENSMUSP00000030278 | 160638\_at | U |
| ENSMUSP00000007602 | 92847\_s\_at | E |
| ENSMUSP00000028128 | 100626\_at | U |
| ENSMUSP00000051708 | 97242\_at | M |
| ENSMUSP00000021038 | 100759\_at | E |
| ENSMUSP00000049296 | 97375\_at | U |
| ENSMUSP00000031093 | 92252\_at | Z |
| ENSMUSP00000029387 | 93841\_at | E |
| ENSMUSP00000035227 | 102059\_at | M |
| ENSMUSP00000042174 | 96730\_at | U |
| ENSMUSP00000059748 | 95419\_at | Z |
| ENSMUSP00000033882 | 96996\_at | Z |
| ENSMUSP00000033211 | 98030\_at | E |
| ENSMUSP00000051349 | 93040\_at | M |
| ENSMUSP00000057094 | 93173\_at | U |
| ENSMUSP00000049070 | 98296\_at | E |
| ENSMUSP00000018637 | 98308\_at | E |
| ENSMUSP00000036740 | 93318\_at | Z |
| ENSMUSP00000002798 | 99885\_at | M |
| ENSMUSP00000050836 | 160914\_at | M |
| ENSMUSP00000028360 | 104436\_at | M |
| ENSMUSP00000023666 | 100890\_at | U |
| ENSMUSP00000022813 | 96195\_at | E |
| ENSMUSP00000022172 | 97651\_at | U |
| ENSMUSP00000021268 | 97784\_at | E |
| ENSMUSP00000046103 | 102335\_at | E |
| ENSMUSP00000028972 | 92806\_at | Z |
| ENSMUSP00000033847 | 103924\_at | U |
| ENSMUSP00000037358 | 95550\_at | E |
| ENSMUSP00000031414 | 160113\_at | E |
| ENSMUSP00000061606 | 160246\_at | Z |
| ENSMUSP00000021381 | 94239\_at | E |
| ENSMUSP00000045422 | 100101\_at | E |
| ENSMUSP00000005497 | 97128\_at | M |
| ENSMUSP00000004574 | 98572\_at | U |
| ENSMUSP00000032887 | 93582\_at | U |
| ENSMUSP00000025581 | 103256\_at | M |
| ENSMUSP00000019575 | 93727\_at | U |
| ENSMUSP00000022971 | 104712\_at | E |
| ENSMUSP00000014678 | 100674\_f\_at | M |
| ENSMUSP00000023364 | 161034\_at | Z |
| ENSMUSP00000014133 | 95027\_at | E |
| ENSMUSP00000013806 | 96616\_at | U |
| ENSMUSP00000053295 | 101155\_at | E |
| ENSMUSP00000015581 | 102877\_at | Z |
| ENSMUSP00000022269 | 94370\_at | Z |
| ENSMUSP00000024816 | 98049\_at | E |
| ENSMUSP00000057987 | 93059\_at | M |
| ENSMUSP00000062820 | 94515\_at | U |
| ENSMUSP00000010348 | 104044\_at | U |
| ENSMUSP00000021201 | 160655\_at | U |
| ENSMUSP00000043074 | 100510\_at | M |
| ENSMUSP00000020705 | 160788\_at | E |
| ENSMUSP00000019439 | 97392\_at | Z |
| ENSMUSP00000028056 | 95000\_g\_at | Z |
| ENSMUSP00000019323 | 93991\_at | U |
| ENSMUSP00000004480 | 95436\_at | M |
| ENSMUSP00000043203 | 95569\_at | M |
| ENSMUSP00000026406 | 101431\_at | U |
| ENSMUSP00000022059 | 101709\_at | Z |
| ENSMUSP00000034992 | 93202\_at | U |
| ENSMUSP00000044983 | 99914\_at | Z |
| ENSMUSP00000042124 | 160931\_at | E |
| ENSMUSP00000038784 | 93468\_at | Z |
| ENSMUSP00000004222 | 103009\_at | U |
| ENSMUSP00000020826 | 104453\_at | Z |
| ENSMUSP00000007981 | 94924\_at | M |
| ENSMUSP00000025867 | 97813\_at | Z |
| ENSMUSP00000006660 | 97946\_at | M |
| ENSMUSP00000020828 | 102352\_at | U |
| ENSMUSP00000019517 | 99113\_at | E |
| ENSMUSP00000047483 | 92956\_at | E |
| ENSMUSP00000045177 | 160130\_at | U |
| ENSMUSP00000051379 | 94123\_at | Z |
| ENSMUSP00000015858 | 160396\_at | E |
| ENSMUSP00000034132 | 95712\_at | E |
| ENSMUSP00000028298 | 160408\_at | U |
| ENSMUSP00000048904 | 100384\_at | E |
| ENSMUSP00000059636 | 98326\_f\_at | Z |
| ENSMUSP00000038815 | 101973\_at | M |
| ENSMUSP00000032937 | 93611\_at | Z |
| ENSMUSP00000041636 | 93744\_at | E |
| ENSMUSP00000039734 | 97172\_s\_at | U |
| ENSMUSP00000023598 | 103418\_at | U |
| ENSMUSP00000015948 | 95044\_at | Z |
| ENSMUSP00000051118 | 161051\_at | Z |
| ENSMUSP00000015771 | 96500\_at | E |
| ENSMUSP00000033740 | 101172\_at | M |
| ENSMUSP00000005647 | 96899\_at | U |
| ENSMUSP00000025473 | 102761\_at | U |
| ENSMUSP00000029217 | 97307\_f\_at | Z |
| ENSMUSP00000045025 | 102906\_at | E |
| ENSMUSP00000055806 | 99522\_at | E |
| ENSMUSP00000026818 | 99655\_at | U |
| ENSMUSP00000019354 | 94532\_at | E |
| ENSMUSP00000028475 | 160672\_at | U |
| ENSMUSP00000033553 | 104194\_at | U |
| ENSMUSP00000053672 | 104339\_at | Z |
| ENSMUSP00000038015 | 160817\_at | M |
| ENSMUSP00000009058 | 94798\_at | U |
| ENSMUSP00000030032 | 160472\_r\_at | Z |
| ENSMUSP00000029994 | 97421\_at | U |
| ENSMUSP00000029172 | 100938\_at | M |
| ENSMUSP00000022098 | 96098\_at | U |
| ENSMUSP00000028205 | 97554\_at | E |
| ENSMUSP00000041118 | 92697\_at | E |
| ENSMUSP00000020243 | 102238\_at | Z |
| ENSMUSP00000032207 | 95320\_at | M |
| ENSMUSP00000036277 | 100004\_at | E |
| ENSMUSP00000031429 | 95586\_at | M |
| ENSMUSP00000000364 | 101581\_at | E |
| ENSMUSP00000058180 | 98342\_at | U |
| ENSMUSP00000020015 | 101726\_at | Z |
| ENSMUSP00000030136 | 101859\_at | U |
| ENSMUSP00000022947 | 98475\_at | Z |
| ENSMUSP00000015791 | 99931\_at | Z |
| ENSMUSP00000005233 | 94941\_at | U |
| ENSMUSP00000023934 | 101869\_s\_at | M |
| ENSMUSP00000002061 | 97963\_at | E |
| ENSMUSP00000038540 | 96519\_at | U |
| ENSMUSP00000029608 | 101058\_at | Z |
| ENSMUSP00000005006 | 99130\_at | M |
| ENSMUSP00000007799 | 160280\_at | Z |
| ENSMUSP00000025364 | 94273\_at | E |
| ENSMUSP00000034188 | 99408\_at | U |
| ENSMUSP00000021991 | 160425\_at | E |
| ENSMUSP00000052103 | 160558\_at | E |
| ENSMUSP00000006628 | 100546\_at | E |
| ENSMUSP00000032373 | 101990\_at | U |
| ENSMUSP00000031458 | 100679\_at | M |
| ENSMUSP00000008633 | 92317\_at | E |
| ENSMUSP00000033518 | 103568\_at | Z |
| ENSMUSP00000005830 | 95061\_at | E |
| ENSMUSP00000021913 | 96650\_at | U |
| ENSMUSP00000048672 | 96783\_at | Z |
| ENSMUSP00000050933 | 101334\_at | Z |
| ENSMUSP00000047968 | 101467\_at | M |
| ENSMUSP00000044048 | 93093\_at | M |
| ENSMUSP00000007436 | 99672\_at | U |
| ENSMUSP00000046169 | 160701\_at | Z |
| ENSMUSP00000035976 | 99817\_at | E |
| ENSMUSP00000022135 | 104356\_at | U |
| ENSMUSP00000008528 | 160834\_at | M |
| ENSMUSP00000039823 | 94827\_at | M |
| ENSMUSP00000037324 | 104489\_at | Z |
| ENSMUSP00000027687 | 100955\_at | E |
| ENSMUSP00000005631 | 92581\_at | U |
| ENSMUSP00000022746 | 102255\_at | Z |
| ENSMUSP00000034199 | 103844\_at | E |
| ENSMUSP00000043112 | 99149\_at | E |
| ENSMUSP00000020990 | 94026\_at | M |
| ENSMUSP00000040846 | 95470\_at | U |
| ENSMUSP00000028515 | 100021\_at | Z |
| ENSMUSP00000019917 | 160299\_at | E |
| ENSMUSP00000050818 | 100287\_at | E |
| ENSMUSP00000035009 | 98492\_at | M |
| ENSMUSP00000056814 | 97048\_at | M |
| ENSMUSP00000011308 | 93927\_f\_at | M |
| ENSMUSP00000006009 | 93514\_at | E |
| ENSMUSP00000034164 | 96669\_at | E |
| ENSMUSP00000032696 | 92990\_at | M |
| ENSMUSP00000052768 | 102664\_at | Z |
| ENSMUSP00000030333 | 102797\_at | U |
| ENSMUSP00000049764 | 94290\_at | M |
| ENSMUSP00000053750 | 94302\_at | E |
| ENSMUSP00000022176 | 99425\_at | U |
| ENSMUSP00000023830 | 94435\_at | U |
| ENSMUSP00000050966 | 99558\_at | E |
| ENSMUSP00000037385 | 100430\_at | M |
| ENSMUSP00000035506 | 104109\_at | U |
| ENSMUSP00000021642 | 94568\_at | Z |
| ENSMUSP00000025468 | 100696\_at | E |
| ENSMUSP00000060218 | 97324\_at | U |
| ENSMUSP00000036346 | 103452\_at | U |
| ENSMUSP00000003066 | 95356\_at | M |
| ENSMUSP00000001812 | 96812\_at | Z |
| ENSMUSP00000058187 | 101351\_at | Z |
| ENSMUSP00000028743 | 96945\_at | E |
| ENSMUSP00000045129 | 101484\_at | E |
| ENSMUSP00000025262 | 102940\_at | M |
| ENSMUSP00000026498 | 93122\_at | E |
| ENSMUSP00000004097 | 104240\_at | E |
| ENSMUSP00000036437 | 99834\_at | M |
| ENSMUSP00000031771 | 103809\_r\_at | E |
| ENSMUSP00000024104 | 94711\_at | Z |
| ENSMUSP00000029402 | 160201\_r\_at | U |
| ENSMUSP00000061081 | 104518\_at | M |
| ENSMUSP00000021810 | 96144\_at | Z |
| ENSMUSP00000025227 | 92610\_at | E |
| ENSMUSP00000004173 | 100587\_f\_at | Z |
| ENSMUSP00000031693 | 92743\_at | Z |
| ENSMUSP00000037466 | 102272\_at | M |
| ENSMUSP00000022286 | 92876\_at | U |
| ENSMUSP00000046027 | 99033\_at | E |
| ENSMUSP00000056804 | 103994\_at | E |
| ENSMUSP00000055971 | 99166\_at | M |
| ENSMUSP00000019231 | 94043\_at | Z |
| ENSMUSP00000051896 | 94176\_at | E |
| ENSMUSP00000041814 | 160328\_at | U |
| ENSMUSP00000001284 | 93656\_g\_at | E |
| ENSMUSP00000057950 | 101760\_at | Z |
| ENSMUSP00000041579 | 103060\_at | Z |
| ENSMUSP00000030010 | 97198\_at | U |
| ENSMUSP00000028258 | 93531\_at | E |
| ENSMUSP00000060996 | 98787\_at | Z |
| ENSMUSP00000001801 | 103205\_at | U |
| ENSMUSP00000047353 | 93664\_at | Z |
| ENSMUSP00000063065 | 103338\_at | M |
| ENSMUSP00000000709 | 93809\_at | Z |
| ENSMUSP00000062577 | 95109\_at | M |
| ENSMUSP00000021479 | 95097\_at | E |
| ENSMUSP00000046143 | 96553\_at | Z |
| ENSMUSP00000023615 | 101104\_at | E |
| ENSMUSP00000030952 | 102681\_at | M |
| ENSMUSP00000007449 | 102826\_at | U |
| ENSMUSP00000057527 | 102959\_at | U |
| ENSMUSP00000043783 | 99442\_at | M |
| ENSMUSP00000034311 | 93008\_at | E |
| ENSMUSP00000052196 | 160604\_at | E |
| ENSMUSP00000062502 | 104126\_at | U |
| ENSMUSP00000009719 | 104259\_at | E |
| ENSMUSP00000046856 | 160737\_at | U |
| ENSMUSP00000033489 | 97341\_at | Z |
| ENSMUSP00000015645 | 92484\_at | Z |
| ENSMUSP00000023840 | 102025\_at | M |
| ENSMUSP00000003667 | 98930\_at | E |
| ENSMUSP00000031773 | 93940\_at | Z |
| ENSMUSP00000062156 | 103614\_at | U |
| ENSMUSP00000029456 | 95373\_at | M |
| ENSMUSP00000006898 | 160069\_at | M |
| ENSMUSP00000050566 | 95518\_at | M |
| ENSMUSP00000022590 | 97204\_s\_at | E |
| ENSMUSP00000033006 | 100057\_at | E |
| ENSMUSP00000023390 | 101779\_at | Z |
| ENSMUSP00000003568 | 98395\_at | Z |
| ENSMUSP00000027401 | 93272\_at | U |
| ENSMUSP00000033563 | 98407\_at | Z |
| ENSMUSP00000008542 | 99984\_at | Z |
| ENSMUSP00000035105 | 97750\_at | U |
| ENSMUSP00000038562 | 102301\_at | E |
| ENSMUSP00000033505 | 92760\_at | E |
| ENSMUSP00000029964 | 92905\_at | Z |
| ENSMUSP00000001872 | 102567\_at | Z |
| ENSMUSP00000020319 | 99183\_at | E |
| ENSMUSP00000001125 | 94060\_at | E |
| ENSMUSP00000059127 | 94193\_at | M |
| ENSMUSP00000001551 | 99328\_at | Z |
| ENSMUSP00000009503 | 94205\_at | E |
| ENSMUSP00000044497 | 160345\_at | U |
| ENSMUSP00000043892 | 100599\_at | M |
| ENSMUSP00000001908 | 101922\_at | E |
| ENSMUSP00000000153 | 97227\_at | E |
| ENSMUSP00000061136 | 160178\_r\_at | U |
| ENSMUSP00000034834 | 96066\_s\_at | U |
| ENSMUSP00000015987 | 92237\_at | Z |
| ENSMUSP00000052182 | 93681\_at | Z |
| ENSMUSP00000027939 | 93826\_at | E |
| ENSMUSP00000050399 | 103488\_at | E |
| ENSMUSP00000014290 | 102710\_at | Z |
| ENSMUSP00000031383 | 101254\_at | E |
| ENSMUSP00000028292 | 96848\_at | U |
| ENSMUSP00000029684 | 98015\_at | E |
| ENSMUSP00000017290 | 102976\_at | E |
| ENSMUSP00000031632 | 104010\_at | M |
| ENSMUSP00000050312 | 99604\_at | Z |
| ENSMUSP00000036161 | 93158\_at | Z |
| ENSMUSP00000035034 | 160621\_at | Z |
| ENSMUSP00000018816 | 104143\_at | E |
| ENSMUSP00000047564 | 160754\_at | U |
| ENSMUSP00000030202 | 104276\_at | E |
| ENSMUSP00000025951 | 96047\_at | M |
| ENSMUSP00000030425 | 97491\_at | E |
| ENSMUSP00000009356 | 92978\_s\_at | U |
| ENSMUSP00000015427 | 102042\_at | Z |
| ENSMUSP00000029670 | 97769\_at | Z |
| ENSMUSP00000051848 | 92646\_at | E |
| ENSMUSP00000060114 | 103631\_at | Z |
| ENSMUSP00000035486 | 103909\_at | U |
| ENSMUSP00000054719 | 95390\_at | Z |
| ENSMUSP00000018544 | 94079\_at | E |
| ENSMUSP00000021967 | 100074\_at | E |
| ENSMUSP00000021306 | 101530\_at | U |
| ENSMUSP00000000804 | 101542\_f\_at | U |
| ENSMUSP00000061551 | 101808\_at | M |
| ENSMUSP00000048119 | 98424\_at | Z |
| ENSMUSP00000046786 | 93301\_at | Z |
| ENSMUSP00000000755 | 93434\_at | E |
| ENSMUSP00000029381 | 93567\_at | M |
| ENSMUSP00000029177 | 97520\_s\_at | M |
| ENSMUSP00000053412 | 99478\_at | M |
| ENSMUSP00000019400 | 160495\_at | Z |
| ENSMUSP00000002259 | 104029\_at | E |
| ENSMUSP00000020827 | 160507\_at | E |
| ENSMUSP00000062953 | 94488\_at | E |
| ENSMUSP00000026302 | 94289\_r\_at | E |
| ENSMUSP00000023689 | 100483\_at | M |
| ENSMUSP00000047588 | 97377\_at | E |
| ENSMUSP00000027004 | 92254\_at | E |
| ENSMUSP00000052996 | 93710\_at | M |
| ENSMUSP00000034497 | 98833\_at | E |
| ENSMUSP00000003017 | 92387\_at | U |
| ENSMUSP00000015270 | 103372\_at | U |
| ENSMUSP00000002403 | 93843\_at | U |
| ENSMUSP00000000349 | 98966\_at | U |
| ENSMUSP00000046364 | 93976\_at | E |
| ENSMUSP00000058361 | 95010\_at | E |
| ENSMUSP00000059245 | 93498\_s\_at | Z |
| ENSMUSP00000030145 | 96732\_at | Z |
| ENSMUSP00000056705 | 96998\_at | U |
| ENSMUSP00000053631 | 98032\_at | M |
| ENSMUSP00000041250 | 102860\_at | U |
| ENSMUSP00000060087 | 92805\_s\_at | E |
| ENSMUSP00000048924 | 102993\_at | M |
| ENSMUSP00000037039 | 93042\_at | U |
| ENSMUSP00000039921 | 93439\_f\_at | M |
| ENSMUSP00000059655 | 104160\_at | U |
| ENSMUSP00000023441 | 94631\_at | M |
| ENSMUSP00000053161 | 94764\_at | Z |
| ENSMUSP00000047083 | 160916\_at | U |
| ENSMUSP00000040281 | 94909\_at | E |
| ENSMUSP00000032089 | 96064\_at | E |
| ENSMUSP00000028768 | 100892\_at | E |
| ENSMUSP00000026172 | 97786\_at | U |
| ENSMUSP00000056213 | 102204\_at | Z |
| ENSMUSP00000030551 | 92796\_at | U |
| ENSMUSP00000033075 | 103781\_at | E |
| ENSMUSP00000008619 | 103926\_at | M |
| ENSMUSP00000045434 | 95552\_at | M |
| ENSMUSP00000021243 | 100091\_at | E |
| ENSMUSP00000043315 | 95685\_at | E |
| ENSMUSP00000028154 | 100369\_at | E |
| ENSMUSP00000000857 | 98441\_at | Z |
| ENSMUSP00000035607 | 101825\_at | U |
| ENSMUSP00000018449 | 98574\_at | E |
| ENSMUSP00000028362 | 103258\_at | E |
| ENSMUSP00000026643 | 104714\_at | E |
| ENSMUSP00000020173 | 93729\_at | M |
| ENSMUSP00000034326 | 95029\_at | U |
| ENSMUSP00000020051 | 161036\_at | U |
| ENSMUSP00000022438 | 100678\_s\_at | Z |
| ENSMUSP00000025319 | 96618\_at | E |
| ENSMUSP00000050372 | 102746\_at | M |
| ENSMUSP00000059985 | 94517\_r\_at | M |
| ENSMUSP00000059983 | 94372\_at | U |
| ENSMUSP00000034146 | 99507\_at | E |
| ENSMUSP00000034301 | 160524\_at | E |
| ENSMUSP00000002898 | 160657\_at | Z |
| ENSMUSP00000045174 | 95961\_at | E |
| ENSMUSP00000018333 | 100512\_at | E |
| ENSMUSP00000030118 | 97261\_at | U |
| ENSMUSP00000053657 | 93519\_s\_at | E |
| ENSMUSP00000030964 | 100778\_at | M |
| ENSMUSP00000039112 | 92271\_at | Z |
| ENSMUSP00000000199 | 92416\_at | E |
| ENSMUSP00000019050 | 98983\_at | U |
| ENSMUSP00000031524 | 103401\_at | U |
| ENSMUSP00000032715 | 97539\_at | U |
| ENSMUSP00000055095 | 102078\_at | M |
| ENSMUSP00000021940 | 93993\_at | E |
| ENSMUSP00000036562 | 92549\_at | M |
| ENSMUSP00000016427 | 101300\_at | M |
| ENSMUSP00000028592 | 96882\_at | E |
| ENSMUSP00000057669 | 95438\_at | E |
| ENSMUSP00000062790 | 101699\_at | M |
| ENSMUSP00000010051 | 93337\_at | U |
| ENSMUSP00000039518 | 104322\_at | M |
| ENSMUSP00000021527 | 99916\_at | E |
| ENSMUSP00000046539 | 104455\_at | E |
| ENSMUSP00000047356 | 160933\_at | M |
| ENSMUSP00000026661 | 96081\_at | U |
| ENSMUSP00000017320 | 96226\_at | E |
| ENSMUSP00000043047 | 96359\_at | E |
| ENSMUSP00000009728 | 102221\_at | Z |
| ENSMUSP00000022701 | 97948\_at | E |
| ENSMUSP00000051685 | 102354\_at | M |
| ENSMUSP00000024724 | 92825\_at | E |
| ENSMUSP00000038117 | 103810\_at | Z |
| ENSMUSP00000030473 | 99115\_at | Z |
| ENSMUSP00000025062 | 160132\_at | M |
| ENSMUSP00000034600 | 94125\_at | M |
| ENSMUSP00000061596 | 160265\_at | U |
| ENSMUSP00000005532 | 100120\_at | Z |
| ENSMUSP00000043662 | 95714\_at | E |
| ENSMUSP00000032344 | 94258\_at | E |
| ENSMUSP00000021799 | 160398\_at | M |
| ENSMUSP00000039589 | 101975\_at | Z |
| ENSMUSP00000034973 | 93613\_at | Z |
| ENSMUSP00000044838 | 103275\_at | U |
| ENSMUSP00000017836 | 96490\_at | U |
| ENSMUSP00000026756 | 96502\_at | U |
| ENSMUSP00000019859 | 96635\_at | E |
| ENSMUSP00000036505 | 96768\_at | U |
| ENSMUSP00000042402 | 99378\_f\_at | M |
| ENSMUSP00000000708 | 102896\_at | Z |
| ENSMUSP00000023987 | 102908\_at | Z |
| ENSMUSP00000023248 | 93078\_at | M |
| ENSMUSP00000020849 | 104063\_at | E |
| ENSMUSP00000034818 | 94534\_at | U |
| ENSMUSP00000030630 | 104196\_at | Z |
| ENSMUSP00000031729 | 160674\_at | E |
| ENSMUSP00000036162 | 104208\_at | E |
| ENSMUSP00000036226 | 160819\_at | E |
| ENSMUSP00000002099 | 97423\_at | Z |
| ENSMUSP00000000291 | 92300\_at | Z |
| ENSMUSP00000028102 | 92433\_at | E |
| ENSMUSP00000029771 | 97689\_at | M |
| ENSMUSP00000056666 | 103551\_at | M |
| ENSMUSP00000032703 | 92699\_at | U |
| ENSMUSP00000030658 | 103684\_at | U |
| ENSMUSP00000063119 | 103829\_at | E |
| ENSMUSP00000031726 | 96911\_at | U |
| ENSMUSP00000014743 | 101450\_at | M |
| ENSMUSP00000022852 | 95588\_at | U |
| ENSMUSP00000040342 | 100139\_at | M |
| ENSMUSP00000048012 | 101583\_at | Z |
| ENSMUSP00000060003 | 101728\_at | Z |
| ENSMUSP00000033767 | 99800\_at | Z |
| ENSMUSP00000045571 | 93354\_at | M |
| ENSMUSP00000009223 | 94810\_at | U |
| ENSMUSP00000003067 | 99933\_at | Z |
| ENSMUSP00000020664 | 103028\_at | Z |
| ENSMUSP00000049394 | 96110\_at | U |
| ENSMUSP00000002964 | 97832\_at | E |
| ENSMUSP00000023580 | 93087\_r\_at | E |
| ENSMUSP00000044234 | 97965\_at | U |
| ENSMUSP00000023779 | 102371\_at | Z |
| ENSMUSP00000001110 | 92975\_at | Z |
| ENSMUSP00000006750 | 103960\_at | Z |
| ENSMUSP00000037762 | 94142\_at | M |
| ENSMUSP00000030439 | 160282\_at | Z |
| ENSMUSP00000030446 | 94275\_at | U |
| ENSMUSP00000043034 | 95731\_at | Z |
| ENSMUSP00000013842 | 100548\_at | M |
| ENSMUSP00000018430 | 101992\_at | U |
| ENSMUSP00000037569 | 103437\_at | M |
| ENSMUSP00000030104 | 93896\_at | E |
| ENSMUSP00000019121 | 95063\_at | E |
| ENSMUSP00000060675 | 99872\_s\_at | U |
| ENSMUSP00000056617 | 96652\_at | E |
| ENSMUSP00000021209 | 101191\_at | E |
| ENSMUSP00000046196 | 102780\_at | E |
| ENSMUSP00000019701 | 102925\_at | E |
| ENSMUSP00000012587 | 99541\_at | E |
| ENSMUSP00000059373 | 99674\_at | E |
| ENSMUSP00000027502 | 94551\_at | E |
| ENSMUSP00000054548 | 160703\_at | E |
| ENSMUSP00000019611 | 104358\_at | E |
| ENSMUSP00000036871 | 160969\_at | M |
| ENSMUSP00000027076 | 94829\_at | Z |
| ENSMUSP00000031971 | 100957\_at | U |
| ENSMUSP00000001036 | 97573\_at | M |
| ENSMUSP00000034370 | 92450\_at | U |
| ENSMUSP00000042095 | 92583\_at | U |
| ENSMUSP00000027164 | 97718\_at | M |
| ENSMUSP00000006887 | 102257\_at | E |
| ENSMUSP00000028467 | 92728\_at | Z |
| ENSMUSP00000043583 | 99018\_at | M |
| ENSMUSP00000015283 | 103846\_at | Z |
| ENSMUSP00000028603 | 160168\_at | U |
| ENSMUSP00000018005 | 100023\_at | E |
| ENSMUSP00000057479 | 95617\_at | E |
| ENSMUSP00000005545 | 100156\_at | U |
| ENSMUSP00000029673 | 100289\_at | M |
| ENSMUSP00000041467 | 160547\_s\_at | E |
| ENSMUSP00000030179 | 101878\_at | M |
| ENSMUSP00000026096 | 93371\_at | Z |
| ENSMUSP00000048246 | 99950\_at | U |
| ENSMUSP00000020078 | 104634\_at | E |
| ENSMUSP00000001460 | 96260\_at | E |
| ENSMUSP00000052095 | 97982\_at | M |
| ENSMUSP00000006749 | 101077\_at | E |
| ENSMUSP00000004770 | 102666\_at | U |
| ENSMUSP00000027657 | 102799\_at | Z |
| ENSMUSP00000032349 | 94292\_at | U |
| ENSMUSP00000001878 | 99427\_at | Z |
| ENSMUSP00000024553 | 160444\_at | M |
| ENSMUSP00000029060 | 160577\_at | U |
| ENSMUSP00000057231 | 100698\_at | Z |
| ENSMUSP00000050907 | 92191\_at | U |
| ENSMUSP00000031170 | 98770\_at | M |
| ENSMUSP00000034848 | 97459\_at | U |
| ENSMUSP00000006900 | 93780\_at | U |
| ENSMUSP00000050388 | 98915\_at | E |
| ENSMUSP00000002883 | 92469\_at | Z |
| ENSMUSP00000004473 | 103454\_at | Z |
| ENSMUSP00000004729 | 96947\_at | U |
| ENSMUSP00000049773 | 101353\_at | Z |
| ENSMUSP00000014271 | 99580\_s\_at | U |
| ENSMUSP00000018619 | 99120\_f\_at | U |
| ENSMUSP00000034369 | 101486\_at | U |
| ENSMUSP00000025279 | 98114\_at | E |
| ENSMUSP00000035422 | 93257\_at | U |
| ENSMUSP00000006907 | 99836\_at | U |
| ENSMUSP00000026568 | 160853\_at | E |
| ENSMUSP00000036482 | 94979\_at | M |
| ENSMUSP00000057753 | 96146\_at | Z |
| ENSMUSP00000008031 | 100974\_at | E |
| ENSMUSP00000036320 | 97735\_at | Z |
| ENSMUSP00000032539 | 92612\_at | U |
| ENSMUSP00000025192 | 102274\_at | M |
| ENSMUSP00000004171 | 97868\_at | U |
| ENSMUSP00000041229 | 103863\_at | E |
| ENSMUSP00000005755 | 99035\_at | U |
| ENSMUSP00000030041 | 94045\_at | Z |
| ENSMUSP00000029005 | 95501\_at | E |
| ENSMUSP00000033170 | 100040\_at | U |
| ENSMUSP00000016637 | 94178\_at | Z |
| ENSMUSP00000056287 | 101762\_at | M |
| ENSMUSP00000059074 | 100318\_at | U |
| ENSMUSP00000033139 | 103475\_s\_at | U |
| ENSMUSP00000030905 | 93533\_at | E |
| ENSMUSP00000029302 | 103062\_at | E |
| ENSMUSP00000030237 | 98789\_at | M |
| ENSMUSP00000034995 | 104651\_at | E |
| ENSMUSP00000006856 | 103207\_at | U |
| ENSMUSP00000029507 | 96688\_at | Z |
| ENSMUSP00000054881 | 101094\_at | Z |
| ENSMUSP00000031037 | 102683\_at | U |
| ENSMUSP00000001271 | 99444\_at | M |
| ENSMUSP00000000356 | 94454\_at | M |
| ENSMUSP00000020129 | 99577\_at | M |
| ENSMUSP00000048569 | 160594\_at | Z |
| ENSMUSP00000038765 | 92219\_s\_at | E |
| ENSMUSP00000061997 | 104128\_at | E |
| ENSMUSP00000047231 | 160739\_at | E |
| ENSMUSP00000031029 | 100582\_at | E |
| ENSMUSP00000028800 | 97210\_at | Z |
| ENSMUSP00000031740 | 97343\_at | E |
| ENSMUSP00000059869 | 100727\_at | E |
| ENSMUSP00000039762 | 92353\_at | Z |
| ENSMUSP00000017732 | 93800\_f\_at | E |
| ENSMUSP00000018754 | 92486\_at | U |
| ENSMUSP00000027271 | 93942\_at | U |
| ENSMUSP00000038800 | 103616\_at | M |
| ENSMUSP00000032934 | 160090\_f\_at | U |
| ENSMUSP00000004054 | 101370\_at | E |
| ENSMUSP00000060686 | 100059\_at | M |
| ENSMUSP00000029850 | 98131\_at | U |
| ENSMUSP00000021129 | 101515\_at | U |
| ENSMUSP00000058575 | 101648\_at | E |
| ENSMUSP00000026036 | 93141\_at | Z |
| ENSMUSP00000027195 | 93274\_at | E |
| ENSMUSP00000027866 | 98397\_at | E |
| ENSMUSP00000018561 | 98409\_at | E |
| ENSMUSP00000020984 | 160870\_at | U |
| ENSMUSP00000032403 | 93419\_at | Z |
| ENSMUSP00000041450 | 104404\_at | U |
| ENSMUSP00000059122 | 104537\_at | U |
| ENSMUSP00000049177 | 96030\_at | M |
| ENSMUSP00000047559 | 96296\_at | U |
| ENSMUSP00000033477 | 102291\_at | Z |
| ENSMUSP00000060006 | 97885\_at | M |
| ENSMUSP00000032242 | 92762\_at | Z |
| ENSMUSP00000022140 | 92907\_at | M |
| ENSMUSP00000020071 | 92895\_at | Z |
| ENSMUSP00000025081 | 99052\_at | Z |
| ENSMUSP00000057880 | 102569\_at | Z |
| ENSMUSP00000024909 | 94062\_at | U |
| ENSMUSP00000043561 | 99185\_at | E |
| ENSMUSP00000001331 | 95651\_at | U |
| ENSMUSP00000031814 | 160214\_at | E |
| ENSMUSP00000008834 | 160347\_at | E |
| ENSMUSP00000006742 | 100335\_at | U |
| ENSMUSP00000057489 | 95929\_at | Z |
| ENSMUSP00000021034 | 101924\_at | U |
| ENSMUSP00000062364 | 97229\_at | E |
| ENSMUSP00000020403 | 93550\_at | E |
| ENSMUSP00000003501 | 92239\_at | E |
| ENSMUSP00000025300 | 98818\_at | Z |
| ENSMUSP00000035255 | 93683\_at | E |
| ENSMUSP00000033430 | 103224\_at | E |
| ENSMUSP00000029240 | 103357\_at | U |
| ENSMUSP00000028341 | 95128\_at | E |
| ENSMUSP00000022704 | 101123\_at | Z |
| ENSMUSP00000032326 | 96717\_at | U |
| ENSMUSP00000006956 | 102712\_at | M |
| ENSMUSP00000031377 | 101389\_at | Z |
| ENSMUSP00000036128 | 98017\_at | U |
| ENSMUSP00000023531 | 99461\_at | E |
| ENSMUSP00000032485 | 99594\_at | Z |
| ENSMUSP00000063080 | 99606\_at | E |
| ENSMUSP00000031350 | 160623\_at | E |
| ENSMUSP00000004565 | 160756\_at | E |
| ENSMUSP00000031554 | 160889\_at | M |
| ENSMUSP00000034343 | 97360\_at | M |
| ENSMUSP00000004474 | 97505\_at | E |
| ENSMUSP00000033741 | 96049\_at | Z |
| ENSMUSP00000005255 | 102044\_at | Z |
| ENSMUSP00000044879 | 92515\_at | E |
| ENSMUSP00000034368 | 160744\_r\_at | Z |
| ENSMUSP00000029481 | 92648\_at | E |
| ENSMUSP00000029729 | 160088\_at | U |
| ENSMUSP00000021789 | 95392\_at | E |
| ENSMUSP00000004920 | 95537\_at | E |
| ENSMUSP00000015618 | 98426\_at | E |
| ENSMUSP00000005394 | 93303\_at | E |
| ENSMUSP00000020718 | 98559\_at | E |
| ENSMUSP00000028010 | 104421\_at | U |
| ENSMUSP00000002466 | 104554\_at | Z |
| ENSMUSP00000050412 | 104687\_at | Z |
| ENSMUSP00000057371 | 96325\_at | Z |
| ENSMUSP00000025217 | 97914\_at | U |
| ENSMUSP00000023952 | 101009\_at | Z |
| ENSMUSP00000015583 | 92924\_at | Z |
| ENSMUSP00000048171 | 102586\_at | Z |
| ENSMUSP00000003906 | 160231\_at | U |
| ENSMUSP00000015605 | 92395\_r\_at | E |
| ENSMUSP00000010249 | 160497\_at | M |
| ENSMUSP00000011178 | 94357\_at | U |
| ENSMUSP00000023889 | 160509\_at | U |
| ENSMUSP00000020926 | 92986\_g\_at | M |
| ENSMUSP00000000834 | 97113\_at | M |
| ENSMUSP00000022013 | 103241\_at | U |
| ENSMUSP00000021907 | 97379\_at | U |
| ENSMUSP00000012104 | 93712\_at | E |
| ENSMUSP00000030714 | 98835\_at | Z |
| ENSMUSP00000044971 | 98968\_at | E |
| ENSMUSP00000030795 | 93845\_at | U |
| ENSMUSP00000037555 | 103519\_at | U |
| ENSMUSP00000056310 | 93978\_at | E |
| ENSMUSP00000022811 | 95012\_at | U |
| ENSMUSP00000022235 | 101140\_at | Z |
| ENSMUSP00000038559 | 96867\_at | M |
| ENSMUSP00000036240 | 160640\_at | U |
| ENSMUSP00000038134 | 160773\_at | E |
| ENSMUSP00000051085 | 99889\_at | Z |
| ENSMUSP00000042457 | 94766\_at | U |
| ENSMUSP00000045120 | 160918\_at | M |
| ENSMUSP00000005553 | 94899\_at | Z |
| ENSMUSP00000024881 | 100761\_at | U |
| ENSMUSP00000030872 | 100894\_at | E |
| ENSMUSP00000001327 | 100906\_at | E |
| ENSMUSP00000057182 | 96199\_at | E |
| ENSMUSP00000007737 | 93418\_g\_at | E |
| ENSMUSP00000020323 | 92532\_at | Z |
| ENSMUSP00000041477 | 102194\_at | E |
| ENSMUSP00000037273 | 102206\_at | U |
| ENSMUSP00000061317 | 103650\_at | E |
| ENSMUSP00000057578 | 94098\_at | M |
| ENSMUSP00000053496 | 160117\_at | Z |
| ENSMUSP00000058662 | 100093\_at | E |
| ENSMUSP00000059715 | 98310\_at | E |
| ENSMUSP00000051627 | 101827\_at | M |
| ENSMUSP00000025835 | 93320\_at | E |
| ENSMUSP00000003772 | 93453\_at | Z |
| ENSMUSP00000026649 | 93586\_at | Z |
| ENSMUSP00000025027 | 95114\_s\_at | U |
| ENSMUSP00000020156 | 92644\_s\_at | E |
| ENSMUSP00000015000 | 96342\_at | E |
| ENSMUSP00000026839 | 161038\_at | U |
| ENSMUSP00000044459 | 101159\_at | E |
| ENSMUSP00000027869 | 102748\_at | Z |
| ENSMUSP00000001806 | 94241\_at | U |
| ENSMUSP00000033171 | 99497\_at | Z |
| ENSMUSP00000033506 | 94374\_at | U |
| ENSMUSP00000031226 | 93692\_f\_at | M |
| ENSMUSP00000010899 | 104048\_at | U |
| ENSMUSP00000000304 | 160659\_at | U |
| ENSMUSP00000049716 | 95963\_at | M |
| ENSMUSP00000020930 | 100514\_at | E |
| ENSMUSP00000027302 | 92273\_at | E |
| ENSMUSP00000057630 | 98852\_at | E |
| ENSMUSP00000031354 | 92418\_at | U |
| ENSMUSP00000033241 | 103403\_at | U |
| ENSMUSP00000041675 | 103391\_at | U |
| ENSMUSP00000027281 | 103536\_at | Z |
| ENSMUSP00000009875 | 101302\_at | U |
| ENSMUSP00000015956 | 101290\_at | M |
| ENSMUSP00000008537 | 96884\_at | U |
| ENSMUSP00000026558 | 98051\_at | Z |
| ENSMUSP00000053508 | 101435\_at | M |
| ENSMUSP00000033875 | 101568\_at | U |
| ENSMUSP00000025827 | 99640\_at | E |
| ENSMUSP00000004827 | 93194\_at | Z |
| ENSMUSP00000027669 | 98329\_at | U |
| ENSMUSP00000004203 | 160802\_at | E |
| ENSMUSP00000050088 | 160935\_at | E |
| ENSMUSP00000022882 | 100923\_at | E |
| ENSMUSP00000034874 | 96228\_at | U |
| ENSMUSP00000039332 | 94791\_s\_at | Z |
| ENSMUSP00000039360 | 102223\_at | M |
| ENSMUSP00000033423 | 92682\_at | Z |
| ENSMUSP00000059534 | 97817\_at | Z |
| ENSMUSP00000022825 | 102356\_at | U |
| ENSMUSP00000029932 | 103812\_at | M |
| ENSMUSP00000021944 | 92827\_at | U |
| ENSMUSP00000027298 | 103945\_at | Z |
| ENSMUSP00000036951 | 160147\_r\_at | M |
| ENSMUSP00000005952 | 95571\_at | U |
| ENSMUSP00000027727 | 160134\_at | U |
| ENSMUSP00000060892 | 160267\_at | E |
| ENSMUSP00000034714 | 100122\_at | U |
| ENSMUSP00000051057 | 101711\_at | M |
| ENSMUSP00000017597 | 101844\_at | U |
| ENSMUSP00000059061 | 101977\_at | U |
| ENSMUSP00000028055 | 93470\_at | U |
| ENSMUSP00000045044 | 103011\_at | E |
| ENSMUSP00000032419 | 98593\_at | U |
| ENSMUSP00000025396 | 97149\_at | Z |
| ENSMUSP00000031137 | 104600\_at | E |
| ENSMUSP00000053779 | 93748\_at | M |
| ENSMUSP00000033713 | 104733\_at | E |
| ENSMUSP00000028355 | 96504\_at | Z |
| ENSMUSP00000031071 | 96637\_at | E |
| ENSMUSP00000028283 | 101176\_at | M |
| ENSMUSP00000059159 | 102632\_at | E |
| ENSMUSP00000027446 | 102765\_at | E |
| ENSMUSP00000030683 | 102898\_at | Z |
| ENSMUSP00000024721 | 102762\_r\_at | U |
| ENSMUSP00000008605 | 99381\_at | Z |
| ENSMUSP00000028900 | 160410\_at | E |
| ENSMUSP00000031094 | 94403\_at | E |
| ENSMUSP00000032195 | 104065\_at | E |
| ENSMUSP00000019939 | 160543\_at | E |
| ENSMUSP00000042057 | 104198\_at | M |
| ENSMUSP00000005062 | 104428\_s\_at | Z |
| ENSMUSP00000043215 | 92290\_at | E |
| ENSMUSP00000044866 | 92302\_at | E |
| ENSMUSP00000002029 | 103420\_at | M |
| ENSMUSP00000027769 | 92568\_at | Z |
| ENSMUSP00000052683 | 102109\_at | E |
| ENSMUSP00000011152 | 103686\_at | Z |
| ENSMUSP00000032444 | 95324\_at | U |
| ENSMUSP00000006539 | 101585\_at | E |
| ENSMUSP00000053424 | 93223\_at | M |
| ENSMUSP00000000505 | 93356\_at | U |
| ENSMUSP00000058728 | 99802\_at | Z |
| ENSMUSP00000006774 | 94812\_at | E |
| ENSMUSP00000056700 | 104341\_at | Z |
| ENSMUSP00000055209 | 94945\_at | M |
| ENSMUSP00000003352 | 104619\_at | U |
| ENSMUSP00000034866 | 96112\_at | U |
| ENSMUSP00000004507 | 96245\_at | U |
| ENSMUSP00000031059 | 102240\_at | Z |
| ENSMUSP00000024995 | 92711\_at | Z |
| ENSMUSP00000057764 | 98524\_f\_at | E |
| ENSMUSP00000026922 | 160695\_i\_at | Z |
| ENSMUSP00000053934 | 99134\_at | Z |
| ENSMUSP00000040005 | 94011\_at | E |
| ENSMUSP00000013338 | 95600\_at | E |
| ENSMUSP00000035533 | 94277\_at | E |
| ENSMUSP00000024737 | 95733\_at | E |
| ENSMUSP00000038329 | 160429\_at | E |
| ENSMUSP00000004750 | 101861\_at | Z |
| ENSMUSP00000034378 | 100417\_at | U |
| ENSMUSP00000020859 | 97166\_at | U |
| ENSMUSP00000041975 | 104750\_at | M |
| ENSMUSP00000041016 | 103306\_at | U |
| ENSMUSP00000031042 | 93898\_at | Z |
| ENSMUSP00000057970 | 103439\_at | M |
| ENSMUSP00000012540 | 161072\_at | Z |
| ENSMUSP00000029147 | 95065\_at | U |
| ENSMUSP00000029894 | 96521\_at | U |
| ENSMUSP00000028683 | 101060\_at | U |
| ENSMUSP00000032551 | 101193\_at | M |
| ENSMUSP00000059116 | 96787\_at | U |
| ENSMUSP00000021866 | 102782\_at | U |
| ENSMUSP00000033981 | 99410\_at | U |
| ENSMUSP00000020316 | 98087\_at | E |
| ENSMUSP00000020161 | 93097\_at | U |
| ENSMUSP00000024227 | 104082\_at | E |
| ENSMUSP00000013970 | 160693\_at | E |
| ENSMUSP00000051079 | 104227\_at | Z |
| ENSMUSP00000051789 | 160705\_at | Z |
| ENSMUSP00000059492 | 99623\_s\_at | M |
| ENSMUSP00000027198 | 160838\_at | E |
| ENSMUSP00000049529 | 97442\_at | U |
| ENSMUSP00000005066 | 92585\_at | E |
| ENSMUSP00000059928 | 103570\_at | M |
| ENSMUSP00000060843 | 102126\_at | E |
| ENSMUSP00000037649 | 103715\_at | E |
| ENSMUSP00000000394 | 95341\_at | Z |
| ENSMUSP00000025684 | 96930\_at | E |
| ENSMUSP00000032080 | 95619\_at | E |
| ENSMUSP00000056786 | 98363\_at | M |
| ENSMUSP00000003964 | 98496\_at | E |
| ENSMUSP00000001802 | 93373\_at | E |
| ENSMUSP00000027713 | 99952\_at | U |
| ENSMUSP00000059415 | 103047\_at | E |
| ENSMUSP00000019317 | 96262\_at | E |
| ENSMUSP00000031386 | 102402\_at | Z |
| ENSMUSP00000010241 | 101079\_at | E |
| ENSMUSP00000026891 | 99151\_at | U |
| ENSMUSP00000023726 | 92994\_at | Z |
| ENSMUSP00000059719 | 102668\_at | Z |
| ENSMUSP00000001706 | 92891\_f\_at | Z |
| ENSMUSP00000050458 | 94161\_at | E |
| ENSMUSP00000044951 | 95750\_at | M |
| ENSMUSP00000034742 | 94294\_at | E |
| ENSMUSP00000039133 | 160313\_at | E |
| ENSMUSP00000057244 | 99429\_at | M |
| ENSMUSP00000028987 | 160446\_at | M |
| ENSMUSP00000021680 | 100301\_at | Z |
| ENSMUSP00000026865 | 95883\_at | E |
| ENSMUSP00000039632 | 94439\_at | E |
| ENSMUSP00000038514 | 92205\_at | Z |
| ENSMUSP00000032557 | 97328\_at | E |
| ENSMUSP00000031318 | 98772\_at | M |
| ENSMUSP00000045067 | 98917\_at | U |
| ENSMUSP00000030992 | 93782\_at | E |
| ENSMUSP00000007280 | 103589\_at | E |
| ENSMUSP00000030482 | 103353\_f\_at | U |
| ENSMUSP00000027607 | 94743\_f\_at | Z |
| ENSMUSP00000009392 | 101355\_at | Z |
| ENSMUSP00000036946 | 102811\_at | E |
| ENSMUSP00000033185 | 98116\_at | Z |
| ENSMUSP00000001304 | 93126\_at | U |
| ENSMUSP00000034865 | 94715\_at | E |
| ENSMUSP00000021757 | 99838\_at | M |
| ENSMUSP00000056245 | 160855\_at | M |
| ENSMUSP00000034832 | 100976\_at | E |
| ENSMUSP00000047220 | 96148\_at | E |
| ENSMUSP00000008016 | 92614\_at | Z |
| ENSMUSP00000028922 | 92747\_at | Z |
| ENSMUSP00000046625 | 103732\_at | E |
| ENSMUSP00000042551 | 103998\_at | E |
| ENSMUSP00000001454 | 94047\_at | M |
| ENSMUSP00000047451 | 95503\_at | M |
| ENSMUSP00000030805 | 95491\_at | U |
| ENSMUSP00000024974 | 100042\_at | U |
| ENSMUSP00000042164 | 101764\_at | Z |
| ENSMUSP00000051105 | 103064\_at | E |
| ENSMUSP00000041857 | 104653\_at | E |
| ENSMUSP00000001927 | 103209\_at | Z |
| ENSMUSP00000061397 | 99347\_f\_at | U |
| ENSMUSP00000030443 | 96557\_at | E |
| ENSMUSP00000030458 | 101108\_at | E |
| ENSMUSP00000059587 | 102552\_at | M |
| ENSMUSP00000031214 | 102685\_at | M |
| ENSMUSP00000025576 | 99446\_at | M |
| ENSMUSP00000061999 | 94323\_at | E |
| ENSMUSP00000034983 | 99579\_at | Z |
| ENSMUSP00000019067 | 160596\_at | Z |
| ENSMUSP00000023215 | 100451\_at | E |
| ENSMUSP00000001187 | 100584\_at | E |
| ENSMUSP00000060035 | 100729\_at | U |
| ENSMUSP00000030627 | 103340\_at | U |
| ENSMUSP00000022038 | 97478\_at | E |
| ENSMUSP00000022917 | 92355\_at | Z |
| ENSMUSP00000032851 | 98934\_at | E |
| ENSMUSP00000027009 | 92488\_at | Z |
| ENSMUSP00000001792 | 102029\_at | Z |
| ENSMUSP00000055288 | 98573\_r\_at | E |
| ENSMUSP00000039794 | 95244\_at | M |
| ENSMUSP00000046002 | 97381\_s\_at | E |
| ENSMUSP00000035753 | 93077\_s\_at | M |
| ENSMUSP00000027694 | 96833\_at | M |
| ENSMUSP00000022053 | 101372\_at | U |
| ENSMUSP00000023520 | 98000\_at | E |
| ENSMUSP00000029876 | 98133\_at | E |
| ENSMUSP00000014892 | 101517\_at | E |
| ENSMUSP00000023590 | 102961\_at | E |
| ENSMUSP00000033497 | 93010\_at | E |
| ENSMUSP00000060254 | 93143\_at | M |
| ENSMUSP00000021083 | 93276\_at | M |
| ENSMUSP00000044998 | 94732\_at | Z |
| ENSMUSP00000017344 | 99855\_at | E |
| ENSMUSP00000034455 | 104394\_at | U |
| ENSMUSP00000047054 | 99988\_at | E |
| ENSMUSP00000040319 | 94865\_at | E |
| ENSMUSP00000054679 | 104406\_at | Z |
| ENSMUSP00000031199 | 104539\_at | U |
| ENSMUSP00000046974 | 102293\_at | Z |
| ENSMUSP00000027682 | 102305\_at | Z |
| ENSMUSP00000003074 | 97887\_at | M |
| ENSMUSP00000026210 | 103882\_at | E |
| ENSMUSP00000060749 | 99054\_at | E |
| ENSMUSP00000034620 | 92897\_at | M |
| ENSMUSP00000004913 | 92909\_at | Z |
| ENSMUSP00000025600 | 94064\_at | E |
| ENSMUSP00000025714 | 160071\_at | E |
| ENSMUSP00000016309 | 95520\_at | M |
| ENSMUSP00000030365 | 95653\_at | Z |
| ENSMUSP00000025546 | 160349\_at | U |
| ENSMUSP00000023906 | 95786\_at | E |
| ENSMUSP00000033495 | 101926\_at | E |
| ENSMUSP00000028045 | 103226\_at | Z |
| ENSMUSP00000058725 | 99807\_r\_at | M |
| ENSMUSP00000022019 | 96574\_at | M |
| ENSMUSP00000060098 | 98809\_s\_at | E |
| ENSMUSP00000007248 | 102714\_at | U |
| ENSMUSP00000033046 | 98019\_at | E |
| ENSMUSP00000031741 | 99463\_at | U |
| ENSMUSP00000025037 | 160480\_at | E |
| ENSMUSP00000056502 | 93029\_at | U |
| ENSMUSP00000006787 | 104014\_at | M |
| ENSMUSP00000009435 | 94473\_at | M |
| ENSMUSP00000030018 | 104147\_at | U |
| ENSMUSP00000025421 | 160758\_at | U |
| ENSMUSP00000059910 | 94618\_at | E |
| ENSMUSP00000019572 | 100613\_at | U |
| ENSMUSP00000006626 | 100879\_at | E |
| ENSMUSP00000035579 | 97507\_at | Z |
| ENSMUSP00000057840 | 92517\_at | E |
| ENSMUSP00000030438 | 102046\_at | E |
| ENSMUSP00000033005 | 103490\_at | Z |
| ENSMUSP00000006101 | 93961\_at | Z |
| ENSMUSP00000022727 | 103635\_at | U |
| ENSMUSP00000061442 | 103768\_at | Z |
| ENSMUSP00000046504 | 96850\_at | E |
| ENSMUSP00000020375 | 95539\_at | Z |
| ENSMUSP00000034585 | 100078\_at | M |
| ENSMUSP00000053078 | 101534\_at | M |
| ENSMUSP00000062760 | 98150\_at | E |
| ENSMUSP00000024869 | 98428\_at | U |
| ENSMUSP00000021674 | 160901\_at | Z |
| ENSMUSP00000062344 | 104689\_at | M |
| ENSMUSP00000031103 | 102322\_at | U |
| ENSMUSP00000062882 | 92781\_at | Z |
| ENSMUSP00000028816 | 97916\_at | E |
| ENSMUSP00000062621 | 99071\_at | M |
| ENSMUSP00000032191 | 103911\_at | U |
| ENSMUSP00000006556 | 92926\_at | M |
| ENSMUSP00000041831 | 94081\_at | E |
| ENSMUSP00000027061 | 99349\_at | M |
| ENSMUSP00000044654 | 94226\_at | Z |
| ENSMUSP00000040450 | 160366\_at | Z |
| ENSMUSP00000035375 | 94359\_at | E |
| ENSMUSP00000020238 | 160499\_at | U |
| ENSMUSP00000040477 | 101810\_at | Z |
| ENSMUSP00000027634 | 97248\_at | E |
| ENSMUSP00000029920 | 160984\_r\_at | M |
| ENSMUSP00000023331 | 92258\_at | M |
| ENSMUSP00000023148 | 103243\_at | M |
| ENSMUSP00000033640 | 161021\_at | E |
| ENSMUSP00000030858 | 95014\_at | Z |
| ENSMUSP00000034264 | 95147\_at | U |
| ENSMUSP00000022906 | 101142\_at | Z |
| ENSMUSP00000018711 | 96869\_at | E |
| ENSMUSP00000048648 | 102864\_at | Z |
| ENSMUSP00000061901 | 94490\_at | E |
| ENSMUSP00000021921 | 104031\_at | E |
| ENSMUSP00000022089 | 94502\_at | E |
| ENSMUSP00000032122 | 94635\_at | Z |
| ENSMUSP00000020284 | 104164\_at | U |
| ENSMUSP00000049248 | 160775\_at | E |
| ENSMUSP00000054602 | 104297\_at | E |
| ENSMUSP00000021065 | 100896\_at | M |
| ENSMUSP00000056284 | 101959\_r\_at | E |
| ENSMUSP00000059921 | 100908\_at | E |
| ENSMUSP00000053649 | 96068\_at | U |
| ENSMUSP00000020645 | 92401\_at | M |
| ENSMUSP00000029868 | 92534\_at | E |
| ENSMUSP00000061942 | 102063\_at | E |
| ENSMUSP00000043190 | 102196\_at | E |
| ENSMUSP00000023429 | 102208\_at | E |
| ENSMUSP00000036442 | 98398\_s\_at | M |
| ENSMUSP00000062599 | 95423\_at | U |
| ENSMUSP00000024042 | 160119\_at | Z |
| ENSMUSP00000024809 | 95689\_at | E |
| ENSMUSP00000035795 | 100095\_at | Z |
| ENSMUSP00000030687 | 98312\_at | Z |
| ENSMUSP00000042632 | 101829\_at | Z |
| ENSMUSP00000035992 | 98445\_at | E |
| ENSMUSP00000058321 | 99901\_at | M |
| ENSMUSP00000036132 | 98578\_at | M |
| ENSMUSP00000018698 | 104440\_at | U |
| ENSMUSP00000058825 | 104573\_at | U |
| ENSMUSP00000034249 | 93588\_at | E |
| ENSMUSP00000018433 | 96344\_at | U |
| ENSMUSP00000030800 | 97800\_at | Z |
| ENSMUSP00000051843 | 97933\_at | E |
| ENSMUSP00000036604 | 92810\_at | U |
| ENSMUSP00000046263 | 94243\_at | E |
| ENSMUSP00000054462 | 99366\_at | E |
| ENSMUSP00000006915 | 99499\_at | U |
| ENSMUSP00000025828 | 100516\_at | E |
| ENSMUSP00000001834 | 101960\_at | U |
| ENSMUSP00000021210 | 160485\_r\_at | E |
| ENSMUSP00000063070 | 97265\_at | M |
| ENSMUSP00000051962 | 97398\_at | U |
| ENSMUSP00000030391 | 92275\_at | Z |
| ENSMUSP00000057886 | 98854\_at | Z |
| ENSMUSP00000031795 | 93731\_at | U |
| ENSMUSP00000022507 | 103393\_at | E |
| ENSMUSP00000007444 | 103405\_at | E |
| ENSMUSP00000018748 | 103538\_at | Z |
| ENSMUSP00000050561 | 93997\_at | M |
| ENSMUSP00000058162 | 96620\_at | M |
| ENSMUSP00000000964 | 95297\_at | Z |
| ENSMUSP00000057937 | 96753\_at | Z |
| ENSMUSP00000046199 | 96886\_at | Z |
| ENSMUSP00000054058 | 92677\_s\_at | Z |
| ENSMUSP00000005406 | 93063\_at | Z |
| ENSMUSP00000020190 | 104181\_at | U |
| ENSMUSP00000031929 | 93208\_at | Z |
| ENSMUSP00000001044 | 160792\_at | Z |
| ENSMUSP00000026266 | 104326\_at | Z |
| ENSMUSP00000033198 | 160937\_at | U |
| ENSMUSP00000033683 | 100780\_at | U |
| ENSMUSP00000034903 | 96085\_at | E |
| ENSMUSP00000005787 | 92551\_at | U |
| ENSMUSP00000026050 | 97819\_at | U |
| ENSMUSP00000040885 | 95440\_at | Z |
| ENSMUSP00000052188 | 160269\_at | E |
| ENSMUSP00000057802 | 95573\_at | E |
| ENSMUSP00000029297 | 101713\_at | U |
| ENSMUSP00000001818 | 160751\_i\_at | E |
| ENSMUSP00000021903 | 101979\_at | Z |
| ENSMUSP00000057569 | 103013\_at | E |
| ENSMUSP00000033769 | 98595\_at | E |
| ENSMUSP00000058250 | 93617\_at | Z |
| ENSMUSP00000005839 | 103279\_at | Z |
| ENSMUSP00000023509 | 96494\_at | E |
| ENSMUSP00000026289 | 101045\_at | U |
| ENSMUSP00000036853 | 92960\_at | M |
| ENSMUSP00000030568 | 101178\_at | Z |
| ENSMUSP00000014915 | 160412\_at | E |
| ENSMUSP00000040488 | 160545\_at | E |
| ENSMUSP00000000776 | 104067\_at | E |
| ENSMUSP00000031678 | 160678\_at | Z |
| ENSMUSP00000022962 | 100400\_at | U |
| ENSMUSP00000027054 | 97695\_s\_at | U |
| ENSMUSP00000047402 | 97235\_f\_at | M |
| ENSMUSP00000060060 | 97282\_at | M |
| ENSMUSP00000025797 | 97427\_at | Z |
| ENSMUSP00000026383 | 98871\_at | M |
| ENSMUSP00000029717 | 103422\_at | M |
| ENSMUSP00000036206 | 103555\_at | E |
| ENSMUSP00000063051 | 95326\_at | E |
| ENSMUSP00000057257 | 96770\_at | M |
| ENSMUSP00000041204 | 102910\_at | U |
| ENSMUSP00000047551 | 101587\_at | U |
| ENSMUSP00000003775 | 93080\_at | E |
| ENSMUSP00000055852 | 104210\_at | M |
| ENSMUSP00000059705 | 99804\_at | Z |
| ENSMUSP00000043587 | 99937\_at | Z |
| ENSMUSP00000000001 | 94814\_at | E |
| ENSMUSP00000009538 | 160954\_at | Z |
| ENSMUSP00000029170 | 104476\_at | E |
| ENSMUSP00000023133 | 96114\_at | M |
| ENSMUSP00000030802 | 102242\_at | Z |
| ENSMUSP00000030938 | 99961\_s\_at | E |
| ENSMUSP00000018094 | 92713\_at | Z |
| ENSMUSP00000052856 | 97836\_at | E |
| ENSMUSP00000026699 | 103831\_at | E |
| ENSMUSP00000017868 | 92979\_at | Z |
| ENSMUSP00000025906 | 103964\_at | Z |
| ENSMUSP00000019074 | 94146\_at | M |
| ENSMUSP00000014339 | 160153\_at | U |
| ENSMUSP00000051952 | 104474\_s\_at | Z |
| ENSMUSP00000037574 | 95602\_at | M |
| ENSMUSP00000021807 | 160286\_at | E |
| ENSMUSP00000015320 | 95735\_at | M |
| ENSMUSP00000037113 | 101730\_at | Z |
| ENSMUSP00000060079 | 101996\_at | E |
| ENSMUSP00000029331 | 97168\_at | Z |
| ENSMUSP00000029014 | 98624\_at | U |
| ENSMUSP00000028907 | 100390\_s\_at | Z |
| ENSMUSP00000028935 | 103296\_at | M |
| ENSMUSP00000050048 | 103308\_at | M |
| ENSMUSP00000011055 | 104752\_at | U |
| ENSMUSP00000002844 | 95067\_at | U |
| ENSMUSP00000037274 | 96523\_at | Z |
| ENSMUSP00000057181 | 101062\_at | U |
| ENSMUSP00000005611 | 101195\_at | E |
| ENSMUSP00000044394 | 102651\_at | E |
| ENSMUSP00000039107 | 99412\_at | Z |
| ENSMUSP00000020444 | 98089\_at | M |
| ENSMUSP00000050080 | 99545\_at | Z |
| ENSMUSP00000032078 | 160562\_at | U |
| ENSMUSP00000036472 | 94555\_at | E |
| ENSMUSP00000005923 | 98557\_f\_at | U |
| ENSMUSP00000023967 | 104229\_at | Z |
| ENSMUSP00000001184 | 94688\_at | E |
| ENSMUSP00000041644 | 97311\_at | Z |
| ENSMUSP00000034299 | 97444\_at | E |
| ENSMUSP00000034435 | 160421\_r\_at | Z |
| ENSMUSP00000002087 | 93910\_at | E |
| ENSMUSP00000034552 | 92587\_at | U |
| ENSMUSP00000026378 | 95343\_at | M |
| ENSMUSP00000042729 | 98365\_at | U |
| ENSMUSP00000038580 | 99954\_at | U |
| ENSMUSP00000025079 | 104360\_at | E |
| ENSMUSP00000033300 | 104638\_at | M |
| ENSMUSP00000036861 | 96131\_at | E |
| ENSMUSP00000026173 | 96264\_at | E |
| ENSMUSP00000054008 | 97720\_at | M |
| ENSMUSP00000025363 | 92730\_at | M |
| ENSMUSP00000033277 | 160872\_f\_at | M |
| ENSMUSP00000028328 | 97986\_at | E |
| ENSMUSP00000021692 | 99020\_at | Z |
| ENSMUSP00000054501 | 92863\_at | E |
| ENSMUSP00000032116 | 99153\_at | Z |
| ENSMUSP00000029103 | 160170\_at | M |
| ENSMUSP00000005692 | 94163\_at | U |
| ENSMUSP00000002710 | 160448\_at | E |
| ENSMUSP00000026387 | 95752\_at | U |
| ENSMUSP00000020149 | 95885\_at | M |
| ENSMUSP00000041902 | 100291\_at | E |
| ENSMUSP00000030044 | 100436\_at | M |
| ENSMUSP00000034756 | 100569\_at | E |
| ENSMUSP00000041005 | 92195\_at | Z |
| ENSMUSP00000034432 | 93784\_at | E |
| ENSMUSP00000028233 | 103458\_at | Z |
| ENSMUSP00000027528 | 96540\_at | Z |
| ENSMUSP00000026198 | 161103\_at | E |
| ENSMUSP00000019213 | 96818\_at | Z |
| ENSMUSP00000003035 | 101357\_at | E |
| ENSMUSP00000034121 | 99562\_at | E |
| ENSMUSP00000024778 | 160724\_at | Z |
| ENSMUSP00000001319 | 160857\_at | Z |
| ENSMUSP00000002053 | 100712\_at | Z |
| ENSMUSP00000027916 | 103336\_r\_at | U |
| ENSMUSP00000057689 | 100978\_at | Z |
| ENSMUSP00000016016 | 97739\_at | E |
| ENSMUSP00000061576 | 92616\_at | E |
| ENSMUSP00000030404 | 102278\_at | U |
| ENSMUSP00000045050 | 103734\_at | E |
| ENSMUSP00000052374 | 103867\_at | Z |
| ENSMUSP00000055730 | 94049\_at | U |
| ENSMUSP00000028199 | 95505\_at | U |
| ENSMUSP00000047996 | 95493\_at | Z |
| ENSMUSP00000042181 | 100044\_at | Z |
| ENSMUSP00000031211 | 102739\_s\_at | M |
| ENSMUSP00000055998 | 101633\_at | M |
| ENSMUSP00000028681 | 101899\_at | Z |
| ENSMUSP00000032958 | 93392\_at | E |
| ENSMUSP00000024946 | 98527\_at | U |
| ENSMUSP00000014477 | 94776\_f\_at | M |
| ENSMUSP00000020969 | 103066\_at | U |
| ENSMUSP00000027860 | 103199\_at | M |
| ENSMUSP00000028771 | 161000\_i\_at | M |
| ENSMUSP00000053646 | 96281\_at | E |
| ENSMUSP00000062006 | 93162\_f\_at | Z |
| ENSMUSP00000002349 | 102421\_at | Z |
| ENSMUSP00000061233 | 92880\_at | Z |
| ENSMUSP00000041524 | 102554\_at | M |
| ENSMUSP00000030187 | 99448\_at | E |
| ENSMUSP00000029626 | 94458\_at | Z |
| ENSMUSP00000029353 | 100320\_at | E |
| ENSMUSP00000019133 | 100453\_at | E |
| ENSMUSP00000036865 | 97347\_at | Z |
| ENSMUSP00000026890 | 92224\_at | Z |
| ENSMUSP00000035254 | 98791\_at | M |
| ENSMUSP00000027105 | 92357\_at | E |
| ENSMUSP00000020628 | 98803\_at | M |
| ENSMUSP00000059196 | 98936\_at | U |
| ENSMUSP00000032850 | 103342\_at | U |
| ENSMUSP00000042543 | 98151\_s\_at | E |
| ENSMUSP00000021475 | 92544\_f\_at | U |
| ENSMUSP00000055978 | 95379\_at | Z |
| ENSMUSP00000051009 | 96835\_at | Z |
| ENSMUSP00000055551 | 96968\_at | M |
| ENSMUSP00000040245 | 98002\_at | M |
| ENSMUSP00000009693 | 101519\_at | E |
| ENSMUSP00000015769 | 102963\_at | E |
| ENSMUSP00000045366 | 104263\_at | E |
| ENSMUSP00000006687 | 94734\_at | M |
| ENSMUSP00000016400 | 92633\_at | E |
| ENSMUSP00000036508 | 94628\_r\_at | M |
| ENSMUSP00000023820 | 97889\_at | Z |
| ENSMUSP00000055673 | 102295\_at | U |
| ENSMUSP00000059839 | 103751\_at | M |
| ENSMUSP00000046999 | 92766\_at | Z |
| ENSMUSP00000033642 | 102307\_at | Z |
| ENSMUSP00000031420 | 103884\_at | U |
| ENSMUSP00000028123 | 92899\_at | U |
| ENSMUSP00000020298 | 99056\_at | U |
| ENSMUSP00000004057 | 160218\_at | Z |
| ENSMUSP00000041060 | 94199\_at | M |
| ENSMUSP00000027279 | 95655\_at | E |
| ENSMUSP00000061259 | 96307\_s\_at | U |
| ENSMUSP00000048704 | 101928\_at | U |
| ENSMUSP00000030763 | 93421\_at | E |
| ENSMUSP00000020726 | 98544\_at | U |
| ENSMUSP00000050935 | 103083\_at | U |
| ENSMUSP00000028389 | 104672\_at | Z |
| ENSMUSP00000043367 | 103228\_at | E |
| ENSMUSP00000033176 | 102000\_f\_at | U |
| ENSMUSP00000021665 | 101127\_at | E |
| ENSMUSP00000050077 | 99332\_at | Z |
| ENSMUSP00000030719 | 102716\_at | E |
| ENSMUSP00000032374 | 102849\_at | Z |
| ENSMUSP00000033770 | 99465\_at | M |
| ENSMUSP00000042351 | 160482\_at | U |
| ENSMUSP00000045325 | 104016\_at | M |
| ENSMUSP00000020704 | 103896\_f\_at | M |
| ENSMUSP00000048802 | 160627\_at | U |
| ENSMUSP00000005607 | 97364\_at | E |
| ENSMUSP00000028288 | 97497\_at | E |
| ENSMUSP00000007139 | 98953\_at | E |
| ENSMUSP00000028897 | 103492\_at | U |
| ENSMUSP00000025718 | 102048\_at | U |
| ENSMUSP00000034706 | 99013\_f\_at | Z |
| ENSMUSP00000037115 | 103504\_at | Z |
| ENSMUSP00000044493 | 93963\_at | M |
| ENSMUSP00000023088 | 103637\_at | U |
| ENSMUSP00000028677 | 95408\_at | Z |
| ENSMUSP00000020932 | 96852\_at | U |
| ENSMUSP00000024004 | 101403\_at | M |
| ENSMUSP00000060928 | 96985\_at | M |
| ENSMUSP00000038900 | 101536\_at | M |
| ENSMUSP00000022842 | 93295\_at | U |
| ENSMUSP00000023826 | 104280\_at | M |
| ENSMUSP00000040350 | 160903\_at | Z |
| ENSMUSP00000030290 | 104425\_at | Z |
| ENSMUSP00000062896 | 95718\_f\_at | Z |
| ENSMUSP00000000033 | 98623\_g\_at | M |
| ENSMUSP00000008907 | 92650\_at | E |
| ENSMUSP00000002599 | 96329\_at | E |
| ENSMUSP00000021054 | 92783\_at | M |
| ENSMUSP00000025915 | 102324\_at | U |
| ENSMUSP00000026925 | 103913\_at | U |
| ENSMUSP00000049229 | 99073\_at | E |
| ENSMUSP00000026704 | 160102\_at | U |
| ENSMUSP00000006478 | 160235\_at | E |
| ENSMUSP00000061448 | 95672\_at | U |
| ENSMUSP00000027674 | 98561\_at | Z |
| ENSMUSP00000011877 | 93571\_at | E |
| ENSMUSP00000028931 | 103245\_at | M |
| ENSMUSP00000055952 | 93716\_at | M |
| ENSMUSP00000021779 | 103378\_at | M |
| ENSMUSP00000032194 | 104701\_at | Z |
| ENSMUSP00000031748 | 93849\_at | E |
| ENSMUSP00000043512 | 95016\_at | Z |
| ENSMUSP00000023974 | 161023\_at | M |
| ENSMUSP00000024136 | 96605\_at | M |
| ENSMUSP00000020562 | 101011\_at | U |
| ENSMUSP00000052255 | 95149\_at | E |
| ENSMUSP00000027239 | 101144\_at | Z |
| ENSMUSP00000033960 | 96738\_at | E |
| ENSMUSP00000007245 | 101277\_at | Z |
| ENSMUSP00000015585 | 102733\_at | Z |
| ENSMUSP00000015361 | 98038\_at | E |
| ENSMUSP00000047061 | 104033\_at | Z |
| ENSMUSP00000002735 | 93048\_at | U |
| ENSMUSP00000032226 | 160511\_at | M |
| ENSMUSP00000023111 | 94504\_at | Z |
| ENSMUSP00000058352 | 160644\_at | M |
| ENSMUSP00000055313 | 104166\_at | E |
| ENSMUSP00000032754 | 94637\_at | Z |
| ENSMUSP00000035306 | 104299\_at | E |
| ENSMUSP00000059015 | 97696\_r\_at | E |
| ENSMUSP00000021733 | 98970\_at | U |
| ENSMUSP00000036227 | 92403\_at | E |
| ENSMUSP00000022371 | 97526\_at | E |
| ENSMUSP00000028307 | 102065\_at | Z |
| ENSMUSP00000002283 | 102198\_at | Z |
| ENSMUSP00000033597 | 103654\_at | Z |
| ENSMUSP00000055225 | 103787\_at | U |
| ENSMUSP00000027153 | 95425\_at | U |
| ENSMUSP00000036299 | 101420\_at | E |
| ENSMUSP00000029630 | 101553\_at | M |
| ENSMUSP00000022642 | 101723\_r\_at | E |
| ENSMUSP00000021552 | 93324\_at | E |
| ENSMUSP00000037835 | 98447\_at | Z |
| ENSMUSP00000009883 | 99891\_at | E |
| ENSMUSP00000049881 | 98149\_s\_at | U |
| ENSMUSP00000022806 | 160920\_at | Z |
| ENSMUSP00000050530 | 97802\_at | E |
| ENSMUSP00000033621 | 102341\_at | U |
| ENSMUSP00000042792 | 97935\_at | Z |
| ENSMUSP00000034074 | 103930\_at | Z |
| ENSMUSP00000048692 | 102619\_at | E |
| ENSMUSP00000040271 | 94112\_at | M |
| ENSMUSP00000053638 | 99368\_at | Z |
| ENSMUSP00000032722 | 94245\_at | M |
| ENSMUSP00000050220 | 160252\_at | E |
| ENSMUSP00000037131 | 160385\_at | U |
| ENSMUSP00000054307 | 95701\_at | Z |
| ENSMUSP00000027748 | 94378\_at | Z |
| ENSMUSP00000056988 | 100373\_at | E |
| ENSMUSP00000055535 | 101962\_at | M |
| ENSMUSP00000023837 | 97267\_at | Z |
| ENSMUSP00000030254 | 93600\_at | Z |
| ENSMUSP00000034745 | 92277\_at | E |
| ENSMUSP00000040369 | 98856\_at | Z |
| ENSMUSP00000029438 | 103262\_at | E |
| ENSMUSP00000005329 | 103055\_r\_at | U |
| ENSMUSP00000033413 | 98989\_at | E |
| ENSMUSP00000035163 | 103407\_at | Z |
| ENSMUSP00000050648 | 93999\_at | U |
| ENSMUSP00000058629 | 161040\_at | Z |
| ENSMUSP00000044610 | 95299\_at | E |
| ENSMUSP00000062555 | 101161\_at | Z |
| ENSMUSP00000030455 | 96888\_at | U |
| ENSMUSP00000050995 | 102750\_at | Z |
| ENSMUSP00000037275 | 102883\_at | E |
| ENSMUSP00000060537 | 98055\_at | Z |
| ENSMUSP00000023405 | 101439\_at | E |
| ENSMUSP00000022814 | 104050\_at | Z |
| ENSMUSP00000060752 | 94521\_at | U |
| ENSMUSP00000028691 | 99644\_at | E |
| ENSMUSP00000028213 | 160661\_at | Z |
| ENSMUSP00000027897 | 160794\_at | E |
| ENSMUSP00000034727 | 104328\_at | U |
| ENSMUSP00000007949 | 160939\_at | E |
| ENSMUSP00000056369 | 96087\_at | E |
| ENSMUSP00000061519 | 100927\_at | E |
| ENSMUSP00000052302 | 92420\_at | M |
| ENSMUSP00000027495 | 97543\_at | E |
| ENSMUSP00000022573 | 92553\_at | U |
| ENSMUSP00000058753 | 103671\_at | U |
| ENSMUSP00000029541 | 92686\_at | U |
| ENSMUSP00000041907 | 103816\_at | Z |
| ENSMUSP00000006713 | 103949\_at | Z |
| ENSMUSP00000048832 | 95442\_at | E |
| ENSMUSP00000025998 | 160138\_at | Z |
| ENSMUSP00000036543 | 100126\_at | E |
| ENSMUSP00000056293 | 101715\_at | M |
| ENSMUSP00000028473 | 98597\_at | E |
| ENSMUSP00000019038 | 98609\_at | E |
| ENSMUSP00000047725 | 104604\_at | M |
| ENSMUSP00000021271 | 93619\_at | Z |
| ENSMUSP00000045933 | 96230\_at | Z |
| ENSMUSP00000032454 | 161059\_at | U |
| ENSMUSP00000017798 | 92962\_at | M |
| ENSMUSP00000059174 | 102636\_at | U |
| ENSMUSP00000041089 | 92470\_f\_at | M |
| ENSMUSP00000048675 | 160414\_at | Z |
| ENSMUSP00000003779 | 94407\_at | E |
| ENSMUSP00000036230 | 104069\_at | Z |
| ENSMUSP00000042827 | 97151\_at | Z |
| ENSMUSP00000009256 | 97284\_at | M |
| ENSMUSP00000028239 | 93750\_at | E |
| ENSMUSP00000056389 | 97429\_at | E |
| ENSMUSP00000021241 | 98873\_at | Z |
| ENSMUSP00000015145 | 92439\_at | Z |
| ENSMUSP00000062554 | 103557\_at | E |
| ENSMUSP00000004588 | 100061\_f\_at | Z |
| ENSMUSP00000051577 | 95328\_at | E |
| ENSMUSP00000026461 | 96772\_at | U |
| ENSMUSP00000055737 | 101589\_at | M |
| ENSMUSP00000032864 | 93082\_at | E |
| ENSMUSP00000024934 | 104212\_at | E |
| ENSMUSP00000022817 | 99806\_at | E |
| ENSMUSP00000051642 | 104345\_at | E |
| ENSMUSP00000028161 | 99939\_at | U |
| ENSMUSP00000020163 | 160823\_at | U |
| ENSMUSP00000030206 | 97705\_at | E |
| ENSMUSP00000035808 | 92715\_at | E |
| ENSMUSP00000027299 | 102244\_at | Z |
| ENSMUSP00000049560 | 92848\_at | U |
| ENSMUSP00000060513 | 103833\_at | E |
| ENSMUSP00000022856 | 99005\_at | E |
| ENSMUSP00000016397 | 94015\_at | Z |
| ENSMUSP00000030726 | 99138\_at | E |
| ENSMUSP00000017460 | 94148\_at | M |
| ENSMUSP00000034350 | 95592\_at | E |
| ENSMUSP00000030896 | 95737\_at | M |
| ENSMUSP00000048464 | 101998\_at | M |
| ENSMUSP00000049729 | 98481\_at | Z |
| ENSMUSP00000035614 | 103032\_at | Z |
| ENSMUSP00000015157 | 98626\_at | E |
| ENSMUSP00000029625 | 93503\_at | Z |
| ENSMUSP00000023828 | 93636\_at | M |
| ENSMUSP00000027407 | 103298\_at | Z |
| ENSMUSP00000041934 | 104621\_at | E |
| ENSMUSP00000030760 | 93769\_at | E |
| ENSMUSP00000061474 | 104754\_at | E |
| ENSMUSP00000049973 | 161076\_at | U |
| ENSMUSP00000028463 | 95069\_at | E |
| ENSMUSP00000034594 | 96525\_at | M |
| ENSMUSP00000057512 | 96658\_at | E |
| ENSMUSP00000029628 | 101064\_at | U |
| ENSMUSP00000054987 | 101197\_at | M |
| ENSMUSP00000056882 | 101209\_at | M |
| ENSMUSP00000023244 | 99414\_at | E |
| ENSMUSP00000044417 | 160431\_at | U |
| ENSMUSP00000039663 | 104086\_at | U |
| ENSMUSP00000053962 | 160564\_at | M |
| ENSMUSP00000062395 | 160697\_at | M |
| ENSMUSP00000020188 | 100552\_at | M |
| ENSMUSP00000023790 | 100685\_at | Z |
| ENSMUSP00000015435 | 97313\_at | E |
| ENSMUSP00000052866 | 98890\_at | U |
| ENSMUSP00000035056 | 97446\_at | U |
| ENSMUSP00000015516 | 92323\_at | E |
| ENSMUSP00000034292 | 92456\_at | M |
| ENSMUSP00000031399 | 92589\_at | U |
| ENSMUSP00000052788 | 103699\_i\_at | M |
| ENSMUSP00000055863 | 103719\_at | U |
| ENSMUSP00000022147 | 103620\_s\_at | Z |
| ENSMUSP00000040153 | 95345\_at | Z |
| ENSMUSP00000028159 | 96801\_at | U |
| ENSMUSP00000035113 | 95478\_at | E |
| ENSMUSP00000034808 | 101473\_at | Z |
| ENSMUSP00000040393 | 98101\_at | E |
| ENSMUSP00000049537 | 93244\_at | U |
| ENSMUSP00000051355 | 98367\_at | M |
| ENSMUSP00000029975 | 94700\_at | M |
| ENSMUSP00000046486 | 160840\_at | E |
| ENSMUSP00000023511 | 94833\_at | Z |
| ENSMUSP00000044714 | 160973\_at | Z |
| ENSMUSP00000004327 | 94966\_at | U |
| ENSMUSP00000062857 | 100961\_at | E |
| ENSMUSP00000021864 | 97722\_at | E |
| ENSMUSP00000002362 | 96266\_at | E |
| ENSMUSP00000022618 | 92732\_at | E |
| ENSMUSP00000006136 | 92865\_at | M |
| ENSMUSP00000062782 | 92998\_at | E |
| ENSMUSP00000021696 | 160172\_at | M |
| ENSMUSP00000017743 | 95621\_at | E |
| ENSMUSP00000023231 | 94298\_at | U |
| ENSMUSP00000059769 | 160317\_at | E |
| ENSMUSP00000034293 | 95754\_at | U |
| ENSMUSP00000047630 | 100438\_at | Z |
| ENSMUSP00000030613 | 93520\_at | E |
| ENSMUSP00000040332 | 103327\_at | Z |
| ENSMUSP00000044129 | 95086\_at | Z |
| ENSMUSP00000015011 | 96542\_at | E |
| ENSMUSP00000036640 | 101081\_at | U |
| ENSMUSP00000027285 | 96675\_at | E |
| ENSMUSP00000006221 | 102670\_at | E |
| ENSMUSP00000022416 | 102815\_at | E |
| ENSMUSP00000033121 | 104140\_s\_at | E |
| ENSMUSP00000001258 | 99564\_at | E |
| ENSMUSP00000061126 | 92600\_f\_at | E |
| ENSMUSP00000027512 | 160581\_at | U |
| ENSMUSP00000045460 | 104115\_at | E |
| ENSMUSP00000036872 | 94719\_at | U |
| ENSMUSP00000023880 | 97330\_at | U |
| ENSMUSP00000020885 | 96019\_at | Z |
| ENSMUSP00000057822 | 92340\_at | Z |
| ENSMUSP00000059495 | 100009\_r\_at | E |
| ENSMUSP00000003669 | 102014\_at | Z |
| ENSMUSP00000037497 | 102147\_at | U |
| ENSMUSP00000046046 | 103603\_at | U |
| ENSMUSP00000029682 | 103869\_at | Z |
| ENSMUSP00000033809 | 95507\_at | U |
| ENSMUSP00000021536 | 96951\_at | U |
| ENSMUSP00000005810 | 100046\_at | U |
| ENSMUSP00000060521 | 101502\_at | E |
| ENSMUSP00000021607 | 93261\_at | E |
| ENSMUSP00000016511 | 98384\_at | Z |
| ENSMUSP00000028883 | 99840\_at | M |
| ENSMUSP00000018403 | 98529\_at | M |
| ENSMUSP00000021621 | 103068\_at | U |
| ENSMUSP00000039761 | 93539\_at | M |
| ENSMUSP00000012028 | 104524\_at | E |
| ENSMUSP00000020831 | 94983\_at | Z |
| ENSMUSP00000027425 | 96283\_at | Z |
| ENSMUSP00000027218 | 102423\_at | U |
| ENSMUSP00000036203 | 102556\_at | Z |
| ENSMUSP00000019290 | 94182\_at | Z |
| ENSMUSP00000029787 | 160467\_at | M |
| ENSMUSP00000055431 | 100455\_at | E |
| ENSMUSP00000007831 | 100588\_at | Z |
| ENSMUSP00000032510 | 97216\_at | Z |
| ENSMUSP00000048079 | 98793\_at | Z |
| ENSMUSP00000025864 | 98938\_at | E |
| ENSMUSP00000025521 | 103477\_at | Z |
| ENSMUSP00000027219 | 93948\_at | E |
| ENSMUSP00000036793 | 96692\_at | Z |
| ENSMUSP00000014002 | 96837\_at | E |
| ENSMUSP00000057424 | 102832\_at | E |
| ENSMUSP00000049404 | 102965\_at | U |
| ENSMUSP00000026356 | 98137\_at | U |
| ENSMUSP00000020504 | 99581\_at | U |
| ENSMUSP00000034277 | 104132\_at | E |
| ENSMUSP00000053564 | 104265\_at | Z |
| ENSMUSP00000010753 | 99930\_s\_at | E |
| ENSMUSP00000045282 | 104398\_at | Z |
| ENSMUSP00000020979 | 160876\_at | E |
| ENSMUSP00000060390 | 94736\_at | E |
| ENSMUSP00000027409 | 100997\_at | Z |
| ENSMUSP00000043441 | 92490\_at | E |
| ENSMUSP00000020959 | 102031\_at | U |
| ENSMUSP00000061742 | 102164\_at | M |
| ENSMUSP00000027402 | 92635\_at | E |
| ENSMUSP00000018491 | 102297\_at | M |
| ENSMUSP00000031227 | 102309\_at | M |
| ENSMUSP00000061553 | 99058\_at | M |
| ENSMUSP00000048491 | 94068\_at | U |
| ENSMUSP00000014141 | 160075\_at | U |
| ENSMUSP00000062164 | 92577\_f\_at | U |
| ENSMUSP00000033043 | 99990\_at | E |
| ENSMUSP00000042232 | 103085\_at | E |
| ENSMUSP00000006679 | 104541\_at | Z |
| ENSMUSP00000061704 | 93689\_at | U |
| ENSMUSP00000019881 | 161008\_at | M |
| ENSMUSP00000006752 | 97901\_at | E |
| ENSMUSP00000039943 | 160605\_s\_at | E |
| ENSMUSP00000029368 | 92911\_at | E |
| ENSMUSP00000060926 | 102573\_at | M |
| ENSMUSP00000031200 | 101129\_at | U |
| ENSMUSP00000021164 | 94211\_at | M |
| ENSMUSP00000046127 | 160351\_at | U |
| ENSMUSP00000059986 | 160484\_at | E |
| ENSMUSP00000033133 | 160629\_at | Z |
| ENSMUSP00000058874 | 97100\_at | Z |
| ENSMUSP00000058221 | 97366\_at | E |
| ENSMUSP00000047395 | 92243\_at | E |
| ENSMUSP00000029692 | 92376\_at | E |
| ENSMUSP00000018431 | 93832\_at | Z |
| ENSMUSP00000049243 | 103494\_at | Z |
| ENSMUSP00000061910 | 103639\_at | U |
| ENSMUSP00000027833 | 96854\_at | U |
| ENSMUSP00000059420 | 96987\_at | E |
| ENSMUSP00000031447 | 101393\_at | E |
| ENSMUSP00000023759 | 101405\_at | E |
| ENSMUSP00000059941 | 98021\_at | E |
| ENSMUSP00000047210 | 98154\_at | E |
| ENSMUSP00000002292 | 98287\_at | U |
| ENSMUSP00000023002 | 99876\_at | Z |
| ENSMUSP00000047586 | 160893\_at | Z |
| ENSMUSP00000049175 | 94753\_at | E |
| ENSMUSP00000033058 | 104427\_at | E |
| ENSMUSP00000022782 | 96186\_at | Z |
| ENSMUSP00000015612 | 92652\_at | E |
| ENSMUSP00000042816 | 103770\_at | E |
| ENSMUSP00000027754 | 102326\_at | E |
| ENSMUSP00000001672 | 160092\_at | E |
| ENSMUSP00000036245 | 160104\_at | U |
| ENSMUSP00000023085 | 160237\_at | E |
| ENSMUSP00000046080 | 100080\_at | M |
| ENSMUSP00000035697 | 100054\_s\_at | U |
| ENSMUSP00000026408 | 101814\_at | Z |
| ENSMUSP00000015920 | 98430\_at | E |
| ENSMUSP00000058419 | 101947\_at | Z |
| ENSMUSP00000061109 | 93573\_at | M |
| ENSMUSP00000062848 | 93718\_at | E |
| ENSMUSP00000048827 | 96595\_at | Z |
| ENSMUSP00000055900 | 101013\_at | M |
| ENSMUSP00000055957 | 101146\_at | Z |
| ENSMUSP00000034744 | 102735\_at | E |
| ENSMUSP00000042691 | 94361\_at | U |
| ENSMUSP00000034622 | 99629\_at | Z |
| ENSMUSP00000022032 | 160513\_at | E |
| ENSMUSP00000003032 | 94494\_at | U |
| ENSMUSP00000033992 | 160646\_at | U |
| ENSMUSP00000033826 | 100501\_at | Z |
| ENSMUSP00000042389 | 94639\_at | M |
| ENSMUSP00000025846 | 160779\_at | E |
| ENSMUSP00000028553 | 97250\_at | E |
| ENSMUSP00000039252 | 100634\_at | U |
| ENSMUSP00000015903 | 97528\_at | E |
| ENSMUSP00000003828 | 92405\_at | E |
| ENSMUSP00000028841 | 98972\_at | E |
| ENSMUSP00000046465 | 103523\_at | E |
| ENSMUSP00000027149 | 103656\_at | E |
| ENSMUSP00000021698 | 95282\_at | U |
| ENSMUSP00000000767 | 95427\_at | E |
| ENSMUSP00000016401 | 96871\_at | E |
| ENSMUSP00000037528 | 94745\_f\_at | E |
| ENSMUSP00000013759 | 101422\_at | E |
| ENSMUSP00000042187 | 100099\_at | E |
| ENSMUSP00000001903 | 101555\_at | E |
| ENSMUSP00000033690 | 98316\_at | Z |
| ENSMUSP00000054919 | 103562\_f\_at | M |
| ENSMUSP00000047397 | 98449\_at | E |
| ENSMUSP00000033514 | 93326\_at | Z |
| ENSMUSP00000027592 | 104311\_at | E |
| ENSMUSP00000033473 | 99893\_at | Z |
| ENSMUSP00000033245 | 99905\_at | E |
| ENSMUSP00000002469 | 104444\_at | M |
| ENSMUSP00000060571 | 160922\_at | E |
| ENSMUSP00000034947 | 94915\_at | U |
| ENSMUSP00000035079 | 104577\_at | U |
| ENSMUSP00000015017 | 100910\_at | M |
| ENSMUSP00000040777 | 96348\_at | E |
| ENSMUSP00000036221 | 102210\_at | Z |
| ENSMUSP00000029587 | 97792\_at | Z |
| ENSMUSP00000002379 | 102343\_at | Z |
| ENSMUSP00000058671 | 97937\_at | Z |
| ENSMUSP00000023593 | 99104\_at | M |
| ENSMUSP00000028636 | 160121\_at | U |
| ENSMUSP00000062030 | 94114\_at | M |
| ENSMUSP00000028356 | 160387\_at | M |
| ENSMUSP00000060784 | 97003\_at | M |
| ENSMUSP00000002708 | 101831\_at | Z |
| ENSMUSP00000022529 | 101964\_at | U |
| ENSMUSP00000056307 | 98580\_at | U |
| ENSMUSP00000016157 | 98508\_s\_at | E |
| ENSMUSP00000025903 | 93602\_at | E |
| ENSMUSP00000015358 | 103264\_at | E |
| ENSMUSP00000014495 | 98858\_at | M |
| ENSMUSP00000032372 | 92279\_at | E |
| ENSMUSP00000061843 | 104720\_at | M |
| ENSMUSP00000036555 | 95035\_at | E |
| ENSMUSP00000001242 | 96757\_at | U |
| ENSMUSP00000038204 | 101163\_at | M |
| ENSMUSP00000057981 | 101308\_at | U |
| ENSMUSP00000027853 | 95058\_f\_at | E |
| ENSMUSP00000032629 | 102752\_at | E |
| ENSMUSP00000030412 | 99646\_at | E |
| ENSMUSP00000050389 | 104185\_at | Z |
| ENSMUSP00000021956 | 160663\_at | U |
| ENSMUSP00000031486 | 160808\_at | E |
| ENSMUSP00000046654 | 97412\_at | E |
| ENSMUSP00000021993 | 95472\_f\_at | E |
| ENSMUSP00000021610 | 92422\_at | M |
| ENSMUSP00000033604 | 92555\_at | Z |
| ENSMUSP00000025230 | 103673\_at | Z |
| ENSMUSP00000002172 | 92688\_at | Z |
| ENSMUSP00000057038 | 102229\_at | E |
| ENSMUSP00000000984 | 103818\_at | U |
| ENSMUSP00000018315 | 95444\_at | E |
| ENSMUSP00000059575 | 96900\_at | E |
| ENSMUSP00000036198 | 95577\_at | E |
| ENSMUSP00000020099 | 100128\_at | E |
| ENSMUSP00000063180 | 98333\_at | U |
| ENSMUSP00000044573 | 101717\_at | Z |
| ENSMUSP00000030669 | 99922\_at | U |
| ENSMUSP00000002911 | 98599\_at | E |
| ENSMUSP00000038870 | 94932\_at | Z |
| ENSMUSP00000023352 | 93476\_at | E |
| ENSMUSP00000000696 | 104606\_at | M |
| ENSMUSP00000045063 | 104739\_at | M |
| ENSMUSP00000025073 | 96232\_at | E |
| ENSMUSP00000023065 | 96498\_at | U |
| ENSMUSP00000021930 | 92831\_at | E |
| ENSMUSP00000001620 | 99121\_at | U |
| ENSMUSP00000028943 | 102638\_at | M |
| ENSMUSP00000013766 | 160271\_at | M |
| ENSMUSP00000000451 | 94264\_at | E |
| ENSMUSP00000052894 | 99387\_at | Z |
| ENSMUSP00000051863 | 94397\_at | E |
| ENSMUSP00000021332 | 160416\_at | U |
| ENSMUSP00000031144 | 160549\_at | U |
| ENSMUSP00000045638 | 100404\_at | U |
| ENSMUSP00000046012 | 100392\_at | M |
| ENSMUSP00000053977 | 97153\_at | Z |
| ENSMUSP00000047511 | 93752\_at | E |
| ENSMUSP00000028678 | 98875\_at | Z |
| ENSMUSP00000027554 | 92296\_at | E |
| ENSMUSP00000024709 | 103281\_at | E |
| ENSMUSP00000005606 | 103559\_at | E |
| ENSMUSP00000024338 | 95052\_at | M |
| ENSMUSP00000045039 | 101180\_at | E |
| ENSMUSP00000044554 | 96774\_at | Z |
| ENSMUSP00000055818 | 96919\_at | U |
| ENSMUSP00000033326 | 101458\_at | E |
| ENSMUSP00000054057 | 99530\_at | M |
| ENSMUSP00000034049 | 93084\_at | E |
| ENSMUSP00000006094 | 94540\_at | E |
| ENSMUSP00000022787 | 104214\_at | U |
| ENSMUSP00000056315 | 99808\_at | Z |
| ENSMUSP00000014812 | 160825\_at | E |
| ENSMUSP00000045409 | 94818\_at | U |
| ENSMUSP00000027114 | 160958\_at | Z |
| ENSMUSP00000046900 | 103690\_at | M |
| ENSMUSP00000040364 | 92717\_at | E |
| ENSMUSP00000010211 | 102379\_at | Z |
| ENSMUSP00000054801 | 99007\_at | U |
| ENSMUSP00000000271 | 103968\_at | E |
| ENSMUSP00000030316 | 100012\_at | M |
| ENSMUSP00000029209 | 95594\_at | E |
| ENSMUSP00000025935 | 101867\_at | U |
| ENSMUSP00000003442 | 98483\_at | Z |
| ENSMUSP00000052586 | 93360\_at | E |
| ENSMUSP00000044604 | 98878\_r\_at | E |
| ENSMUSP00000054023 | 103034\_at | E |
| ENSMUSP00000021793 | 94393\_r\_at | E |
| ENSMUSP00000034820 | 104623\_at | U |
| ENSMUSP00000033086 | 97971\_at | E |
| ENSMUSP00000062556 | 92981\_at | M |
| ENSMUSP00000036316 | 101199\_at | Z |
| ENSMUSP00000024831 | 160300\_at | M |
| ENSMUSP00000020374 | 94281\_at | E |
| ENSMUSP00000033236 | 94426\_at | E |
| ENSMUSP00000025276 | 160433\_at | E |
| ENSMUSP00000059253 | 104088\_at | Z |
| ENSMUSP00000025704 | 160699\_at | M |
| ENSMUSP00000056438 | 100421\_at | U |
| ENSMUSP00000045720 | 100554\_at | E |
| ENSMUSP00000060761 | 92180\_at | Z |
| ENSMUSP00000004140 | 93543\_f\_at | E |
| ENSMUSP00000038138 | 103310\_at | E |
| ENSMUSP00000049654 | 97448\_at | E |
| ENSMUSP00000020917 | 98892\_at | E |
| ENSMUSP00000015549 | 160986\_r\_at | Z |
| ENSMUSP00000027241 | 93914\_at | Z |
| ENSMUSP00000030325 | 92458\_at | U |
| ENSMUSP00000020017 | 103443\_at | M |
| ENSMUSP00000010007 | 95053\_s\_at | U |
| ENSMUSP00000059689 | 96803\_at | U |
| ENSMUSP00000036271 | 95347\_at | Z |
| ENSMUSP00000054941 | 101342\_at | Z |
| ENSMUSP00000028071 | 101475\_at | E |
| ENSMUSP00000000391 | 102931\_at | Z |
| ENSMUSP00000047719 | 99825\_at | U |
| ENSMUSP00000015576 | 99958\_at | Z |
| ENSMUSP00000043614 | 160842\_at | E |
| ENSMUSP00000026551 | 93379\_at | U |
| ENSMUSP00000031410 | 104364\_at | E |
| ENSMUSP00000024854 | 160975\_at | E |
| ENSMUSP00000049683 | 104509\_at | U |
| ENSMUSP00000020002 | 96135\_at | E |
| ENSMUSP00000057486 | 100963\_at | M |
| ENSMUSP00000028660 | 97724\_at | U |
| ENSMUSP00000045465 | 92601\_at | Z |
| ENSMUSP00000026892 | 97857\_at | E |
| ENSMUSP00000034408 | 92734\_at | Z |
| ENSMUSP00000052754 | 102263\_at | M |
| ENSMUSP00000042476 | 102396\_at | Z |
| ENSMUSP00000030588 | 92867\_at | Z |
| ENSMUSP00000056843 | 103852\_at | E |
| ENSMUSP00000034524 | 94034\_at | U |
| ENSMUSP00000021130 | 99157\_at | M |
| ENSMUSP00000019614 | 160174\_at | E |
| ENSMUSP00000030834 | 94167\_at | U |
| ENSMUSP00000031249 | 160319\_at | Z |
| ENSMUSP00000021048 | 95756\_at | U |
| ENSMUSP00000051384 | 101884\_at | M |
| ENSMUSP00000041208 | 98512\_at | M |
| ENSMUSP00000001009 | 103051\_at | Z |
| ENSMUSP00000004143 | 92199\_at | Z |
| ENSMUSP00000016450 | 98778\_at | E |
| ENSMUSP00000038090 | 100723\_f\_at | M |
| ENSMUSP00000016086 | 161080\_f\_at | M |
| ENSMUSP00000062025 | 102817\_at | E |
| ENSMUSP00000026496 | 99433\_at | E |
| ENSMUSP00000024223 | 99566\_at | U |
| ENSMUSP00000030995 | 160450\_at | E |
| ENSMUSP00000033050 | 160583\_at | M |
| ENSMUSP00000061593 | 104117\_at | U |
| ENSMUSP00000022867 | 100571\_at | Z |
| ENSMUSP00000003762 | 100716\_at | U |
| ENSMUSP00000003044 | 97332\_at | E |
| ENSMUSP00000053527 | 92342\_at | E |
| ENSMUSP00000050985 | 97465\_at | U |
| ENSMUSP00000020308 | 103460\_at | Z |
| ENSMUSP00000041483 | 93931\_at | M |
| ENSMUSP00000041357 | 103593\_at | M |
| ENSMUSP00000038577 | 160462\_f\_at | E |
| ENSMUSP00000046898 | 103738\_at | M |
| ENSMUSP00000055816 | 95364\_at | E |
| ENSMUSP00000034964 | 95497\_at | E |
| ENSMUSP00000057133 | 95509\_at | M |
| ENSMUSP00000034689 | 101492\_at | U |
| ENSMUSP00000045373 | 98120\_at | U |
| ENSMUSP00000037036 | 101637\_at | M |
| ENSMUSP00000019114 | 93130\_at | E |
| ENSMUSP00000002177 | 104381\_at | Z |
| ENSMUSP00000033009 | 99975\_at | E |
| ENSMUSP00000060884 | 94852\_at | U |
| ENSMUSP00000043442 | 160992\_at | E |
| ENSMUSP00000024786 | 104526\_at | Z |
| ENSMUSP00000034996 | 94985\_at | E |
| ENSMUSP00000029450 | 97741\_at | M |
| ENSMUSP00000030990 | 102280\_at | Z |
| ENSMUSP00000039864 | 99041\_at | Z |
| ENSMUSP00000062490 | 102558\_at | E |
| ENSMUSP00000025675 | 160191\_at | E |
| ENSMUSP00000048059 | 94184\_at | Z |
| ENSMUSP00000022345 | 160203\_at | U |
| ENSMUSP00000043390 | 160336\_at | U |
| ENSMUSP00000003404 | 100457\_at | Z |
| ENSMUSP00000024884 | 93672\_at | U |
| ENSMUSP00000022304 | 98795\_at | Z |
| ENSMUSP00000053818 | 98807\_at | E |
| ENSMUSP00000029685 | 103346\_at | E |
| ENSMUSP00000005669 | 102701\_at | E |
| ENSMUSP00000025752 | 98006\_at | E |
| ENSMUSP00000026879 | 102967\_at | U |
| ENSMUSP00000009138 | 94460\_at | E |
| ENSMUSP00000059099 | 100299\_f\_at | M |
| ENSMUSP00000029459 | 104134\_at | U |
| ENSMUSP00000024829 | 160612\_at | U |
| ENSMUSP00000028815 | 104267\_at | U |
| ENSMUSP00000006973 | 160745\_at | E |
| ENSMUSP00000023217 | 160878\_at | U |
| ENSMUSP00000057983 | 100600\_at | M |
| ENSMUSP00000015815 | 100733\_at | U |
| ENSMUSP00000022428 | 96038\_at | M |
| ENSMUSP00000022553 | 97482\_at | E |
| ENSMUSP00000050087 | 102033\_at | E |
| ENSMUSP00000020522 | 92637\_at | U |
| ENSMUSP00000029723 | 103755\_at | E |
| ENSMUSP00000051278 | 95381\_at | Z |
| ENSMUSP00000022696 | 95526\_at | E |
| ENSMUSP00000029214 | 95659\_at | E |
| ENSMUSP00000017860 | 101521\_at | E |
| ENSMUSP00000061247 | 101654\_at | E |
| ENSMUSP00000040610 | 98415\_at | M |
| ENSMUSP00000004392 | 93425\_at | M |
| ENSMUSP00000046967 | 104410\_at | Z |
| ENSMUSP00000032954 | 103087\_at | E |
| ENSMUSP00000033496 | 93558\_at | E |
| ENSMUSP00000025703 | 97891\_at | E |
| ENSMUSP00000059421 | 97903\_at | U |
| ENSMUSP00000023306 | 92913\_at | U |
| ENSMUSP00000050862 | 95465\_s\_at | M |
| ENSMUSP00000006686 | 102575\_at | M |
| ENSMUSP00000062070 | 99191\_at | M |
| ENSMUSP00000004614 | 160220\_at | M |
| ENSMUSP00000004554 | 99336\_at | U |
| ENSMUSP00000002840 | 99469\_at | U |
| ENSMUSP00000030587 | 160486\_at | Z |
| ENSMUSP00000040163 | 95935\_at | U |
| ENSMUSP00000026896 | 100474\_at | E |
| ENSMUSP00000058590 | 97090\_at | E |
| ENSMUSP00000001976 | 101930\_at | M |
| ENSMUSP00000024775 | 103363\_at | E |
| ENSMUSP00000034472 | 98957\_at | M |
| ENSMUSP00000042193 | 92378\_at | Z |
| ENSMUSP00000019708 | 103496\_at | E |
| ENSMUSP00000058730 | 103508\_at | Z |
| ENSMUSP00000026128 | 93967\_at | E |
| ENSMUSP00000002699 | 95001\_at | M |
| ENSMUSP00000025735 | 101407\_at | U |
| ENSMUSP00000028398 | 93033\_at | E |
| ENSMUSP00000033429 | 98289\_at | Z |
| ENSMUSP00000005503 | 93299\_at | U |
| ENSMUSP00000028882 | 94755\_at | M |
| ENSMUSP00000021916 | 160895\_at | M |
| ENSMUSP00000033512 | 160907\_at | E |
| ENSMUSP00000035120 | 96055\_at | M |
| ENSMUSP00000042373 | 97511\_at | U |
| ENSMUSP00000044612 | 92521\_at | E |
| ENSMUSP00000015723 | 97777\_at | Z |
| ENSMUSP00000027189 | 102328\_at | Z |
| ENSMUSP00000033905 | 92787\_at | U |
| ENSMUSP00000032412 | 160094\_at | E |
| ENSMUSP00000002765 | 94087\_at | E |
| ENSMUSP00000022256 | 160239\_at | E |
| ENSMUSP00000028509 | 94876\_f\_at | E |
| ENSMUSP00000027565 | 101816\_at | U |
| ENSMUSP00000023396 | 101949\_at | E |
| ENSMUSP00000029904 | 93575\_at | E |
| ENSMUSP00000036913 | 104560\_at | E |
| ENSMUSP00000044263 | 103249\_at | Z |
| ENSMUSP00000026927 | 104693\_at | U |
| ENSMUSP00000046125 | 96464\_at | Z |
| ENSMUSP00000023785 | 97920\_at | E |
| ENSMUSP00000060350 | 92930\_at | Z |
| ENSMUSP00000041030 | 101148\_at | E |
| ENSMUSP00000032237 | 94363\_at | E |
| ENSMUSP00000049721 | 99486\_at | E |
| ENSMUSP00000055969 | 160515\_at | E |
| ENSMUSP00000032844 | 94508\_at | M |
| ENSMUSP00000036932 | 160648\_at | U |
| ENSMUSP00000037629 | 100491\_at | U |
| ENSMUSP00000030513 | 97252\_at | U |
| ENSMUSP00000033880 | 100636\_at | Z |
| ENSMUSP00000042026 | 97385\_at | U |
| ENSMUSP00000029199 | 92262\_at | E |
| ENSMUSP00000028096 | 98841\_at | E |
| ENSMUSP00000024847 | 92407\_at | Z |
| ENSMUSP00000061498 | 93851\_at | U |
| ENSMUSP00000021776 | 98023\_r\_at | M |
| ENSMUSP00000024904 | 103525\_at | E |
| ENSMUSP00000028314 | 101424\_at | M |
| ENSMUSP00000033072 | 101557\_at | E |
| ENSMUSP00000014080 | 93050\_at | E |
| ENSMUSP00000040721 | 93183\_at | E |
| ENSMUSP00000019633 | 98318\_at | M |
| ENSMUSP00000028838 | 93328\_at | U |
| ENSMUSP00000038473 | 99895\_at | Z |
| ENSMUSP00000032512 | 94772\_at | Z |
| ENSMUSP00000061227 | 104313\_at | U |
| ENSMUSP00000045552 | 160924\_at | U |
| ENSMUSP00000037544 | 94917\_at | E |
| ENSMUSP00000043730 | 100912\_at | U |
| ENSMUSP00000033938 | 96217\_at | U |
| ENSMUSP00000029274 | 97661\_at | E |
| ENSMUSP00000006362 | 102212\_at | Z |
| ENSMUSP00000041427 | 102345\_at | Z |
| ENSMUSP00000021471 | 97939\_at | U |
| ENSMUSP00000052180 | 103801\_at | U |
| ENSMUSP00000032199 | 103934\_at | Z |
| ENSMUSP00000028630 | 99094\_at | U |
| ENSMUSP00000019638 | 99106\_at | E |
| ENSMUSP00000037417 | 160123\_at | Z |
| ENSMUSP00000058337 | 99239\_at | Z |
| ENSMUSP00000033464 | 94116\_at | Z |
| ENSMUSP00000030851 | 160256\_at | Z |
| ENSMUSP00000053273 | 95693\_at | U |
| ENSMUSP00000051909 | 101700\_at | M |
| ENSMUSP00000060748 | 97005\_at | M |
| ENSMUSP00000023519 | 98582\_at | U |
| ENSMUSP00000021259 | 98727\_at | U |
| ENSMUSP00000023207 | 93592\_at | U |
| ENSMUSP00000051368 | 103266\_at | Z |
| ENSMUSP00000031611 | 93737\_at | U |
| ENSMUSP00000023736 | 104722\_at | Z |
| ENSMUSP00000000087 | 103399\_at | Z |
| ENSMUSP00000049258 | 161044\_at | U |
| ENSMUSP00000060640 | 96481\_at | U |
| ENSMUSP00000054122 | 96626\_at | U |
| ENSMUSP00000045547 | 102621\_at | Z |
| ENSMUSP00000024879 | 102754\_at | E |
| ENSMUSP00000032270 | 99370\_at | Z |
| ENSMUSP00000005733 | 102887\_at | M |
| ENSMUSP00000054681 | 99515\_at | E |
| ENSMUSP00000034928 | 160532\_at | Z |
| ENSMUSP00000029141 | 160665\_at | E |
| ENSMUSP00000026476 | 104187\_at | M |
| ENSMUSP00000030742 | 160798\_at | U |
| ENSMUSP00000038263 | 97414\_at | E |
| ENSMUSP00000039636 | 92424\_at | Z |
| ENSMUSP00000031984 | 98991\_at | U |
| ENSMUSP00000045767 | 103542\_at | M |
| ENSMUSP00000019445 | 92557\_at | U |
| ENSMUSP00000026046 | 95446\_at | M |
| ENSMUSP00000020248 | 96902\_at | Z |
| ENSMUSP00000055563 | 96890\_at | U |
| ENSMUSP00000034955 | 95430\_f\_at | E |
| ENSMUSP00000035549 | 101719\_at | Z |
| ENSMUSP00000048036 | 99924\_at | E |
| ENSMUSP00000026672 | 160941\_at | U |
| ENSMUSP00000004470 | 104463\_at | E |
| ENSMUSP00000007309 | 161052\_r\_at | E |
| ENSMUSP00000033410 | 94934\_at | Z |
| ENSMUSP00000023144 | 99589\_f\_at | M |
| ENSMUSP00000005012 | 92700\_at | Z |
| ENSMUSP00000000220 | 100150\_f\_at | M |
| ENSMUSP00000060029 | 92833\_at | U |
| ENSMUSP00000047880 | 160140\_at | E |
| ENSMUSP00000024697 | 94266\_at | M |
| ENSMUSP00000026241 | 97742\_s\_at | Z |
| ENSMUSP00000022082 | 95722\_at | U |
| ENSMUSP00000029483 | 160418\_at | M |
| ENSMUSP00000026698 | 100394\_at | M |
| ENSMUSP00000033142 | 100406\_at | E |
| ENSMUSP00000002013 | 101850\_at | M |
| ENSMUSP00000061951 | 100539\_at | U |
| ENSMUSP00000027269 | 97155\_at | Z |
| ENSMUSP00000036783 | 93621\_at | E |
| ENSMUSP00000028609 | 103283\_at | Z |
| ENSMUSP00000015304 | 93754\_at | U |
| ENSMUSP00000022849 | 95054\_at | U |
| ENSMUSP00000028167 | 98984\_f\_at | U |
| ENSMUSP00000022921 | 96510\_at | Z |
| ENSMUSP00000017867 | 96643\_at | Z |
| ENSMUSP00000051371 | 101182\_at | M |
| ENSMUSP00000059539 | 102771\_at | U |
| ENSMUSP00000031619 | 98076\_at | E |
| ENSMUSP00000036039 | 99532\_at | Z |
| ENSMUSP00000005288 | 104071\_at | E |
| ENSMUSP00000024720 | 99665\_at | Z |
| ENSMUSP00000025932 | 104216\_at | U |
| ENSMUSP00000001819 | 160827\_at | E |
| ENSMUSP00000060721 | 104349\_at | E |
| ENSMUSP00000055885 | 100670\_at | E |
| ENSMUSP00000010250 | 97431\_at | U |
| ENSMUSP00000022875 | 100948\_at | M |
| ENSMUSP00000000402 | 92441\_at | U |
| ENSMUSP00000000175 | 92574\_at | E |
| ENSMUSP00000045449 | 97709\_at | M |
| ENSMUSP00000005488 | 103837\_at | Z |
| ENSMUSP00000005364 | 95330\_at | Z |
| ENSMUSP00000027194 | 94019\_at | E |
| ENSMUSP00000057243 | 160159\_at | E |
| ENSMUSP00000042001 | 95596\_at | E |
| ENSMUSP00000030112 | 100147\_at | U |
| ENSMUSP00000026001 | 101591\_at | U |
| ENSMUSP00000056576 | 101736\_at | Z |
| ENSMUSP00000032705 | 98485\_at | E |
| ENSMUSP00000059325 | 93362\_at | E |
| ENSMUSP00000047375 | 160700\_i\_at | Z |
| ENSMUSP00000023108 | 103036\_at | E |
| ENSMUSP00000026328 | 93495\_at | U |
| ENSMUSP00000017610 | 93507\_at | Z |
| ENSMUSP00000034543 | 104758\_at | U |
| ENSMUSP00000030489 | 97973\_at | Z |
| ENSMUSP00000016072 | 92850\_at | Z |
| ENSMUSP00000025586 | 99140\_at | U |
| ENSMUSP00000040505 | 102657\_at | Z |
| ENSMUSP00000055194 | 94283\_at | E |
| ENSMUSP00000025747 | 160290\_at | U |
| ENSMUSP00000005209 | 160302\_at | Z |
| ENSMUSP00000042606 | 160435\_at | M |
| ENSMUSP00000044367 | 94428\_at | U |
| ENSMUSP00000018441 | 160568\_at | U |
| ENSMUSP00000017142 | 100689\_at | M |
| ENSMUSP00000055412 | 97317\_at | M |
| ENSMUSP00000057353 | 92182\_at | E |
| ENSMUSP00000062723 | 93771\_at | M |
| ENSMUSP00000001402 | 98906\_at | E |
| ENSMUSP00000000704 | 103445\_at | Z |
| ENSMUSP00000036849 | 103578\_at | E |
| ENSMUSP00000009784 | 96793\_at | E |
| ENSMUSP00000055290 | 102800\_at | E |
| ENSMUSP00000033189 | 101344\_at | Z |
| ENSMUSP00000004326 | 102933\_at | Z |
| ENSMUSP00000037614 | 93248\_at | U |
| ENSMUSP00000029188 | 94704\_at | Z |
| ENSMUSP00000029877 | 160711\_at | U |
| ENSMUSP00000002331 | 104233\_at | E |
| ENSMUSP00000034570 | 160844\_at | U |
| ENSMUSP00000031750 | 160977\_at | E |
| ENSMUSP00000022200 | 104499\_at | Z |
| ENSMUSP00000032992 | 94837\_at | E |
| ENSMUSP00000030244 | 102972\_s\_at | Z |
| ENSMUSP00000003245 | 96004\_at | E |
| ENSMUSP00000056155 | 100965\_at | M |
| ENSMUSP00000019007 | 97726\_at | U |
| ENSMUSP00000013304 | 92603\_at | E |
| ENSMUSP00000047529 | 102265\_at | Z |
| ENSMUSP00000058866 | 92736\_at | U |
| ENSMUSP00000036585 | 102398\_at | Z |
| ENSMUSP00000058799 | 99026\_at | Z |
| ENSMUSP00000025893 | 103854\_at | E |
| ENSMUSP00000057122 | 103987\_at | M |
| ENSMUSP00000022419 | 99159\_at | U |
| ENSMUSP00000054697 | 95480\_at | E |
| ENSMUSP00000060227 | 94036\_at | M |
| ENSMUSP00000032060 | 160176\_at | U |
| ENSMUSP00000025311 | 94169\_at | Z |
| ENSMUSP00000033752 | 95625\_at | U |
| ENSMUSP00000026221 | 95758\_at | U |
| ENSMUSP00000057563 | 101620\_at | Z |
| ENSMUSP00000009520 | 100309\_at | Z |
| ENSMUSP00000051477 | 162297\_s\_at | Z |
| ENSMUSP00000028487 | 98514\_at | Z |
| ENSMUSP00000027730 | 103053\_at | Z |
| ENSMUSP00000014686 | 104642\_at | E |
| ENSMUSP00000047030 | 161097\_at | M |
| ENSMUSP00000025484 | 92595\_r\_at | U |
| ENSMUSP00000023358 | 97990\_at | E |
| ENSMUSP00000020770 | 101085\_at | Z |
| ENSMUSP00000058274 | 102674\_at | Z |
| ENSMUSP00000028637 | 99435\_at | Z |
| ENSMUSP00000033547 | 94445\_at | E |
| ENSMUSP00000038199 | 104119\_at | E |
| ENSMUSP00000035844 | 97334\_at | Z |
| ENSMUSP00000049157 | 92211\_at | M |
| ENSMUSP00000025700 | 98923\_at | U |
| ENSMUSP00000039098 | 102018\_at | E |
| ENSMUSP00000061985 | 93933\_at | E |
| ENSMUSP00000038641 | 103595\_at | Z |
| ENSMUSP00000030332 | 103607\_at | U |
| ENSMUSP00000031432 | 95100\_at | E |
| ENSMUSP00000044110 | 96955\_at | Z |
| ENSMUSP00000029933 | 98122\_at | E |
| ENSMUSP00000032723 | 101506\_at | E |
| ENSMUSP00000053551 | 99844\_at | Z |
| ENSMUSP00000007620 | 104250\_at | U |
| ENSMUSP00000020672 | 99977\_at | Z |
| ENSMUSP00000031004 | 104383\_at | U |
| ENSMUSP00000000310 | 94987\_at | E |
| ENSMUSP00000049860 | 104528\_at | E |
| ENSMUSP00000021667 | 96021\_at | U |
| ENSMUSP00000033762 | 96154\_at | U |
| ENSMUSP00000035863 | 100982\_at | U |
| ENSMUSP00000057854 | 96287\_at | U |
| ENSMUSP00000050021 | 97876\_at | U |
| ENSMUSP00000002495 | 92753\_at | E |
| ENSMUSP00000023489 | 102427\_at | M |
| ENSMUSP00000035610 | 103871\_at | E |
| ENSMUSP00000030901 | 94053\_at | U |
| ENSMUSP00000025847 | 99176\_at | Z |
| ENSMUSP00000026409 | 160193\_at | E |
| ENSMUSP00000028234 | 94186\_at | Z |
| ENSMUSP00000006557 | 95642\_at | E |
| ENSMUSP00000051399 | 160338\_at | M |
| ENSMUSP00000020649 | 100459\_at | U |
| ENSMUSP00000034590 | 93541\_at | E |
| ENSMUSP00000026296 | 93674\_at | E |
| ENSMUSP00000043000 | 95119\_at | E |
| ENSMUSP00000003285 | 96696\_at | U |
| ENSMUSP00000035157 | 101114\_at | E |
| ENSMUSP00000058723 | 96708\_at | E |
| ENSMUSP00000018343 | 92265\_f\_at | Z |
| ENSMUSP00000034230 | 98008\_at | M |
| ENSMUSP00000006286 | 102836\_at | E |
| ENSMUSP00000053235 | 102969\_at | Z |
| ENSMUSP00000001279 | 99452\_at | E |
| ENSMUSP00000031334 | 94462\_at | U |
| ENSMUSP00000033652 | 104003\_at | Z |
| ENSMUSP00000007370 | 104136\_at | Z |
| ENSMUSP00000013807 | 160614\_at | E |
| ENSMUSP00000005087 | 104269\_at | E |
| ENSMUSP00000000788 | 98480\_s\_at | E |
| ENSMUSP00000031905 | 92873\_f\_at | Z |
| ENSMUSP00000025940 | 104582\_g\_at | E |
| ENSMUSP00000001386 | 92361\_at | E |
| ENSMUSP00000021806 | 102035\_at | U |
| ENSMUSP00000034052 | 92494\_at | E |
| ENSMUSP00000056337 | 92506\_at | M |
| ENSMUSP00000045918 | 93950\_at | E |
| ENSMUSP00000062684 | 102168\_at | M |
| ENSMUSP00000028997 | 92639\_at | E |
| ENSMUSP00000028131 | 103624\_at | E |
| ENSMUSP00000034629 | 103757\_at | Z |
| ENSMUSP00000006764 | 93282\_at | U |
| ENSMUSP00000023737 | 99861\_at | Z |
| ENSMUSP00000043258 | 93427\_at | E |
| ENSMUSP00000025404 | 99994\_at | Z |
| ENSMUSP00000015499 | 103089\_at | M |
| ENSMUSP00000051603 | 94725\_f\_at | M |
| ENSMUSP00000032425 | 97862\_s\_at | E |
| ENSMUSP00000026579 | 96171\_at | Z |
| ENSMUSP00000015366 | 97760\_at | Z |
| ENSMUSP00000051837 | 97893\_at | U |
| ENSMUSP00000032133 | 102311\_at | Z |
| ENSMUSP00000001051 | 92770\_at | M |
| ENSMUSP00000024123 | 103900\_at | E |
| ENSMUSP00000018614 | 102577\_at | U |
| ENSMUSP00000047665 | 93512\_f\_at | U |
| ENSMUSP00000022220 | 160488\_at | U |
| ENSMUSP00000055602 | 95792\_at | M |
| ENSMUSP00000032759 | 97092\_at | Z |
| ENSMUSP00000038646 | 101932\_at | E |
| ENSMUSP00000020317 | 97237\_at | U |
| ENSMUSP00000036647 | 92247\_at | E |
| ENSMUSP00000019405 | 103232\_at | Z |
| ENSMUSP00000024699 | 93703\_at | M |
| ENSMUSP00000026119 | 103498\_at | Z |
| ENSMUSP00000033382 | 93836\_at | Z |
| ENSMUSP00000031351 | 95136\_at | Z |
| ENSMUSP00000032738 | 101131\_at | Z |
| ENSMUSP00000005578 | 96725\_at | U |
| ENSMUSP00000041104 | 96858\_at | U |
| ENSMUSP00000058809 | 102720\_at | Z |
| ENSMUSP00000028129 | 101397\_at | U |
| ENSMUSP00000027686 | 101409\_at | U |
| ENSMUSP00000025930 | 102853\_at | U |
| ENSMUSP00000009615 | 102986\_at | Z |
| ENSMUSP00000028807 | 104153\_at | U |
| ENSMUSP00000023101 | 104286\_at | E |
| ENSMUSP00000022099 | 160897\_at | U |
| ENSMUSP00000053751 | 160909\_at | M |
| ENSMUSP00000029646 | 100752\_at | Z |
| ENSMUSP00000027931 | 100885\_at | E |
| ENSMUSP00000035128 | 102052\_at | U |
| ENSMUSP00000015594 | 97779\_at | Z |
| ENSMUSP00000057515 | 92656\_at | Z |
| ENSMUSP00000050531 | 103641\_at | M |
| ENSMUSP00000006203 | 103774\_at | U |
| ENSMUSP00000060450 | 160096\_at | E |
| ENSMUSP00000022060 | 95412\_at | E |
| ENSMUSP00000032961 | 160108\_at | Z |
| ENSMUSP00000006188 | 100084\_at | Z |
| ENSMUSP00000055622 | 101540\_at | U |
| ENSMUSP00000025936 | 98301\_at | M |
| ENSMUSP00000034868 | 93311\_at | E |
| ENSMUSP00000033908 | 98434\_at | E |
| ENSMUSP00000006822 | 102202\_s\_at | E |
| ENSMUSP00000040706 | 93444\_at | Z |
| ENSMUSP00000022692 | 94900\_at | M |
| ENSMUSP00000043199 | 104695\_at | M |
| ENSMUSP00000059926 | 104707\_at | M |
| ENSMUSP00000058901 | 96200\_at | M |
| ENSMUSP00000057565 | 161029\_at | Z |
| ENSMUSP00000036094 | 97922\_at | U |
| ENSMUSP00000034076 | 92932\_at | M |
| ENSMUSP00000003152 | 160372\_at | E |
| ENSMUSP00000030192 | 94365\_at | U |
| ENSMUSP00000043956 | 99488\_at | M |
| ENSMUSP00000025486 | 160517\_at | Z |
| ENSMUSP00000034363 | 100493\_at | U |
| ENSMUSP00000025112 | 100505\_at | E |
| ENSMUSP00000000187 | 97121\_at | Z |
| ENSMUSP00000044548 | 97254\_at | U |
| ENSMUSP00000026626 | 98843\_at | E |
| ENSMUSP00000048573 | 93720\_at | U |
| ENSMUSP00000022641 | 98976\_at | E |
| ENSMUSP00000053916 | 93853\_at | U |
| ENSMUSP00000050978 | 103527\_at | E |
| ENSMUSP00000004200 | 99616\_s\_at | E |
| ENSMUSP00000022616 | 95286\_at | M |
| ENSMUSP00000027861 | 96742\_at | M |
| ENSMUSP00000054361 | 101281\_at | M |
| ENSMUSP00000000287 | 98042\_at | E |
| ENSMUSP00000022938 | 94641\_at | Z |
| ENSMUSP00000050773 | 104170\_at | Z |
| ENSMUSP00000038051 | 99897\_at | Z |
| ENSMUSP00000057965 | 99909\_at | Z |
| ENSMUSP00000000266 | 94774\_at | M |
| ENSMUSP00000028680 | 104315\_at | E |
| ENSMUSP00000050300 | 96074\_at | M |
| ENSMUSP00000015264 | 97530\_at | E |
| ENSMUSP00000033315 | 97796\_at | E |
| ENSMUSP00000002016 | 102214\_at | E |
| ENSMUSP00000030212 | 92673\_at | E |
| ENSMUSP00000026377 | 103803\_at | M |
| ENSMUSP00000032142 | 92818\_at | U |
| ENSMUSP00000040890 | 99096\_at | U |
| ENSMUSP00000056356 | 94707\_s\_at | M |
| ENSMUSP00000020659 | 160125\_at | M |
| ENSMUSP00000062209 | 160258\_at | E |
| ENSMUSP00000035222 | 95695\_at | E |
| ENSMUSP00000025045 | 95707\_at | M |
| ENSMUSP00000033650 | 101702\_at | Z |
| ENSMUSP00000052520 | 98451\_at | U |
| ENSMUSP00000032396 | 101835\_at | M |
| ENSMUSP00000022902 | 101968\_at | M |
| ENSMUSP00000030121 | 103002\_at | Z |
| ENSMUSP00000022142 | 93461\_at | E |
| ENSMUSP00000028198 | 98729\_at | E |
| ENSMUSP00000021090 | 101034\_at | E |
| ENSMUSP00000027914 | 96628\_at | U |
| ENSMUSP00000040904 | 102623\_at | Z |
| ENSMUSP00000053394 | 99372\_at | Z |
| ENSMUSP00000042285 | 94382\_at | Z |
| ENSMUSP00000021049 | 160534\_at | U |
| ENSMUSP00000021689 | 160667\_at | Z |
| ENSMUSP00000048731 | 97271\_at | E |
| ENSMUSP00000002487 | 93870\_at | E |
| ENSMUSP00000021402 | 97549\_at | E |
| ENSMUSP00000058441 | 92426\_at | Z |
| ENSMUSP00000031483 | 103544\_at | U |
| ENSMUSP00000045792 | 96031\_r\_at | Z |
| ENSMUSP00000033008 | 96892\_at | U |
| ENSMUSP00000030769 | 95448\_at | U |
| ENSMUSP00000030124 | 101310\_at | Z |
| ENSMUSP00000053033 | 96904\_at | U |
| ENSMUSP00000052778 | 98337\_at | Z |
| ENSMUSP00000029591 | 93214\_at | Z |
| ENSMUSP00000046188 | 93347\_at | E |
| ENSMUSP00000022153 | 104332\_at | E |
| ENSMUSP00000027675 | 99926\_at | Z |
| ENSMUSP00000040945 | 160943\_at | E |
| ENSMUSP00000031218 | 101139\_r\_at | M |
| ENSMUSP00000025169 | 94936\_at | Z |
| ENSMUSP00000025025 | 104598\_at | E |
| ENSMUSP00000023292 | 100931\_at | U |
| ENSMUSP00000047950 | 96236\_at | U |
| ENSMUSP00000028663 | 102231\_at | Z |
| ENSMUSP00000025585 | 92690\_at | M |
| ENSMUSP00000019998 | 97825\_at | M |
| ENSMUSP00000056402 | 102364\_at | Z |
| ENSMUSP00000042469 | 92835\_at | U |
| ENSMUSP00000031697 | 94002\_at | E |
| ENSMUSP00000010198 | 160142\_at | Z |
| ENSMUSP00000009877 | 94135\_at | Z |
| ENSMUSP00000054270 | 100130\_at | Z |
| ENSMUSP00000014578 | 101985\_at | Z |
| ENSMUSP00000021205 | 98613\_at | U |
| ENSMUSP00000022646 | 97157\_at | Z |
| ENSMUSP00000032469 | 103285\_at | E |
| ENSMUSP00000044734 | 104741\_at | E |
| ENSMUSP00000043860 | 96512\_at | E |
| ENSMUSP00000060700 | 96778\_at | E |
| ENSMUSP00000044217 | 101184\_at | U |
| ENSMUSP00000045879 | 102773\_at | U |
| ENSMUSP00000027979 | 99401\_at | E |
| ENSMUSP00000041963 | 102918\_at | Z |
| ENSMUSP00000015894 | 98078\_at | Z |
| ENSMUSP00000032437 | 99534\_at | M |
| ENSMUSP00000033049 | 99667\_at | E |
| ENSMUSP00000037320 | 93088\_at | M |
| ENSMUSP00000009036 | 160551\_at | E |
| ENSMUSP00000032141 | 160684\_at | U |
| ENSMUSP00000020424 | 160829\_at | M |
| ENSMUSP00000001178 | 97433\_at | E |
| ENSMUSP00000022212 | 92310\_at | E |
| ENSMUSP00000032170 | 97699\_at | Z |
| ENSMUSP00000040110 | 103694\_at | E |
| ENSMUSP00000004762 | 97509\_f\_at | Z |
| ENSMUSP00000026011 | 103706\_at | E |
| ENSMUSP00000016010 | 103839\_at | E |
| ENSMUSP00000027431 | 95332\_at | Z |
| ENSMUSP00000040779 | 96921\_at | U |
| ENSMUSP00000000924 | 100016\_at | E |
| ENSMUSP00000006519 | 101593\_at | E |
| ENSMUSP00000041729 | 101738\_at | M |
| ENSMUSP00000044953 | 99810\_at | U |
| ENSMUSP00000049007 | 93364\_at | Z |
| ENSMUSP00000031378 | 104482\_at | E |
| ENSMUSP00000024988 | 93497\_at | Z |
| ENSMUSP00000024777 | 103038\_at | E |
| ENSMUSP00000020657 | 93509\_at | E |
| ENSMUSP00000059898 | 94953\_at | E |
| ENSMUSP00000028818 | 104627\_at | U |
| ENSMUSP00000008733 | 96120\_at | U |
| ENSMUSP00000030518 | 95142\_s\_at | E |
| ENSMUSP00000034376 | 97975\_at | Z |
| ENSMUSP00000004780 | 103970\_at | E |
| ENSMUSP00000047447 | 99142\_at | E |
| ENSMUSP00000032962 | 102659\_at | E |
| ENSMUSP00000023285 | 160292\_at | U |
| ENSMUSP00000025200 | 94285\_at | M |
| ENSMUSP00000037304 | 100280\_at | M |
| ENSMUSP00000023401 | 160437\_at | U |
| ENSMUSP00000058741 | 98896\_at | E |
| ENSMUSP00000050196 | 92329\_at | E |
| ENSMUSP00000031530 | 98908\_at | E |
| ENSMUSP00000022239 | 103314\_at | M |
| ENSMUSP00000030242 | 96662\_at | E |
| ENSMUSP00000023851 | 97201\_s\_at | E |
| ENSMUSP00000041543 | 96795\_at | U |
| ENSMUSP00000031505 | 101213\_at | U |
| ENSMUSP00000029546 | 102790\_at | Z |
| ENSMUSP00000054591 | 102802\_at | M |
| ENSMUSP00000004646 | 98107\_at | U |
| ENSMUSP00000044644 | 94561\_at | E |
| ENSMUSP00000037968 | 104102\_at | U |
| ENSMUSP00000031860 | 93117\_at | E |
| ENSMUSP00000047864 | 104235\_at | E |
| ENSMUSP00000023022 | 98468\_r\_at | E |
| ENSMUSP00000031509 | 160713\_at | E |
| ENSMUSP00000022075 | 94694\_at | U |
| ENSMUSP00000031058 | 104368\_at | E |
| ENSMUSP00000049723 | 160846\_at | U |
| ENSMUSP00000033096 | 94839\_at | Z |
| ENSMUSP00000057595 | 100967\_at | U |
| ENSMUSP00000000641 | 92460\_at | Z |
| ENSMUSP00000020980 | 102001\_at | U |
| ENSMUSP00000032114 | 96139\_at | E |
| ENSMUSP00000021284 | 97728\_at | Z |
| ENSMUSP00000052881 | 92593\_at | U |
| ENSMUSP00000060822 | 102267\_at | E |
| ENSMUSP00000022744 | 92738\_at | M |
| ENSMUSP00000033418 | 103723\_at | M |
| ENSMUSP00000031976 | 103989\_at | M |
| ENSMUSP00000024817 | 99028\_at | E |
| ENSMUSP00000022341 | 94038\_at | Z |
| ENSMUSP00000024967 | 100033\_at | U |
| ENSMUSP00000029380 | 95627\_at | E |
| ENSMUSP00000047962 | 101622\_at | Z |
| ENSMUSP00000035551 | 93381\_at | M |
| ENSMUSP00000041282 | 99960\_at | E |
| ENSMUSP00000029773 | 160150\_f\_at | Z |
| ENSMUSP00000048383 | 104644\_at | E |
| ENSMUSP00000048271 | 96270\_at | Z |
| ENSMUSP00000001451 | 97992\_at | Z |
| ENSMUSP00000059206 | 102376\_r\_at | M |
| ENSMUSP00000013562 | 96548\_at | M |
| ENSMUSP00000051055 | 102410\_at | Z |
| ENSMUSP00000039418 | 102676\_at | M |
| ENSMUSP00000025659 | 160321\_at | E |
| ENSMUSP00000023247 | 99437\_at | M |
| ENSMUSP00000026304 | 92768\_s\_at | U |
| ENSMUSP00000038845 | 160587\_at | E |
| ENSMUSP00000033763 | 100575\_at | U |
| ENSMUSP00000055637 | 97203\_at | M |
| ENSMUSP00000033979 | 92213\_at | Z |
| ENSMUSP00000006627 | 97336\_at | E |
| ENSMUSP00000053426 | 98780\_at | Z |
| ENSMUSP00000022638 | 92346\_at | Z |
| ENSMUSP00000039910 | 103331\_at | Z |
| ENSMUSP00000054861 | 97469\_at | M |
| ENSMUSP00000054789 | 93802\_at | M |
| ENSMUSP00000022725 | 103597\_at | U |
| ENSMUSP00000059025 | 95102\_at | M |
| ENSMUSP00000027952 | 95368\_at | Z |
| ENSMUSP00000048524 | 96824\_at | E |
| ENSMUSP00000026827 | 98124\_at | U |
| ENSMUSP00000026670 | 93134\_at | Z |
| ENSMUSP00000029149 | 93267\_at | U |
| ENSMUSP00000026677 | 94723\_at | U |
| ENSMUSP00000046789 | 99846\_at | E |
| ENSMUSP00000041841 | 160730\_at | U |
| ENSMUSP00000024894 | 99979\_at | E |
| ENSMUSP00000062332 | 160863\_at | E |
| ENSMUSP00000051727 | 160996\_at | E |
| ENSMUSP00000034190 | 96023\_at | E |
| ENSMUSP00000058624 | 100984\_at | E |
| ENSMUSP00000040847 | 102151\_at | Z |
| ENSMUSP00000030169 | 96289\_at | U |
| ENSMUSP00000032994 | 92622\_at | U |
| ENSMUSP00000000966 | 97745\_at | Z |
| ENSMUSP00000059843 | 161058\_f\_at | M |
| ENSMUSP00000025900 | 102429\_at | U |
| ENSMUSP00000061463 | 99045\_at | U |
| ENSMUSP00000033706 | 99178\_at | Z |
| ENSMUSP00000028522 | 95511\_at | Z |
| ENSMUSP00000038652 | 100050\_at | Z |
| ENSMUSP00000029686 | 94188\_at | U |
| ENSMUSP00000007131 | 160207\_at | U |
| ENSMUSP00000022227 | 98400\_at | M |
| ENSMUSP00000038183 | 96577\_i\_at | Z |
| ENSMUSP00000025549 | 98533\_at | E |
| ENSMUSP00000029465 | 103072\_at | U |
| ENSMUSP00000027192 | 103217\_at | Z |
| ENSMUSP00000032496 | 93676\_at | M |
| ENSMUSP00000028225 | 96698\_at | E |
| ENSMUSP00000035768 | 102560\_at | Z |
| ENSMUSP00000037418 | 99321\_at | Z |
| ENSMUSP00000027871 | 102838\_at | Z |
| ENSMUSP00000002216 | 94331\_at | Z |
| ENSMUSP00000030266 | 104005\_at | Z |
| ENSMUSP00000004765 | 160471\_at | Z |
| ENSMUSP00000030993 | 160616\_at | Z |
| ENSMUSP00000042202 | 104138\_at | U |
| ENSMUSP00000031419 | 160749\_at | M |
| ENSMUSP00000023630 | 97220\_at | M |
| ENSMUSP00000058020 | 100737\_at | Z |
| ENSMUSP00000022223 | 92230\_at | U |
| ENSMUSP00000014684 | 97486\_at | E |
| ENSMUSP00000055114 | 102848\_f\_at | M |
| ENSMUSP00000058872 | 103481\_at | M |
| ENSMUSP00000025127 | 102037\_at | E |
| ENSMUSP00000030962 | 103759\_at | M |
| ENSMUSP00000037134 | 96915\_f\_at | M |
| ENSMUSP00000044603 | 96841\_at | E |
| ENSMUSP00000002881 | 101380\_at | U |
| ENSMUSP00000062261 | 100069\_at | E |
| ENSMUSP00000043543 | 101525\_at | Z |
| ENSMUSP00000022823 | 93151\_at | E |
| ENSMUSP00000052642 | 93284\_at | M |
| ENSMUSP00000051158 | 98419\_at | Z |
| ENSMUSP00000052392 | 160880\_at | Z |
| ENSMUSP00000033207 | 93429\_at | M |
| ENSMUSP00000027667 | 99996\_at | E |
| ENSMUSP00000022218 | 104547\_at | U |
| ENSMUSP00000031188 | 96796\_f\_at | U |
| ENSMUSP00000030348 | 160550\_i\_at | E |
| ENSMUSP00000019723 | 96318\_at | M |
| ENSMUSP00000061469 | 97907\_at | E |
| ENSMUSP00000032748 | 103890\_at | U |
| ENSMUSP00000023334 | 99062\_at | Z |
| ENSMUSP00000018767 | 92917\_at | E |
| ENSMUSP00000032492 | 95661\_at | Z |
| ENSMUSP00000061825 | 101801\_at | Z |
| ENSMUSP00000025078 | 97106\_at | E |
| ENSMUSP00000008966 | 93560\_at | U |
| ENSMUSP00000023813 | 103101\_at | U |
| ENSMUSP00000058993 | 93705\_at | Z |
| ENSMUSP00000057050 | 160934\_s\_at | E |
| ENSMUSP00000033051 | 98828\_at | Z |
| ENSMUSP00000006914 | 103367\_at | M |
| ENSMUSP00000016488 | 93838\_at | M |
| ENSMUSP00000048239 | 161012\_at | M |
| ENSMUSP00000045059 | 101000\_at | M |
| ENSMUSP00000028304 | 101133\_at | M |
| ENSMUSP00000061774 | 101399\_at | Z |
| ENSMUSP00000030372 | 98027\_at | Z |
| ENSMUSP00000028845 | 99471\_at | E |
| ENSMUSP00000048057 | 102988\_at | E |
| ENSMUSP00000032735 | 104022\_at | E |
| ENSMUSP00000001954 | 94481\_at | U |
| ENSMUSP00000026125 | 160633\_at | E |
| ENSMUSP00000051639 | 94626\_at | Z |
| ENSMUSP00000044790 | 160766\_at | E |
| ENSMUSP00000016680 | 104288\_at | E |
| ENSMUSP00000045524 | 100621\_at | E |
| ENSMUSP00000062539 | 160899\_at | M |
| ENSMUSP00000054731 | 97370\_at | Z |
| ENSMUSP00000020931 | 100887\_at | U |
| ENSMUSP00000032948 | 96059\_at | Z |
| ENSMUSP00000025385 | 97515\_at | U |
| ENSMUSP00000054621 | 102054\_at | U |
| ENSMUSP00000032200 | 103510\_at | U |
| ENSMUSP00000036952 | 92658\_at | E |
| ENSMUSP00000026154 | 103643\_at | E |
| ENSMUSP00000036031 | 95547\_at | E |
| ENSMUSP00000030986 | 100086\_at | Z |
| ENSMUSP00000057521 | 98291\_at | E |
| ENSMUSP00000028236 | 98303\_at | U |
| ENSMUSP00000005600 | 99880\_at | E |
| ENSMUSP00000053688 | 93446\_at | M |
| ENSMUSP00000058610 | 98569\_at | E |
| ENSMUSP00000055019 | 104431\_at | E |
| ENSMUSP00000017637 | 101571\_g\_at | M |
| ENSMUSP00000062942 | 94902\_at | U |
| ENSMUSP00000059909 | 93579\_at | Z |
| ENSMUSP00000034699 | 104564\_at | Z |
| ENSMUSP00000059498 | 104697\_at | E |
| ENSMUSP00000005220 | 96202\_at | U |
| ENSMUSP00000050812 | 98112\_r\_at | U |
| ENSMUSP00000051615 | 96335\_at | E |
| ENSMUSP00000025257 | 102330\_at | Z |
| ENSMUSP00000030201 | 97924\_at | U |
| ENSMUSP00000034382 | 92934\_at | M |
| ENSMUSP00000005709 | 94101\_at | M |
| ENSMUSP00000047218 | 95084\_f\_at | U |
| ENSMUSP00000027839 | 94367\_at | U |
| ENSMUSP00000020234 | 160519\_at | Z |
| ENSMUSP00000019362 | 100495\_at | E |
| ENSMUSP00000054389 | 100507\_at | Z |
| ENSMUSP00000039175 | 97123\_at | Z |
| ENSMUSP00000004485 | 92266\_at | M |
| ENSMUSP00000045937 | 93722\_at | E |
| ENSMUSP00000031280 | 98845\_at | Z |
| ENSMUSP00000000369 | 93855\_at | E |
| ENSMUSP00000053608 | 103529\_at | Z |
| ENSMUSP00000029082 | 93988\_at | U |
| ENSMUSP00000002452 | 96611\_at | Z |
| ENSMUSP00000006474 | 101150\_at | Z |
| ENSMUSP00000029734 | 96744\_at | E |
| ENSMUSP00000025493 | 99500\_at | U |
| ENSMUSP00000024885 | 94466\_f\_at | E |
| ENSMUSP00000010278 | 94510\_at | U |
| ENSMUSP00000030637 | 99633\_at | E |
| ENSMUSP00000000793 | 160650\_at | E |
| ENSMUSP00000021120 | 93187\_at | M |
| ENSMUSP00000035459 | 104172\_at | M |
| ENSMUSP00000039205 | 94643\_at | Z |
| ENSMUSP00000061661 | 99899\_at | Z |
| ENSMUSP00000039047 | 160783\_at | Z |
| ENSMUSP00000002790 | 104317\_at | E |
| ENSMUSP00000045286 | 92412\_s\_at | E |
| ENSMUSP00000024596 | 100916\_at | U |
| ENSMUSP00000053334 | 96076\_at | E |
| ENSMUSP00000043232 | 92542\_at | M |
| ENSMUSP00000043910 | 97798\_at | M |
| ENSMUSP00000035250 | 102216\_at | E |
| ENSMUSP00000032761 | 103660\_at | E |
| ENSMUSP00000032919 | 103793\_at | M |
| ENSMUSP00000029879 | 103805\_at | E |
| ENSMUSP00000055361 | 102289\_r\_at | Z |
| ENSMUSP00000020576 | 160127\_at | E |
| ENSMUSP00000020776 | 95564\_at | M |
| ENSMUSP00000006851 | 100115\_at | Z |
| ENSMUSP00000033074 | 95709\_at | M |
| ENSMUSP00000025946 | 98320\_at | U |
| ENSMUSP00000017832 | 101704\_at | Z |
| ENSMUSP00000004774 | 93330\_at | U |
| ENSMUSP00000003274 | 93608\_at | M |
| ENSMUSP00000060592 | 92888\_s\_at | E |
| ENSMUSP00000015227 | 104726\_at | M |
| ENSMUSP00000059558 | 102872\_f\_at | M |
| ENSMUSP00000033131 | 96352\_at | Z |
| ENSMUSP00000045392 | 92951\_at | Z |
| ENSMUSP00000024773 | 101169\_at | Z |
| ENSMUSP00000031723 | 99374\_at | M |
| ENSMUSP00000010807 | 160391\_at | U |
| ENSMUSP00000003635 | 94384\_at | M |
| ENSMUSP00000048683 | 160403\_at | M |
| ENSMUSP00000048443 | 104058\_at | E |
| ENSMUSP00000026572 | 160536\_at | E |
| ENSMUSP00000043123 | 97273\_at | E |
| ENSMUSP00000006718 | 98862\_at | Z |
| ENSMUSP00000015941 | 97418\_at | U |
| ENSMUSP00000005278 | 92428\_at | U |
| ENSMUSP00000029069 | 98995\_at | E |
| ENSMUSP00000028139 | 104762\_r\_at | Z |
| ENSMUSP00000001836 | 103413\_at | U |
| ENSMUSP00000031017 | 103546\_at | M |
| ENSMUSP00000035208 | 95317\_at | M |
| ENSMUSP00000007249 | 96761\_at | E |
| ENSMUSP00000004508 | 96894\_at | E |
| ENSMUSP00000062284 | 101312\_at | E |
| ENSMUSP00000023158 | 98061\_at | M |
| ENSMUSP00000004202 | 101445\_at | U |
| ENSMUSP00000005705 | 93071\_at | E |
| ENSMUSP00000057947 | 98339\_at | E |
| ENSMUSP00000037694 | 93216\_at | Z |
| ENSMUSP00000051844 | 104334\_at | E |
| ENSMUSP00000033160 | 160812\_at | E |
| ENSMUSP00000057002 | 93349\_at | Z |
| ENSMUSP00000021676 | 96093\_at | E |
| ENSMUSP00000005509 | 100933\_at | E |
| ENSMUSP00000042102 | 102233\_at | U |
| ENSMUSP00000029448 | 92692\_at | M |
| ENSMUSP00000023206 | 92704\_at | U |
| ENSMUSP00000012849 | 102366\_at | M |
| ENSMUSP00000019309 | 103822\_at | Z |
| ENSMUSP00000019964 | 92508\_s\_at | E |
| ENSMUSP00000021892 | 97678\_r\_at | E |
| ENSMUSP00000004784 | 94004\_at | E |
| ENSMUSP00000030698 | 160277\_at | E |
| ENSMUSP00000032214 | 95726\_at | Z |
| ENSMUSP00000056197 | 100398\_at | E |
| ENSMUSP00000033431 | 98470\_at | E |
| ENSMUSP00000062201 | 93480\_at | U |
| ENSMUSP00000019257 | 97159\_at | E |
| ENSMUSP00000012566 | 98615\_at | Z |
| ENSMUSP00000027491 | 93625\_at | U |
| ENSMUSP00000047053 | 104610\_at | Z |
| ENSMUSP00000048636 | 160999\_r\_at | E |
| ENSMUSP00000034302 | 104743\_at | Z |
| ENSMUSP00000034371 | 161065\_at | U |
| ENSMUSP00000021452 | 96514\_at | Z |
| ENSMUSP00000053803 | 101053\_at | U |
| ENSMUSP00000034499 | 96647\_at | E |
| ENSMUSP00000046209 | 101186\_at | M |
| ENSMUSP00000050148 | 102642\_at | U |
| ENSMUSP00000054708 | 99391\_at | Z |
| ENSMUSP00000038527 | 99536\_at | E |
| ENSMUSP00000047419 | 160553\_at | M |
| ENSMUSP00000001233 | 99669\_at | Z |
| ENSMUSP00000023918 | 97302\_at | U |
| ENSMUSP00000027615 | 97435\_at | Z |
| ENSMUSP00000039351 | 92445\_at | E |
| ENSMUSP00000026194 | 93901\_at | M |
| ENSMUSP00000029533 | 101755\_f\_at | M |
| ENSMUSP00000055654 | 103563\_at | M |
| ENSMUSP00000029663 | 92578\_at | U |
| ENSMUSP00000059378 | 95467\_at | Z |
| ENSMUSP00000030723 | 100018\_at | Z |
| ENSMUSP00000048218 | 93100\_at | E |
| ENSMUSP00000040416 | 98489\_at | Z |
| ENSMUSP00000036898 | 104351\_at | U |
| ENSMUSP00000032968 | 99945\_at | M |
| ENSMUSP00000051540 | 93499\_at | E |
| ENSMUSP00000020551 | 104629\_at | U |
| ENSMUSP00000022843 | 96122\_at | U |
| ENSMUSP00000022634 | 96255\_at | M |
| ENSMUSP00000005601 | 102250\_at | M |
| ENSMUSP00000027606 | 97844\_at | Z |
| ENSMUSP00000049162 | 102383\_at | E |
| ENSMUSP00000028378 | 99011\_at | U |
| ENSMUSP00000004892 | 92854\_at | E |
| ENSMUSP00000027415 | 92987\_at | E |
| ENSMUSP00000052052 | 94021\_at | Z |
| ENSMUSP00000042896 | 94154\_at | Z |
| ENSMUSP00000020552 | 160161\_at | M |
| ENSMUSP00000011981 | 160294\_at | M |
| ENSMUSP00000042988 | 160306\_at | M |
| ENSMUSP00000036021 | 95743\_at | M |
| ENSMUSP00000025712 | 100282\_at | Z |
| ENSMUSP00000032348 | 100427\_at | E |
| ENSMUSP00000049039 | 92186\_at | Z |
| ENSMUSP00000045892 | 93642\_at | E |
| ENSMUSP00000021626 | 93775\_at | M |
| ENSMUSP00000010192 | 104760\_at | E |
| ENSMUSP00000055036 | 103449\_at | E |
| ENSMUSP00000028382 | 95075\_at | E |
| ENSMUSP00000046830 | 96531\_at | U |
| ENSMUSP00000026292 | 96664\_at | E |
| ENSMUSP00000000572 | 101215\_at | Z |
| ENSMUSP00000031587 | 102792\_at | U |
| ENSMUSP00000048814 | 98109\_at | Z |
| ENSMUSP00000017908 | 94430\_at | M |
| ENSMUSP00000038961 | 93119\_at | E |
| ENSMUSP00000055016 | 104092\_at | M |
| ENSMUSP00000031316 | 94563\_at | M |
| ENSMUSP00000062677 | 94696\_at | Z |
| ENSMUSP00000021958 | 104237\_at | U |
| ENSMUSP00000037498 | 160715\_at | M |
| ENSMUSP00000022989 | 160848\_at | Z |
| ENSMUSP00000029633 | 100703\_at | Z |
| ENSMUSP00000022781 | 96008\_at | E |
| ENSMUSP00000062014 | 92462\_at | E |
| ENSMUSP00000026254 | 102003\_at | E |
| ENSMUSP00000040961 | 103580\_at | M |
| ENSMUSP00000019051 | 102269\_at | E |
| ENSMUSP00000033060 | 96940\_at | E |
| ENSMUSP00000038901 | 101624\_at | U |
| ENSMUSP00000026740 | 93383\_at | Z |
| ENSMUSP00000054614 | 100043\_f\_at | Z |
| ENSMUSP00000039776 | 103057\_at | U |
| ENSMUSP00000003808 | 104646\_at | Z |
| ENSMUSP00000020416 | 102412\_at | E |
| ENSMUSP00000034053 | 101089\_at | E |
| ENSMUSP00000033264 | 102678\_at | E |
| ENSMUSP00000000161 | 94171\_at | Z |
| ENSMUSP00000039048 | 99439\_at | Z |
| ENSMUSP00000006209 | 160323\_at | M |
| ENSMUSP00000023786 | 94316\_at | Z |
| ENSMUSP00000013966 | 95760\_at | E |
| ENSMUSP00000057225 | 94449\_at | Z |
| ENSMUSP00000045945 | 160589\_at | U |
| ENSMUSP00000034862 | 95905\_at | Z |
| ENSMUSP00000030814 | 100444\_at | E |
| ENSMUSP00000030238 | 101900\_at | U |
| ENSMUSP00000002551 | 100577\_at | E |
| ENSMUSP00000054421 | 97205\_at | E |
| ENSMUSP00000060277 | 92215\_at | M |
| ENSMUSP00000008589 | 103200\_at | M |
| ENSMUSP00000017357 | 92348\_at | Z |
| ENSMUSP00000019469 | 103333\_at | Z |
| ENSMUSP00000020437 | 103599\_at | M |
| ENSMUSP00000039683 | 93937\_at | Z |
| ENSMUSP00000029808 | 95092\_at | U |
| ENSMUSP00000022871 | 95104\_at | E |
| ENSMUSP00000020216 | 96959\_at | E |
| ENSMUSP00000062431 | 102954\_at | E |
| ENSMUSP00000001592 | 104121\_at | E |
| ENSMUSP00000020094 | 93136\_at | U |
| ENSMUSP00000021262 | 99848\_at | E |
| ENSMUSP00000001809 | 100720\_at | E |
| ENSMUSP00000030127 | 160998\_at | Z |
| ENSMUSP00000008280 | 100986\_at | E |
| ENSMUSP00000001155 | 102286\_at | E |
| ENSMUSP00000000646 | 92757\_at | Z |
| ENSMUSP00000054756 | 103875\_at | Z |
| ENSMUSP00000033519 | 95056\_r\_at | E |
| ENSMUSP00000020174 | 160064\_at | E |
| ENSMUSP00000025120 | 95513\_at | U |
| ENSMUSP00000049605 | 160197\_at | U |
| ENSMUSP00000043974 | 160209\_at | E |
| ENSMUSP00000030345 | 95646\_at | E |
| ENSMUSP00000027329 | 101774\_at | Z |
| ENSMUSP00000057047 | 98402\_at | E |
| ENSMUSP00000003592 | 98390\_at | U |
| ENSMUSP00000000105 | 101919\_at | M |
| ENSMUSP00000000335 | 98535\_at | U |
| ENSMUSP00000045134 | 93412\_at | M |
| ENSMUSP00000035172 | 103219\_at | Z |
| ENSMUSP00000005771 | 104663\_at | E |
| ENSMUSP00000032471 | 96567\_at | Z |
| ENSMUSP00000003145 | 102695\_at | M |
| ENSMUSP00000010605 | 99323\_at | Z |
| ENSMUSP00000048508 | 94200\_at | E |
| ENSMUSP00000025007 | 160473\_at | U |
| ENSMUSP00000021740 | 160618\_at | Z |
| ENSMUSP00000038505 | 100461\_at | E |
| ENSMUSP00000043217 | 100606\_at | M |
| ENSMUSP00000052791 | 92232\_at | Z |
| ENSMUSP00000049116 | 97355\_at | E |
| ENSMUSP00000061124 | 98811\_at | Z |
| ENSMUSP00000033751 | 92365\_at | Z |
| ENSMUSP00000006301 | 97488\_at | Z |
| ENSMUSP00000028916 | 98944\_at | E |
| ENSMUSP00000027214 | 103483\_at | U |
| ENSMUSP00000001565 | 102039\_at | E |
| ENSMUSP00000030170 | 92498\_at | E |
| ENSMUSP00000049985 | 96710\_at | E |
| ENSMUSP00000027471 | 96843\_at | E |
| ENSMUSP00000040464 | 101382\_at | E |
| ENSMUSP00000021463 | 98143\_at | Z |
| ENSMUSP00000034601 | 102971\_at | M |
| ENSMUSP00000060005 | 93020\_at | M |
| ENSMUSP00000004622 | 98276\_at | Z |
| ENSMUSP00000057006 | 97566\_f\_at | M |
| ENSMUSP00000051320 | 99865\_at | E |
| ENSMUSP00000062704 | 104271\_at | Z |
| ENSMUSP00000019608 | 99998\_at | Z |
| ENSMUSP00000030942 | 94875\_at | U |
| ENSMUSP00000007012 | 96042\_at | U |
| ENSMUSP00000054711 | 97909\_at | M |
| ENSMUSP00000048377 | 102315\_at | E |
| ENSMUSP00000035236 | 99064\_at | E |
| ENSMUSP00000003826 | 92919\_at | Z |
| ENSMUSP00000023807 | 103904\_at | Z |
| ENSMUSP00000046636 | 99197\_at | M |
| ENSMUSP00000030642 | 94219\_at | U |
| ENSMUSP00000029342 | 160226\_at | U |
| ENSMUSP00000022595 | 160359\_at | M |
| ENSMUSP00000059301 | 101803\_at | Z |
| ENSMUSP00000021097 | 97108\_at | U |
| ENSMUSP00000035220 | 97096\_at | U |
| ENSMUSP00000062420 | 98552\_at | M |
| ENSMUSP00000027193 | 93562\_at | Z |
| ENSMUSP00000003064 | 103091\_at | Z |
| ENSMUSP00000025183 | 103236\_at | E |
| ENSMUSP00000004233 | 93695\_at | U |
| ENSMUSP00000063168 | 92889\_r\_at | E |
| ENSMUSP00000052170 | 161014\_at | M |
| ENSMUSP00000001411 | 101135\_at | Z |
| ENSMUSP00000020640 | 99340\_at | U |
| ENSMUSP00000061132 | 102857\_at | M |
| ENSMUSP00000036289 | 98029\_at | E |
| ENSMUSP00000001522 | 160490\_at | E |
| ENSMUSP00000039046 | 93039\_at | U |
| ENSMUSP00000041234 | 160502\_at | E |
| ENSMUSP00000020372 | 99618\_at | M |
| ENSMUSP00000031008 | 160635\_at | E |
| ENSMUSP00000061933 | 160768\_at | M |
| ENSMUSP00000036322 | 92382\_at | E |
| ENSMUSP00000050940 | 97517\_at | U |
| ENSMUSP00000005719 | 92527\_at | U |
| ENSMUSP00000024794 | 103512\_at | U |
| ENSMUSP00000056592 | 103645\_at | U |
| ENSMUSP00000020334 | 95416\_at | E |
| ENSMUSP00000007105 | 93102\_f\_at | E |
| ENSMUSP00000015100 | 100088\_at | U |
| ENSMUSP00000043088 | 93448\_at | E |
| ENSMUSP00000042114 | 104433\_at | U |
| ENSMUSP00000028518 | 96192\_at | E |
| ENSMUSP00000006792 | 97781\_at | M |
| ENSMUSP00000018061 | 92803\_at | M |
| ENSMUSP00000023994 | 99081\_at | U |
| ENSMUSP00000000109 | 92936\_at | Z |
| ENSMUSP00000023732 | 92750\_s\_at | Z |
| ENSMUSP00000050375 | 94236\_at | M |
| ENSMUSP00000037627 | 160376\_at | M |
| ENSMUSP00000042860 | 94369\_at | E |
| ENSMUSP00000029441 | 100364\_at | E |
| ENSMUSP00000023246 | 101820\_at | M |
| ENSMUSP00000037317 | 100497\_at | E |
| ENSMUSP00000022890 | 100509\_at | E |
| ENSMUSP00000027071 | 97258\_at | U |
| ENSMUSP00000003971 | 103253\_at | Z |
| ENSMUSP00000017451 | 103386\_at | U |
| ENSMUSP00000005218 | 103005\_s\_at | M |
| ENSMUSP00000022496 | 93857\_at | E |
| ENSMUSP00000032198 | 95024\_at | E |
| ENSMUSP00000049923 | 161031\_at | Z |
| ENSMUSP00000034945 | 96613\_at | E |
| ENSMUSP00000038884 | 101152\_at | Z |
| ENSMUSP00000003461 | 96879\_at | U |
| ENSMUSP00000031615 | 102874\_at | E |
| ENSMUSP00000026990 | 99490\_at | U |
| ENSMUSP00000023505 | 99502\_at | M |
| ENSMUSP00000046684 | 104041\_at | E |
| ENSMUSP00000032480 | 99635\_at | E |
| ENSMUSP00000046090 | 104174\_at | U |
| ENSMUSP00000061361 | 160652\_at | U |
| ENSMUSP00000033707 | 94645\_at | Z |
| ENSMUSP00000029345 | 100773\_at | M |
| ENSMUSP00000023061 | 97401\_at | E |
| ENSMUSP00000020927 | 92411\_at | M |
| ENSMUSP00000024270 | 94217\_f\_at | M |
| ENSMUSP00000040376 | 103662\_at | E |
| ENSMUSP00000026845 | 102218\_at | M |
| ENSMUSP00000019434 | 103807\_at | M |
| ENSMUSP00000028466 | 95300\_at | Z |
| ENSMUSP00000031598 | 95433\_at | U |
| ENSMUSP00000023235 | 160129\_at | E |
| ENSMUSP00000021773 | 95566\_at | E |
| ENSMUSP00000034214 | 101561\_at | M |
| ENSMUSP00000027288 | 101706\_at | U |
| ENSMUSP00000019044 | 98322\_at | U |
| ENSMUSP00000031935 | 101839\_at | Z |
| ENSMUSP00000001256 | 99911\_at | Z |
| ENSMUSP00000032865 | 98588\_at | U |
| ENSMUSP00000047771 | 103006\_at | Z |
| ENSMUSP00000027943 | 93465\_at | Z |
| ENSMUSP00000023629 | 104728\_at | Z |
| ENSMUSP00000019987 | 96221\_at | M |
| ENSMUSP00000061051 | 92953\_at | M |
| ENSMUSP00000013559 | 102627\_at | E |
| ENSMUSP00000022258 | 160260\_at | E |
| ENSMUSP00000021538 | 94253\_at | E |
| ENSMUSP00000034533 | 99376\_at | Z |
| ENSMUSP00000050327 | 94386\_at | E |
| ENSMUSP00000034453 | 100381\_at | E |
| ENSMUSP00000000727 | 98731\_at | E |
| ENSMUSP00000023018 | 103270\_at | M |
| ENSMUSP00000029358 | 93741\_at | E |
| ENSMUSP00000021832 | 103415\_at | U |
| ENSMUSP00000026466 | 103548\_at | M |
| ENSMUSP00000048415 | 95041\_at | E |
| ENSMUSP00000029084 | 95319\_at | Z |
| ENSMUSP00000025191 | 101314\_at | M |
| ENSMUSP00000023134 | 98063\_at | M |
| ENSMUSP00000006025 | 101447\_at | E |
| ENSMUSP00000033990 | 102891\_at | U |
| ENSMUSP00000007251 | 99652\_at | E |
| ENSMUSP00000033325 | 93218\_at | E |
| ENSMUSP00000008451 | 94662\_at | E |
| ENSMUSP00000040860 | 101084\_f\_at | M |
| ENSMUSP00000041442 | 94795\_at | U |
| ENSMUSP00000027500 | 160814\_at | U |
| ENSMUSP00000031018 | 104336\_at | U |
| ENSMUSP00000035662 | 160947\_at | E |
| ENSMUSP00000003622 | 94807\_at | E |
| ENSMUSP00000000939 | 97551\_at | E |
| ENSMUSP00000056619 | 100935\_at | Z |
| ENSMUSP00000018295 | 97684\_at | Z |
| ENSMUSP00000021662 | 92561\_at | E |
| ENSMUSP00000053923 | 92694\_at | U |
| ENSMUSP00000032920 | 97829\_at | U |
| ENSMUSP00000030407 | 102235\_at | E |
| ENSMUSP00000061451 | 102368\_at | M |
| ENSMUSP00000048053 | 103824\_at | Z |
| ENSMUSP00000035877 | 101886\_f\_at | M |
| ENSMUSP00000044350 | 94006\_at | E |
| ENSMUSP00000025418 | 99129\_at | E |
| ENSMUSP00000006164 | 94139\_at | E |
| ENSMUSP00000002101 | 100001\_at | M |
| ENSMUSP00000045043 | 160279\_at | E |
| ENSMUSP00000009705 | 100134\_at | M |
| ENSMUSP00000026743 | 101989\_at | U |
| ENSMUSP00000038232 | 103023\_at | E |
| ENSMUSP00000023538 | 93482\_at | Z |
| ENSMUSP00000032340 | 98617\_at | Z |
| ENSMUSP00000005352 | 103289\_at | Z |
| ENSMUSP00000050065 | 100673\_f\_at | M |
| ENSMUSP00000036512 | 104745\_at | Z |
| ENSMUSP00000027075 | 103582\_r\_at | E |
| ENSMUSP00000053546 | 96516\_at | E |
| ENSMUSP00000041263 | 97960\_at | E |
| ENSMUSP00000000962 | 92970\_at | Z |
| ENSMUSP00000017904 | 101055\_at | E |
| ENSMUSP00000059582 | 96649\_at | U |
| ENSMUSP00000028165 | 101188\_at | Z |
| ENSMUSP00000044984 | 102644\_at | M |
| ENSMUSP00000056545 | 102777\_at | M |
| ENSMUSP00000023803 | 94270\_at | E |
| ENSMUSP00000012281 | 99393\_at | Z |
| ENSMUSP00000052969 | 99405\_at | M |
| ENSMUSP00000033087 | 160422\_at | U |
| ENSMUSP00000057375 | 160688\_at | E |
| ENSMUSP00000051752 | 100410\_at | E |
| ENSMUSP00000019722 | 97285\_f\_at | E |
| ENSMUSP00000009885 | 97304\_at | Z |
| ENSMUSP00000034885 | 92314\_at | Z |
| ENSMUSP00000026485 | 98881\_at | U |
| ENSMUSP00000040080 | 103432\_at | U |
| ENSMUSP00000004294 | 93891\_at | E |
| ENSMUSP00000035093 | 93903\_at | E |
| ENSMUSP00000011621 | 103565\_at | M |
| ENSMUSP00000004439 | 95336\_at | Z |
| ENSMUSP00000022457 | 95469\_at | U |
| ENSMUSP00000047923 | 96925\_at | U |
| ENSMUSP00000009530 | 101464\_at | Z |
| ENSMUSP00000057777 | 102920\_at | M |
| ENSMUSP00000036285 | 104220\_at | Z |
| ENSMUSP00000032736 | 93235\_at | E |
| ENSMUSP00000054054 | 99814\_at | Z |
| ENSMUSP00000030661 | 160831\_at | U |
| ENSMUSP00000050585 | 99947\_at | E |
| ENSMUSP00000023451 | 160964\_at | M |
| ENSMUSP00000032203 | 104486\_at | Z |
| ENSMUSP00000018918 | 103016\_s\_at | M |
| ENSMUSP00000044482 | 96124\_at | Z |
| ENSMUSP00000033289 | 100952\_at | Z |
| ENSMUSP00000004192 | 100124\_r\_at | E |
| ENSMUSP00000030541 | 96257\_at | Z |
| ENSMUSP00000006579 | 102252\_at | E |
| ENSMUSP00000027494 | 97979\_at | U |
| ENSMUSP00000041745 | 103841\_at | M |
| ENSMUSP00000027743 | 99146\_at | E |
| ENSMUSP00000027449 | 94156\_at | M |
| ENSMUSP00000031308 | 95612\_at | U |
| ENSMUSP00000031309 | 160296\_at | U |
| ENSMUSP00000007914 | 160308\_at | Z |
| ENSMUSP00000017851 | 100151\_at | E |
| ENSMUSP00000043366 | 100284\_at | E |
| ENSMUSP00000053703 | 101740\_at | Z |
| ENSMUSP00000014126 | 100429\_at | U |
| ENSMUSP00000054914 | 98501\_at | M |
| ENSMUSP00000033591 | 93511\_at | Z |
| ENSMUSP00000028839 | 103318\_at | U |
| ENSMUSP00000058654 | 93777\_at | E |
| ENSMUSP00000062474 | 161084\_at | M |
| ENSMUSP00000035955 | 95077\_at | E |
| ENSMUSP00000023362 | 96666\_at | E |
| ENSMUSP00000041053 | 102661\_at | Z |
| ENSMUSP00000053489 | 102794\_at | Z |
| ENSMUSP00000023601 | 94432\_at | E |
| ENSMUSP00000018755 | 104094\_at | E |
| ENSMUSP00000044035 | 104106\_at | E |
| ENSMUSP00000048395 | 160717\_at | E |
| ENSMUSP00000016231 | 94698\_at | Z |
| ENSMUSP00000000249 | 104239\_at | M |
| ENSMUSP00000021091 | 100560\_at | U |
| ENSMUSP00000024916 | 100705\_at | Z |
| ENSMUSP00000058894 | 98910\_at | M |
| ENSMUSP00000029400 | 92331\_at | U |
| ENSMUSP00000019677 | 92464\_at | E |
| ENSMUSP00000021300 | 93920\_at | E |
| ENSMUSP00000047570 | 95486\_at | E |
| ENSMUSP00000038839 | 96942\_at | Z |
| ENSMUSP00000031434 | 101481\_at | E |
| ENSMUSP00000022640 | 101626\_at | E |
| ENSMUSP00000011315 | 98375\_at | Z |
| ENSMUSP00000002091 | 93252\_at | E |
| ENSMUSP00000030815 | 99831\_at | M |
| ENSMUSP00000047413 | 93385\_at | U |
| ENSMUSP00000047400 | 94841\_at | U |
| ENSMUSP00000009828 | 103059\_at | M |
| ENSMUSP00000025786 | 104648\_at | Z |
| ENSMUSP00000013995 | 97730\_at | U |
| ENSMUSP00000040356 | 97863\_at | U |
| ENSMUSP00000003981 | 99030\_at | M |
| ENSMUSP00000021559 | 94040\_at | E |
| ENSMUSP00000052444 | 94173\_at | Z |
| ENSMUSP00000023965 | 92291\_f\_at | Z |
| ENSMUSP00000000049 | 94318\_at | Z |
| ENSMUSP00000034650 | 160458\_at | Z |
| ENSMUSP00000028106 | 100313\_at | Z |
| ENSMUSP00000040694 | 101902\_at | E |
| ENSMUSP00000036087 | 97195\_at | E |
| ENSMUSP00000045443 | 100402\_f\_at | Z |
| ENSMUSP00000038245 | 98784\_at | Z |
| ENSMUSP00000029935 | 103202\_at | E |
| ENSMUSP00000059339 | 98929\_at | M |
| ENSMUSP00000033598 | 93806\_at | Z |
| ENSMUSP00000034349 | 93794\_at | U |
| ENSMUSP00000001151 | 103335\_at | Z |
| ENSMUSP00000034746 | 103468\_at | M |
| ENSMUSP00000041611 | 93939\_at | Z |
| ENSMUSP00000059224 | 96550\_at | Z |
| ENSMUSP00000002846 | 96828\_at | U |
| ENSMUSP00000034329 | 101367\_at | M |
| ENSMUSP00000021922 | 102956\_at | Z |
| ENSMUSP00000023608 | 98128\_at | Z |
| ENSMUSP00000014387 | 93138\_at | M |
| ENSMUSP00000031555 | 160601\_at | Z |
| ENSMUSP00000025357 | 160734\_at | E |
| ENSMUSP00000025522 | 160867\_at | Z |
| ENSMUSP00000028517 | 160166\_r\_at | U |
| ENSMUSP00000002198 | 96027\_at | E |
| ENSMUSP00000029125 | 97471\_at | Z |
| ENSMUSP00000047855 | 102022\_at | U |
| ENSMUSP00000031497 | 92481\_at | E |
| ENSMUSP00000015238 | 92626\_at | Z |
| ENSMUSP00000023321 | 103611\_at | M |
| ENSMUSP00000057227 | 102288\_at | M |
| ENSMUSP00000034795 | 103744\_at | Z |
| ENSMUSP00000017808 | 103877\_at | E |
| ENSMUSP00000031895 | 99049\_at | Z |
| ENSMUSP00000026269 | 160066\_at | E |
| ENSMUSP00000031034 | 94059\_at | U |
| ENSMUSP00000022827 | 101510\_at | Z |
| ENSMUSP00000039115 | 95648\_at | E |
| ENSMUSP00000018310 | 101776\_at | Z |
| ENSMUSP00000032217 | 98392\_at | M |
| ENSMUSP00000025541 | 99981\_at | E |
| ENSMUSP00000030128 | 103076\_at | E |
| ENSMUSP00000018466 | 104532\_at | U |
| ENSMUSP00000057030 | 94991\_at | M |
| ENSMUSP00000047772 | 92902\_at | E |
| ENSMUSP00000021574 | 99180\_at | U |
| ENSMUSP00000023140 | 94190\_at | M |
| ENSMUSP00000000127 | 99325\_at | Z |
| ENSMUSP00000062641 | 96290\_f\_at | U |
| ENSMUSP00000036259 | 104616\_g\_at | U |
| ENSMUSP00000024874 | 160475\_at | E |
| ENSMUSP00000037718 | 95924\_at | M |
| ENSMUSP00000002313 | 100463\_at | M |
| ENSMUSP00000021920 | 100608\_at | U |
| ENSMUSP00000030490 | 92367\_at | M |
| ENSMUSP00000056282 | 103352\_at | U |
| ENSMUSP00000001042 | 93823\_at | Z |
| ENSMUSP00000059925 | 93956\_at | U |
| ENSMUSP00000034163 | 95123\_at | Z |
| ENSMUSP00000021564 | 96712\_at | Z |
| ENSMUSP00000031123 | 95389\_at | U |
| ENSMUSP00000031129 | 96845\_at | U |
| ENSMUSP00000021691 | 101384\_at | E |
| ENSMUSP00000025295 | 98278\_at | Z |
| ENSMUSP00000041983 | 104273\_at | U |
| ENSMUSP00000015237 | 160884\_at | E |
| ENSMUSP00000032472 | 94744\_at | E |
| ENSMUSP00000034709 | 96044\_at | M |
| ENSMUSP00000026168 | 96177\_at | E |
| ENSMUSP00000021579 | 92510\_at | Z |
| ENSMUSP00000051544 | 102317\_at | E |
| ENSMUSP00000051228 | 97790\_s\_at | U |
| ENSMUSP00000005520 | 100987\_f\_at | E |
| ENSMUSP00000023426 | 160228\_at | Z |
| ENSMUSP00000017858 | 97098\_at | U |
| ENSMUSP00000032568 | 93431\_at | E |
| ENSMUSP00000029162 | 98554\_at | U |
| ENSMUSP00000001365 | 93564\_at | U |
| ENSMUSP00000003681 | 96758\_s\_at | E |
| ENSMUSP00000032233 | 104682\_at | E |
| ENSMUSP00000026665 | 93697\_at | E |
| ENSMUSP00000036580 | 103238\_at | Z |
| ENSMUSP00000024936 | 93709\_at | Z |
| ENSMUSP00000008284 | 96320\_at | E |
| ENSMUSP00000054158 | 102581\_at | Z |
| ENSMUSP00000032998 | 101137\_at | U |
| ENSMUSP00000031767 | 102726\_at | M |
| ENSMUSP00000030925 | 99342\_at | Z |
| ENSMUSP00000060626 | 99475\_at | Z |
| ENSMUSP00000032520 | 160504\_at | M |
| ENSMUSP00000021854 | 94485\_at | U |
| ENSMUSP00000001984 | 104159\_at | Z |
| ENSMUSP00000056010 | 160637\_at | U |
| ENSMUSP00000049521 | 97374\_at | M |
| ENSMUSP00000050453 | 92384\_at | E |
| ENSMUSP00000031243 | 97519\_at | M |
| ENSMUSP00000018651 | 98963\_at | Z |
| ENSMUSP00000025249 | 93840\_at | M |
| ENSMUSP00000029786 | 102058\_at | U |
| ENSMUSP00000062607 | 161108\_r\_at | E |
| ENSMUSP00000024708 | 103514\_at | Z |
| ENSMUSP00000031544 | 93973\_at | E |
| ENSMUSP00000027332 | 95140\_at | Z |
| ENSMUSP00000053389 | 95418\_at | E |
| ENSMUSP00000053671 | 96862\_at | M |
| ENSMUSP00000007253 | 101546\_at | U |
| ENSMUSP00000033771 | 101679\_at | Z |
| ENSMUSP00000025968 | 98295\_at | E |
| ENSMUSP00000028823 | 98307\_at | E |
| ENSMUSP00000029950 | 104290\_at | M |
| ENSMUSP00000021011 | 94761\_at | M |
| ENSMUSP00000050136 | 160913\_at | M |
| ENSMUSP00000002095 | 104568\_at | U |
| ENSMUSP00000004232 | 94906\_at | U |
| ENSMUSP00000041534 | 96206\_at | Z |
| ENSMUSP00000034232 | 97783\_at | M |
| ENSMUSP00000022698 | 102334\_at | Z |
| ENSMUSP00000032491 | 92793\_at | M |
| ENSMUSP00000036373 | 99083\_at | E |
| ENSMUSP00000057055 | 103923\_at | M |
| ENSMUSP00000004965 | 160112\_at | E |
| ENSMUSP00000054634 | 94105\_at | E |
| ENSMUSP00000045191 | 94238\_at | M |
| ENSMUSP00000053048 | 160245\_at | Z |
| ENSMUSP00000039529 | 160378\_at | M |
| ENSMUSP00000035539 | 93388\_s\_at | M |
| ENSMUSP00000047358 | 101822\_at | Z |
| ENSMUSP00000028222 | 101955\_at | U |
| ENSMUSP00000026222 | 93581\_at | Z |
| ENSMUSP00000027951 | 103255\_at | E |
| ENSMUSP00000034388 | 104711\_at | U |
| ENSMUSP00000058718 | 103388\_at | M |
| ENSMUSP00000020749 | 93859\_at | U |
| ENSMUSP00000025306 | 95159\_at | Z |
| ENSMUSP00000037332 | 96615\_at | E |
| ENSMUSP00000015578 | 102876\_at | Z |
| ENSMUSP00000031028 | 95046\_s\_at | U |
| ENSMUSP00000025477 | 99504\_at | E |
| ENSMUSP00000019950 | 160521\_at | E |
| ENSMUSP00000054394 | 99637\_at | Z |
| ENSMUSP00000021278 | 160787\_at | M |
| ENSMUSP00000039018 | 92977\_s\_at | Z |
| ENSMUSP00000022784 | 97391\_at | E |
| ENSMUSP00000042134 | 97403\_at | U |
| ENSMUSP00000033063 | 98980\_at | Z |
| ENSMUSP00000040647 | 97536\_at | E |
| ENSMUSP00000007994 | 93990\_at | E |
| ENSMUSP00000031221 | 103797\_at | U |
| ENSMUSP00000018778 | 95290\_at | Z |
| ENSMUSP00000051259 | 101773\_r\_at | Z |
| ENSMUSP00000062902 | 101735\_f\_at | M |
| ENSMUSP00000043173 | 98324\_at | E |
| ENSMUSP00000002422 | 101708\_at | E |
| ENSMUSP00000005812 | 98457\_at | E |
| ENSMUSP00000047844 | 99913\_at | Z |
| ENSMUSP00000026270 | 160930\_at | E |
| ENSMUSP00000020575 | 104452\_at | Z |
| ENSMUSP00000001921 | 102351\_at | U |
| ENSMUSP00000026899 | 99112\_at | E |
| ENSMUSP00000025263 | 102629\_at | M |
| ENSMUSP00000028020 | 94122\_at | Z |
| ENSMUSP00000061152 | 160262\_at | E |
| ENSMUSP00000020578 | 160395\_at | E |
| ENSMUSP00000055340 | 100383\_at | E |
| ENSMUSP00000002274 | 101972\_at | E |
| ENSMUSP00000052463 | 98600\_at | M |
| ENSMUSP00000028083 | 94263\_f\_at | U |
| ENSMUSP00000031508 | 97277\_at | E |
| ENSMUSP00000038591 | 92287\_at | Z |
| ENSMUSP00000031768 | 98866\_at | Z |
| ENSMUSP00000023043 | 98999\_at | U |
| ENSMUSP00000003318 | 101171\_at | E |
| ENSMUSP00000032180 | 101316\_at | Z |
| ENSMUSP00000037055 | 101449\_at | M |
| ENSMUSP00000041455 | 98065\_at | E |
| ENSMUSP00000027012 | 102905\_at | Z |
| ENSMUSP00000023311 | 104060\_at | U |
| ENSMUSP00000002368 | 160687\_r\_at | E |
| ENSMUSP00000034234 | 94531\_at | E |
| ENSMUSP00000042419 | 160671\_at | E |
| ENSMUSP00000060844 | 94664\_at | Z |
| ENSMUSP00000051561 | 94797\_at | U |
| ENSMUSP00000022470 | 160949\_at | E |
| ENSMUSP00000038048 | 97420\_at | U |
| ENSMUSP00000006440 | 96109\_at | E |
| ENSMUSP00000034458 | 97553\_at | U |
| ENSMUSP00000021019 | 96554\_r\_at | E |
| ENSMUSP00000027530 | 92563\_at | E |
| ENSMUSP00000028086 | 92696\_at | Z |
| ENSMUSP00000027165 | 102237\_at | M |
| ENSMUSP00000028874 | 92225\_f\_at | U |
| ENSMUSP00000047333 | 103826\_at | Z |
| ENSMUSP00000062590 | 103959\_at | E |
| ENSMUSP00000059821 | 94008\_at | E |
| ENSMUSP00000032813 | 100003\_at | E |
| ENSMUSP00000025245 | 95585\_at | Z |
| ENSMUSP00000016678 | 100136\_at | M |
| ENSMUSP00000001830 | 101042\_f\_at | U |
| ENSMUSP00000029812 | 98427\_s\_at | U |
| ENSMUSP00000033582 | 101580\_at | M |
| ENSMUSP00000041343 | 101725\_at | E |
| ENSMUSP00000026156 | 101858\_at | Z |
| ENSMUSP00000034026 | 93351\_at | U |
| ENSMUSP00000002297 | 103025\_at | U |
| ENSMUSP00000001746 | 93484\_at | E |
| ENSMUSP00000029259 | 94940\_at | U |
| ENSMUSP00000032446 | 160151\_i\_at | E |
| ENSMUSP00000047199 | 104614\_at | Z |
| ENSMUSP00000054638 | 101057\_at | M |
| ENSMUSP00000031780 | 99395\_at | Z |
| ENSMUSP00000051704 | 94417\_at | Z |
| ENSMUSP00000056582 | 160557\_at | M |
| ENSMUSP00000036348 | 92309\_i\_at | E |
| ENSMUSP00000024959 | 93760\_at | E |
| ENSMUSP00000020302 | 95060\_at | U |
| ENSMUSP00000023719 | 96782\_at | E |
| ENSMUSP00000034811 | 101200\_at | U |
| ENSMUSP00000024957 | 101466\_at | E |
| ENSMUSP00000037088 | 93092\_at | M |
| ENSMUSP00000020333 | 99671\_at | Z |
| ENSMUSP00000038863 | 93104\_at | Z |
| ENSMUSP00000049814 | 99816\_at | U |
| ENSMUSP00000050029 | 104355\_at | E |
| ENSMUSP00000049851 | 99949\_at | M |
| ENSMUSP00000052704 | 104488\_at | M |
| ENSMUSP00000029142 | 94826\_at | U |
| ENSMUSP00000001416 | 92580\_at | U |
| ENSMUSP00000001480 | 96259\_at | U |
| ENSMUSP00000035883 | 99154\_s\_at | M |
| ENSMUSP00000017146 | 92858\_at | Z |
| ENSMUSP00000044626 | 99015\_at | Z |
| ENSMUSP00000034198 | 103843\_at | E |
| ENSMUSP00000055131 | 94025\_at | U |
| ENSMUSP00000005714 | 160165\_at | E |
| ENSMUSP00000023345 | 160298\_at | M |
| ENSMUSP00000030810 | 100020\_at | E |
| ENSMUSP00000035211 | 100286\_at | Z |
| ENSMUSP00000029406 | 101742\_at | E |
| ENSMUSP00000002839 | 101875\_at | E |
| ENSMUSP00000011975 | 98503\_at | E |
| ENSMUSP00000023087 | 93646\_at | E |
| ENSMUSP00000028583 | 161086\_at | Z |
| ENSMUSP00000030538 | 101074\_at | E |
| ENSMUSP00000033498 | 96668\_at | E |
| ENSMUSP00000002284 | 102663\_at | Z |
| ENSMUSP00000059967 | 102796\_at | M |
| ENSMUSP00000030200 | 100579\_s\_at | E |
| ENSMUSP00000026631 | 99424\_at | Z |
| ENSMUSP00000015719 | 94301\_at | E |
| ENSMUSP00000050165 | 160441\_at | M |
| ENSMUSP00000006646 | 99557\_at | M |
| ENSMUSP00000031601 | 94434\_at | M |
| ENSMUSP00000028098 | 104096\_at | E |
| ENSMUSP00000003621 | 160574\_at | E |
| ENSMUSP00000037346 | 104108\_at | E |
| ENSMUSP00000058098 | 160719\_at | U |
| ENSMUSP00000038485 | 100562\_at | E |
| ENSMUSP00000014476 | 100326\_f\_at | M |
| ENSMUSP00000030384 | 100695\_at | M |
| ENSMUSP00000025104 | 92200\_at | M |
| ENSMUSP00000046585 | 97456\_at | U |
| ENSMUSP00000060448 | 103451\_at | Z |
| ENSMUSP00000020768 | 92599\_at | U |
| ENSMUSP00000061912 | 103729\_at | Z |
| ENSMUSP00000018699 | 93433\_s\_at | Z |
| ENSMUSP00000045273 | 96811\_at | E |
| ENSMUSP00000053877 | 95488\_at | M |
| ENSMUSP00000026446 | 100039\_at | Z |
| ENSMUSP00000062496 | 101483\_at | M |
| ENSMUSP00000031664 | 98111\_at | U |
| ENSMUSP00000053914 | 93640\_s\_at | M |
| ENSMUSP00000032238 | 99700\_at | E |
| ENSMUSP00000023462 | 93254\_at | E |
| ENSMUSP00000005889 | 96511\_s\_at | E |
| ENSMUSP00000034462 | 99833\_at | E |
| ENSMUSP00000020091 | 93387\_at | U |
| ENSMUSP00000008094 | 104372\_at | U |
| ENSMUSP00000028148 | 160850\_at | U |
| ENSMUSP00000025773 | 94843\_at | E |
| ENSMUSP00000033311 | 94976\_at | E |
| ENSMUSP00000026452 | 97732\_at | M |
| ENSMUSP00000000342 | 92742\_at | M |
| ENSMUSP00000062639 | 102271\_at | E |
| ENSMUSP00000004374 | 97998\_at | Z |
| ENSMUSP00000051959 | 99032\_at | E |
| ENSMUSP00000026012 | 102416\_at | E |
| ENSMUSP00000060124 | 103993\_at | Z |
| ENSMUSP00000024639 | 94175\_at | M |
| ENSMUSP00000017065 | 160182\_at | U |
| ENSMUSP00000032936 | 95631\_at | U |
| ENSMUSP00000033913 | 160327\_at | Z |
| ENSMUSP00000042215 | 100315\_at | U |
| ENSMUSP00000062083 | 98786\_at | Z |
| ENSMUSP00000030487 | 101103\_at | U |
| ENSMUSP00000052931 | 96685\_at | M |
| ENSMUSP00000027175 | 101091\_at | E |
| ENSMUSP00000053320 | 99441\_at | U |
| ENSMUSP00000022637 | 102958\_at | Z |
| ENSMUSP00000045530 | 93007\_at | Z |
| ENSMUSP00000061311 | 160591\_at | Z |
| ENSMUSP00000059711 | 104258\_at | U |
| ENSMUSP00000041035 | 160736\_at | E |
| ENSMUSP00000026559 | 160869\_at | U |
| ENSMUSP00000019118 | 97340\_at | E |
| ENSMUSP00000030734 | 96029\_at | E |
| ENSMUSP00000026585 | 97473\_at | Z |
| ENSMUSP00000017061 | 102024\_at | M |
| ENSMUSP00000019585 | 103613\_at | U |
| ENSMUSP00000038924 | 103879\_at | U |
| ENSMUSP00000034022 | 160068\_at | Z |
| ENSMUSP00000051598 | 100056\_at | U |
| ENSMUSP00000042338 | 101778\_at | M |
| ENSMUSP00000039600 | 98406\_at | M |
| ENSMUSP00000055354 | 160124\_r\_at | E |
| ENSMUSP00000042413 | 98394\_at | Z |
| ENSMUSP00000003868 | 99850\_at | M |
| ENSMUSP00000009553 | 94860\_at | E |
| ENSMUSP00000022592 | 93416\_at | M |
| ENSMUSP00000031063 | 104534\_at | U |
| ENSMUSP00000032218 | 104667\_at | U |
| ENSMUSP00000030269 | 96160\_at | U |
| ENSMUSP00000002173 | 102300\_at | Z |
| ENSMUSP00000055048 | 92892\_at | M |
| ENSMUSP00000024112 | 102699\_at | E |
| ENSMUSP00000055679 | 94192\_at | M |
| ENSMUSP00000038192 | 160211\_at | E |
| ENSMUSP00000035904 | 99327\_at | Z |
| ENSMUSP00000021668 | 160344\_at | Z |
| ENSMUSP00000019622 | 101921\_at | E |
| ENSMUSP00000002391 | 97359\_at | E |
| ENSMUSP00000020512 | 93680\_at | E |
| ENSMUSP00000006948 | 103221\_at | E |
| ENSMUSP00000033934 | 103354\_at | Z |
| ENSMUSP00000047119 | 98948\_at | U |
| ENSMUSP00000023241 | 103487\_at | M |
| ENSMUSP00000062675 | 161132\_at | M |
| ENSMUSP00000004301 | 96847\_at | E |
| ENSMUSP00000006704 | 98014\_at | U |
| ENSMUSP00000050137 | 93024\_at | E |
| ENSMUSP00000041299 | 98147\_at | E |
| ENSMUSP00000038418 | 104142\_at | Z |
| ENSMUSP00000057557 | 99869\_at | Z |
| ENSMUSP00000046106 | AFFX-GapdhMur/M32599\_M\_st | Z |
| ENSMUSP00000000423 | 96046\_at | U |
| ENSMUSP00000002980 | 97502\_at | U |
| ENSMUSP00000031692 | 97490\_at | Z |
| ENSMUSP00000033842 | 102041\_at | Z |
| ENSMUSP00000025373 | 103630\_at | U |
| ENSMUSP00000050043 | 103908\_at | E |
| ENSMUSP00000014499 | 99068\_at | E |
| ENSMUSP00000055743 | 160085\_at | U |
| ENSMUSP00000060379 | 95401\_at | M |
| ENSMUSP00000054856 | 94078\_at | E |
| ENSMUSP00000022518 | 100073\_at | E |
| ENSMUSP00000046715 | 101807\_at | Z |
| ENSMUSP00000054343 | 98423\_at | M |
| ENSMUSP00000043849 | 93300\_at | Z |
| ENSMUSP00000032768 | 103052\_r\_at | Z |
| ENSMUSP00000019456 | 103095\_at | Z |
| ENSMUSP00000021060 | 93699\_at | U |
| ENSMUSP00000015236 | 96322\_at | E |
| ENSMUSP00000056693 | 97911\_at | Z |
| ENSMUSP00000056048 | 96588\_at | Z |
| ENSMUSP00000007005 | 101006\_at | U |
| ENSMUSP00000042213 | 92921\_at | M |
| ENSMUSP00000060365 | 102583\_at | M |
| ENSMUSP00000062134 | 160361\_at | E |
| ENSMUSP00000046557 | 99477\_at | Z |
| ENSMUSP00000015481 | 160494\_at | U |
| ENSMUSP00000022844 | 160506\_at | M |
| ENSMUSP00000001281 | 160639\_at | Z |
| ENSMUSP00000038367 | 100482\_at | E |
| ENSMUSP00000021077 | 97243\_at | Z |
| ENSMUSP00000054821 | 92253\_at | M |
| ENSMUSP00000021005 | 98832\_at | U |
| ENSMUSP00000025186 | 103371\_at | U |
| ENSMUSP00000047920 | 92386\_at | Z |
| ENSMUSP00000004381 | 98965\_at | E |
| ENSMUSP00000047186 | 93842\_at | M |
| ENSMUSP00000016172 | 103516\_at | Z |
| ENSMUSP00000029464 | 103649\_at | U |
| ENSMUSP00000020518 | 95275\_at | M |
| ENSMUSP00000041244 | 96864\_at | Z |
| ENSMUSP00000029641 | 96997\_at | Z |
| ENSMUSP00000027499 | 98031\_at | Z |
| ENSMUSP00000019829 | 101548\_at | Z |
| ENSMUSP00000023353 | 93041\_at | U |
| ENSMUSP00000020350 | 98297\_at | U |
| ENSMUSP00000042681 | 93174\_at | Z |
| ENSMUSP00000050119 | 98309\_at | Z |
| ENSMUSP00000027146 | 94630\_at | Z |
| ENSMUSP00000033833 | 93319\_at | E |
| ENSMUSP00000018041 | 104292\_at | E |
| ENSMUSP00000006692 | 160770\_at | U |
| ENSMUSP00000027038 | 99886\_at | Z |
| ENSMUSP00000020616 | 94896\_at | E |
| ENSMUSP00000032803 | 104437\_at | M |
| ENSMUSP00000014130 | 160915\_at | Z |
| ENSMUSP00000059235 | 96063\_at | E |
| ENSMUSP00000061336 | 100891\_at | M |
| ENSMUSP00000048877 | 100903\_at | E |
| ENSMUSP00000058574 | 96208\_at | M |
| ENSMUSP00000004773 | 97652\_at | Z |
| ENSMUSP00000046980 | 97785\_at | E |
| ENSMUSP00000023105 | 102203\_at | E |
| ENSMUSP00000035055 | 92795\_at | Z |
| ENSMUSP00000027290 | 102336\_at | Z |
| ENSMUSP00000060483 | 92807\_at | U |
| ENSMUSP00000019932 | 103780\_at | Z |
| ENSMUSP00000050983 | 103925\_at | Z |
| ENSMUSP00000026315 | 94107\_at | M |
| ENSMUSP00000060953 | 95551\_at | Z |
| ENSMUSP00000002026 | 160114\_at | E |
| ENSMUSP00000030069 | 98440\_at | U |
| ENSMUSP00000054309 | 103376\_s\_at | E |
| ENSMUSP00000030329 | 104713\_at | E |
| ENSMUSP00000044517 | 93728\_at | Z |
| ENSMUSP00000025853 | 96617\_at | E |
| ENSMUSP00000042647 | 102612\_at | Z |
| ENSMUSP00000032269 | 102878\_at | E |
| ENSMUSP00000012426 | 99361\_at | Z |
| ENSMUSP00000018429 | 94371\_at | E |
| ENSMUSP00000029423 | 99494\_at | U |
| ENSMUSP00000034944 | 99506\_at | E |
| ENSMUSP00000034283 | 99639\_at | E |
| ENSMUSP00000036360 | 104045\_at | E |
| ENSMUSP00000041019 | 100511\_at | E |
| ENSMUSP00000041202 | 160789\_at | E |
| ENSMUSP00000003741 | 97405\_at | E |
| ENSMUSP00000021537 | 97393\_at | U |
| ENSMUSP00000040412 | 92415\_at | M |
| ENSMUSP00000026613 | 97538\_at | U |
| ENSMUSP00000025579 | 102104\_f\_at | M |
| ENSMUSP00000002473 | 93992\_at | E |
| ENSMUSP00000035423 | 103666\_at | Z |
| ENSMUSP00000006776 | 95304\_at | E |
| ENSMUSP00000037643 | 95437\_at | U |
| ENSMUSP00000018744 | 98459\_at | U |
| ENSMUSP00000021672 | 93336\_at | E |
| ENSMUSP00000031325 | 99915\_at | M |
| ENSMUSP00000051168 | 104454\_at | U |
| ENSMUSP00000006112 | 93469\_at | Z |
| ENSMUSP00000019992 | 104587\_at | E |
| ENSMUSP00000011029 | 94925\_at | E |
| ENSMUSP00000053255 | 102220\_at | M |
| ENSMUSP00000000299 | 102353\_at | Z |
| ENSMUSP00000035053 | 92824\_at | U |
| ENSMUSP00000032338 | 103942\_at | U |
| ENSMUSP00000033392 | 92957\_at | Z |
| ENSMUSP00000004989 | 99978\_s\_at | E |
| ENSMUSP00000002891 | 94124\_at | U |
| ENSMUSP00000035121 | 160131\_at | E |
| ENSMUSP00000057660 | 94257\_at | E |
| ENSMUSP00000062382 | 95713\_at | E |
| ENSMUSP00000007042 | 160397\_at | E |
| ENSMUSP00000017925 | 160409\_at | Z |
| ENSMUSP00000000275 | 100385\_at | Z |
| ENSMUSP00000017153 | 98590\_at | Z |
| ENSMUSP00000031788 | 97279\_at | U |
| ENSMUSP00000034243 | 93612\_at | E |
| ENSMUSP00000020368 | 92289\_at | E |
| ENSMUSP00000029949 | 104730\_at | E |
| ENSMUSP00000028076 | 93878\_at | Z |
| ENSMUSP00000033163 | 95045\_at | Z |
| ENSMUSP00000049704 | 101781\_f\_at | E |
| ENSMUSP00000022317 | 96634\_at | E |
| ENSMUSP00000020864 | 101173\_at | E |
| ENSMUSP00000026427 | 96767\_at | E |
| ENSMUSP00000037045 | 101318\_at | Z |
| ENSMUSP00000046496 | 102907\_at | Z |
| ENSMUSP00000001974 | 99656\_at | U |
| ENSMUSP00000030125 | 160540\_at | Z |
| ENSMUSP00000039301 | 160673\_at | E |
| ENSMUSP00000033539 | 94799\_at | E |
| ENSMUSP00000035078 | 160818\_at | Z |
| ENSMUSP00000002360 | 96119\_s\_at | Z |
| ENSMUSP00000027927 | 97422\_at | U |
| ENSMUSP00000053643 | 100939\_at | M |
| ENSMUSP00000056067 | 93455\_s\_at | Z |
| ENSMUSP00000057026 | 92565\_at | E |
| ENSMUSP00000022718 | 103550\_at | Z |
| ENSMUSP00000014505 | 92698\_at | Z |
| ENSMUSP00000034166 | 103683\_at | U |
| ENSMUSP00000025178 | 103828\_at | E |
| ENSMUSP00000033248 | 96910\_at | U |
| ENSMUSP00000057236 | 100005\_at | E |
| ENSMUSP00000030684 | 101582\_at | U |
| ENSMUSP00000024742 | 101727\_at | U |
| ENSMUSP00000033630 | 93220\_at | Z |
| ENSMUSP00000033229 | 97180\_f\_at | M |
| ENSMUSP00000040877 | 93353\_at | U |
| ENSMUSP00000006377 | 99932\_at | M |
| ENSMUSP00000033501 | 104471\_at | U |
| ENSMUSP00000034267 | 93486\_at | U |
| ENSMUSP00000029000 | 94942\_at | U |
| ENSMUSP00000029989 | 104749\_at | U |
| ENSMUSP00000031251 | 102370\_at | U |
| ENSMUSP00000003445 | 97964\_at | U |
| ENSMUSP00000030085 | 92974\_at | M |
| ENSMUSP00000062072 | 92816\_r\_at | U |
| ENSMUSP00000005678 | 94141\_at | Z |
| ENSMUSP00000002711 | 160281\_at | E |
| ENSMUSP00000019535 | 94274\_at | E |
| ENSMUSP00000045111 | 95730\_at | Z |
| ENSMUSP00000004265 | 160426\_at | U |
| ENSMUSP00000001475 | 94419\_at | Z |
| ENSMUSP00000037259 | 101991\_at | U |
| ENSMUSP00000038256 | 100547\_at | E |
| ENSMUSP00000059494 | 97308\_at | Z |
| ENSMUSP00000027464 | 97296\_at | Z |
| ENSMUSP00000043903 | 103303\_at | Z |
| ENSMUSP00000046837 | 93762\_at | E |
| ENSMUSP00000020898 | 101075\_f\_at | Z |
| ENSMUSP00000043575 | 103436\_at | U |
| ENSMUSP00000036851 | 96651\_at | Z |
| ENSMUSP00000045873 | 96784\_at | Z |
| ENSMUSP00000038717 | 101335\_at | Z |
| ENSMUSP00000001156 | 101468\_at | Z |
| ENSMUSP00000031607 | 102924\_at | Z |
| ENSMUSP00000034405 | 94376\_s\_at | E |
| ENSMUSP00000050744 | 98084\_at | M |
| ENSMUSP00000033169 | 93094\_at | Z |
| ENSMUSP00000034946 | 94550\_at | E |
| ENSMUSP00000037921 | 160702\_at | E |
| ENSMUSP00000056027 | 94828\_at | E |
| ENSMUSP00000027697 | 96128\_at | E |
| ENSMUSP00000016208 | 100956\_at | U |
| ENSMUSP00000025694 | 102123\_at | E |
| ENSMUSP00000001983 | 92582\_at | U |
| ENSMUSP00000028962 | 97717\_at | Z |
| ENSMUSP00000032732 | 92727\_at | Z |
| ENSMUSP00000027736 | 103978\_at | E |
| ENSMUSP00000045536 | 160146\_r\_at | U |
| ENSMUSP00000037302 | 95471\_at | Z |
| ENSMUSP00000060544 | 94027\_at | M |
| ENSMUSP00000056785 | 160167\_at | E |
| ENSMUSP00000020159 | 95616\_at | E |
| ENSMUSP00000035195 | 100022\_at | Z |
| ENSMUSP00000035192 | 95749\_at | Z |
| ENSMUSP00000003628 | 100155\_at | Z |
| ENSMUSP00000035914 | 98360\_at | U |
| ENSMUSP00000040286 | 98493\_at | M |
| ENSMUSP00000044335 | 104500\_at | Z |
| ENSMUSP00000034352 | 93515\_at | Z |
| ENSMUSP00000055949 | 92991\_at | Z |
| ENSMUSP00000033054 | 102798\_at | M |
| ENSMUSP00000025554 | 94291\_at | M |
| ENSMUSP00000041216 | 160310\_at | E |
| ENSMUSP00000019128 | 94303\_at | E |
| ENSMUSP00000058490 | 99426\_at | Z |
| ENSMUSP00000050692 | 99559\_at | U |
| ENSMUSP00000031472 | 104098\_at | E |
| ENSMUSP00000001716 | 100564\_at | E |
| ENSMUSP00000037289 | 93593\_f\_at | M |
| ENSMUSP00000035265 | 97325\_at | E |
| ENSMUSP00000021885 | 100709\_at | M |
| ENSMUSP00000002073 | 92335\_at | Z |
| ENSMUSP00000020602 | 102009\_at | E |
| ENSMUSP00000034406 | 92468\_at | U |
| ENSMUSP00000041022 | 96946\_at | E |
| ENSMUSP00000026667 | 101485\_at | U |
| ENSMUSP00000025850 | 99835\_at | Z |
| ENSMUSP00000033919 | 94712\_at | Z |
| ENSMUSP00000021506 | 104374\_at | U |
| ENSMUSP00000046530 | 104519\_at | U |
| ENSMUSP00000058784 | 96145\_at | E |
| ENSMUSP00000023810 | 96278\_at | M |
| ENSMUSP00000028607 | 92611\_at | E |
| ENSMUSP00000016338 | 97867\_at | U |
| ENSMUSP00000037719 | 92877\_at | U |
| ENSMUSP00000048592 | 102418\_at | M |
| ENSMUSP00000034181 | 99034\_at | E |
| ENSMUSP00000056900 | 103995\_at | M |
| ENSMUSP00000034844 | 99167\_at | Z |
| ENSMUSP00000001619 | 160184\_at | E |
| ENSMUSP00000022429 | 160082\_s\_at | E |
| ENSMUSP00000027989 | 94177\_at | U |
| ENSMUSP00000031359 | 100317\_at | U |
| ENSMUSP00000029350 | 101906\_at | U |
| ENSMUSP00000032812 | 98522\_at | E |
| ENSMUSP00000028507 | 103061\_at | U |
| ENSMUSP00000050074 | 97199\_at | E |
| ENSMUSP00000004964 | 98788\_at | E |
| ENSMUSP00000024099 | 104650\_at | U |
| ENSMUSP00000024738 | 96421\_at | E |
| ENSMUSP00000052721 | 95098\_at | E |
| ENSMUSP00000029665 | 161117\_at | M |
| ENSMUSP00000025762 | 101105\_at | Z |
| ENSMUSP00000030420 | 102682\_at | Z |
| ENSMUSP00000020281 | 102262\_r\_at | U |
| ENSMUSP00000012348 | 93009\_at | E |
| ENSMUSP00000002655 | 94453\_at | Z |
| ENSMUSP00000020580 | 160738\_at | E |
| ENSMUSP00000005185 | 100581\_at | M |
| ENSMUSP00000028054 | 97342\_at | U |
| ENSMUSP00000028921 | 97475\_at | E |
| ENSMUSP00000037110 | 92352\_at | Z |
| ENSMUSP00000024049 | 92485\_at | Z |
| ENSMUSP00000024642 | 93941\_at | Z |
| ENSMUSP00000026956 | 103470\_at | M |
| ENSMUSP00000045549 | 103748\_at | E |
| ENSMUSP00000031495 | 160120\_i\_at | Z |
| ENSMUSP00000052750 | 100058\_at | M |
| ENSMUSP00000030660 | 101514\_at | E |
| ENSMUSP00000053011 | 98130\_at | U |
| ENSMUSP00000033691 | 93140\_at | M |
| ENSMUSP00000027017 | 93273\_at | Z |
| ENSMUSP00000025944 | 98408\_at | E |
| ENSMUSP00000020484 | 99985\_at | U |
| ENSMUSP00000002874 | 94995\_at | U |
| ENSMUSP00000025453 | 104536\_at | Z |
| ENSMUSP00000026571 | 104669\_at | M |
| ENSMUSP00000045925 | 96162\_at | M |
| ENSMUSP00000025542 | 96295\_at | U |
| ENSMUSP00000023251 | 101487\_f\_at | M |
| ENSMUSP00000032840 | 97884\_at | U |
| ENSMUSP00000000329 | 102290\_at | E |
| ENSMUSP00000001046 | 99051\_at | M |
| ENSMUSP00000031749 | 102568\_at | E |
| ENSMUSP00000006523 | 94061\_at | E |
| ENSMUSP00000023805 | 99184\_at | U |
| ENSMUSP00000023863 | 160213\_at | E |
| ENSMUSP00000004389 | 94206\_at | Z |
| ENSMUSP00000023850 | 99329\_at | U |
| ENSMUSP00000060656 | 95650\_at | U |
| ENSMUSP00000019183 | 160346\_at | Z |
| ENSMUSP00000001126 | 94339\_at | U |
| ENSMUSP00000028610 | 160479\_at | U |
| ENSMUSP00000062947 | 160905\_s\_at | M |
| ENSMUSP00000055667 | 101923\_at | U |
| ENSMUSP00000043613 | 93682\_at | Z |
| ENSMUSP00000022287 | 98817\_at | Z |
| ENSMUSP00000030286 | 103356\_at | E |
| ENSMUSP00000051484 | 103489\_at | M |
| ENSMUSP00000050156 | 96849\_at | E |
| ENSMUSP00000058677 | 101388\_at | U |
| ENSMUSP00000029589 | 98016\_at | Z |
| ENSMUSP00000029123 | 102977\_at | M |
| ENSMUSP00000001027 | 104011\_at | U |
| ENSMUSP00000050864 | 99593\_at | Z |
| ENSMUSP00000008684 | 93026\_at | Z |
| ENSMUSP00000024748 | 104144\_at | U |
| ENSMUSP00000035569 | 160622\_at | U |
| ENSMUSP00000043580 | 104277\_at | U |
| ENSMUSP00000020001 | 160888\_at | E |
| ENSMUSP00000055342 | 100743\_at | E |
| ENSMUSP00000034630 | 100876\_at | Z |
| ENSMUSP00000030296 | 97492\_at | E |
| ENSMUSP00000006123 | 102043\_at | M |
| ENSMUSP00000021813 | 92514\_at | Z |
| ENSMUSP00000059625 | 103632\_at | E |
| ENSMUSP00000030794 | 103765\_at | Z |
| ENSMUSP00000048711 | 96980\_at | E |
| ENSMUSP00000030427 | 95536\_at | E |
| ENSMUSP00000029881 | 98280\_at | E |
| ENSMUSP00000029722 | 101664\_at | U |
| ENSMUSP00000050469 | 101809\_at | M |
| ENSMUSP00000043926 | 93290\_at | U |
| ENSMUSP00000024826 | 93302\_at | M |
| ENSMUSP00000018610 | 104420\_at | U |
| ENSMUSP00000047954 | 93435\_at | U |
| ENSMUSP00000025570 | 103097\_at | E |
| ENSMUSP00000049689 | 101008\_at | E |
| ENSMUSP00000045715 | 99553\_f\_at | M |
| ENSMUSP00000053119 | 92779\_f\_at | M |
| ENSMUSP00000021636 | 94223\_at | E |
| ENSMUSP00000006366 | 99479\_at | Z |
| ENSMUSP00000009102 | 160363\_at | E |
| ENSMUSP00000060765 | 94489\_at | E |
| ENSMUSP00000023265 | 160508\_at | M |
| ENSMUSP00000015394 | 100484\_at | E |
| ENSMUSP00000005751 | 97112\_at | M |
| ENSMUSP00000019965 | 101940\_at | E |
| ENSMUSP00000055177 | 92255\_at | Z |
| ENSMUSP00000044144 | 92388\_at | U |
| ENSMUSP00000020024 | 98967\_at | Z |
| ENSMUSP00000053145 | 93844\_at | E |
| ENSMUSP00000055382 | 95011\_at | Z |
| ENSMUSP00000031625 | 95144\_at | E |
| ENSMUSP00000029796 | 96733\_at | E |
| ENSMUSP00000018965 | 96866\_at | M |
| ENSMUSP00000046474 | 96999\_at | M |
| ENSMUSP00000032336 | 98033\_at | Z |
| ENSMUSP00000056082 | 102861\_at | U |
| ENSMUSP00000027277 | 102994\_at | Z |
| ENSMUSP00000034887 | 93043\_at | Z |
| ENSMUSP00000003116 | 99622\_at | Z |
| ENSMUSP00000000003 | 94632\_at | M |
| ENSMUSP00000022855 | 160266\_r\_at | E |
| ENSMUSP00000063022 | 99888\_at | Z |
| ENSMUSP00000025851 | 104306\_at | E |
| ENSMUSP00000010209 | 104439\_at | Z |
| ENSMUSP00000042303 | 100760\_at | Z |
| ENSMUSP00000060732 | 96065\_at | M |
| ENSMUSP00000020258 | 100905\_at | E |
| ENSMUSP00000047592 | 102060\_at | E |
| ENSMUSP00000030922 | 96198\_at | E |
| ENSMUSP00000048520 | 103782\_at | U |
| ENSMUSP00000038005 | 102338\_at | E |
| ENSMUSP00000004478 | 92797\_at | E |
| ENSMUSP00000055509 | 95420\_at | U |
| ENSMUSP00000039003 | 94109\_at | Z |
| ENSMUSP00000026474 | 94097\_at | E |
| ENSMUSP00000048607 | 93452\_at | M |
| ENSMUSP00000052872 | 98575\_at | U |
| ENSMUSP00000003137 | 93585\_at | E |
| ENSMUSP00000031205 | 103259\_at | Z |
| ENSMUSP00000029645 | 99269\_g\_at | U |
| ENSMUSP00000030143 | 104715\_at | Z |
| ENSMUSP00000030622 | 96341\_at | E |
| ENSMUSP00000034148 | 161037\_at | M |
| ENSMUSP00000033722 | 101883\_s\_at | M |
| ENSMUSP00000003319 | 96619\_at | U |
| ENSMUSP00000010319 | 102614\_at | Z |
| ENSMUSP00000024745 | 102747\_at | E |
| ENSMUSP00000040094 | 160380\_at | E |
| ENSMUSP00000028209 | 94373\_at | E |
| ENSMUSP00000039127 | 99508\_at | M |
| ENSMUSP00000035586 | 94518\_at | M |
| ENSMUSP00000023008 | 100513\_at | E |
| ENSMUSP00000004407 | 100779\_at | M |
| ENSMUSP00000025642 | 97395\_at | U |
| ENSMUSP00000040220 | 97407\_at | E |
| ENSMUSP00000041694 | 98851\_at | E |
| ENSMUSP00000045650 | 103402\_at | M |
| ENSMUSP00000021043 | 94166\_g\_at | M |
| ENSMUSP00000047611 | 95161\_at | E |
| ENSMUSP00000027534 | 96750\_at | E |
| ENSMUSP00000046920 | 95306\_at | Z |
| ENSMUSP00000004206 | 96883\_at | U |
| ENSMUSP00000029325 | 95439\_at | U |
| ENSMUSP00000062256 | 93193\_at | Z |
| ENSMUSP00000021346 | 98328\_at | Z |
| ENSMUSP00000056352 | 160801\_at | E |
| ENSMUSP00000031698 | 99917\_at | U |
| ENSMUSP00000032582 | 104589\_at | E |
| ENSMUSP00000027256 | 96082\_at | Z |
| ENSMUSP00000044033 | 96227\_at | U |
| ENSMUSP00000053369 | 92681\_at | Z |
| ENSMUSP00000061539 | 102222\_at | Z |
| ENSMUSP00000010752 | 97816\_at | E |
| ENSMUSP00000053718 | 92826\_at | Z |
| ENSMUSP00000046131 | 97949\_at | Z |
| ENSMUSP00000062742 | 92959\_at | Z |
| ENSMUSP00000029429 | 94126\_at | Z |
| ENSMUSP00000058112 | 95715\_at | E |
| ENSMUSP00000037283 | 94259\_at | E |
| ENSMUSP00000027020 | 101710\_at | U |
| ENSMUSP00000061902 | 101843\_at | Z |
| ENSMUSP00000048416 | 101976\_at | E |
| ENSMUSP00000038431 | 98604\_at | E |
| ENSMUSP00000029946 | 93614\_at | E |
| ENSMUSP00000022217 | 96491\_at | E |
| ENSMUSP00000055503 | 96636\_at | U |
| ENSMUSP00000025242 | 101175\_at | E |
| ENSMUSP00000032742 | 102631\_at | U |
| ENSMUSP00000059448 | 102764\_at | M |
| ENSMUSP00000041702 | 99380\_at | U |
| ENSMUSP00000002572 | 104064\_at | Z |
| ENSMUSP00000059521 | 94535\_at | U |
| ENSMUSP00000028170 | 100530\_at | E |
| ENSMUSP00000041503 | 97424\_at | Z |
| ENSMUSP00000009707 | 97557\_at | Z |
| ENSMUSP00000040001 | 92434\_at | E |
| ENSMUSP00000036403 | 104635\_r\_at | M |
| ENSMUSP00000027205 | 92567\_at | Z |
| ENSMUSP00000047569 | 160835\_i\_at | M |
| ENSMUSP00000061234 | 100007\_at | Z |
| ENSMUSP00000002335 | 101451\_at | M |
| ENSMUSP00000028059 | 101584\_at | U |
| ENSMUSP00000057751 | 98144\_f\_at | E |
| ENSMUSP00000062552 | 101729\_at | M |
| ENSMUSP00000039653 | 99801\_at | Z |
| ENSMUSP00000031331 | 98478\_at | E |
| ENSMUSP00000025446 | 104340\_at | Z |
| ENSMUSP00000022459 | 161064\_f\_at | E |
| ENSMUSP00000025931 | 103029\_at | E |
| ENSMUSP00000045572 | 160951\_at | U |
| ENSMUSP00000031131 | 96244\_at | E |
| ENSMUSP00000022166 | 92710\_at | E |
| ENSMUSP00000031220 | 102372\_at | M |
| ENSMUSP00000046388 | 97966\_at | Z |
| ENSMUSP00000001060 | 99000\_at | E |
| ENSMUSP00000046692 | 92976\_at | M |
| ENSMUSP00000010239 | 99133\_at | U |
| ENSMUSP00000005849 | 99399\_at | M |
| ENSMUSP00000028619 | 94276\_at | U |
| ENSMUSP00000061665 | 95732\_at | E |
| ENSMUSP00000043806 | 100416\_at | E |
| ENSMUSP00000042369 | 101860\_at | E |
| ENSMUSP00000020531 | 100549\_at | M |
| ENSMUSP00000030056 | 101993\_at | Z |
| ENSMUSP00000054512 | 99036\_s\_at | E |
| ENSMUSP00000042073 | 98621\_at | E |
| ENSMUSP00000006181 | 98887\_at | E |
| ENSMUSP00000010536 | 103293\_at | E |
| ENSMUSP00000036453 | 93764\_at | E |
| ENSMUSP00000021107 | 103305\_at | Z |
| ENSMUSP00000020566 | 93897\_at | Z |
| ENSMUSP00000037348 | 95064\_at | U |
| ENSMUSP00000031591 | 101192\_at | Z |
| ENSMUSP00000030945 | 102781\_at | E |
| ENSMUSP00000031937 | 101337\_at | Z |
| ENSMUSP00000025224 | 102926\_at | M |
| ENSMUSP00000060774 | 99542\_at | U |
| ENSMUSP00000037018 | 93096\_at | Z |
| ENSMUSP00000025811 | 99675\_at | E |
| ENSMUSP00000044342 | 160704\_at | Z |
| ENSMUSP00000022794 | 97441\_at | E |
| ENSMUSP00000005964 | 98625\_s\_at | U |
| ENSMUSP00000035203 | 97719\_at | Z |
| ENSMUSP00000059559 | 92584\_at | E |
| ENSMUSP00000034880 | 102258\_at | M |
| ENSMUSP00000019896 | 103714\_at | U |
| ENSMUSP00000034211 | 95340\_at | M |
| ENSMUSP00000005651 | 99019\_at | U |
| ENSMUSP00000030312 | 160169\_at | U |
| ENSMUSP00000031328 | 100024\_at | M |
| ENSMUSP00000016639 | 98495\_at | Z |
| ENSMUSP00000029795 | 99951\_at | Z |
| ENSMUSP00000017381 | 98507\_at | Z |
| ENSMUSP00000001181 | 93517\_at | Z |
| ENSMUSP00000000822 | 103046\_at | U |
| ENSMUSP00000055238 | 102401\_at | M |
| ENSMUSP00000023943 | 101078\_at | Z |
| ENSMUSP00000021079 | 99150\_at | U |
| ENSMUSP00000010044 | 102667\_at | Z |
| ENSMUSP00000063146 | 97061\_g\_at | E |
| ENSMUSP00000035007 | 160312\_at | M |
| ENSMUSP00000044774 | 160445\_at | M |
| ENSMUSP00000015484 | 100300\_at | E |
| ENSMUSP00000059801 | 94438\_at | U |
| ENSMUSP00000051211 | 160712\_r\_at | M |
| ENSMUSP00000029866 | 97182\_at | E |
| ENSMUSP00000027377 | 100566\_at | Z |
| ENSMUSP00000032965 | 100699\_at | Z |
| ENSMUSP00000025651 | 97327\_at | U |
| ENSMUSP00000023282 | 93781\_at | E |
| ENSMUSP00000029676 | 103588\_at | E |
| ENSMUSP00000031897 | 96670\_at | Z |
| ENSMUSP00000024739 | 95359\_at | U |
| ENSMUSP00000015950 | 96948\_at | U |
| ENSMUSP00000032457 | 102943\_at | U |
| ENSMUSP00000045184 | 104110\_at | Z |
| ENSMUSP00000034641 | 93258\_at | U |
| ENSMUSP00000005928 | 94714\_at | Z |
| ENSMUSP00000046515 | 160721\_at | Z |
| ENSMUSP00000050846 | 104376\_at | U |
| ENSMUSP00000040307 | 160854\_at | E |
| ENSMUSP00000030672 | 96014\_at | E |
| ENSMUSP00000032974 | 98126\_s\_at | U |
| ENSMUSP00000029386 | 97869\_at | U |
| ENSMUSP00000033718 | 102275\_at | M |
| ENSMUSP00000054510 | 92746\_at | U |
| ENSMUSP00000031032 | 92879\_at | E |
| ENSMUSP00000006397 | 103997\_at | M |
| ENSMUSP00000034703 | 99169\_at | E |
| ENSMUSP00000002855 | 95490\_at | E |
| ENSMUSP00000034863 | 94046\_at | E |
| ENSMUSP00000050347 | 95502\_at | U |
| ENSMUSP00000018821 | 100041\_at | E |
| ENSMUSP00000039583 | 101896\_at | M |
| ENSMUSP00000056226 | 103063\_at | M |
| ENSMUSP00000020087 | 93534\_at | U |
| ENSMUSP00000048541 | 103208\_at | U |
| ENSMUSP00000029727 | 93667\_at | U |
| ENSMUSP00000039920 | 104652\_at | E |
| ENSMUSP00000014747 | 96423\_at | Z |
| ENSMUSP00000030759 | 101095\_at | M |
| ENSMUSP00000031779 | 101107\_at | E |
| ENSMUSP00000031216 | 102684\_at | M |
| ENSMUSP00000022977 | 94322\_at | U |
| ENSMUSP00000034697 | 99445\_at | E |
| ENSMUSP00000044178 | 94455\_at | U |
| ENSMUSP00000023104 | 160595\_at | U |
| ENSMUSP00000019744 | 104129\_at | U |
| ENSMUSP00000060507 | 160607\_at | Z |
| ENSMUSP00000035075 | 93002\_r\_at | Z |
| ENSMUSP00000005914 | 100583\_at | M |
| ENSMUSP00000049147 | 97211\_at | E |
| ENSMUSP00000030107 | 100728\_at | Z |
| ENSMUSP00000027405 | 98800\_at | U |
| ENSMUSP00000039335 | 97477\_at | E |
| ENSMUSP00000008035 | 93810\_at | E |
| ENSMUSP00000043755 | 98933\_at | E |
| ENSMUSP00000047827 | 92487\_at | E |
| ENSMUSP00000027650 | 103617\_at | Z |
| ENSMUSP00000028087 | 160821\_r\_at | U |
| ENSMUSP00000052809 | 95376\_at | U |
| ENSMUSP00000031629 | 101371\_at | E |
| ENSMUSP00000048041 | 101516\_at | M |
| ENSMUSP00000053484 | 98132\_at | U |
| ENSMUSP00000029565 | 102960\_at | E |
| ENSMUSP00000026703 | 93142\_at | Z |
| ENSMUSP00000003268 | 93275\_at | E |
| ENSMUSP00000034359 | 160689\_r\_at | M |
| ENSMUSP00000035083 | 94731\_at | E |
| ENSMUSP00000019918 | 99854\_at | E |
| ENSMUSP00000030804 | 160871\_at | E |
| ENSMUSP00000018113 | 104538\_at | U |
| ENSMUSP00000045877 | 100992\_at | Z |
| ENSMUSP00000030501 | 96297\_at | E |
| ENSMUSP00000044034 | 102292\_at | E |
| ENSMUSP00000048111 | 97886\_at | U |
| ENSMUSP00000001055 | 99053\_at | M |
| ENSMUSP00000029644 | 103881\_at | U |
| ENSMUSP00000021795 | 92908\_at | Z |
| ENSMUSP00000029700 | 94063\_at | Z |
| ENSMUSP00000026081 | 160070\_at | Z |
| ENSMUSP00000029270 | 99186\_at | E |
| ENSMUSP00000039692 | 95652\_at | Z |
| ENSMUSP00000002518 | 160215\_at | Z |
| ENSMUSP00000004330 | 94196\_at | M |
| ENSMUSP00000002445 | 160348\_at | E |
| ENSMUSP00000024793 | 100469\_at | E |
| ENSMUSP00000043204 | 93551\_at | U |
| ENSMUSP00000059717 | 103080\_at | U |
| ENSMUSP00000062878 | 94138\_s\_at | E |
| ENSMUSP00000052209 | 103225\_at | M |
| ENSMUSP00000028977 | 161003\_at | E |
| ENSMUSP00000047446 | 96573\_at | E |
| ENSMUSP00000025885 | 96718\_at | Z |
| ENSMUSP00000062032 | 98018\_at | M |
| ENSMUSP00000045135 | 102979\_at | Z |
| ENSMUSP00000017629 | 99462\_at | U |
| ENSMUSP00000000031 | 93028\_at | M |
| ENSMUSP00000038744 | 99607\_at | E |
| ENSMUSP00000003362 | 104146\_at | M |
| ENSMUSP00000052402 | 97001\_r\_at | M |
| ENSMUSP00000033283 | 100612\_at | U |
| ENSMUSP00000043501 | 92371\_at | M |
| ENSMUSP00000030399 | 98950\_at | E |
| ENSMUSP00000059404 | 103501\_at | E |
| ENSMUSP00000025509 | 92516\_at | Z |
| ENSMUSP00000062506 | 103634\_at | M |
| ENSMUSP00000055217 | 102337\_s\_at | Z |
| ENSMUSP00000002027 | 92649\_at | E |
| ENSMUSP00000033824 | 160089\_at | Z |
| ENSMUSP00000039986 | 95405\_at | Z |
| ENSMUSP00000028739 | 95393\_at | E |
| ENSMUSP00000057157 | 160917\_r\_at | Z |
| ENSMUSP00000059764 | 98282\_at | M |
| ENSMUSP00000024944 | 93304\_at | U |
| ENSMUSP00000022035 | 160900\_at | M |
| ENSMUSP00000049679 | 104555\_at | M |
| ENSMUSP00000043739 | 104688\_at | E |
| ENSMUSP00000001720 | 96326\_at | U |
| ENSMUSP00000003448 | 102321\_at | U |
| ENSMUSP00000027297 | 97915\_at | M |
| ENSMUSP00000026217 | 99070\_at | U |
| ENSMUSP00000033183 | 103910\_at | E |
| ENSMUSP00000022062 | 94080\_at | U |
| ENSMUSP00000044769 | 94225\_at | E |
| ENSMUSP00000029121 | 160365\_at | U |
| ENSMUSP00000026252 | 160498\_at | Z |
| ENSMUSP00000030122 | 94358\_at | Z |
| ENSMUSP00000007116 | 100486\_at | U |
| ENSMUSP00000004316 | 97114\_at | E |
| ENSMUSP00000047037 | 101942\_at | E |
| ENSMUSP00000025503 | 97247\_at | U |
| ENSMUSP00000031148 | 92257\_at | Z |
| ENSMUSP00000027507 | 98836\_at | M |
| ENSMUSP00000002084 | 98969\_at | U |
| ENSMUSP00000035744 | 93846\_at | M |
| ENSMUSP00000033933 | 95146\_at | Z |
| ENSMUSP00000032927 | 96735\_at | Z |
| ENSMUSP00000039472 | 96868\_at | Z |
| ENSMUSP00000003630 | 101419\_at | E |
| ENSMUSP00000063026 | 102863\_at | Z |
| ENSMUSP00000008028 | 102996\_at | Z |
| ENSMUSP00000029770 | 93045\_at | U |
| ENSMUSP00000021450 | 94501\_at | U |
| ENSMUSP00000046759 | 104163\_at | E |
| ENSMUSP00000027477 | 93178\_at | E |
| ENSMUSP00000020278 | 94634\_at | Z |
| ENSMUSP00000053355 | 160774\_at | E |
| ENSMUSP00000033053 | 104308\_at | Z |
| ENSMUSP00000054848 | 100762\_at | Z |
| ENSMUSP00000023190 | 100895\_at | U |
| ENSMUSP00000060152 | 100907\_at | M |
| ENSMUSP00000040227 | 92400\_at | Z |
| ENSMUSP00000035057 | 102062\_at | E |
| ENSMUSP00000032907 | 92533\_at | M |
| ENSMUSP00000036591 | 97789\_at | E |
| ENSMUSP00000052181 | 92666\_at | E |
| ENSMUSP00000019516 | 102207\_at | E |
| ENSMUSP00000028854 | 99089\_at | Z |
| ENSMUSP00000004354 | 94099\_at | E |
| ENSMUSP00000000983 | 160118\_at | E |
| ENSMUSP00000002127 | 95555\_at | Z |
| ENSMUSP00000003527 | 100094\_at | E |
| ENSMUSP00000048519 | 95688\_at | E |
| ENSMUSP00000024827 | 100106\_at | M |
| ENSMUSP00000058345 | 103651\_r\_at | Z |
| ENSMUSP00000032201 | 101828\_at | Z |
| ENSMUSP00000043436 | 99900\_at | Z |
| ENSMUSP00000042071 | 93321\_at | M |
| ENSMUSP00000028923 | 93454\_at | Z |
| ENSMUSP00000023359 | 94910\_at | Z |
| ENSMUSP00000045290 | 104572\_at | E |
| ENSMUSP00000022352 | 93587\_at | E |
| ENSMUSP00000005685 | 102847\_s\_at | E |
| ENSMUSP00000038047 | 104717\_at | M |
| ENSMUSP00000039606 | 96343\_at | E |
| ENSMUSP00000055000 | 161039\_at | E |
| ENSMUSP00000029909 | 99365\_at | U |
| ENSMUSP00000021158 | 94242\_at | E |
| ENSMUSP00000018184 | 160382\_at | E |
| ENSMUSP00000000642 | 94375\_at | E |
| ENSMUSP00000057815 | 92830\_s\_at | E |
| ENSMUSP00000035870 | 100370\_at | U |
| ENSMUSP00000027414 | 97131\_at | U |
| ENSMUSP00000032744 | 100515\_at | U |
| ENSMUSP00000048001 | 97397\_at | U |
| ENSMUSP00000018066 | 92274\_at | M |
| ENSMUSP00000058606 | 93730\_at | U |
| ENSMUSP00000028353 | 98853\_at | E |
| ENSMUSP00000050446 | 97409\_at | M |
| ENSMUSP00000021040 | 92419\_at | U |
| ENSMUSP00000034081 | 103392\_at | U |
| ENSMUSP00000026552 | 93996\_at | E |
| ENSMUSP00000005400 | 95030\_at | M |
| ENSMUSP00000002398 | 95308\_at | U |
| ENSMUSP00000019617 | 96752\_at | M |
| ENSMUSP00000031365 | 101436\_at | M |
| ENSMUSP00000026557 | 98052\_at | E |
| ENSMUSP00000023710 | 101569\_at | U |
| ENSMUSP00000023607 | 93062\_at | U |
| ENSMUSP00000017878 | 99641\_at | M |
| ENSMUSP00000018156 | 104180\_at | E |
| ENSMUSP00000023295 | 93207\_at | Z |
| ENSMUSP00000009776 | 160803\_at | E |
| ENSMUSP00000043890 | 104325\_at | M |
| ENSMUSP00000029053 | 94929\_at | E |
| ENSMUSP00000015763 | 100924\_at | E |
| ENSMUSP00000007317 | 92550\_at | E |
| ENSMUSP00000058277 | 102224\_at | Z |
| ENSMUSP00000034602 | 92683\_at | M |
| ENSMUSP00000023502 | 97818\_at | E |
| ENSMUSP00000029055 | 92828\_at | U |
| ENSMUSP00000020997 | 103813\_at | U |
| ENSMUSP00000020681 | 160772\_i\_at | E |
| ENSMUSP00000023432 | 160135\_at | U |
| ENSMUSP00000027393 | 94128\_at | U |
| ENSMUSP00000027247 | 160268\_at | E |
| ENSMUSP00000022609 | 95717\_at | U |
| ENSMUSP00000031425 | 101712\_at | M |
| ENSMUSP00000022441 | 100389\_at | M |
| ENSMUSP00000028494 | 101978\_at | E |
| ENSMUSP00000026381 | 103278\_at | M |
| ENSMUSP00000026013 | 93749\_at | U |
| ENSMUSP00000026124 | 96360\_at | E |
| ENSMUSP00000037597 | 95049\_at | E |
| ENSMUSP00000055546 | 96505\_at | M |
| ENSMUSP00000030090 | 101044\_at | U |
| ENSMUSP00000002848 | 99382\_at | U |
| ENSMUSP00000005103 | 99527\_at | Z |
| ENSMUSP00000015891 | 94404\_at | E |
| ENSMUSP00000025094 | 104066\_at | M |
| ENSMUSP00000029046 | 160544\_at | Z |
| ENSMUSP00000049791 | 104199\_at | E |
| ENSMUSP00000042364 | 94537\_at | E |
| ENSMUSP00000020360 | 92303\_at | E |
| ENSMUSP00000032330 | 97426\_at | M |
| ENSMUSP00000002081 | 92436\_at | U |
| ENSMUSP00000046101 | 97559\_at | U |
| ENSMUSP00000039487 | 103421\_at | E |
| ENSMUSP00000011400 | 103554\_at | E |
| ENSMUSP00000017276 | 95325\_at | E |
| ENSMUSP00000003863 | 101453\_at | M |
| ENSMUSP00000033689 | 98347\_at | Z |
| ENSMUSP00000055056 | 99803\_at | M |
| ENSMUSP00000041232 | 160820\_at | M |
| ENSMUSP00000051651 | 94813\_at | Z |
| ENSMUSP00000025463 | 96113\_at | U |
| ENSMUSP00000009425 | 97835\_at | M |
| ENSMUSP00000045146 | 92712\_at | Z |
| ENSMUSP00000050581 | 103830\_at | Z |
| ENSMUSP00000022792 | 92845\_at | U |
| ENSMUSP00000019615 | 99135\_at | E |
| ENSMUSP00000056720 | 94145\_at | M |
| ENSMUSP00000021595 | 160152\_at | U |
| ENSMUSP00000018569 | 160285\_at | U |
| ENSMUSP00000022575 | 94278\_at | E |
| ENSMUSP00000055256 | 100418\_at | Z |
| ENSMUSP00000045569 | 101862\_at | E |
| ENSMUSP00000015981 | 101995\_at | E |
| ENSMUSP00000043320 | 93500\_at | U |
| ENSMUSP00000062221 | 98942\_r\_at | U |
| ENSMUSP00000033127 | 93633\_at | E |
| ENSMUSP00000049303 | 104751\_at | Z |
| ENSMUSP00000023832 | 93766\_at | U |
| ENSMUSP00000053188 | 93899\_at | E |
| ENSMUSP00000026576 | 95066\_at | U |
| ENSMUSP00000045456 | 101061\_at | E |
| ENSMUSP00000031876 | 101194\_at | M |
| ENSMUSP00000025020 | 102650\_at | Z |
| ENSMUSP00000041271 | 102783\_at | U |
| ENSMUSP00000050898 | 99411\_at | Z |
| ENSMUSP00000056669 | 98088\_at | E |
| ENSMUSP00000034339 | 104083\_at | Z |
| ENSMUSP00000028672 | 160561\_at | M |
| ENSMUSP00000036541 | 94554\_at | E |
| ENSMUSP00000057176 | 99677\_at | M |
| ENSMUSP00000023687 | 160694\_at | M |
| ENSMUSP00000062051 | 94687\_at | E |
| ENSMUSP00000030826 | 160839\_at | U |
| ENSMUSP00000010550 | 97443\_at | Z |
| ENSMUSP00000022322 | 92586\_at | U |
| ENSMUSP00000006293 | 103849\_at | E |
| ENSMUSP00000052123 | 95342\_at | M |
| ENSMUSP00000032402 | 100026\_at | U |
| ENSMUSP00000037588 | 101470\_at | Z |
| ENSMUSP00000045335 | 101748\_at | Z |
| ENSMUSP00000026357 | 93374\_at | M |
| ENSMUSP00000006439 | 98497\_at | E |
| ENSMUSP00000041082 | 99953\_at | E |
| ENSMUSP00000059669 | 98509\_at | M |
| ENSMUSP00000019276 | 94830\_at | M |
| ENSMUSP00000045993 | 103048\_at | E |
| ENSMUSP00000033378 | 104492\_at | Z |
| ENSMUSP00000024572 | 96263\_at | Z |
| ENSMUSP00000000028 | 102403\_at | E |
| ENSMUSP00000019149 | 92995\_at | E |
| ENSMUSP00000006614 | 103980\_at | Z |
| ENSMUSP00000028794 | 102669\_at | Z |
| ENSMUSP00000047661 | 99631\_f\_at | E |
| ENSMUSP00000020286 | 160314\_at | U |
| ENSMUSP00000029570 | 160447\_at | U |
| ENSMUSP00000036570 | 100302\_at | Z |
| ENSMUSP00000052581 | 100435\_at | Z |
| ENSMUSP00000034105 | 100568\_at | U |
| ENSMUSP00000029542 | 97329\_at | Z |
| ENSMUSP00000023775 | 93783\_at | Z |
| ENSMUSP00000041839 | 98918\_at | U |
| ENSMUSP00000019986 | 103457\_at | U |
| ENSMUSP00000054463 | 96672\_at | Z |
| ENSMUSP00000053616 | 101356\_at | U |
| ENSMUSP00000050414 | 94571\_at | M |
| ENSMUSP00000032461 | 160723\_at | E |
| ENSMUSP00000024035 | 99839\_at | Z |
| ENSMUSP00000062670 | 104378\_at | Z |
| ENSMUSP00000015434 | 160856\_at | E |
| ENSMUSP00000038813 | 97460\_at | E |
| ENSMUSP00000006669 | 100977\_at | U |
| ENSMUSP00000023834 | 102011\_at | U |
| ENSMUSP00000022694 | 103600\_at | Z |
| ENSMUSP00000020550 | 94048\_at | E |
| ENSMUSP00000023006 | 160952\_r\_at | E |
| ENSMUSP00000052020 | 95637\_at | E |
| ENSMUSP00000027559 | 101632\_at | M |
| ENSMUSP00000043588 | 93391\_at | M |
| ENSMUSP00000021397 | 99970\_at | E |
| ENSMUSP00000028880 | 103065\_at | U |
| ENSMUSP00000053917 | 93536\_at | M |
| ENSMUSP00000032071 | 94980\_at | M |
| ENSMUSP00000031725 | 104654\_at | E |
| ENSMUSP00000014438 | 96280\_at | E |
| ENSMUSP00000030449 | 96558\_at | U |
| ENSMUSP00000025745 | 101097\_at | E |
| ENSMUSP00000036680 | 102686\_at | E |
| ENSMUSP00000026976 | 99447\_at | E |
| ENSMUSP00000029677 | 160331\_at | Z |
| ENSMUSP00000028964 | 94457\_at | E |
| ENSMUSP00000055658 | 160597\_at | M |
| ENSMUSP00000019181 | 100452\_at | Z |
| ENSMUSP00000031324 | 98802\_at | M |
| ENSMUSP00000036747 | 92223\_at | M |
| ENSMUSP00000029433 | 92356\_at | E |
| ENSMUSP00000044853 | 97479\_at | E |
| ENSMUSP00000030381 | 103341\_at | U |
| ENSMUSP00000019902 | 93945\_at | M |
| ENSMUSP00000031513 | 96834\_at | E |
| ENSMUSP00000046469 | 98001\_at | E |
| ENSMUSP00000001132 | 101518\_at | Z |
| ENSMUSP00000000497 | 102962\_at | E |
| ENSMUSP00000032264 | 93011\_at | E |
| ENSMUSP00000051433 | 104262\_at | M |
| ENSMUSP00000061684 | 93277\_at | U |
| ENSMUSP00000003717 | 94733\_at | U |
| ENSMUSP00000023312 | 104407\_at | Z |
| ENSMUSP00000020911 | 96033\_at | Z |
| ENSMUSP00000026441 | 100994\_at | Z |
| ENSMUSP00000026569 | 97755\_at | Z |
| ENSMUSP00000033292 | 97888\_at | E |
| ENSMUSP00000043066 | 102306\_at | Z |
| ENSMUSP00000037487 | 92898\_at | U |
| ENSMUSP00000021169 | 99055\_at | E |
| ENSMUSP00000017920 | 94065\_at | E |
| ENSMUSP00000002320 | 94198\_at | Z |
| ENSMUSP00000040840 | 160217\_at | U |
| ENSMUSP00000007257 | 95654\_at | U |
| ENSMUSP00000046641 | 160660\_r\_at | Z |
| ENSMUSP00000056001 | 98410\_at | M |
| ENSMUSP00000015667 | 98543\_at | E |
| ENSMUSP00000041393 | 103082\_at | U |
| ENSMUSP00000031181 | 103227\_at | U |
| ENSMUSP00000005829 | 104671\_at | U |
| ENSMUSP00000022377 | 161005\_at | E |
| ENSMUSP00000004072 | 96575\_at | U |
| ENSMUSP00000045263 | 99331\_at | E |
| ENSMUSP00000052128 | 92624\_r\_at | M |
| ENSMUSP00000037774 | 94341\_at | E |
| ENSMUSP00000059813 | 99464\_at | M |
| ENSMUSP00000029017 | 160481\_at | U |
| ENSMUSP00000029804 | 104015\_at | U |
| ENSMUSP00000023867 | 160759\_at | U |
| ENSMUSP00000044009 | 94619\_at | U |
| ENSMUSP00000019037 | 100614\_at | M |
| ENSMUSP00000059308 | 97363\_at | Z |
| ENSMUSP00000058380 | 92240\_at | M |
| ENSMUSP00000043803 | 103491\_at | Z |
| ENSMUSP00000021314 | 102047\_at | E |
| ENSMUSP00000047721 | 103503\_at | E |
| ENSMUSP00000027370 | 103636\_at | U |
| ENSMUSP00000023156 | 103769\_at | Z |
| ENSMUSP00000045684 | 92480\_f\_at | M |
| ENSMUSP00000062802 | 95395\_at | E |
| ENSMUSP00000020241 | 95407\_at | U |
| ENSMUSP00000021257 | 101402\_at | Z |
| ENSMUSP00000022980 | 100079\_at | E |
| ENSMUSP00000020171 | 93294\_at | Z |
| ENSMUSP00000030429 | 98429\_at | U |
| ENSMUSP00000028981 | 93306\_at | E |
| ENSMUSP00000010736 | 94750\_at | U |
| ENSMUSP00000049898 | 99873\_at | Z |
| ENSMUSP00000005003 | 160890\_at | E |
| ENSMUSP00000022749 | 104424\_at | M |
| ENSMUSP00000053437 | 160902\_at | M |
| ENSMUSP00000051467 | 96053\_i\_at | U |
| ENSMUSP00000062899 | 96050\_at | E |
| ENSMUSP00000022368 | 97772\_at | Z |
| ENSMUSP00000050541 | 102323\_at | Z |
| ENSMUSP00000026405 | 97917\_at | E |
| ENSMUSP00000020124 | 92782\_at | Z |
| ENSMUSP00000055525 | 92927\_at | Z |
| ENSMUSP00000005548 | 160101\_at | U |
| ENSMUSP00000030289 | 160234\_at | E |
| ENSMUSP00000038014 | 95671\_at | E |
| ENSMUSP00000039801 | 101811\_at | Z |
| ENSMUSP00000053422 | 100488\_at | U |
| ENSMUSP00000023044 | 97116\_at | M |
| ENSMUSP00000034218 | 93570\_at | U |
| ENSMUSP00000002681 | 97249\_at | U |
| ENSMUSP00000005821 | 93715\_at | M |
| ENSMUSP00000004587 | 103244\_at | M |
| ENSMUSP00000030583 | 95148\_at | U |
| ENSMUSP00000047004 | 96592\_at | E |
| ENSMUSP00000026151 | 96604\_at | U |
| ENSMUSP00000044827 | 101010\_at | E |
| ENSMUSP00000041825 | 101143\_at | Z |
| ENSMUSP00000031146 | 161032\_i\_at | M |
| ENSMUSP00000022022 | 102865\_at | Z |
| ENSMUSP00000046752 | 98037\_at | Z |
| ENSMUSP00000007326 | 99481\_at | U |
| ENSMUSP00000034860 | 102998\_at | E |
| ENSMUSP00000004904 | 93047\_at | E |
| ENSMUSP00000020523 | 94491\_at | E |
| ENSMUSP00000022803 | 101558\_s\_at | U |
| ENSMUSP00000019911 | 160643\_at | U |
| ENSMUSP00000040599 | 104165\_at | U |
| ENSMUSP00000038630 | 104298\_at | M |
| ENSMUSP00000027835 | 160776\_at | U |
| ENSMUSP00000039170 | 94636\_at | Z |
| ENSMUSP00000018765 | 94769\_at | E |
| ENSMUSP00000055879 | 97380\_at | E |
| ENSMUSP00000006875 | 100764\_at | M |
| ENSMUSP00000061482 | 100909\_at | Z |
| ENSMUSP00000062774 | 96069\_at | U |
| ENSMUSP00000027015 | 102064\_at | Z |
| ENSMUSP00000047945 | 103520\_at | Z |
| ENSMUSP00000032895 | 102197\_at | Z |
| ENSMUSP00000046312 | 102209\_at | Z |
| ENSMUSP00000060102 | 103653\_at | E |
| ENSMUSP00000033617 | 92668\_at | Z |
| ENSMUSP00000020501 | 95424\_at | E |
| ENSMUSP00000059138 | 101552\_at | U |
| ENSMUSP00000000500 | 99890\_at | Z |
| ENSMUSP00000033486 | 93323\_at | Z |
| ENSMUSP00000039559 | 98579\_at | Z |
| ENSMUSP00000006991 | 99902\_at | U |
| ENSMUSP00000015890 | 94912\_at | Z |
| ENSMUSP00000038958 | 104574\_at | E |
| ENSMUSP00000022653 | 93589\_at | E |
| ENSMUSP00000060727 | 104719\_at | U |
| ENSMUSP00000006035 | 96345\_at | E |
| ENSMUSP00000023407 | 102340\_at | U |
| ENSMUSP00000000206 | 161082\_r\_at | Z |
| ENSMUSP00000000430 | 97934\_at | Z |
| ENSMUSP00000035029 | 92811\_at | Z |
| ENSMUSP00000016698 | 99101\_at | E |
| ENSMUSP00000028502 | 160251\_at | E |
| ENSMUSP00000005618 | 160384\_at | U |
| ENSMUSP00000031670 | 94377\_at | Z |
| ENSMUSP00000031119 | 100372\_at | Z |
| ENSMUSP00000015832 | 101961\_at | U |
| ENSMUSP00000030677 | 92276\_at | E |
| ENSMUSP00000052551 | 103261\_at | U |
| ENSMUSP00000059335 | 103394\_at | M |
| ENSMUSP00000041173 | 98988\_at | U |
| ENSMUSP00000031021 | 103406\_at | U |
| ENSMUSP00000006349 | 103539\_at | E |
| ENSMUSP00000043379 | 95032\_at | E |
| ENSMUSP00000061123 | 95165\_at | M |
| ENSMUSP00000003515 | 96621\_at | E |
| ENSMUSP00000031326 | 101160\_at | M |
| ENSMUSP00000052088 | 101305\_at | E |
| ENSMUSP00000033373 | 96887\_at | Z |
| ENSMUSP00000051649 | 101293\_at | U |
| ENSMUSP00000057541 | 102882\_at | M |
| ENSMUSP00000034754 | 93064\_at | E |
| ENSMUSP00000007260 | 93197\_at | M |
| ENSMUSP00000030985 | 104182\_at | Z |
| ENSMUSP00000003908 | 160938\_at | E |
| ENSMUSP00000010038 | 96086\_at | M |
| ENSMUSP00000038158 | 92685\_at | Z |
| ENSMUSP00000049082 | 103670\_at | E |
| ENSMUSP00000047457 | 103948\_at | Z |
| ENSMUSP00000057863 | 95441\_at | E |
| ENSMUSP00000060247 | 160137\_at | E |
| ENSMUSP00000006659 | 101874\_s\_at | M |
| ENSMUSP00000016294 | 101714\_at | Z |
| ENSMUSP00000005446 | 98330\_at | E |
| ENSMUSP00000050902 | 94120\_s\_at | M |
| ENSMUSP00000034707 | 98463\_at | U |
| ENSMUSP00000046324 | 104603\_at | U |
| ENSMUSP00000001867 | 96507\_at | Z |
| ENSMUSP00000018842 | 92961\_at | Z |
| ENSMUSP00000025941 | 102635\_at | E |
| ENSMUSP00000034741 | 93101\_s\_at | E |
| ENSMUSP00000058137 | 94406\_at | Z |
| ENSMUSP00000042150 | 94394\_at | E |
| ENSMUSP00000020537 | 160413\_at | M |
| ENSMUSP00000017534 | 160546\_at | U |
| ENSMUSP00000023835 | 97150\_at | U |
| ENSMUSP00000045230 | 100534\_at | E |
| ENSMUSP00000062832 | 97283\_at | M |
| ENSMUSP00000020942 | 92293\_at | Z |
| ENSMUSP00000047018 | 97428\_at | E |
| ENSMUSP00000050852 | 98872\_at | U |
| ENSMUSP00000004208 | 103556\_at | Z |
| ENSMUSP00000021231 | 103689\_at | U |
| ENSMUSP00000006600 | 95327\_at | E |
| ENSMUSP00000049295 | 96771\_at | Z |
| ENSMUSP00000050973 | 96916\_at | E |
| ENSMUSP00000045216 | 101588\_at | U |
| ENSMUSP00000038576 | 102911\_at | E |
| ENSMUSP00000033720 | 93081\_at | E |
| ENSMUSP00000022276 | 98349\_at | Z |
| ENSMUSP00000001548 | 104211\_at | Z |
| ENSMUSP00000002034 | 99938\_at | E |
| ENSMUSP00000042473 | 160822\_at | E |
| ENSMUSP00000047393 | 94815\_at | U |
| ENSMUSP00000026907 | 104344\_at | Z |
| ENSMUSP00000029038 | 160729\_f\_at | Z |
| ENSMUSP00000024119 | 94948\_at | E |
| ENSMUSP00000004634 | 100943\_at | U |
| ENSMUSP00000051488 | 97692\_at | Z |
| ENSMUSP00000019616 | 92714\_at | Z |
| ENSMUSP00000057199 | 102243\_at | Z |
| ENSMUSP00000044979 | 97496\_f\_at | M |
| ENSMUSP00000031288 | 103832\_at | E |
| ENSMUSP00000031654 | 94014\_at | E |
| ENSMUSP00000039586 | 94147\_at | U |
| ENSMUSP00000025778 | 95603\_at | U |
| ENSMUSP00000003250 | 100142\_at | Z |
| ENSMUSP00000003386 | 95736\_at | U |
| ENSMUSP00000058506 | 101864\_at | E |
| ENSMUSP00000061255 | 93490\_at | E |
| ENSMUSP00000020999 | 93635\_at | E |
| ENSMUSP00000026305 | 103297\_at | E |
| ENSMUSP00000034138 | 95068\_at | U |
| ENSMUSP00000022468 | 101063\_at | E |
| ENSMUSP00000026318 | 96657\_at | U |
| ENSMUSP00000032725 | 101196\_at | Z |
| ENSMUSP00000046714 | 101208\_at | U |
| ENSMUSP00000051527 | 102652\_at | E |
| ENSMUSP00000047576 | 102785\_at | Z |
| ENSMUSP00000026911 | 99413\_at | Z |
| ENSMUSP00000051032 | 94423\_at | M |
| ENSMUSP00000007130 | 160430\_at | E |
| ENSMUSP00000048810 | 104085\_at | M |
| ENSMUSP00000032064 | 160696\_at | E |
| ENSMUSP00000044165 | 94556\_at | E |
| ENSMUSP00000021220 | 92794\_f\_at | U |
| ENSMUSP00000044618 | 160708\_at | Z |
| ENSMUSP00000003493 | 100684\_at | E |
| ENSMUSP00000019958 | 97312\_at | Z |
| ENSMUSP00000021669 | 98901\_at | E |
| ENSMUSP00000035274 | 92322\_at | M |
| ENSMUSP00000029382 | 97445\_at | U |
| ENSMUSP00000017608 | 92455\_at | M |
| ENSMUSP00000054112 | 93911\_at | U |
| ENSMUSP00000025800 | 103573\_at | E |
| ENSMUSP00000018681 | 103718\_at | E |
| ENSMUSP00000033646 | 95344\_at | M |
| ENSMUSP00000030361 | 95477\_at | M |
| ENSMUSP00000033138 | 93091\_s\_at | Z |
| ENSMUSP00000028499 | 98366\_at | Z |
| ENSMUSP00000009143 | 93243\_at | Z |
| ENSMUSP00000038084 | 104361\_at | M |
| ENSMUSP00000050838 | 94832\_at | E |
| ENSMUSP00000046076 | 160972\_at | U |
| ENSMUSP00000033389 | 97721\_at | Z |
| ENSMUSP00000028156 | 102260\_at | Z |
| ENSMUSP00000029421 | 92731\_at | Z |
| ENSMUSP00000054814 | 102393\_at | Z |
| ENSMUSP00000060169 | 97987\_at | U |
| ENSMUSP00000027878 | 99021\_at | E |
| ENSMUSP00000041971 | 102405\_at | Z |
| ENSMUSP00000032421 | 94164\_at | E |
| ENSMUSP00000027097 | 102672\_g\_at | E |
| ENSMUSP00000021512 | 95620\_at | U |
| ENSMUSP00000061503 | 94297\_at | U |
| ENSMUSP00000045737 | 95753\_at | E |
| ENSMUSP00000029709 | 100292\_at | Z |
| ENSMUSP00000013907 | 101083\_s\_at | E |
| ENSMUSP00000054404 | 97053\_at | Z |
| ENSMUSP00000045583 | 92208\_at | M |
| ENSMUSP00000018476 | 98775\_at | E |
| ENSMUSP00000036428 | 103326\_at | E |
| ENSMUSP00000001882 | 93785\_at | M |
| ENSMUSP00000025119 | 103459\_at | U |
| ENSMUSP00000025912 | 101358\_at | E |
| ENSMUSP00000054062 | 99430\_at | Z |
| ENSMUSP00000041186 | 102947\_at | U |
| ENSMUSP00000059396 | 98119\_at | U |
| ENSMUSP00000053251 | 93129\_at | E |
| ENSMUSP00000028789 | 104247\_at | E |
| ENSMUSP00000057308 | 94718\_at | M |
| ENSMUSP00000056586 | 160858\_at | E |
| ENSMUSP00000050417 | 100713\_at | M |
| ENSMUSP00000049471 | 102013\_at | U |
| ENSMUSP00000049986 | 102146\_at | Z |
| ENSMUSP00000035216 | 102279\_at | U |
| ENSMUSP00000044453 | 103602\_at | U |
| ENSMUSP00000006716 | 103735\_at | Z |
| ENSMUSP00000022586 | 103868\_at | Z |
| ENSMUSP00000017918 | 95506\_at | E |
| ENSMUSP00000027470 | 95639\_at | Z |
| ENSMUSP00000039970 | 101634\_at | M |
| ENSMUSP00000016621 | 93405\_at | E |
| ENSMUSP00000030590 | 98528\_at | E |
| ENSMUSP00000037752 | 99972\_at | U |
| ENSMUSP00000061817 | 104523\_at | U |
| ENSMUSP00000035660 | 93538\_at | E |
| ENSMUSP00000031009 | 101099\_at | M |
| ENSMUSP00000039190 | 160200\_at | Z |
| ENSMUSP00000034023 | 94181\_at | M |
| ENSMUSP00000016491 | 99449\_at | U |
| ENSMUSP00000029165 | 160333\_at | E |
| ENSMUSP00000029013 | 160466\_at | U |
| ENSMUSP00000055847 | 94459\_at | M |
| ENSMUSP00000048979 | 95915\_at | M |
| ENSMUSP00000030741 | 100454\_at | E |
| ENSMUSP00000006263 | 103210\_at | Z |
| ENSMUSP00000041999 | 98804\_at | Z |
| ENSMUSP00000011259 | 98937\_at | E |
| ENSMUSP00000053266 | 103343\_at | M |
| ENSMUSP00000060795 | 103476\_at | U |
| ENSMUSP00000025057 | 96691\_at | E |
| ENSMUSP00000026142 | 96703\_at | E |
| ENSMUSP00000063033 | 96969\_at | M |
| ENSMUSP00000019559 | 98003\_at | M |
| ENSMUSP00000029203 | 102964\_at | Z |
| ENSMUSP00000026755 | 98136\_at | U |
| ENSMUSP00000020974 | 93013\_at | Z |
| ENSMUSP00000051887 | 93146\_at | M |
| ENSMUSP00000002950 | 104131\_at | E |
| ENSMUSP00000010974 | 104464\_s\_at | E |
| ENSMUSP00000029721 | 104264\_at | E |
| ENSMUSP00000004968 | 160742\_at | E |
| ENSMUSP00000022124 | 100730\_at | E |
| ENSMUSP00000006838 | 94868\_at | U |
| ENSMUSP00000046969 | 96035\_at | U |
| ENSMUSP00000024059 | 97757\_at | M |
| ENSMUSP00000028904 | 102296\_at | U |
| ENSMUSP00000022324 | 92767\_at | E |
| ENSMUSP00000001562 | 102308\_at | E |
| ENSMUSP00000008517 | 103885\_at | U |
| ENSMUSP00000034509 | 99057\_at | M |
| ENSMUSP00000034539 | 94067\_at | E |
| ENSMUSP00000020400 | 160074\_at | U |
| ENSMUSP00000051250 | 95523\_at | E |
| ENSMUSP00000030155 | 101651\_at | M |
| ENSMUSP00000020775 | 101929\_at | E |
| ENSMUSP00000004375 | 98545\_at | U |
| ENSMUSP00000023717 | 93555\_at | E |
| ENSMUSP00000032658 | 103084\_at | E |
| ENSMUSP00000027451 | 104673\_at | Z |
| ENSMUSP00000020978 | 103229\_at | Z |
| ENSMUSP00000061045 | 93688\_at | M |
| ENSMUSP00000053495 | 96311\_at | M |
| ENSMUSP00000015853 | 92910\_at | Z |
| ENSMUSP00000022221 | 102572\_at | E |
| ENSMUSP00000027874 | 99333\_at | Z |
| ENSMUSP00000020440 | 94210\_at | E |
| ENSMUSP00000053540 | 160350\_at | U |
| ENSMUSP00000050797 | 99466\_at | M |
| ENSMUSP00000033634 | 104017\_at | U |
| ENSMUSP00000006544 | 160628\_at | U |
| ENSMUSP00000025641 | 95932\_at | M |
| ENSMUSP00000017318 | 100471\_at | Z |
| ENSMUSP00000031073 | 100616\_at | E |
| ENSMUSP00000054266 | 100749\_at | Z |
| ENSMUSP00000006952 | 92242\_at | M |
| ENSMUSP00000031648 | 98821\_at | Z |
| ENSMUSP00000052351 | 92375\_at | E |
| ENSMUSP00000019721 | 102049\_at | U |
| ENSMUSP00000026817 | 103493\_at | M |
| ENSMUSP00000001513 | 160461\_f\_at | E |
| ENSMUSP00000033321 | 102248\_f\_at | E |
| ENSMUSP00000047700 | 101392\_at | E |
| ENSMUSP00000042918 | 101404\_at | M |
| ENSMUSP00000052874 | 96986\_at | M |
| ENSMUSP00000020741 | 98020\_at | U |
| ENSMUSP00000052823 | 101537\_at | M |
| ENSMUSP00000043069 | 93030\_at | E |
| ENSMUSP00000007259 | 98286\_at | M |
| ENSMUSP00000020713 | 93163\_at | Z |
| ENSMUSP00000022691 | 99875\_at | E |
| ENSMUSP00000036066 | 93296\_at | M |
| ENSMUSP00000000901 | 160892\_at | U |
| ENSMUSP00000055516 | 94885\_at | E |
| ENSMUSP00000049934 | 160904\_at | Z |
| ENSMUSP00000026120 | 104559\_at | M |
| ENSMUSP00000020420 | 96185\_at | E |
| ENSMUSP00000060590 | 97774\_at | Z |
| ENSMUSP00000029479 | 97919\_at | M |
| ENSMUSP00000028430 | 92929\_at | U |
| ENSMUSP00000026129 | 103914\_at | U |
| ENSMUSP00000027366 | 99074\_at | E |
| ENSMUSP00000056325 | 160091\_at | U |
| ENSMUSP00000009545 | 94084\_at | M |
| ENSMUSP00000028354 | 160103\_at | E |
| ENSMUSP00000031737 | 94229\_at | Z |
| ENSMUSP00000048836 | 98562\_at | M |
| ENSMUSP00000027111 | 93572\_at | U |
| ENSMUSP00000000194 | 93717\_at | M |
| ENSMUSP00000020767 | 104690\_at | U |
| ENSMUSP00000025170 | 104702\_at | E |
| ENSMUSP00000013481 | 101145\_at | M |
| ENSMUSP00000030264 | 101278\_at | M |
| ENSMUSP00000019937 | 99350\_at | U |
| ENSMUSP00000052908 | 98039\_at | M |
| ENSMUSP00000047235 | 94360\_at | Z |
| ENSMUSP00000021562 | 99628\_at | U |
| ENSMUSP00000036088 | 94493\_at | M |
| ENSMUSP00000042277 | 160512\_at | M |
| ENSMUSP00000020342 | 94505\_at | Z |
| ENSMUSP00000013949 | 160645\_at | E |
| ENSMUSP00000033394 | 100500\_at | M |
| ENSMUSP00000062237 | 160908\_r\_at | Z |
| ENSMUSP00000029030 | 100766\_at | M |
| ENSMUSP00000023015 | 92404\_at | Z |
| ENSMUSP00000058674 | 97527\_at | E |
| ENSMUSP00000020613 | 103522\_at | Z |
| ENSMUSP00000033941 | 93981\_at | Z |
| ENSMUSP00000029752 | 102199\_at | U |
| ENSMUSP00000037981 | 103655\_at | E |
| ENSMUSP00000026538 | 95426\_at | U |
| ENSMUSP00000023116 | 96870\_at | U |
| ENSMUSP00000018595 | 95559\_at | M |
| ENSMUSP00000015622 | 101421\_at | E |
| ENSMUSP00000021413 | 101554\_at | U |
| ENSMUSP00000058713 | 92192\_s\_at | E |
| ENSMUSP00000004786 | 93325\_at | E |
| ENSMUSP00000055797 | 99892\_at | Z |
| ENSMUSP00000021028 | 99904\_at | E |
| ENSMUSP00000028944 | 160921\_at | U |
| ENSMUSP00000040588 | 104443\_at | Z |
| ENSMUSP00000062918 | 93458\_at | Z |
| ENSMUSP00000030917 | 104576\_at | M |
| ENSMUSP00000033775 | 97803\_at | U |
| ENSMUSP00000060446 | 97791\_at | Z |
| ENSMUSP00000048947 | 102342\_at | U |
| ENSMUSP00000038536 | 103931\_at | Z |
| ENSMUSP00000003284 | 99103\_at | M |
| ENSMUSP00000028306 | 99236\_at | M |
| ENSMUSP00000023612 | 94246\_at | Z |
| ENSMUSP00000026565 | 160253\_at | M |
| ENSMUSP00000022102 | 160386\_at | E |
| ENSMUSP00000035010 | 95702\_at | U |
| ENSMUSP00000023071 | 95690\_at | U |
| ENSMUSP00000025755 | 100374\_at | Z |
| ENSMUSP00000028546 | 101830\_at | E |
| ENSMUSP00000021933 | 101963\_at | E |
| ENSMUSP00000025087 | 103263\_at | E |
| ENSMUSP00000047016 | 98857\_at | Z |
| ENSMUSP00000021666 | 93867\_at | U |
| ENSMUSP00000052256 | 103408\_at | E |
| ENSMUSP00000000445 | 101162\_at | Z |
| ENSMUSP00000029925 | 96756\_at | Z |
| ENSMUSP00000003687 | 102751\_at | Z |
| ENSMUSP00000030483 | 101307\_at | U |
| ENSMUSP00000033958 | 100526\_f\_at | E |
| ENSMUSP00000044647 | 102884\_at | E |
| ENSMUSP00000040614 | 98056\_at | M |
| ENSMUSP00000041792 | 99512\_at | E |
| ENSMUSP00000046340 | 93066\_at | E |
| ENSMUSP00000030158 | 94522\_at | M |
| ENSMUSP00000030883 | 104184\_at | M |
| ENSMUSP00000022197 | 160795\_at | E |
| ENSMUSP00000021052 | 161077\_f\_at | E |
| ENSMUSP00000001240 | 160807\_at | U |
| ENSMUSP00000038616 | 93004\_r\_at | E |
| ENSMUSP00000029248 | 97411\_at | E |
| ENSMUSP00000048334 | 100928\_at | Z |
| ENSMUSP00000004673 | 96088\_at | E |
| ENSMUSP00000022894 | 97544\_at | E |
| ENSMUSP00000028368 | 92421\_at | U |
| ENSMUSP00000033269 | 92554\_at | U |
| ENSMUSP00000018716 | 103672\_at | E |
| ENSMUSP00000035004 | 103817\_at | Z |
| ENSMUSP00000031338 | 95310\_at | M |
| ENSMUSP00000037007 | 160139\_at | Z |
| ENSMUSP00000004377 | 102851\_s\_at | E |
| ENSMUSP00000005019 | 100127\_at | Z |
| ENSMUSP00000041372 | 101716\_at | M |
| ENSMUSP00000027626 | 93342\_at | E |
| ENSMUSP00000021235 | 104460\_at | E |
| ENSMUSP00000028801 | 93475\_at | Z |
| ENSMUSP00000032956 | 104593\_at | E |
| ENSMUSP00000032272 | 104605\_at | U |
| ENSMUSP00000046168 | 96231\_at | U |
| ENSMUSP00000021114 | 97820\_at | U |
| ENSMUSP00000025583 | 96509\_at | M |
| ENSMUSP00000045260 | 94130\_at | Z |
| ENSMUSP00000047041 | 99386\_at | E |
| ENSMUSP00000002960 | 94408\_at | Z |
| ENSMUSP00000062593 | 94396\_at | E |
| ENSMUSP00000020763 | 100403\_at | E |
| ENSMUSP00000022504 | 100391\_at | E |
| ENSMUSP00000005960 | 92295\_at | Z |
| ENSMUSP00000004949 | 98874\_at | E |
| ENSMUSP00000007318 | 103558\_at | E |
| ENSMUSP00000027965 | 96640\_at | M |
| ENSMUSP00000033153 | 95329\_at | U |
| ENSMUSP00000030028 | 96773\_at | U |
| ENSMUSP00000021909 | 96918\_at | U |
| ENSMUSP00000025705 | 101457\_at | Z |
| ENSMUSP00000029266 | 93083\_at | E |
| ENSMUSP00000051717 | 104213\_at | U |
| ENSMUSP00000025965 | 93228\_at | U |
| ENSMUSP00000049124 | 104346\_at | U |
| ENSMUSP00000036765 | 104479\_at | Z |
| ENSMUSP00000058832 | 103701\_at | M |
| ENSMUSP00000032383 | 102378\_at | M |
| ENSMUSP00000019071 | 92849\_at | M |
| ENSMUSP00000027050 | 95460\_at | E |
| ENSMUSP00000034470 | 99139\_at | E |
| ENSMUSP00000030383 | 94149\_at | Z |
| ENSMUSP00000023010 | 160156\_at | Z |
| ENSMUSP00000022039 | 95593\_at | M |
| ENSMUSP00000027438 | 100144\_at | E |
| ENSMUSP00000025979 | 95738\_at | U |
| ENSMUSP00000047894 | 100277\_at | Z |
| ENSMUSP00000055009 | 101733\_at | Z |
| ENSMUSP00000040442 | 101866\_at | E |
| ENSMUSP00000006005 | 98482\_at | Z |
| ENSMUSP00000051423 | 93492\_at | E |
| ENSMUSP00000055491 | 98627\_at | M |
| ENSMUSP00000056119 | 104622\_at | E |
| ENSMUSP00000025571 | 93637\_at | M |
| ENSMUSP00000051125 | 103299\_at | Z |
| ENSMUSP00000018482 | 104755\_at | M |
| ENSMUSP00000023464 | 97970\_at | Z |
| ENSMUSP00000053594 | 101065\_at | U |
| ENSMUSP00000008090 | 92980\_at | Z |
| ENSMUSP00000038502 | 101198\_at | Z |
| ENSMUSP00000033502 | 102654\_at | E |
| ENSMUSP00000057963 | 102787\_at | Z |
| ENSMUSP00000039660 | 99415\_at | Z |
| ENSMUSP00000039406 | 160432\_at | E |
| ENSMUSP00000019246 | 99548\_at | U |
| ENSMUSP00000021860 | 94425\_at | M |
| ENSMUSP00000060377 | 160565\_at | U |
| ENSMUSP00000020307 | 104087\_at | M |
| ENSMUSP00000021761 | 100553\_at | Z |
| ENSMUSP00000052316 | 92324\_at | Z |
| ENSMUSP00000060631 | 98891\_at | M |
| ENSMUSP00000029128 | 97447\_at | E |
| ENSMUSP00000022639 | 103575\_at | Z |
| ENSMUSP00000035515 | 96802\_at | M |
| ENSMUSP00000025325 | 101341\_at | M |
| ENSMUSP00000000594 | 95479\_at | E |
| ENSMUSP00000030488 | 96935\_at | M |
| ENSMUSP00000048765 | 101474\_at | Z |
| ENSMUSP00000051869 | 102930\_at | M |
| ENSMUSP00000055333 | 98102\_at | U |
| ENSMUSP00000002949 | 93112\_at | U |
| ENSMUSP00000031456 | 94701\_at | E |
| ENSMUSP00000003359 | 160841\_at | Z |
| ENSMUSP00000017881 | 99957\_at | E |
| ENSMUSP00000001703 | 93378\_at | Z |
| ENSMUSP00000027777 | 101957\_f\_at | E |
| ENSMUSP00000034915 | 94834\_at | E |
| ENSMUSP00000035400 | 104508\_at | E |
| ENSMUSP00000026469 | 100962\_at | Z |
| ENSMUSP00000043722 | 96134\_at | E |
| ENSMUSP00000033123 | 103273\_s\_at | U |
| ENSMUSP00000042967 | 96267\_at | U |
| ENSMUSP00000056368 | 92733\_at | E |
| ENSMUSP00000022355 | 97989\_at | U |
| ENSMUSP00000018361 | 102395\_at | M |
| ENSMUSP00000003215 | 99023\_at | U |
| ENSMUSP00000046105 | 92866\_at | M |
| ENSMUSP00000051068 | 99156\_at | Z |
| ENSMUSP00000021362 | 95622\_at | E |
| ENSMUSP00000014457 | 100550\_f\_at | Z |
| ENSMUSP00000040431 | 160318\_at | U |
| ENSMUSP00000058503 | 95755\_at | U |
| ENSMUSP00000051479 | 95888\_at | M |
| ENSMUSP00000030893 | 100294\_at | Z |
| ENSMUSP00000056816 | 100306\_at | Z |
| ENSMUSP00000058105 | 98511\_at | E |
| ENSMUSP00000053178 | 103050\_at | Z |
| ENSMUSP00000035115 | 98777\_at | Z |
| ENSMUSP00000023167 | 93654\_at | Z |
| ENSMUSP00000045318 | 103328\_at | M |
| ENSMUSP00000041196 | 96676\_at | E |
| ENSMUSP00000037431 | 99432\_at | E |
| ENSMUSP00000021508 | 102816\_at | U |
| ENSMUSP00000028718 | 160582\_at | Z |
| ENSMUSP00000054322 | 104116\_at | M |
| ENSMUSP00000013673 | 160727\_at | M |
| ENSMUSP00000030796 | 100570\_at | E |
| ENSMUSP00000027488 | 97331\_at | E |
| ENSMUSP00000022464 | 100715\_at | M |
| ENSMUSP00000046118 | 92341\_at | E |
| ENSMUSP00000034923 | 103592\_at | E |
| ENSMUSP00000032111 | 92619\_at | M |
| ENSMUSP00000020629 | 103737\_at | U |
| ENSMUSP00000020549 | 95363\_at | Z |
| ENSMUSP00000021153 | 95496\_at | U |
| ENSMUSP00000021412 | 96952\_at | U |
| ENSMUSP00000028727 | 100047\_at | E |
| ENSMUSP00000060672 | 98385\_at | E |
| ENSMUSP00000050444 | 99841\_at | Z |
| ENSMUSP00000029970 | 104380\_at | E |
| ENSMUSP00000002850 | 93407\_at | U |
| ENSMUSP00000022294 | 160991\_at | E |
| ENSMUSP00000020489 | 104525\_at | E |
| ENSMUSP00000003435 | 96284\_at | U |
| ENSMUSP00000055694 | 160373\_i\_at | M |
| ENSMUSP00000021195 | 99040\_at | U |
| ENSMUSP00000001008 | 102424\_at | M |
| ENSMUSP00000033313 | 160202\_at | Z |
| ENSMUSP00000025110 | 160190\_at | E |
| ENSMUSP00000061716 | 94183\_at | M |
| ENSMUSP00000057466 | 100899\_s\_at | Z |
| ENSMUSP00000029769 | 160335\_at | E |
| ENSMUSP00000041912 | 93671\_at | Z |
| ENSMUSP00000021592 | 103212\_at | E |
| ENSMUSP00000016498 | 98794\_at | Z |
| ENSMUSP00000015464 | 103345\_at | U |
| ENSMUSP00000029212 | 93949\_at | U |
| ENSMUSP00000022293 | 160529\_r\_at | E |
| ENSMUSP00000018992 | 96693\_at | U |
| ENSMUSP00000025823 | 96838\_at | E |
| ENSMUSP00000046787 | 102700\_at | Z |
| ENSMUSP00000061935 | 99582\_at | M |
| ENSMUSP00000049315 | 104000\_at | U |
| ENSMUSP00000024769 | 98138\_at | U |
| ENSMUSP00000027067 | 93015\_at | U |
| ENSMUSP00000061360 | 93148\_at | Z |
| ENSMUSP00000034063 | 160611\_at | U |
| ENSMUSP00000023007 | 94737\_at | U |
| ENSMUSP00000062904 | 100732\_at | U |
| ENSMUSP00000041008 | 100998\_at | M |
| ENSMUSP00000024906 | 102032\_at | Z |
| ENSMUSP00000040345 | 102298\_at | M |
| ENSMUSP00000042083 | 100342\_i\_at | E |
| ENSMUSP00000017365 | 92769\_at | E |
| ENSMUSP00000049567 | 101551\_s\_at | E |
| ENSMUSP00000029538 | 103887\_at | M |
| ENSMUSP00000004829 | 95380\_at | M |
| ENSMUSP00000003135 | 99059\_at | Z |
| ENSMUSP00000028511 | 160076\_at | E |
| ENSMUSP00000036964 | 101520\_at | M |
| ENSMUSP00000037958 | 101786\_at | U |
| ENSMUSP00000062066 | 98547\_at | U |
| ENSMUSP00000039012 | 103086\_at | Z |
| ENSMUSP00000059519 | 93557\_at | U |
| ENSMUSP00000034912 | 96313\_at | E |
| ENSMUSP00000033811 | 161009\_at | E |
| ENSMUSP00000020145 | 97890\_at | E |
| ENSMUSP00000045901 | 99925\_f\_at | E |
| ENSMUSP00000057312 | 92912\_at | Z |
| ENSMUSP00000011285 | 102574\_at | Z |
| ENSMUSP00000052349 | 99190\_at | Z |
| ENSMUSP00000024260 | 160352\_at | E |
| ENSMUSP00000017975 | 94478\_at | E |
| ENSMUSP00000031229 | 161062\_r\_at | E |
| ENSMUSP00000039376 | 92244\_at | U |
| ENSMUSP00000037192 | 98823\_at | Z |
| ENSMUSP00000048736 | 103362\_at | Z |
| ENSMUSP00000004850 | 103507\_at | Z |
| ENSMUSP00000030801 | 93966\_at | E |
| ENSMUSP00000031766 | 95133\_at | U |
| ENSMUSP00000041152 | 96855\_at | Z |
| ENSMUSP00000047508 | 98022\_at | U |
| ENSMUSP00000023480 | 102850\_at | Z |
| ENSMUSP00000058868 | 101406\_at | U |
| ENSMUSP00000035245 | 103664\_r\_at | M |
| ENSMUSP00000007161 | 98288\_at | Z |
| ENSMUSP00000054508 | 160761\_at | M |
| ENSMUSP00000029666 | 93298\_at | U |
| ENSMUSP00000029852 | 94754\_at | E |
| ENSMUSP00000051754 | 100882\_at | M |
| ENSMUSP00000042249 | 96187\_at | E |
| ENSMUSP00000048368 | 97173\_f\_at | M |
| ENSMUSP00000034798 | 97776\_at | Z |
| ENSMUSP00000057742 | 103771\_at | E |
| ENSMUSP00000027472 | 92786\_at | E |
| ENSMUSP00000017316 | 102327\_at | E |
| ENSMUSP00000033210 | 103916\_at | M |
| ENSMUSP00000019058 | 94086\_at | M |
| ENSMUSP00000025918 | 100081\_at | U |
| ENSMUSP00000025089 | 95675\_at | Z |
| ENSMUSP00000027970 | 98431\_at | E |
| ENSMUSP00000032125 | 101815\_at | Z |
| ENSMUSP00000059474 | 93574\_at | U |
| ENSMUSP00000020964 | 103248\_at | U |
| ENSMUSP00000027870 | 104692\_at | Z |
| ENSMUSP00000053471 | 93719\_at | Z |
| ENSMUSP00000001713 | 95019\_at | U |
| ENSMUSP00000061149 | 94100\_s\_at | E |
| ENSMUSP00000037888 | 100434\_s\_at | M |
| ENSMUSP00000027975 | 96608\_at | U |
| ENSMUSP00000023339 | 101147\_at | M |
| ENSMUSP00000000193 | 102736\_at | M |
| ENSMUSP00000003154 | 102869\_at | Z |
| ENSMUSP00000028332 | 104036\_at | E |
| ENSMUSP00000034046 | 94507\_at | U |
| ENSMUSP00000033458 | 92665\_f\_at | M |
| ENSMUSP00000034763 | 160514\_at | M |
| ENSMUSP00000032240 | 95951\_at | Z |
| ENSMUSP00000030817 | 160647\_at | U |
| ENSMUSP00000034927 | 104169\_at | E |
| ENSMUSP00000053285 | 100635\_at | E |
| ENSMUSP00000046977 | 97384\_at | E |
| ENSMUSP00000028137 | 92261\_at | E |
| ENSMUSP00000015547 | 98840\_at | Z |
| ENSMUSP00000022519 | 97529\_at | E |
| ENSMUSP00000026159 | 92406\_at | M |
| ENSMUSP00000032751 | 93850\_at | E |
| ENSMUSP00000036949 | 92539\_at | M |
| ENSMUSP00000026208 | 95283\_at | U |
| ENSMUSP00000005067 | 96872\_at | U |
| ENSMUSP00000027303 | 95428\_at | E |
| ENSMUSP00000021962 | 101423\_at | U |
| ENSMUSP00000012664 | 98317\_at | Z |
| ENSMUSP00000055792 | 93327\_at | Z |
| ENSMUSP00000047322 | 104312\_at | U |
| ENSMUSP00000024210 | 99906\_at | Z |
| ENSMUSP00000031835 | 160923\_at | E |
| ENSMUSP00000001507 | 94916\_at | U |
| ENSMUSP00000023221 | 100911\_at | E |
| ENSMUSP00000027266 | 96216\_at | E |
| ENSMUSP00000002051 | 97793\_at | U |
| ENSMUSP00000026428 | 92670\_at | E |
| ENSMUSP00000003321 | 103800\_at | U |
| ENSMUSP00000019060 | 92948\_at | M |
| ENSMUSP00000002007 | 103933\_at | M |
| ENSMUSP00000062640 | 99093\_at | E |
| ENSMUSP00000024206 | 99238\_at | U |
| ENSMUSP00000051709 | 160122\_at | U |
| ENSMUSP00000056885 | 94115\_at | Z |
| ENSMUSP00000003117 | 94248\_at | E |
| ENSMUSP00000009234 | 95704\_at | E |
| ENSMUSP00000021133 | 95692\_at | E |
| ENSMUSP00000034015 | 160388\_at | E |
| ENSMUSP00000060230 | 97004\_at | M |
| ENSMUSP00000062423 | 93591\_at | E |
| ENSMUSP00000023391 | 93603\_at | Z |
| ENSMUSP00000034334 | 98726\_at | Z |
| ENSMUSP00000001383 | 98859\_at | E |
| ENSMUSP00000020710 | 93736\_at | M |
| ENSMUSP00000031503 | 103398\_at | E |
| ENSMUSP00000015934 | 101031\_at | U |
| ENSMUSP00000061965 | 102620\_at | U |
| ENSMUSP00000028024 | 101164\_at | M |
| ENSMUSP00000058040 | 101309\_at | Z |
| ENSMUSP00000043111 | 93987\_f\_at | E |
| ENSMUSP00000033450 | 102886\_at | Z |
| ENSMUSP00000057748 | 99514\_at | M |
| ENSMUSP00000029689 | 94524\_at | E |
| ENSMUSP00000022873 | 104053\_at | E |
| ENSMUSP00000027156 | 99647\_at | U |
| ENSMUSP00000004379 | 160531\_at | U |
| ENSMUSP00000025728 | 160664\_at | Z |
| ENSMUSP00000034571 | 97413\_at | M |
| ENSMUSP00000034433 | 98990\_at | E |
| ENSMUSP00000005842 | 92423\_at | Z |
| ENSMUSP00000032118 | 97679\_at | Z |
| ENSMUSP00000021630 | 92556\_at | U |
| ENSMUSP00000025133 | 160079\_i\_at | Z |
| ENSMUSP00000033309 | 92689\_at | M |
| ENSMUSP00000047656 | 103819\_at | Z |
| ENSMUSP00000001709 | 95312\_at | Z |
| ENSMUSP00000026989 | 95445\_at | M |
| ENSMUSP00000034519 | 98334\_at | Z |
| ENSMUSP00000053018 | 93211\_at | U |
| ENSMUSP00000006703 | 98467\_at | U |
| ENSMUSP00000044036 | 99923\_at | E |
| ENSMUSP00000031355 | 103018\_at | E |
| ENSMUSP00000026835 | 94933\_at | U |
| ENSMUSP00000032559 | 104607\_at | Z |
| ENSMUSP00000038121 | 92832\_at | Z |
| ENSMUSP00000047157 | 103950\_at | U |
| ENSMUSP00000061000 | 94132\_at | U |
| ENSMUSP00000050576 | 99388\_at | Z |
| ENSMUSP00000019906 | 100393\_at | M |
| ENSMUSP00000023707 | 100538\_at | U |
| ENSMUSP00000038305 | 98610\_at | Z |
| ENSMUSP00000023143 | 93753\_at | Z |
| ENSMUSP00000041135 | 103282\_at | E |
| ENSMUSP00000025836 | 98876\_at | U |
| ENSMUSP00000006020 | 101181\_at | U |
| ENSMUSP00000018810 | 96775\_at | E |
| ENSMUSP00000034153 | 102770\_at | M |
| ENSMUSP00000024627 | 101459\_at | U |
| ENSMUSP00000047035 | 99531\_at | Z |
| ENSMUSP00000054575 | 102915\_at | Z |
| ENSMUSP00000043033 | 98075\_at | E |
| ENSMUSP00000052203 | 93085\_at | U |
| ENSMUSP00000021535 | 160681\_at | E |
| ENSMUSP00000004340 | 99809\_at | Z |
| ENSMUSP00000056604 | 93889\_f\_at | E |
| ENSMUSP00000029910 | 160826\_at | U |
| ENSMUSP00000034624 | 97430\_at | U |
| ENSMUSP00000046890 | 104314\_r\_at | M |
| ENSMUSP00000016336 | 92440\_at | M |
| ENSMUSP00000007708 | 92573\_at | E |
| ENSMUSP00000028728 | 102247\_at | E |
| ENSMUSP00000048566 | 103969\_at | M |
| ENSMUSP00000020856 | 95462\_at | E |
| ENSMUSP00000036003 | 95595\_at | U |
| ENSMUSP00000018311 | 95607\_at | Z |
| ENSMUSP00000010502 | 100013\_at | M |
| ENSMUSP00000034197 | 100279\_at | E |
| ENSMUSP00000008123 | 98484\_at | Z |
| ENSMUSP00000006090 | 99940\_at | Z |
| ENSMUSP00000059093 | 103035\_at | U |
| ENSMUSP00000028290 | 104624\_at | E |
| ENSMUSP00000034803 | 104757\_at | E |
| ENSMUSP00000019854 | 101067\_at | U |
| ENSMUSP00000037779 | 92982\_at | Z |
| ENSMUSP00000015197 | 102789\_at | E |
| ENSMUSP00000034000 | 94282\_at | Z |
| ENSMUSP00000025270 | 160301\_at | U |
| ENSMUSP00000027440 | 99417\_at | Z |
| ENSMUSP00000033333 | 160434\_at | Z |
| ENSMUSP00000018382 | 160567\_at | Z |
| ENSMUSP00000054730 | 100555\_at | Z |
| ENSMUSP00000031295 | 100688\_at | M |
| ENSMUSP00000051339 | 92181\_at | Z |
| ENSMUSP00000023559 | 97316\_at | U |
| ENSMUSP00000026284 | 98760\_at | Z |
| ENSMUSP00000031634 | 93770\_at | U |
| ENSMUSP00000046609 | 103311\_at | E |
| ENSMUSP00000058218 | 98905\_at | E |
| ENSMUSP00000009329 | 92459\_at | M |
| ENSMUSP00000034554 | 93915\_at | Z |
| ENSMUSP00000020265 | 103444\_at | U |
| ENSMUSP00000047123 | 96804\_at | U |
| ENSMUSP00000027984 | 101343\_at | Z |
| ENSMUSP00000022808 | 101476\_at | U |
| ENSMUSP00000031264 | 98092\_at | M |
| ENSMUSP00000047682 | 98104\_at | U |
| ENSMUSP00000023712 | 94703\_at | E |
| ENSMUSP00000026507 | 160710\_at | E |
| ENSMUSP00000062716 | 104232\_at | M |
| ENSMUSP00000038350 | 104365\_at | E |
| ENSMUSP00000056498 | 104498\_at | Z |
| ENSMUSP00000003773 | 96003\_at | Z |
| ENSMUSP00000054026 | 96136\_at | U |
| ENSMUSP00000021549 | 100964\_at | E |
| ENSMUSP00000049063 | 97580\_at | E |
| ENSMUSP00000029468 | 92590\_at | U |
| ENSMUSP00000020957 | 103429\_i\_at | U |
| ENSMUSP00000050917 | 96269\_at | U |
| ENSMUSP00000029948 | 97725\_at | E |
| ENSMUSP00000008021 | 92602\_at | M |
| ENSMUSP00000046326 | 92735\_at | Z |
| ENSMUSP00000047782 | 102264\_at | M |
| ENSMUSP00000051920 | 103720\_at | Z |
| ENSMUSP00000006525 | 102397\_at | Z |
| ENSMUSP00000047898 | 99025\_at | U |
| ENSMUSP00000026472 | 103986\_at | Z |
| ENSMUSP00000025631 | 99158\_at | U |
| ENSMUSP00000020677 | 100030\_at | U |
| ENSMUSP00000020650 | 94168\_at | M |
| ENSMUSP00000051784 | 100308\_at | M |
| ENSMUSP00000024955 | 98513\_at | U |
| ENSMUSP00000057944 | 98779\_at | M |
| ENSMUSP00000036582 | 161096\_at | Z |
| ENSMUSP00000022821 | 96678\_at | U |
| ENSMUSP00000062128 | 94042\_f\_at | Z |
| ENSMUSP00000025218 | 160451\_at | U |
| ENSMUSP00000054593 | 104118\_at | E |
| ENSMUSP00000026624 | 100572\_at | E |
| ENSMUSP00000018851 | 97333\_at | E |
| ENSMUSP00000031356 | 100717\_at | M |
| ENSMUSP00000033846 | 92210\_at | Z |
| ENSMUSP00000021850 | 102017\_at | E |
| ENSMUSP00000032211 | 92476\_at | Z |
| ENSMUSP00000026256 | 103594\_at | E |
| ENSMUSP00000049407 | 95232\_at | E |
| ENSMUSP00000030675 | 95498\_at | U |
| ENSMUSP00000016138 | 98121\_at | E |
| ENSMUSP00000028963 | 101505\_at | E |
| ENSMUSP00000021390 | 93131\_at | Z |
| ENSMUSP00000020846 | 93264\_at | E |
| ENSMUSP00000001652 | 98387\_at | Z |
| ENSMUSP00000051303 | 94720\_at | Z |
| ENSMUSP00000049909 | 93397\_at | Z |
| ENSMUSP00000022512 | 160860\_at | Z |
| ENSMUSP00000037211 | 160993\_at | E |
| ENSMUSP00000044433 | 94986\_at | Z |
| ENSMUSP00000028795 | 104527\_at | U |
| ENSMUSP00000040246 | 96020\_at | M |
| ENSMUSP00000035061 | 96153\_at | M |
| ENSMUSP00000060438 | 100981\_at | U |
| ENSMUSP00000030146 | 96286\_at | Z |
| ENSMUSP00000050076 | 97875\_at | E |
| ENSMUSP00000061405 | 92885\_at | M |
| ENSMUSP00000042705 | 99175\_at | Z |
| ENSMUSP00000056492 | 160192\_at | U |
| ENSMUSP00000025908 | 94185\_at | U |
| ENSMUSP00000056665 | 95641\_at | M |
| ENSMUSP00000018653 | 160204\_at | U |
| ENSMUSP00000030205 | 101222\_f\_at | Z |
| ENSMUSP00000027948 | 101914\_at | E |
| ENSMUSP00000002923 | 93540\_at | U |
| ENSMUSP00000022262 | 92229\_at | Z |
| ENSMUSP00000026455 | 98796\_at | U |
| ENSMUSP00000046512 | 93673\_at | M |
| ENSMUSP00000041373 | 98808\_at | Z |
| ENSMUSP00000027368 | 96562\_at | U |
| ENSMUSP00000016452 | 96695\_at | E |
| ENSMUSP00000056046 | 96707\_at | M |
| ENSMUSP00000048587 | 102690\_at | E |
| ENSMUSP00000025193 | 98066\_r\_at | E |
| ENSMUSP00000018522 | 102702\_at | Z |
| ENSMUSP00000024575 | 98007\_at | E |
| ENSMUSP00000006507 | 102968\_at | U |
| ENSMUSP00000044731 | 99451\_at | Z |
| ENSMUSP00000033731 | 104002\_at | M |
| ENSMUSP00000020886 | 94461\_at | U |
| ENSMUSP00000029912 | 93017\_at | U |
| ENSMUSP00000028644 | 99584\_at | E |
| ENSMUSP00000026009 | 104135\_at | E |
| ENSMUSP00000030560 | 160613\_at | E |
| ENSMUSP00000023515 | 100601\_at | E |
| ENSMUSP00000000498 | 100734\_at | U |
| ENSMUSP00000028050 | 92360\_at | E |
| ENSMUSP00000016396 | 93596\_i\_at | Z |
| ENSMUSP00000017548 | 97483\_at | U |
| ENSMUSP00000017151 | 92493\_at | E |
| ENSMUSP00000023081 | 94539\_f\_at | E |
| ENSMUSP00000063120 | 160078\_at | Z |
| ENSMUSP00000002914 | 95527\_at | E |
| ENSMUSP00000038745 | 100066\_at | U |
| ENSMUSP00000032673 | 101655\_at | M |
| ENSMUSP00000034871 | 93281\_at | E |
| ENSMUSP00000010205 | 99860\_at | E |
| ENSMUSP00000028311 | 98416\_at | E |
| ENSMUSP00000017488 | 98549\_at | Z |
| ENSMUSP00000035943 | 99993\_at | U |
| ENSMUSP00000032163 | 103088\_at | Z |
| ENSMUSP00000042602 | 93559\_at | U |
| ENSMUSP00000041794 | 104544\_at | U |
| ENSMUSP00000036996 | 104677\_at | E |
| ENSMUSP00000003876 | 97892\_at | E |
| ENSMUSP00000034231 | 102310\_at | M |
| ENSMUSP00000027579 | 97904\_at | E |
| ENSMUSP00000032456 | 102576\_at | Z |
| ENSMUSP00000030557 | 94214\_at | Z |
| ENSMUSP00000051137 | 160221\_at | Z |
| ENSMUSP00000022330 | 160354\_at | Z |
| ENSMUSP00000018800 | 160487\_at | E |
| ENSMUSP00000001384 | 99942\_s\_at | E |
| ENSMUSP00000000284 | 100475\_at | E |
| ENSMUSP00000021844 | 97091\_at | E |
| ENSMUSP00000021990 | 101931\_at | E |
| ENSMUSP00000031106 | 103231\_at | E |
| ENSMUSP00000054882 | 92246\_at | M |
| ENSMUSP00000039580 | 93702\_at | E |
| ENSMUSP00000052248 | 98825\_at | M |
| ENSMUSP00000052905 | 98958\_at | E |
| ENSMUSP00000030434 | 93835\_at | U |
| ENSMUSP00000060558 | 103497\_at | Z |
| ENSMUSP00000030808 | 103509\_at | M |
| ENSMUSP00000032577 | 93968\_at | U |
| ENSMUSP00000031668 | 101130\_at | E |
| ENSMUSP00000021016 | 101396\_at | M |
| ENSMUSP00000025166 | 102852\_at | Z |
| ENSMUSP00000020359 | 101408\_at | E |
| ENSMUSP00000004987 | 102985\_at | U |
| ENSMUSP00000057917 | 99613\_at | U |
| ENSMUSP00000031222 | 94623\_at | Z |
| ENSMUSP00000030670 | 104152\_at | E |
| ENSMUSP00000006128 | 160896\_at | E |
| ENSMUSP00000024897 | 94889\_at | E |
| ENSMUSP00000040244 | 100884\_at | U |
| ENSMUSP00000002303 | 96056\_at | E |
| ENSMUSP00000021719 | 97512\_at | M |
| ENSMUSP00000018383 | 96189\_at | E |
| ENSMUSP00000054374 | 92522\_at | Z |
| ENSMUSP00000051012 | 92655\_at | E |
| ENSMUSP00000001497 | 102329\_at | Z |
| ENSMUSP00000020458 | 99078\_at | U |
| ENSMUSP00000023616 | 103918\_at | U |
| ENSMUSP00000025409 | 160095\_at | Z |
| ENSMUSP00000032899 | 95411\_at | M |
| ENSMUSP00000061187 | 160107\_at | U |
| ENSMUSP00000030553 | 95677\_at | U |
| ENSMUSP00000004560 | 98433\_at | M |
| ENSMUSP00000022380 | 93310\_at | U |
| ENSMUSP00000024106 | 93443\_at | M |
| ENSMUSP00000028157 | 104561\_at | E |
| ENSMUSP00000020182 | 104706\_at | E |
| ENSMUSP00000025983 | 96598\_at | E |
| ENSMUSP00000020717 | 101016\_at | E |
| ENSMUSP00000014917 | 92931\_at | Z |
| ENSMUSP00000021898 | 101149\_at | Z |
| ENSMUSP00000032888 | 160371\_at | Z |
| ENSMUSP00000055593 | 96766\_s\_at | Z |
| ENSMUSP00000026666 | 160516\_at | U |
| ENSMUSP00000023460 | 94509\_at | U |
| ENSMUSP00000059270 | 160649\_at | Z |
| ENSMUSP00000000844 | 97253\_at | Z |
| ENSMUSP00000023958 | 92263\_at | M |
| ENSMUSP00000017148 | 98842\_at | M |
| ENSMUSP00000031266 | 103381\_at | E |
| ENSMUSP00000044160 | 98975\_at | E |
| ENSMUSP00000035759 | 92222\_f\_at | M |
| ENSMUSP00000030765 | 103526\_at | M |
| ENSMUSP00000032915 | 95118\_r\_at | E |
| ENSMUSP00000048051 | 93985\_at | Z |
| ENSMUSP00000044990 | 96741\_at | E |
| ENSMUSP00000058349 | 101280\_at | M |
| ENSMUSP00000042977 | 98041\_at | M |
| ENSMUSP00000029137 | 101425\_at | E |
| ENSMUSP00000022619 | 93051\_at | U |
| ENSMUSP00000039951 | 99630\_at | Z |
| ENSMUSP00000005508 | 93184\_at | U |
| ENSMUSP00000026432 | 94640\_at | Z |
| ENSMUSP00000007624 | 99896\_at | Z |
| ENSMUSP00000019481 | 94773\_at | Z |
| ENSMUSP00000031718 | 99908\_at | Z |
| ENSMUSP00000025921 | 99459\_f\_at | E |
| ENSMUSP00000034441 | 94918\_at | U |
| ENSMUSP00000025746 | 96073\_at | E |
| ENSMUSP00000047860 | 100913\_at | Z |
| ENSMUSP00000035498 | 96218\_at | E |
| ENSMUSP00000062272 | 92672\_at | E |
| ENSMUSP00000035102 | 102213\_at | M |
| ENSMUSP00000058261 | 97795\_at | Z |
| ENSMUSP00000006577 | 97807\_at | E |
| ENSMUSP00000045272 | 103790\_at | E |
| ENSMUSP00000034827 | 92817\_at | U |
| ENSMUSP00000025241 | 102346\_at | U |
| ENSMUSP00000003922 | 95561\_at | M |
| ENSMUSP00000037206 | 160257\_at | U |
| ENSMUSP00000051971 | 95706\_at | Z |
| ENSMUSP00000017078 | 95694\_at | E |
| ENSMUSP00000047104 | 101701\_at | Z |
| ENSMUSP00000032947 | 101834\_at | E |
| ENSMUSP00000025914 | 103001\_at | Z |
| ENSMUSP00000028181 | 93460\_at | E |
| ENSMUSP00000034557 | 98583\_at | Z |
| ENSMUSP00000030339 | 98728\_at | M |
| ENSMUSP00000030398 | 93738\_at | U |
| ENSMUSP00000037096 | 161045\_at | U |
| ENSMUSP00000033509 | 96627\_at | E |
| ENSMUSP00000042321 | 101166\_at | M |
| ENSMUSP00000040435 | 101299\_at | M |
| ENSMUSP00000043837 | 102755\_at | M |
| ENSMUSP00000038371 | 99371\_at | U |
| ENSMUSP00000002625 | 94381\_at | U |
| ENSMUSP00000034905 | 99649\_at | E |
| ENSMUSP00000043559 | 94526\_at | E |
| ENSMUSP00000020109 | 160666\_at | E |
| ENSMUSP00000053354 | 93350\_f\_at | M |
| ENSMUSP00000019210 | 97415\_at | E |
| ENSMUSP00000033230 | 102192\_r\_at | U |
| ENSMUSP00000046597 | 97548\_at | E |
| ENSMUSP00000029730 | 92425\_at | U |
| ENSMUSP00000001066 | 98992\_at | Z |
| ENSMUSP00000043008 | 102087\_at | Z |
| ENSMUSP00000052680 | 103676\_at | U |
| ENSMUSP00000046883 | 95314\_at | E |
| ENSMUSP00000030319 | 96891\_at | E |
| ENSMUSP00000002400 | 95447\_at | E |
| ENSMUSP00000014566 | 104272\_s\_at | E |
| ENSMUSP00000021277 | 98469\_at | E |
| ENSMUSP00000044012 | 94790\_at | U |
| ENSMUSP00000039635 | 93479\_at | M |
| ENSMUSP00000029937 | 104597\_at | E |
| ENSMUSP00000010195 | 102230\_at | Z |
| ENSMUSP00000001083 | 97824\_at | U |
| ENSMUSP00000003518 | 92834\_at | U |
| ENSMUSP00000028134 | 97957\_at | U |
| ENSMUSP00000000010 | 103952\_at | Z |
| ENSMUSP00000045140 | 94001\_at | E |
| ENSMUSP00000023734 | 94134\_at | Z |
| ENSMUSP00000056622 | 160274\_at | E |
| ENSMUSP00000054837 | 100395\_at | M |
| ENSMUSP00000024783 | 160227\_s\_at | E |
| ENSMUSP00000025842 | 100407\_at | M |
| ENSMUSP00000023341 | 101851\_at | M |
| ENSMUSP00000018729 | 101984\_at | E |
| ENSMUSP00000030788 | 97156\_at | Z |
| ENSMUSP00000052989 | 103284\_at | U |
| ENSMUSP00000023328 | 93755\_at | M |
| ENSMUSP00000027846 | 104740\_at | Z |
| ENSMUSP00000019117 | 93888\_at | Z |
| ENSMUSP00000019480 | 104495\_f\_at | Z |
| ENSMUSP00000027721 | 101183\_at | E |
| ENSMUSP00000055306 | 102772\_at | Z |
| ENSMUSP00000043819 | 102917\_at | E |
| ENSMUSP00000020397 | 98077\_at | E |
| ENSMUSP00000005826 | 99666\_at | U |
| ENSMUSP00000027824 | 104072\_at | M |
| ENSMUSP00000032944 | 104217\_at | U |
| ENSMUSP00000038147 | 100671\_at | M |
| ENSMUSP00000026972 | 100949\_at | Z |
| ENSMUSP00000017144 | 97698\_at | M |
| ENSMUSP00000034702 | 103560\_at | E |
| ENSMUSP00000025767 | 92575\_at | E |
| ENSMUSP00000026500 | 102249\_at | E |
| ENSMUSP00000029486 | 103838\_at | Z |
| ENSMUSP00000022338 | 95331\_at | Z |
| ENSMUSP00000006909 | 96583\_s\_at | Z |
| ENSMUSP00000006367 | 96920\_at | U |
| ENSMUSP00000059977 | 95597\_at | E |
| ENSMUSP00000056177 | 100015\_at | E |
| ENSMUSP00000057062 | 95609\_at | M |
| ENSMUSP00000051115 | 101096\_s\_at | M |
| ENSMUSP00000028533 | 101737\_at | M |
| ENSMUSP00000022624 | 98486\_at | M |
| ENSMUSP00000049161 | 103037\_at | M |
| ENSMUSP00000034904 | 93496\_at | E |
| ENSMUSP00000046595 | 104481\_at | Z |
| ENSMUSP00000043706 | 104626\_at | Z |
| ENSMUSP00000035086 | 96252\_at | E |
| ENSMUSP00000005711 | 97841\_at | E |
| ENSMUSP00000054328 | 101898\_s\_at | M |
| ENSMUSP00000059216 | 92851\_at | U |
| ENSMUSP00000000608 | 99141\_at | M |
| ENSMUSP00000005228 | 94151\_at | M |
| ENSMUSP00000018186 | 94284\_at | U |
| ENSMUSP00000011492 | 160303\_at | U |
| ENSMUSP00000046121 | 95740\_at | M |
| ENSMUSP00000054556 | 94429\_at | U |
| ENSMUSP00000046480 | 160569\_at | E |
| ENSMUSP00000003645 | 100424\_at | E |
| ENSMUSP00000028917 | 97318\_at | U |
| ENSMUSP00000059356 | 92328\_at | M |
| ENSMUSP00000054190 | 94752\_s\_at | Z |
| ENSMUSP00000028259 | 103446\_at | U |
| ENSMUSP00000036384 | 103579\_at | E |
| ENSMUSP00000005341 | 93917\_at | M |
| ENSMUSP00000023210 | 95072\_at | U |
| ENSMUSP00000059952 | 101212\_at | E |
| ENSMUSP00000059678 | 96794\_at | E |
| ENSMUSP00000053756 | 96806\_at | E |
| ENSMUSP00000015611 | 101345\_at | Z |
| ENSMUSP00000029160 | 96939\_at | E |
| ENSMUSP00000003029 | 98106\_at | E |
| ENSMUSP00000020477 | 160845\_at | U |
| ENSMUSP00000046286 | 160978\_at | Z |
| ENSMUSP00000023450 | 100966\_at | U |
| ENSMUSP00000023760 | 92592\_at | U |
| ENSMUSP00000061427 | 97727\_at | M |
| ENSMUSP00000040310 | 102266\_at | Z |
| ENSMUSP00000021784 | 92737\_at | M |
| ENSMUSP00000030746 | 94037\_at | Z |
| ENSMUSP00000030117 | 160177\_at | U |
| ENSMUSP00000058642 | 100032\_at | E |
| ENSMUSP00000032918 | 95759\_at | M |
| ENSMUSP00000024226 | 101621\_at | M |
| ENSMUSP00000002740 | 100298\_at | M |
| ENSMUSP00000040481 | 98370\_at | M |
| ENSMUSP00000034461 | 101887\_at | Z |
| ENSMUSP00000033993 | 98515\_at | E |
| ENSMUSP00000035847 | 104510\_at | U |
| ENSMUSP00000049595 | 93658\_at | E |
| ENSMUSP00000018993 | 104643\_at | Z |
| ENSMUSP00000041761 | 161098\_at | Z |
| ENSMUSP00000032399 | 97991\_at | E |
| ENSMUSP00000005073 | 102675\_at | M |
| ENSMUSP00000049534 | 99436\_at | M |
| ENSMUSP00000025056 | 94313\_at | E |
| ENSMUSP00000025973 | 160320\_at | E |
| ENSMUSP00000035142 | 160453\_at | Z |
| ENSMUSP00000029679 | 97468\_at | E |
| ENSMUSP00000038935 | 98924\_at | M |
| ENSMUSP00000048439 | 93801\_at | E |
| ENSMUSP00000040724 | 92478\_at | E |
| ENSMUSP00000023000 | 102019\_at | U |
| ENSMUSP00000051073 | 101754\_f\_at | M |
| ENSMUSP00000054219 | 93934\_at | M |
| ENSMUSP00000026414 | 103596\_at | E |
| ENSMUSP00000021857 | 103608\_at | U |
| ENSMUSP00000034775 | 160656\_i\_at | U |
| ENSMUSP00000020027 | 95101\_at | E |
| ENSMUSP00000042744 | 101362\_at | E |
| ENSMUSP00000032487 | 93652\_i\_at | E |
| ENSMUSP00000025904 | 96956\_at | U |
| ENSMUSP00000043768 | 101495\_at | Z |
| ENSMUSP00000060207 | 98123\_at | U |
| ENSMUSP00000005770 | 101507\_at | M |
| ENSMUSP00000033659 | 93133\_at | U |
| ENSMUSP00000029550 | 93266\_at | Z |
| ENSMUSP00000005490 | 99845\_at | U |
| ENSMUSP00000060956 | 160862\_at | E |
| ENSMUSP00000058759 | 97926\_s\_at | Z |
| ENSMUSP00000051618 | 93399\_at | M |
| ENSMUSP00000047536 | 94855\_at | U |
| ENSMUSP00000021963 | 104529\_at | M |
| ENSMUSP00000018813 | 96155\_at | E |
| ENSMUSP00000025490 | 96288\_at | M |
| ENSMUSP00000042326 | 92621\_at | E |
| ENSMUSP00000021078 | 92754\_at | U |
| ENSMUSP00000002588 | 102283\_at | E |
| ENSMUSP00000007255 | 92887\_at | Z |
| ENSMUSP00000060344 | 99044\_at | M |
| ENSMUSP00000003907 | 160194\_at | U |
| ENSMUSP00000035223 | 95643\_at | E |
| ENSMUSP00000021513 | 94187\_at | Z |
| ENSMUSP00000027802 | 160339\_at | U |
| ENSMUSP00000021139 | 101916\_at | E |
| ENSMUSP00000028063 | 93542\_at | U |
| ENSMUSP00000035164 | 103071\_at | E |
| ENSMUSP00000030180 | 104660\_at | M |
| ENSMUSP00000038838 | 103349\_at | Z |
| ENSMUSP00000015800 | 96564\_at | U |
| ENSMUSP00000035077 | 101115\_at | Z |
| ENSMUSP00000038970 | 96709\_at | M |
| ENSMUSP00000062764 | 99320\_at | E |
| ENSMUSP00000037454 | 94330\_at | U |
| ENSMUSP00000051432 | 93019\_at | E |
| ENSMUSP00000028852 | 160470\_at | U |
| ENSMUSP00000048997 | 104004\_at | M |
| ENSMUSP00000028938 | 99586\_at | M |
| ENSMUSP00000050259 | 104137\_at | U |
| ENSMUSP00000043462 | 160748\_at | U |
| ENSMUSP00000028400 | 100603\_at | E |
| ENSMUSP00000053945 | 100736\_at | Z |
| ENSMUSP00000049414 | 92362\_at | E |
| ENSMUSP00000031477 | 93951\_at | U |
| ENSMUSP00000044137 | 92495\_at | E |
| ENSMUSP00000024044 | 103480\_at | M |
| ENSMUSP00000036280 | 102036\_at | M |
| ENSMUSP00000008477 | 92839\_f\_at | E |
| ENSMUSP00000001520 | 103625\_at | U |
| ENSMUSP00000012206 | 99820\_f\_at | E |
| ENSMUSP00000034428 | 96840\_at | E |
| ENSMUSP00000020769 | 95529\_at | E |
| ENSMUSP00000025656 | 100068\_at | U |
| ENSMUSP00000002457 | 101657\_at | Z |
| ENSMUSP00000023583 | 99862\_at | M |
| ENSMUSP00000030948 | 98418\_at | Z |
| ENSMUSP00000057392 | 99995\_at | E |
| ENSMUSP00000020022 | 94872\_at | E |
| ENSMUSP00000059710 | 104679\_at | Z |
| ENSMUSP00000007244 | 97894\_at | U |
| ENSMUSP00000017566 | 92771\_at | E |
| ENSMUSP00000029390 | 97906\_at | E |
| ENSMUSP00000032980 | 92916\_at | E |
| ENSMUSP00000023431 | 103901\_at | E |
| ENSMUSP00000014174 | 102578\_at | Z |
| ENSMUSP00000020756 | 99194\_at | U |
| ENSMUSP00000002039 | 95660\_at | U |
| ENSMUSP00000013738 | 94216\_at | U |
| ENSMUSP00000024882 | 160223\_at | U |
| ENSMUSP00000030491 | 160356\_at | U |
| ENSMUSP00000021710 | 160489\_at | Z |
| ENSMUSP00000039821 | 95793\_at | M |
| ENSMUSP00000027374 | 160533\_r\_at | M |
| ENSMUSP00000023435 | 100477\_at | E |
| ENSMUSP00000027726 | 103922\_f\_at | U |
| ENSMUSP00000035994 | 97238\_at | Z |
| ENSMUSP00000057481 | 93704\_at | E |
| ENSMUSP00000028600 | 103233\_at | E |
| ENSMUSP00000023589 | 93837\_at | Z |
| ENSMUSP00000056504 | 95004\_at | E |
| ENSMUSP00000056237 | 95137\_at | E |
| ENSMUSP00000042405 | 101132\_at | E |
| ENSMUSP00000031640 | 96726\_at | Z |
| ENSMUSP00000004745 | 101398\_at | E |
| ENSMUSP00000040920 | 104021\_at | E |
| ENSMUSP00000003191 | 160632\_at | E |
| ENSMUSP00000009631 | 94625\_at | Z |
| ENSMUSP00000028162 | 160765\_at | E |
| ENSMUSP00000005336 | 104287\_at | E |
| ENSMUSP00000045888 | 160898\_at | E |
| ENSMUSP00000028004 | 96243\_f\_at | U |
| ENSMUSP00000026495 | 100753\_at | U |
| ENSMUSP00000034932 | 102053\_at | E |
| ENSMUSP00000052888 | 92524\_at | Z |
| ENSMUSP00000018727 | 103642\_at | E |
| ENSMUSP00000031038 | 95413\_at | Z |
| ENSMUSP00000025779 | 95679\_at | Z |
| ENSMUSP00000040741 | 95456\_r\_at | E |
| ENSMUSP00000043606 | 98302\_at | E |
| ENSMUSP00000061585 | 98290\_at | M |
